# Supplementary figures and images for: Tubulin glutamylation regulates axon guidance via the selective tuning of microtubule-severing enzymes (part 1 of 2)
Source: EMBO J. 2024 Nov 29;44(1):107–40. doi: 10.1038/s44318-024-00307-x (PMC11695996; doi:10.1038/s44318-024-00307-x)

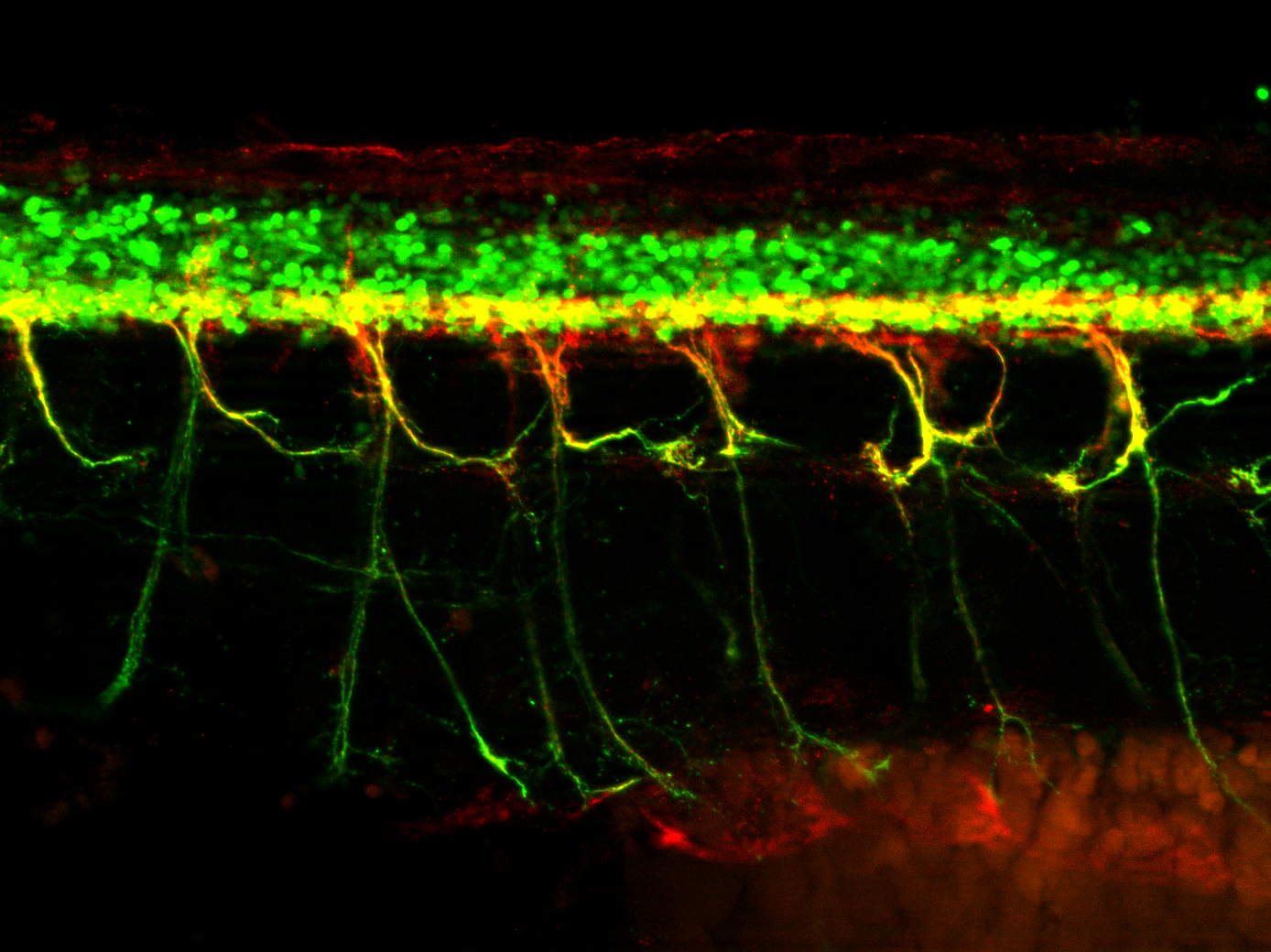

Supplement: Supplementary file 6 — Source data Fig. 1 [file 44318_2024_307_MOESM6_ESM.zip › EMBOJ-2024-116734_sourcedata_Fig 1/Fig1_panelA_MOKat3.4_sMN_zn5GFP.tif]

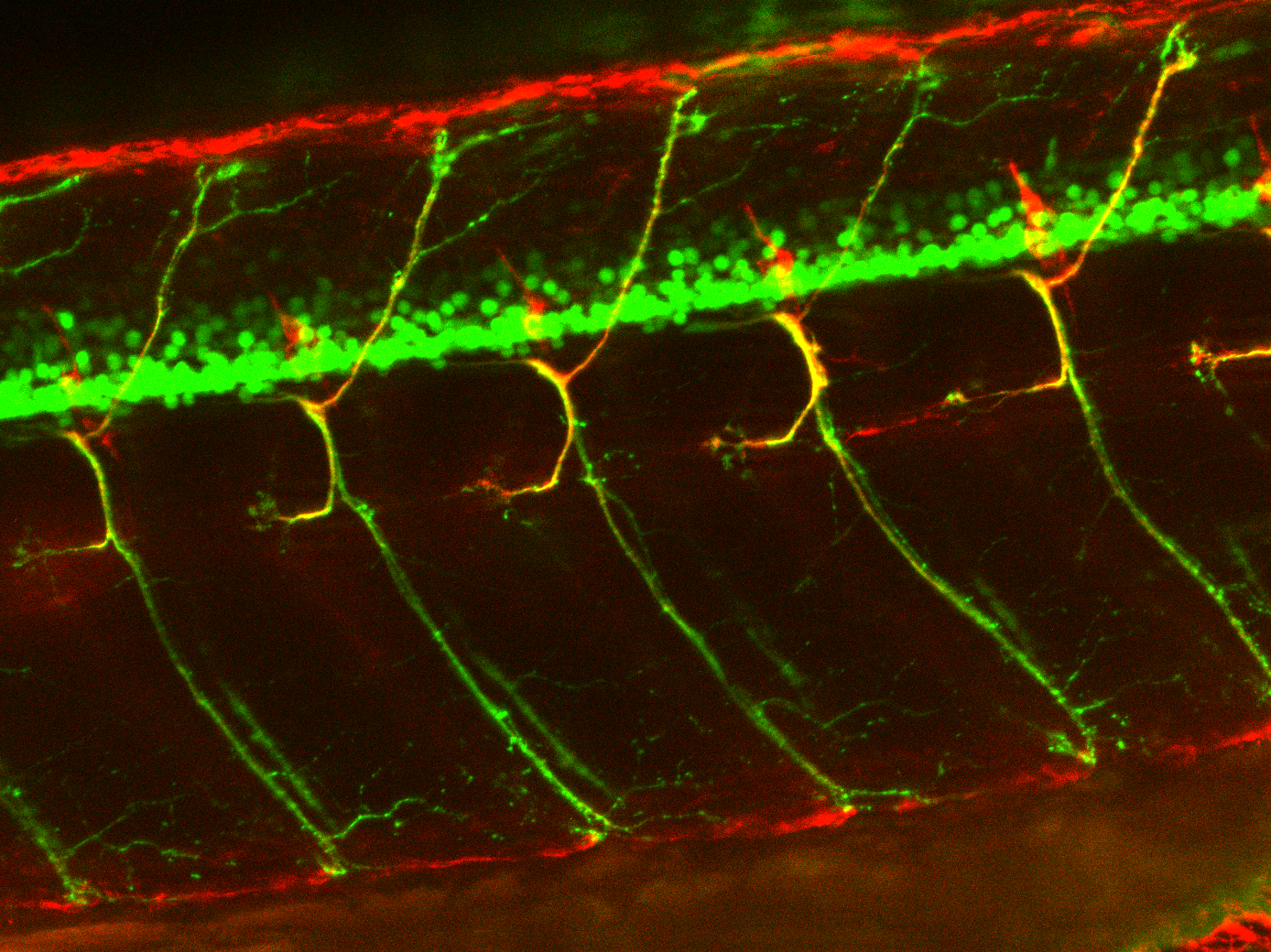

Supplement: Supplementary file 6 — Source data Fig. 1 [file 44318_2024_307_MOESM6_ESM.zip › EMBOJ-2024-116734_sourcedata_Fig 1/Fig1_panelA_MOKat3.4andKATNA1mRNA_sMN_zn5GFP.tif]

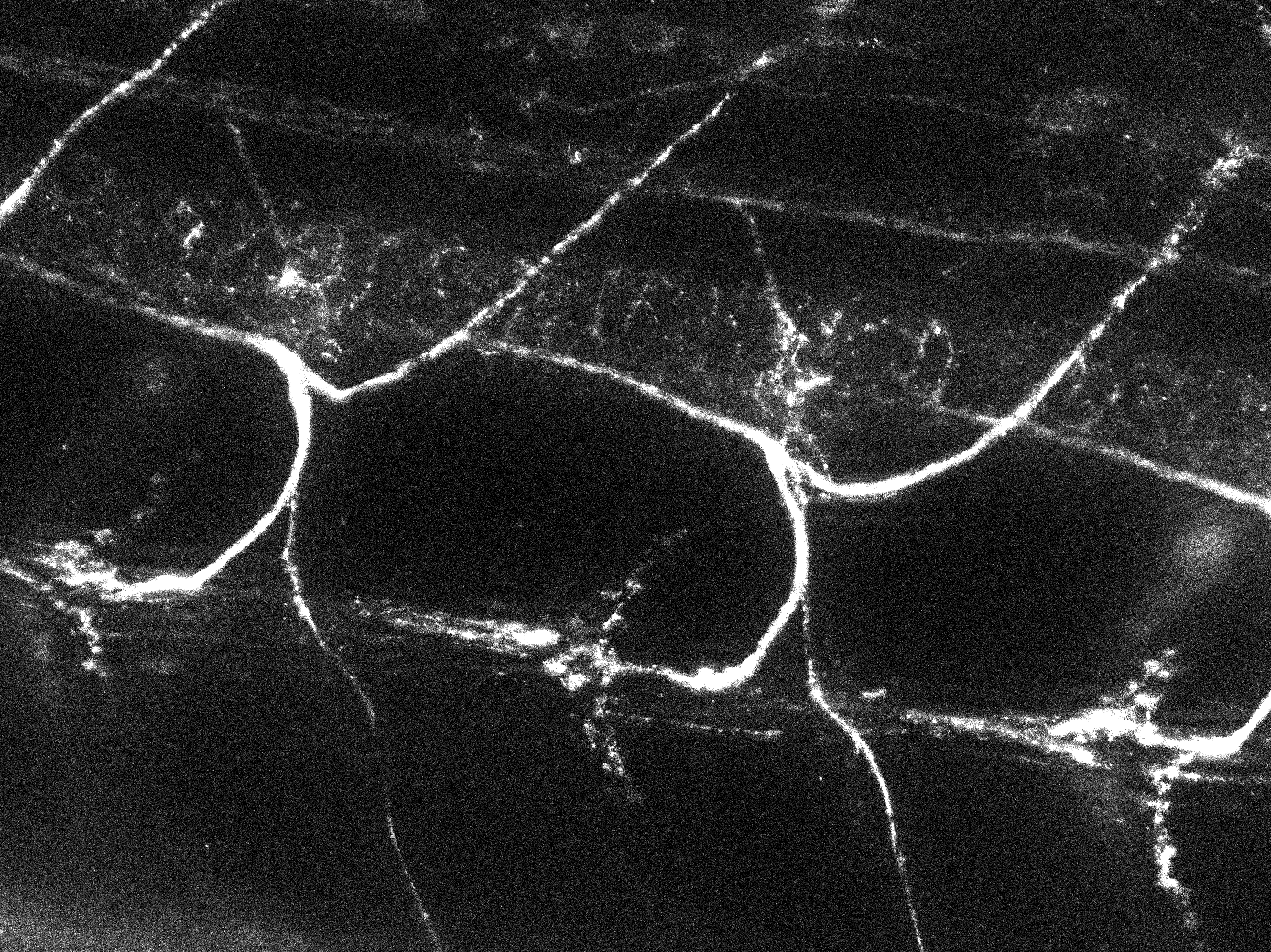

Supplement: Supplementary file 6 — Source data Fig. 1 [file 44318_2024_307_MOESM6_ESM.zip › EMBOJ-2024-116734_sourcedata_Fig 1/Fig1_panelF_katna1hetero_sMN_zn5.tif]

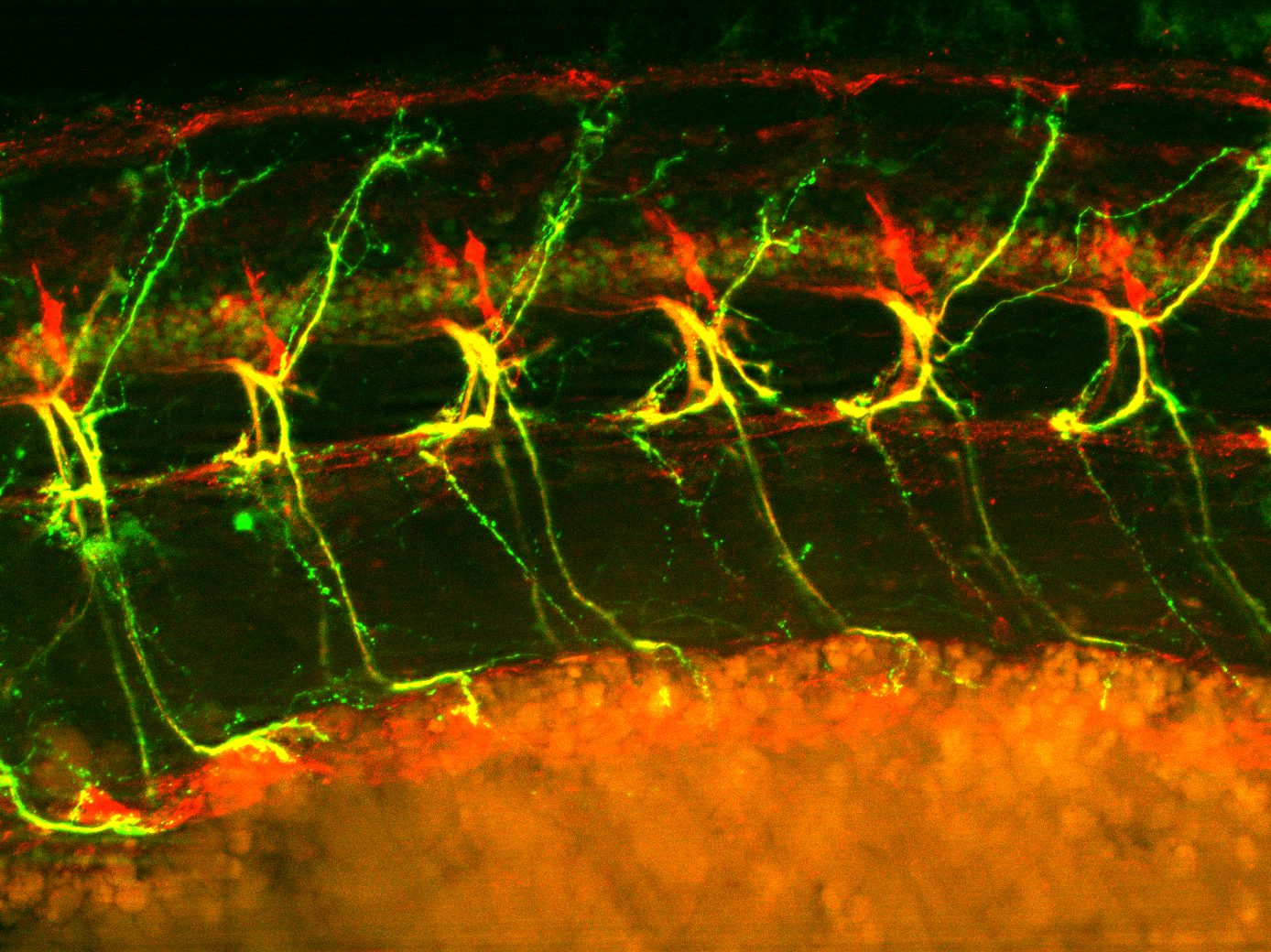

Supplement: Supplementary file 6 — Source data Fig. 1 [file 44318_2024_307_MOESM6_ESM.zip › EMBOJ-2024-116734_sourcedata_Fig 1/Fig1_panelA_MOKat1.3_sMN_zn5GFP.tif]

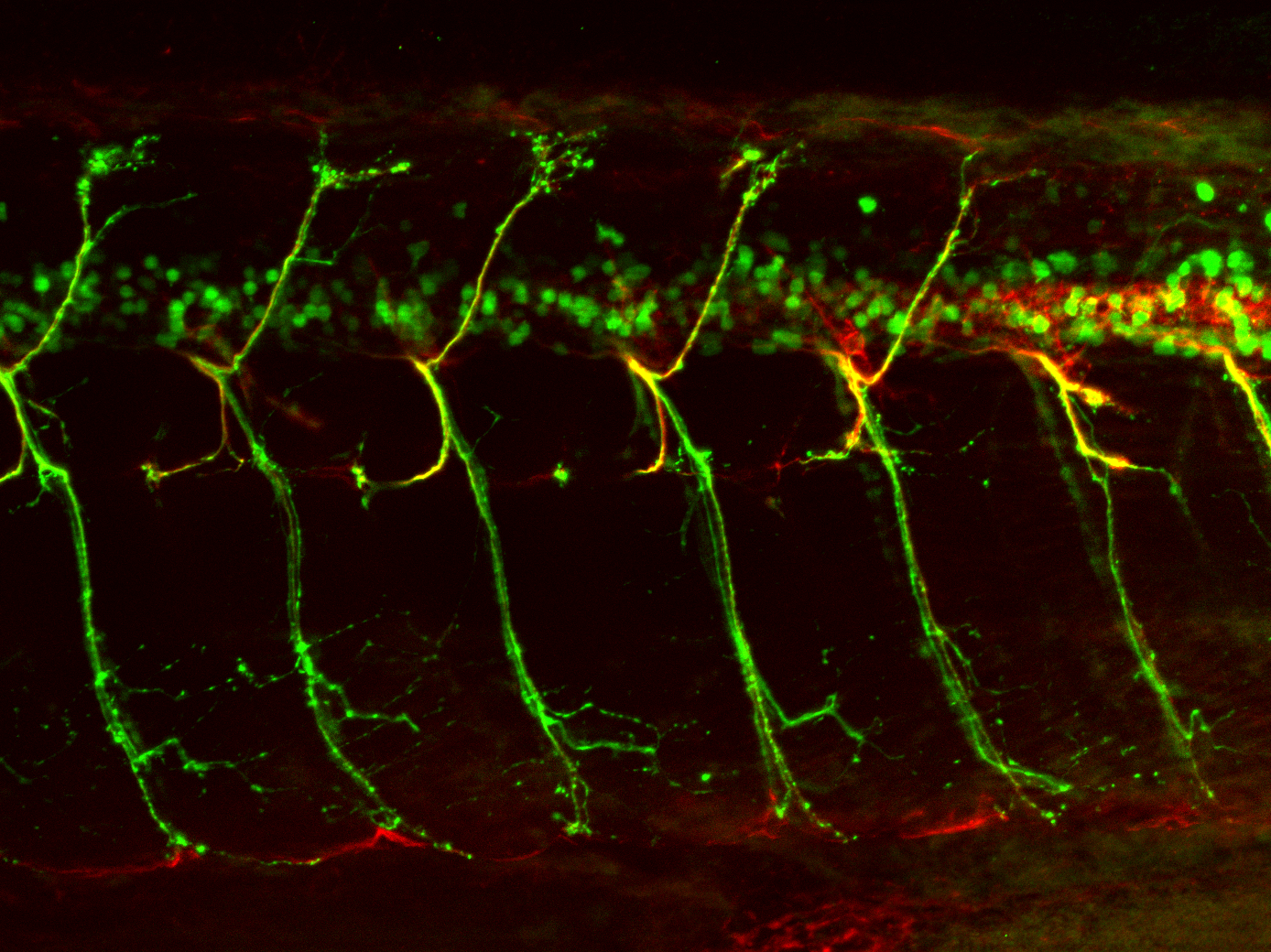

Supplement: Supplementary file 6 — Source data Fig. 1 [file 44318_2024_307_MOESM6_ESM.zip › EMBOJ-2024-116734_sourcedata_Fig 1/Fig1_panelA_MOKat1.3_sMN_zn5GFP_caudal projection.tif]

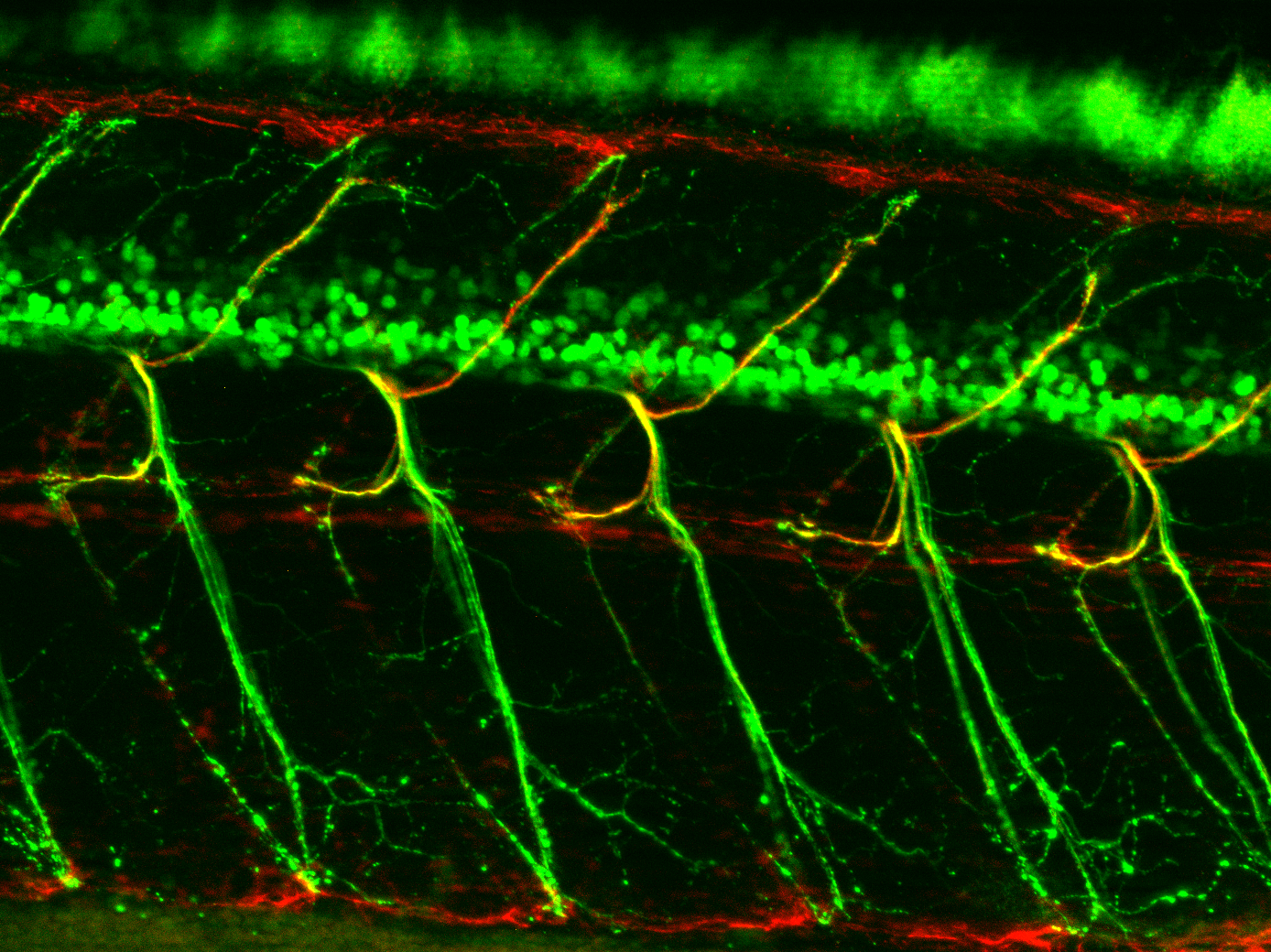

Supplement: Supplementary file 6 — Source data Fig. 1 [file 44318_2024_307_MOESM6_ESM.zip › EMBOJ-2024-116734_sourcedata_Fig 1/Fig1_panelA_MOCtl_sMN_zn5GFP.tif]

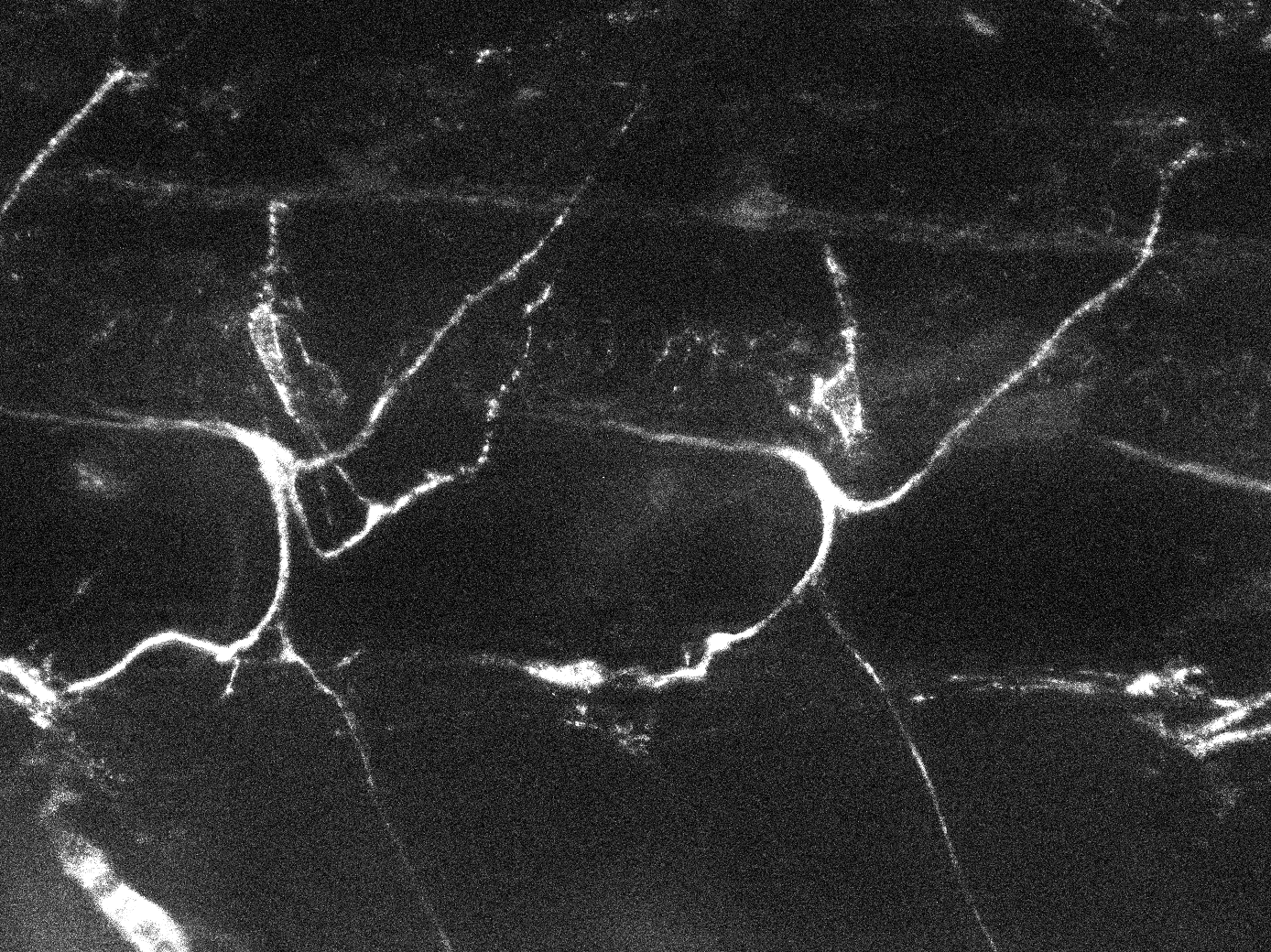

Supplement: Supplementary file 6 — Source data Fig. 1 [file 44318_2024_307_MOESM6_ESM.zip › EMBOJ-2024-116734_sourcedata_Fig 1/Fig1_panelF_katna1KOz_sMN_zn5.tif]

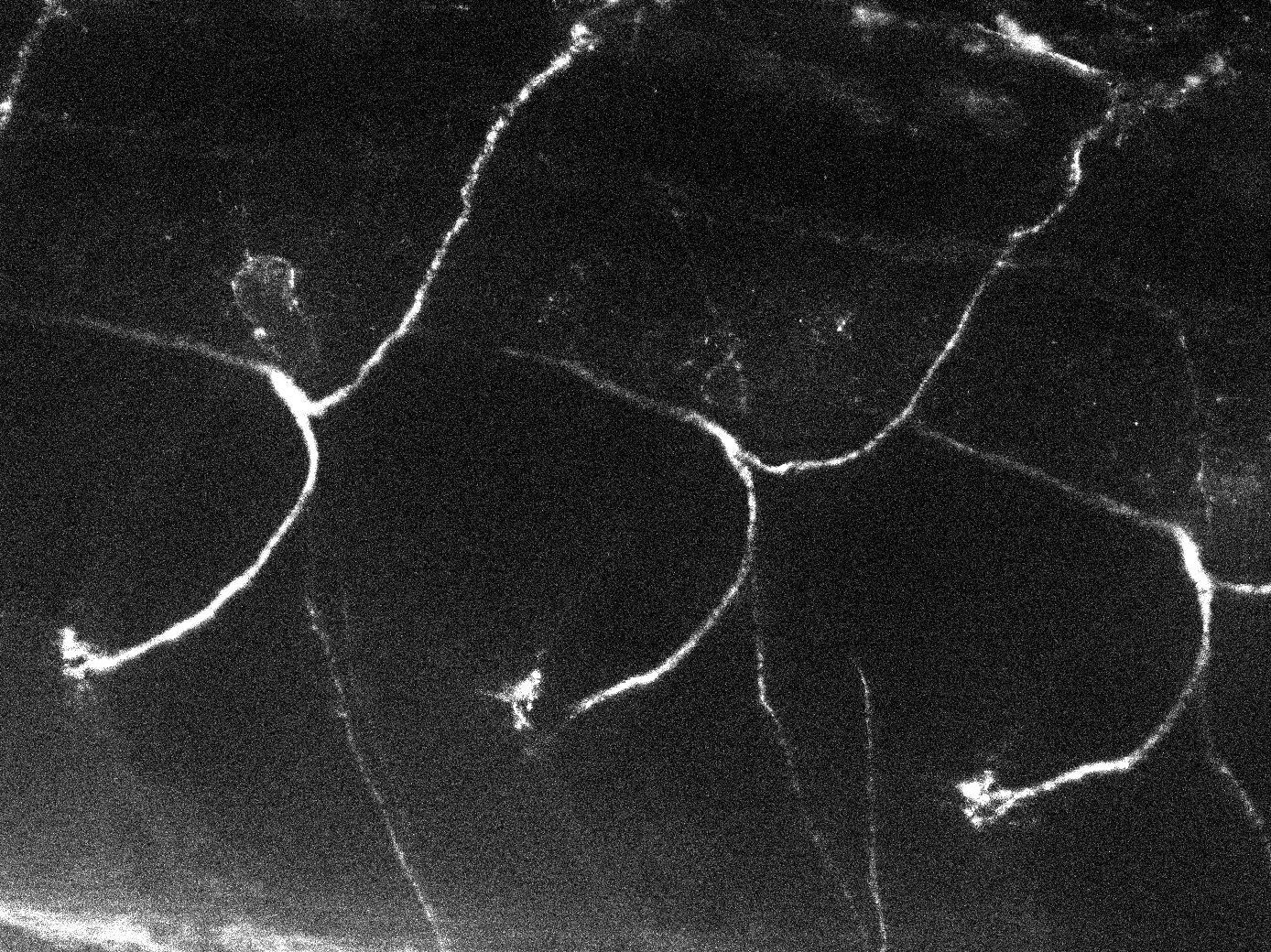

Supplement: Supplementary file 6 — Source data Fig. 1 [file 44318_2024_307_MOESM6_ESM.zip › EMBOJ-2024-116734_sourcedata_Fig 1/Fig1_panelF_katna1WT_sMN_zn5.tif]

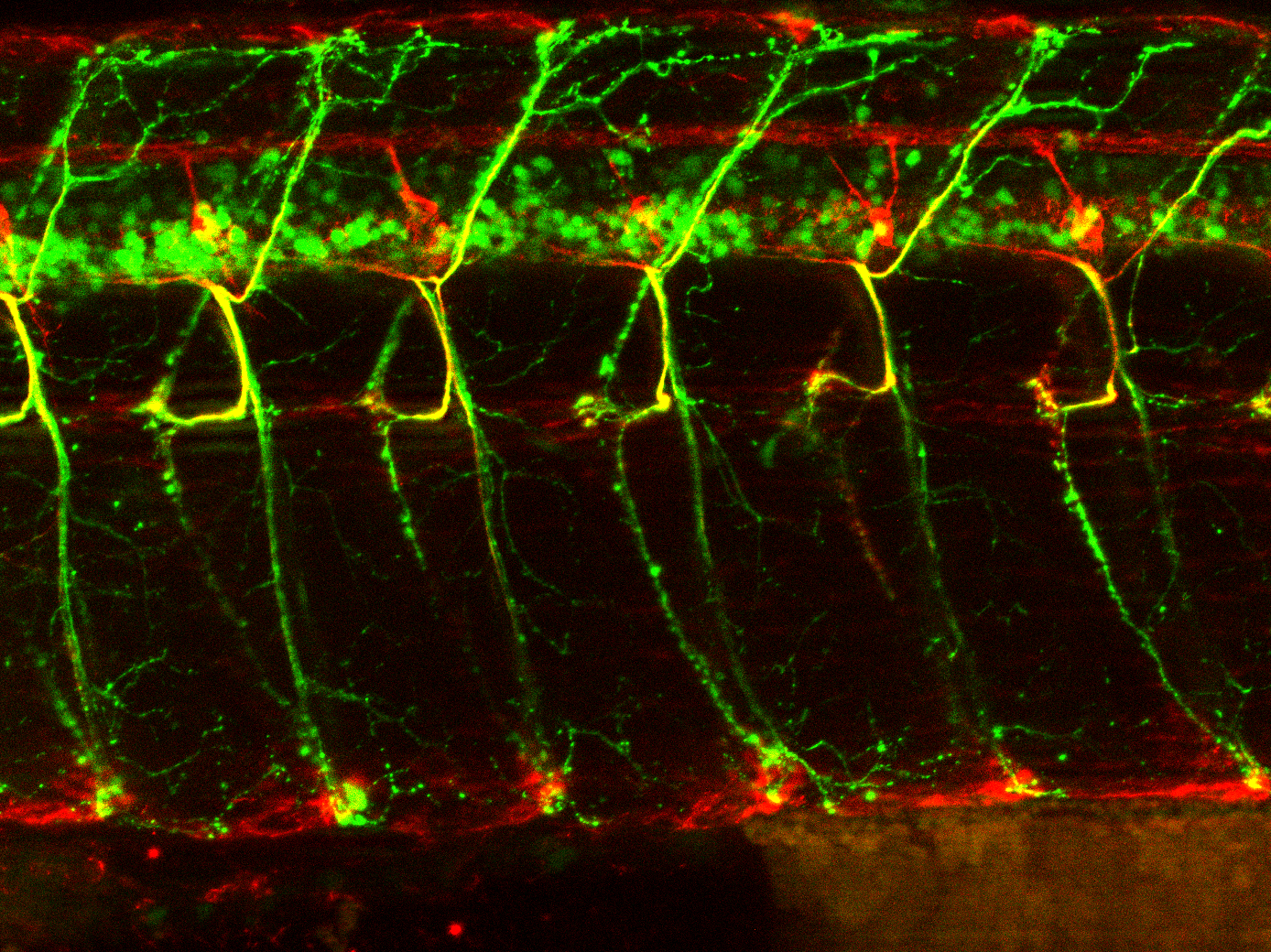

Supplement: Supplementary file 6 — Source data Fig. 1 [file 44318_2024_307_MOESM6_ESM.zip › EMBOJ-2024-116734_sourcedata_Fig 1/Fig1_panelA_MOKat1.3andKATN1mRNA_sMN_zn5GFP.tif]

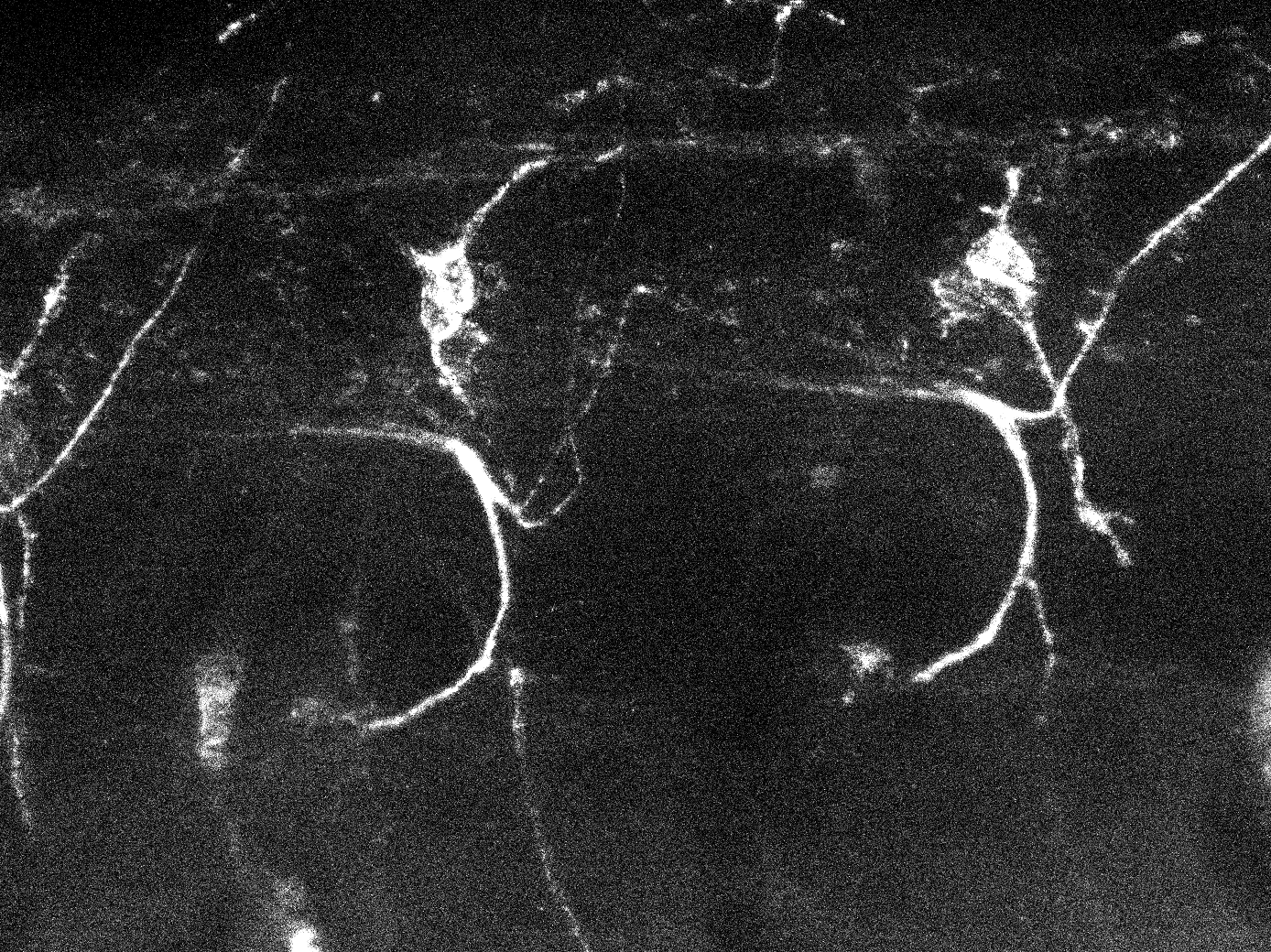

Supplement: Supplementary file 6 — Source data Fig. 1 [file 44318_2024_307_MOESM6_ESM.zip › EMBOJ-2024-116734_sourcedata_Fig 1/Fig1_panelF_katna1KOmz_sMN_zn5.tif]

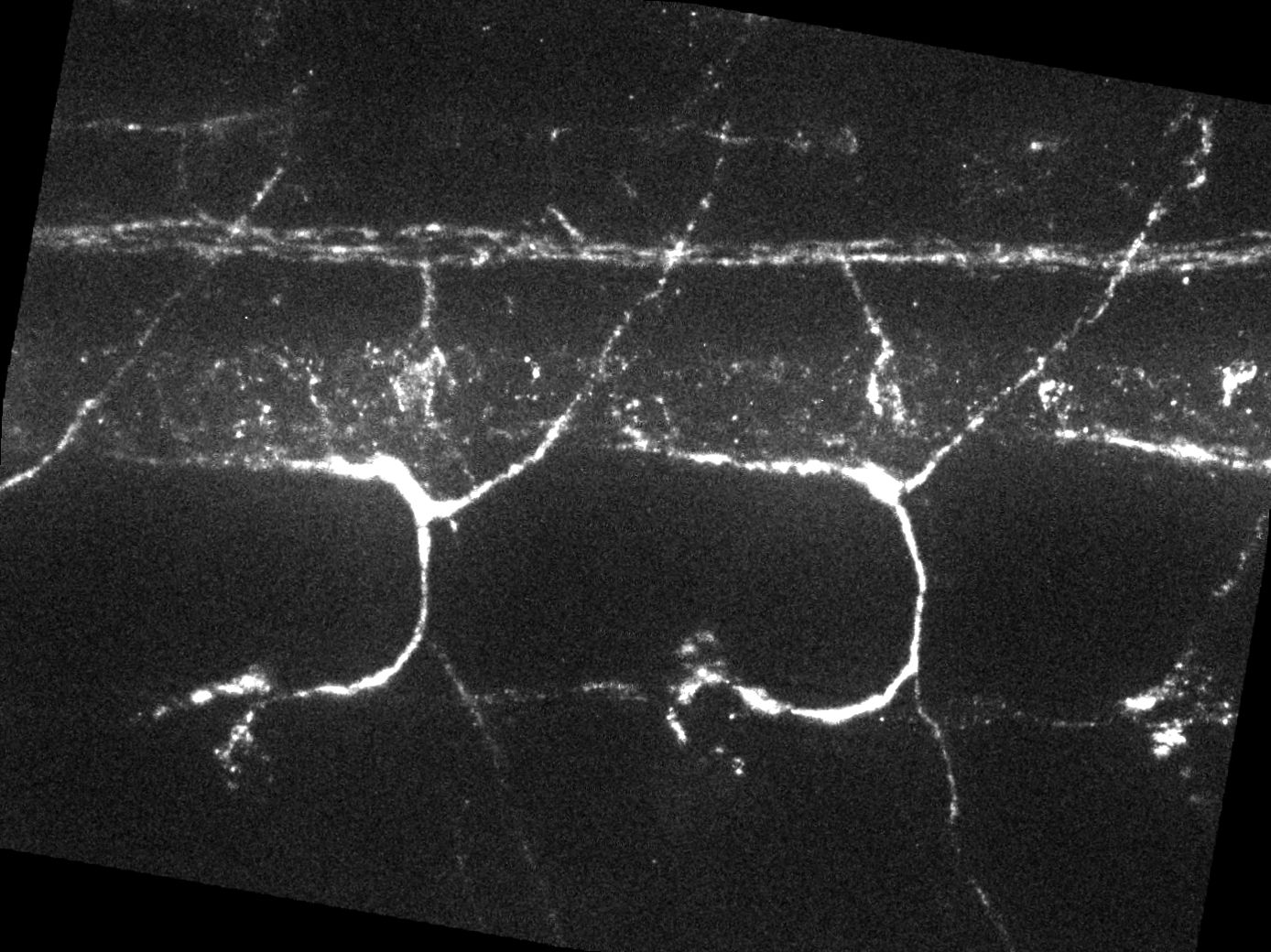

Supplement: Supplementary file 6 — Source data Fig. 1 [file 44318_2024_307_MOESM6_ESM.zip › EMBOJ-2024-116734_sourcedata_Fig 1/Fig1_panelF_katna1KOmzandKATNA1mRNA_sMN_zn5.tif]

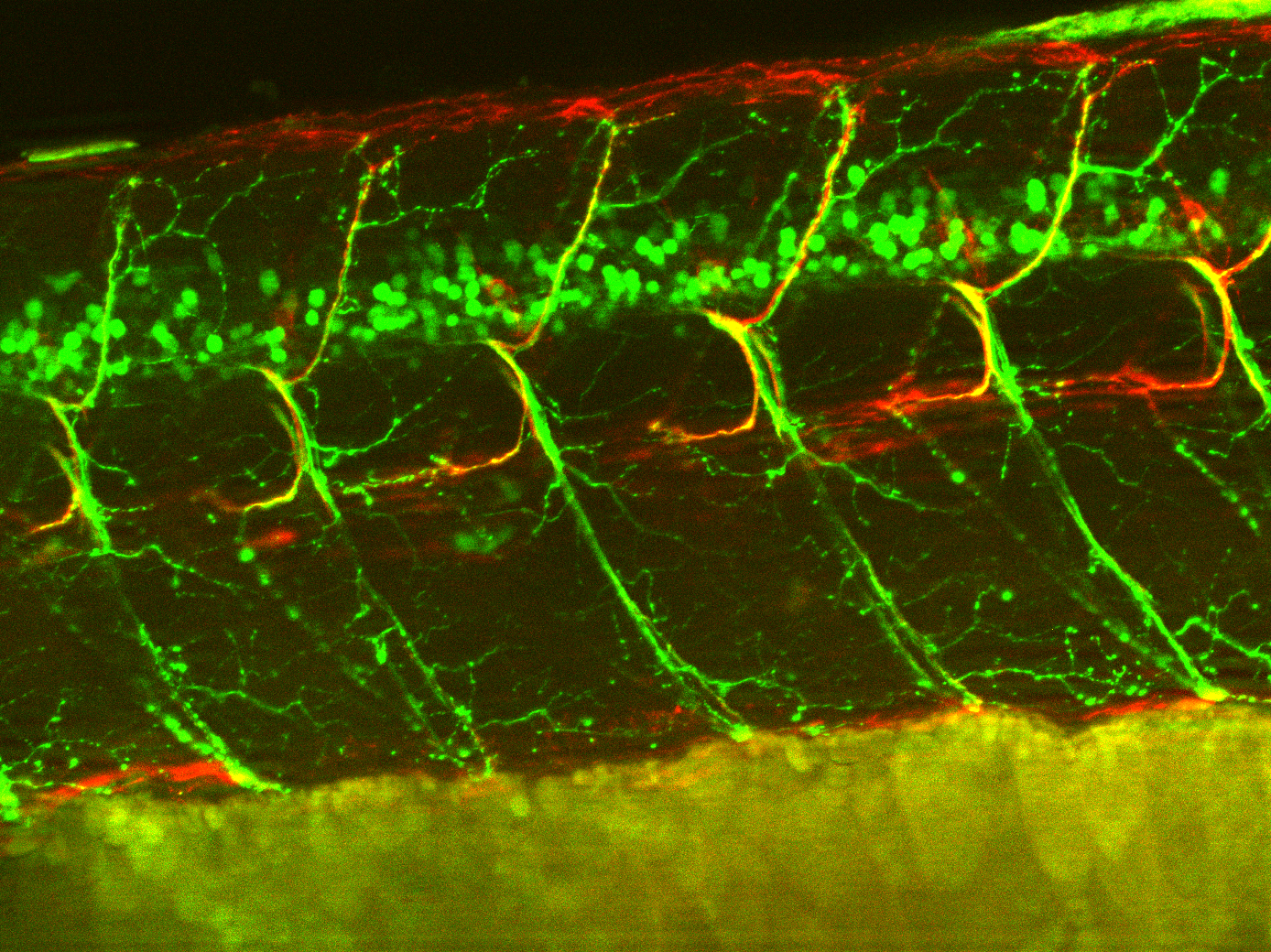

Supplement: Supplementary file 8 — Source data Fig. 3 [file 44318_2024_307_MOESM8_ESM.zip › EMBOJ-2024-116734_sourcedata_Fig 3/Fig3_panelA_MOctl_sMN_zn5GFP.tif]

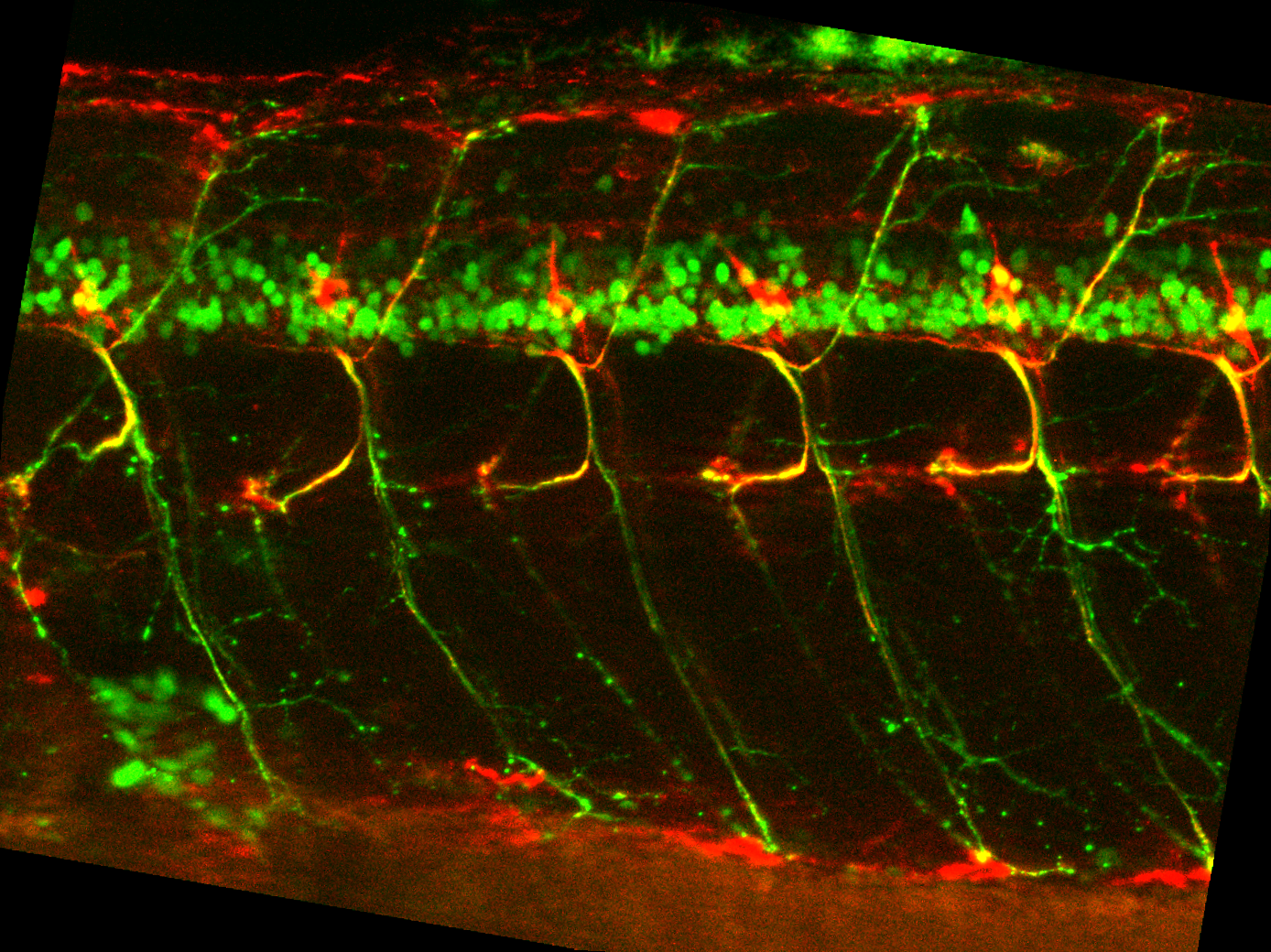

Supplement: Supplementary file 8 — Source data Fig. 3 [file 44318_2024_307_MOESM8_ESM.zip › EMBOJ-2024-116734_sourcedata_Fig 3/Fig3_panelD_KATNA1mRNA_sMN_zn5GFP.tif]

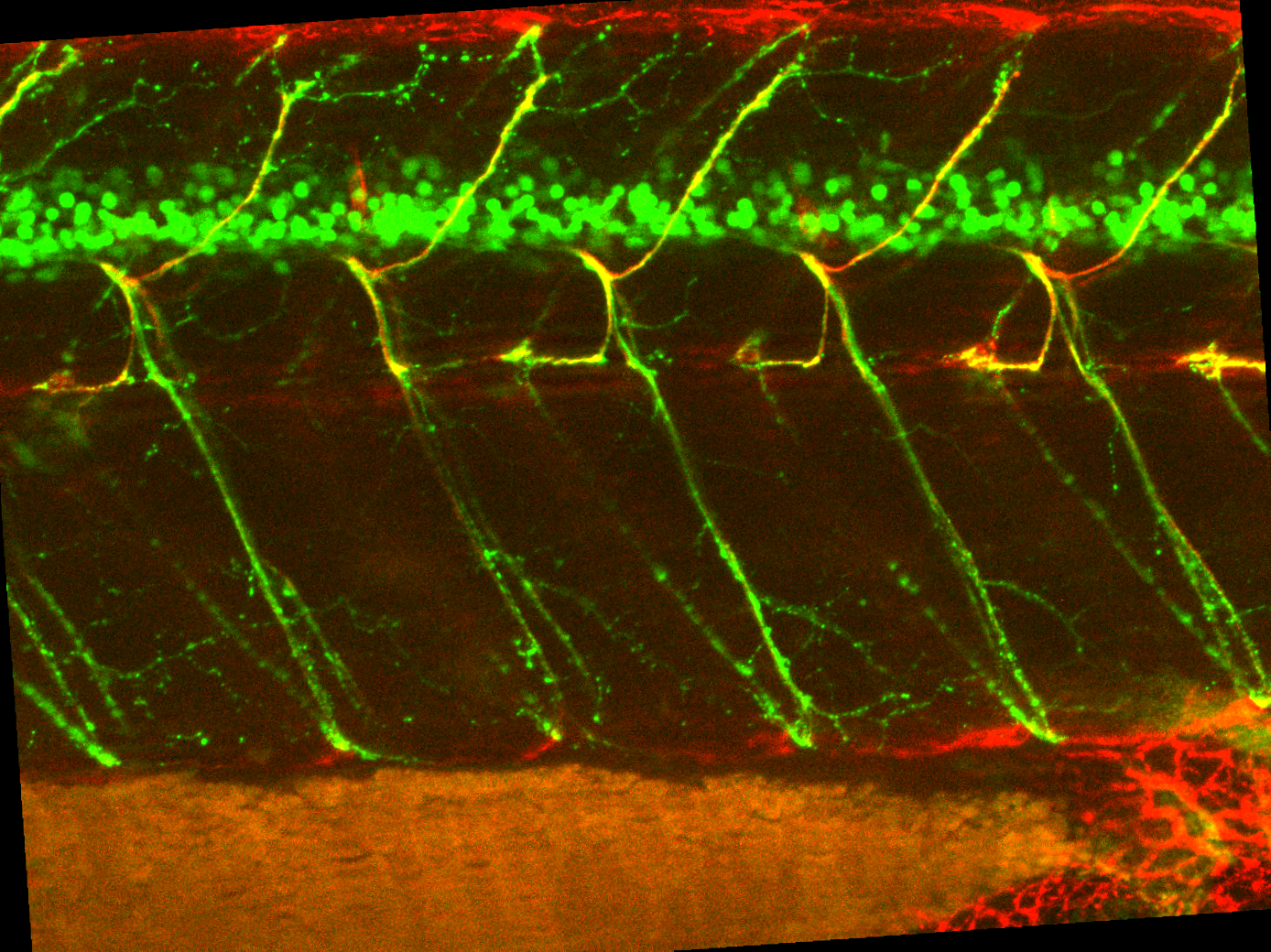

Supplement: Supplementary file 8 — Source data Fig. 3 [file 44318_2024_307_MOESM8_ESM.zip › EMBOJ-2024-116734_sourcedata_Fig 3/Fig3_panelD_MOctl_sMN_zn5GFP.tif]

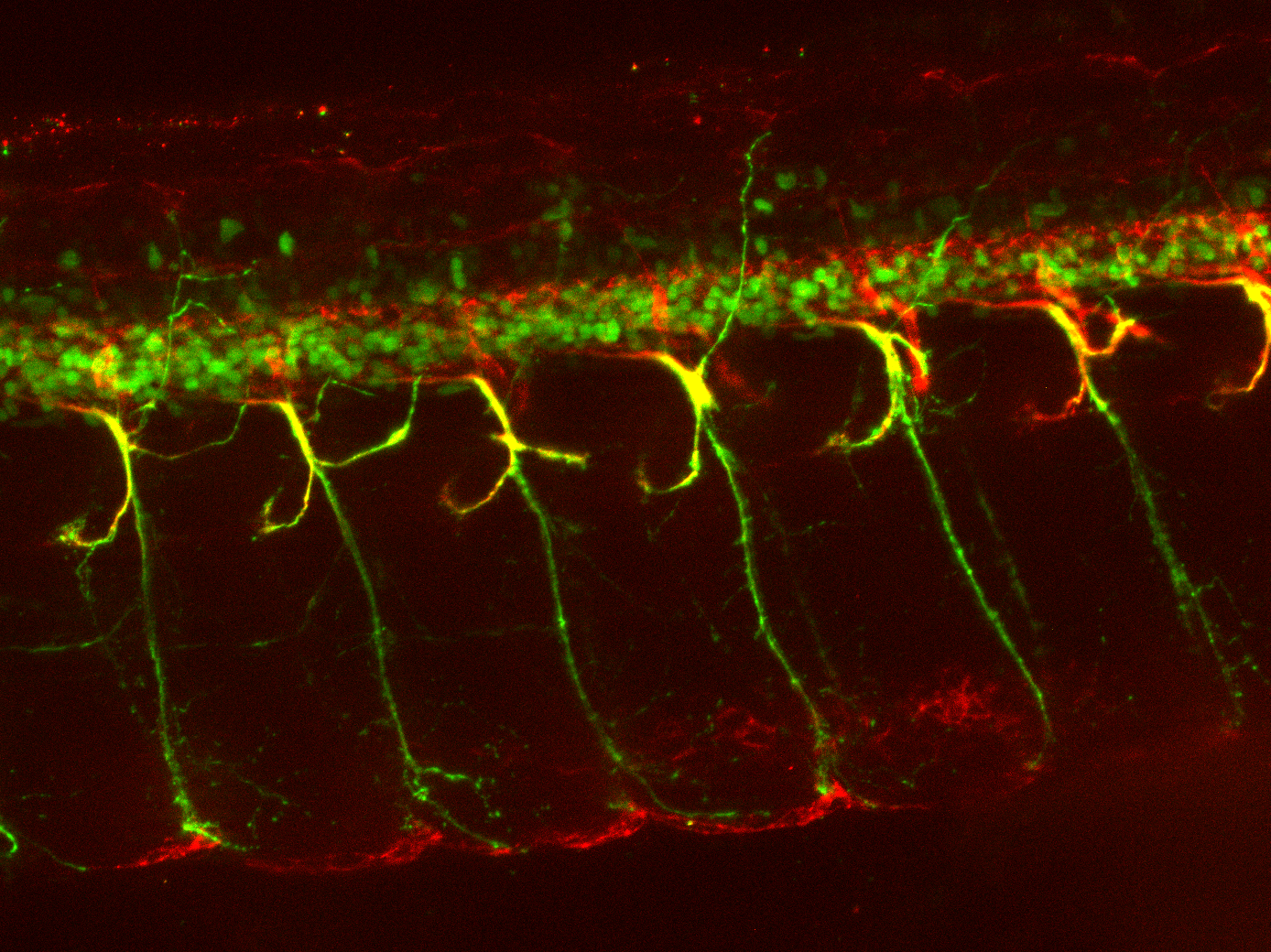

Supplement: Supplementary file 8 — Source data Fig. 3 [file 44318_2024_307_MOESM8_ESM.zip › EMBOJ-2024-116734_sourcedata_Fig 3/Fig3_panelA_MOKat1.3andSPASTmRNA_sMNdorsal_zn5GFP.tif]

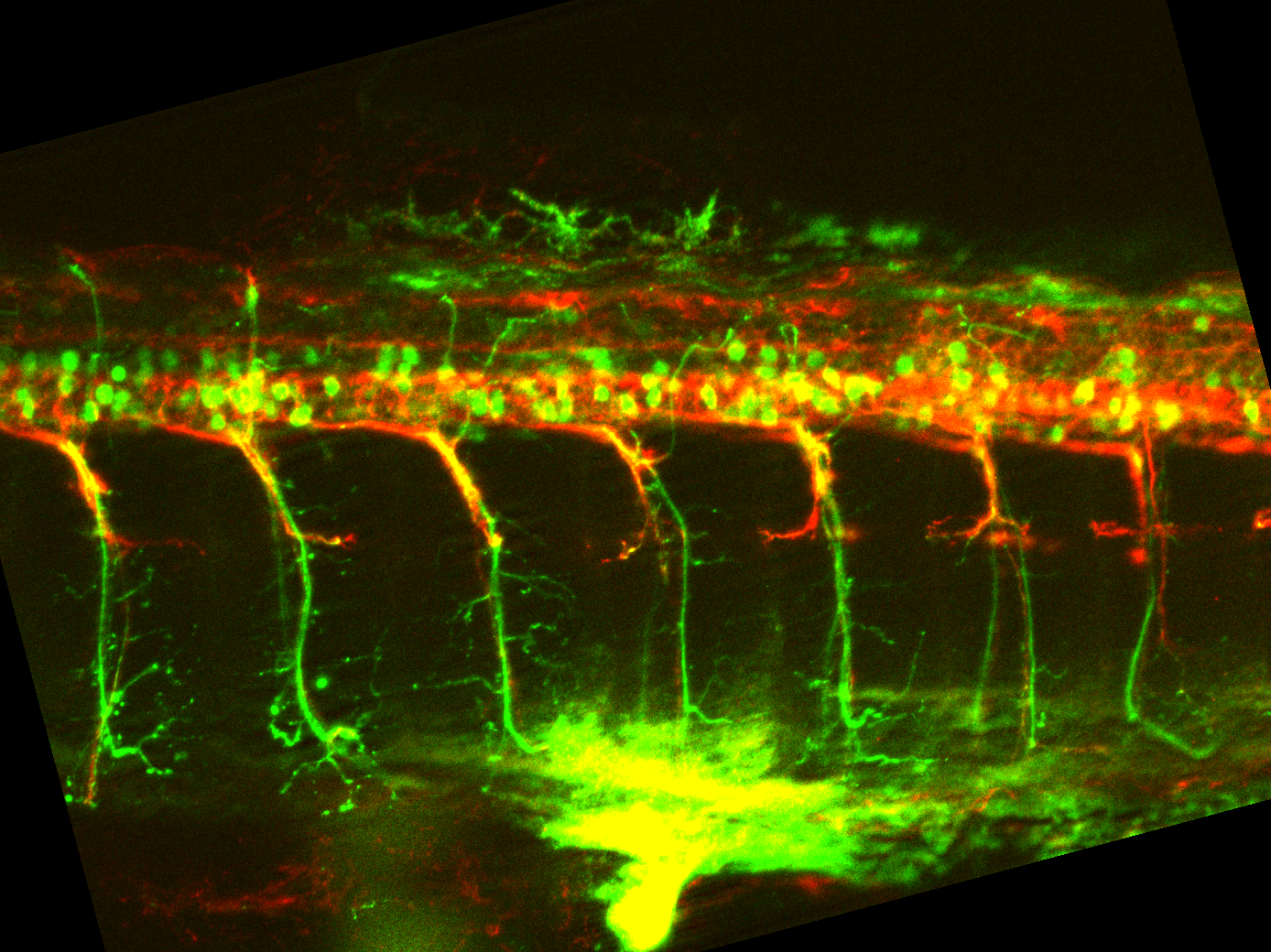

Supplement: Supplementary file 8 — Source data Fig. 3 [file 44318_2024_307_MOESM8_ESM.zip › EMBOJ-2024-116734_sourcedata_Fig 3/Fig3_panelD_MOSPAST_sMN_zn5GFP.tif]

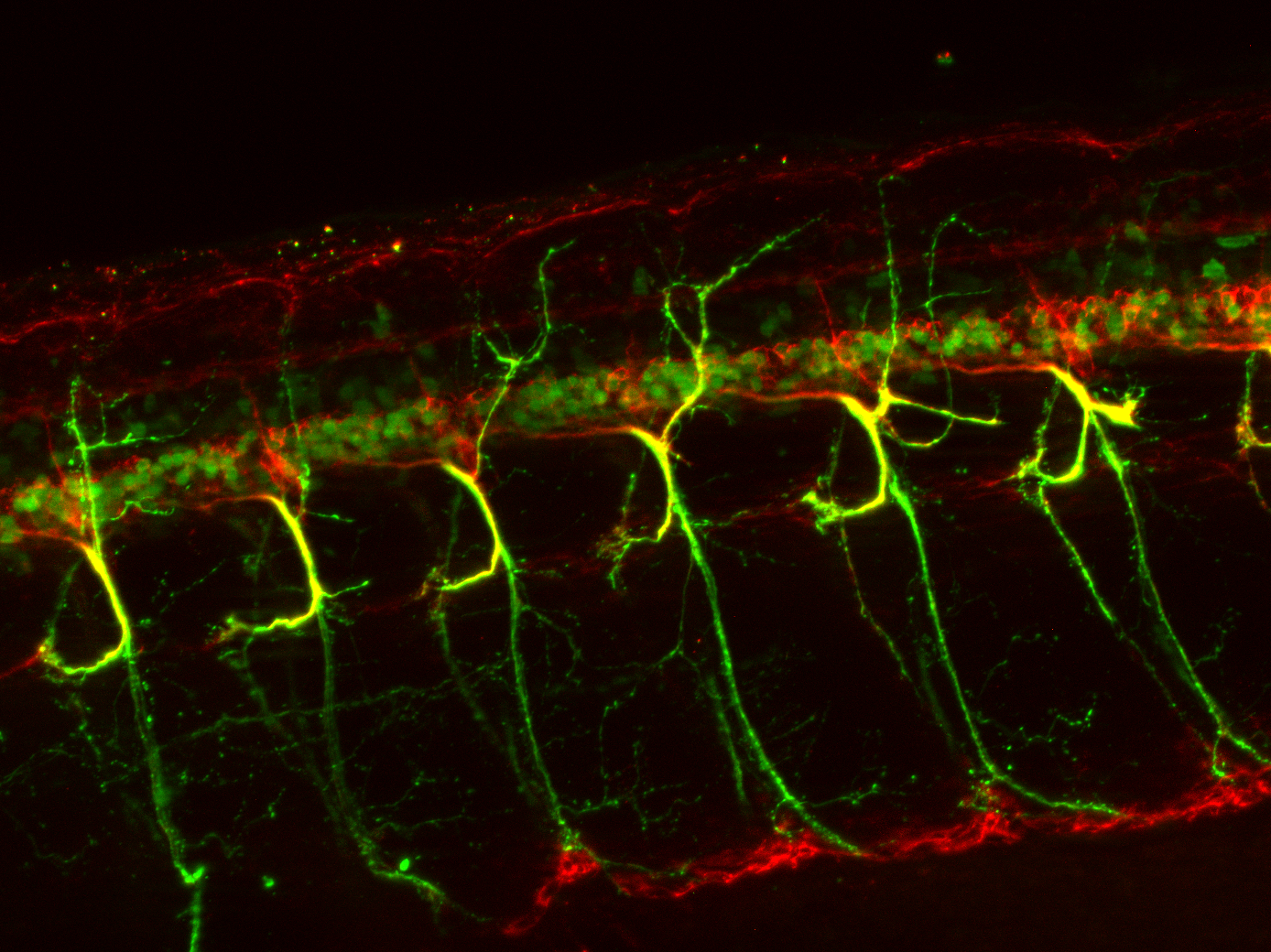

Supplement: Supplementary file 8 — Source data Fig. 3 [file 44318_2024_307_MOESM8_ESM.zip › EMBOJ-2024-116734_sourcedata_Fig 3/Fig3_panelA_MOKat1.3_sMNdorsal_zn5GFP.tif]

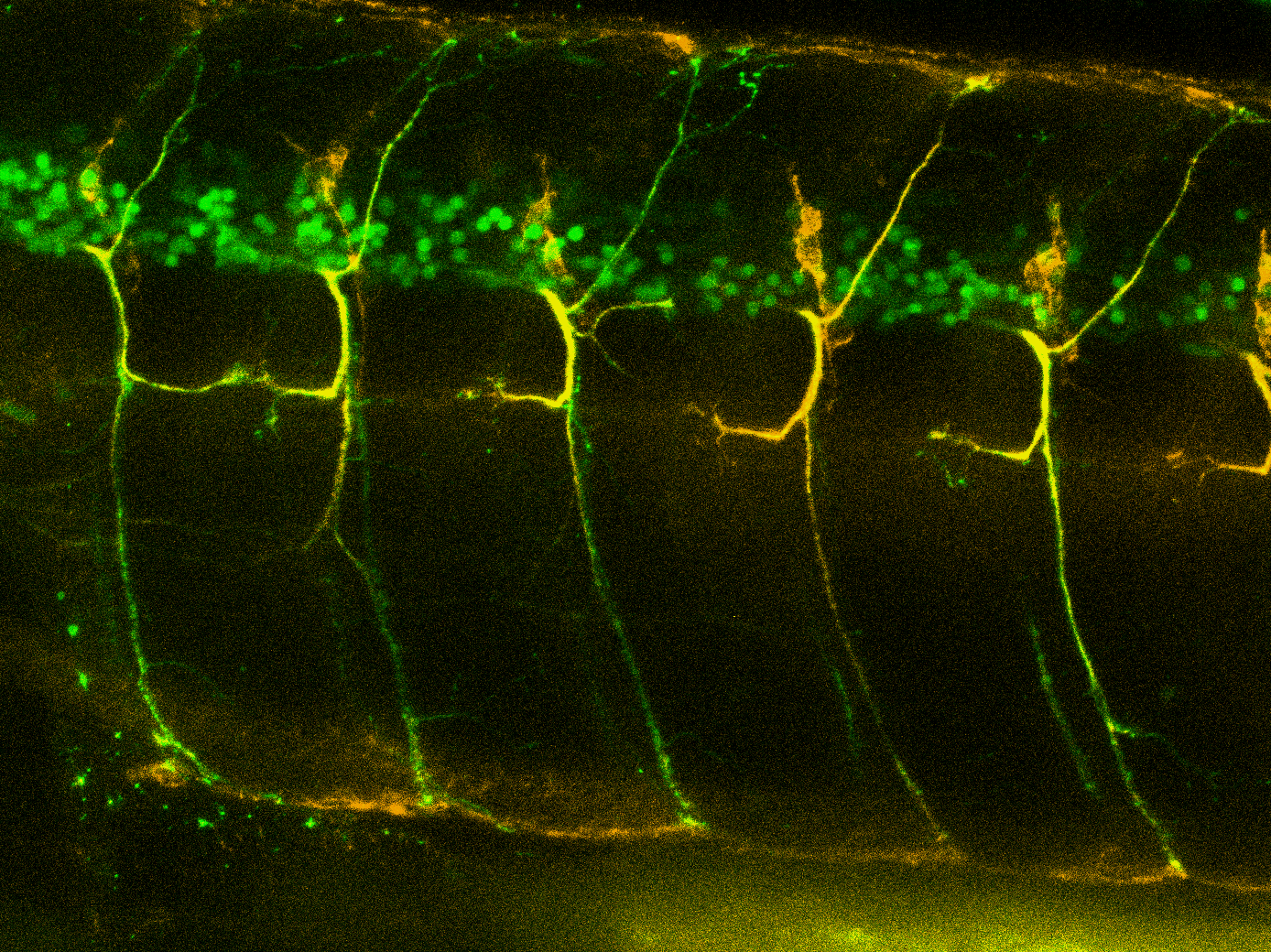

Supplement: Supplementary file 8 — Source data Fig. 3 [file 44318_2024_307_MOESM8_ESM.zip › EMBOJ-2024-116734_sourcedata_Fig 3/Fig3_panelA_MOKat1.3 and SPASTmRNA_sMNrostral_zn5GFP.tif]

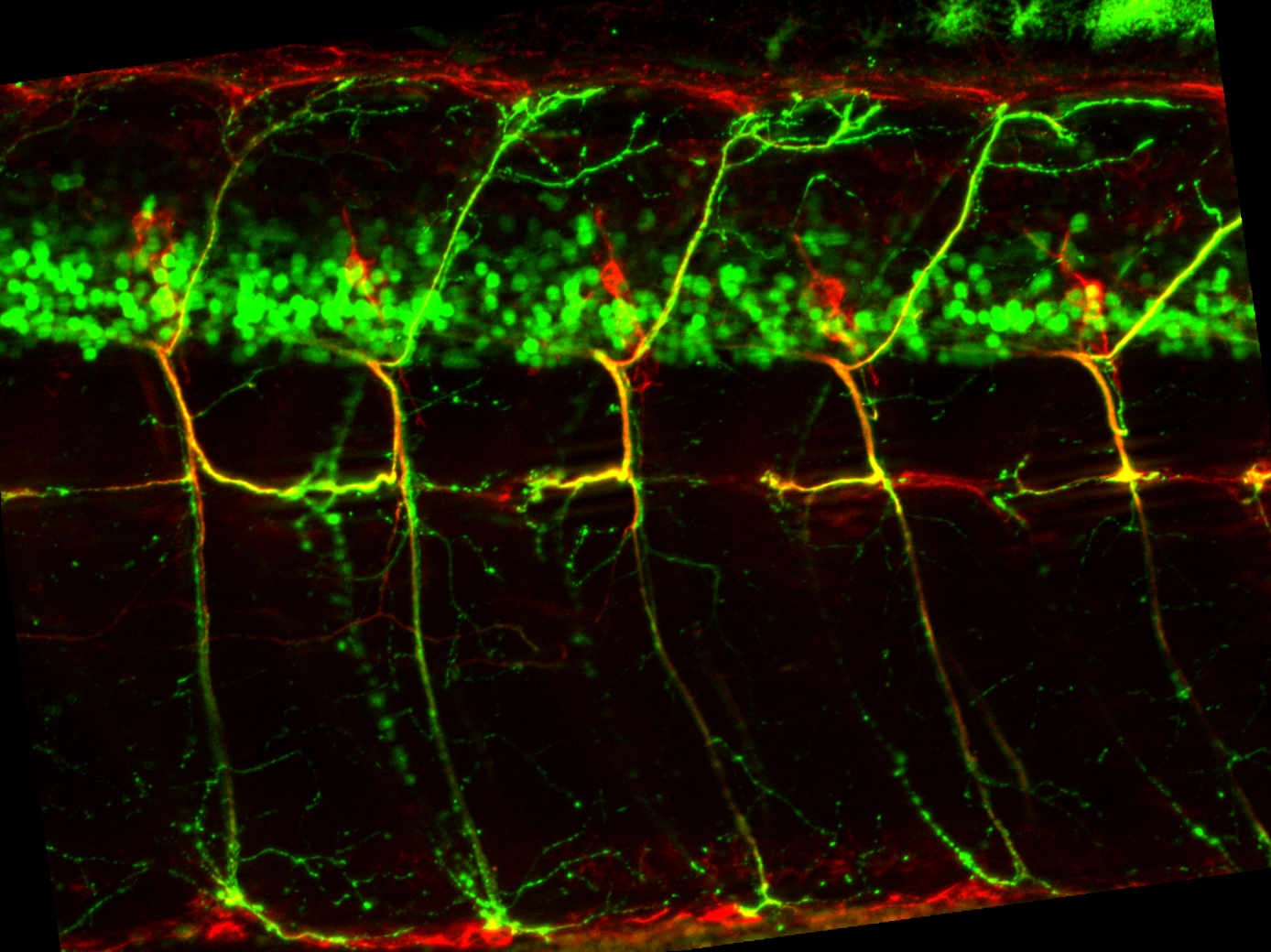

Supplement: Supplementary file 8 — Source data Fig. 3 [file 44318_2024_307_MOESM8_ESM.zip › EMBOJ-2024-116734_sourcedata_Fig 3/Fig3_panelA_MOKat1.3_sMNrostral_zn5GFP_LV.tif]

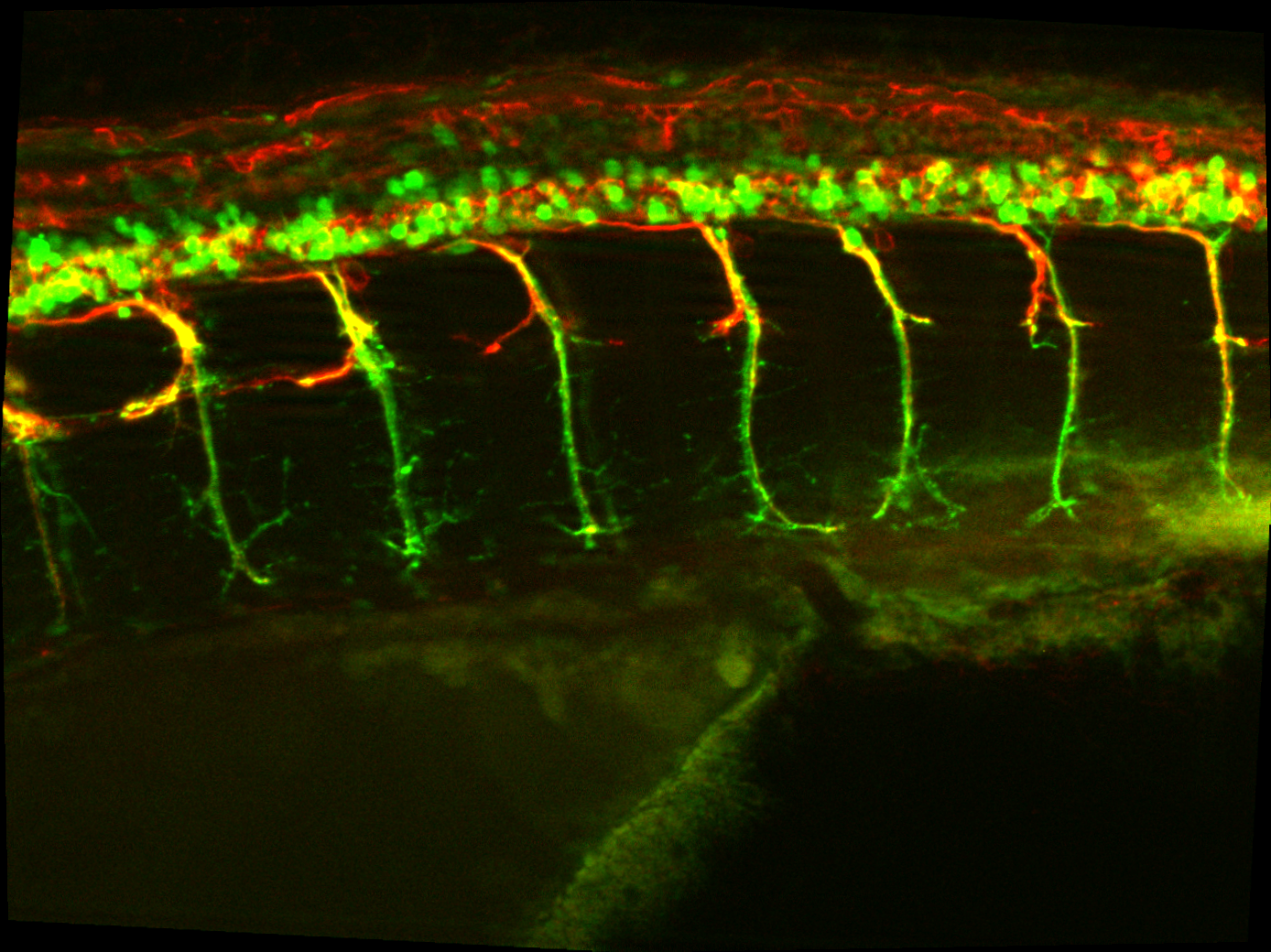

Supplement: Supplementary file 8 — Source data Fig. 3 [file 44318_2024_307_MOESM8_ESM.zip › EMBOJ-2024-116734_sourcedata_Fig 3/Fig3_panelD_MOSPASTandKATNA1mRNA_sMN_zn5GFP.tif]

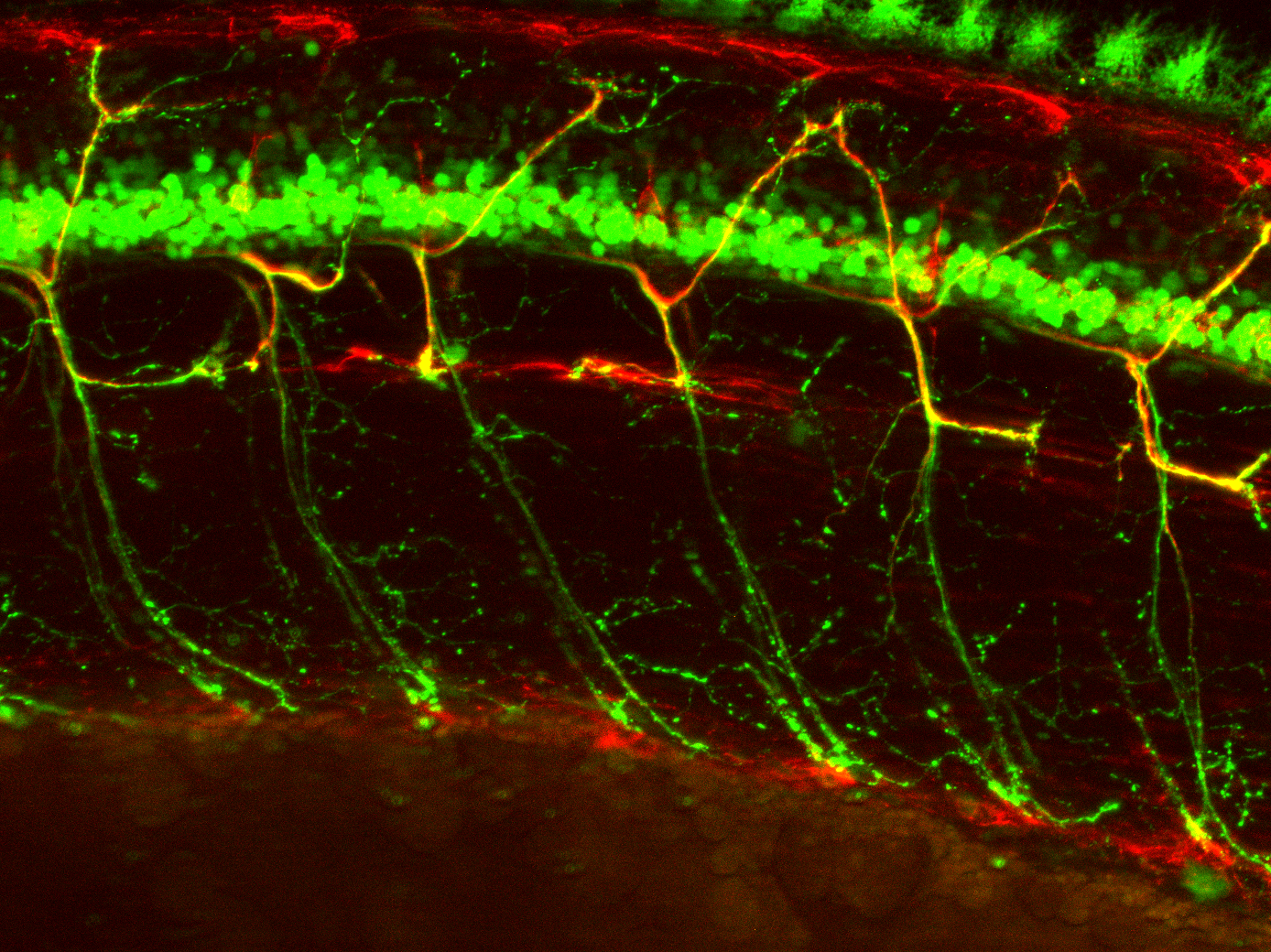

Supplement: Supplementary file 9 — Source data Fig. 4 [file 44318_2024_307_MOESM9_ESM.zip › EMBOJ-2024-116734_sourcedata_Fig 4/Fig.4D_MOTTLL6_smnzn5GFP.tif]

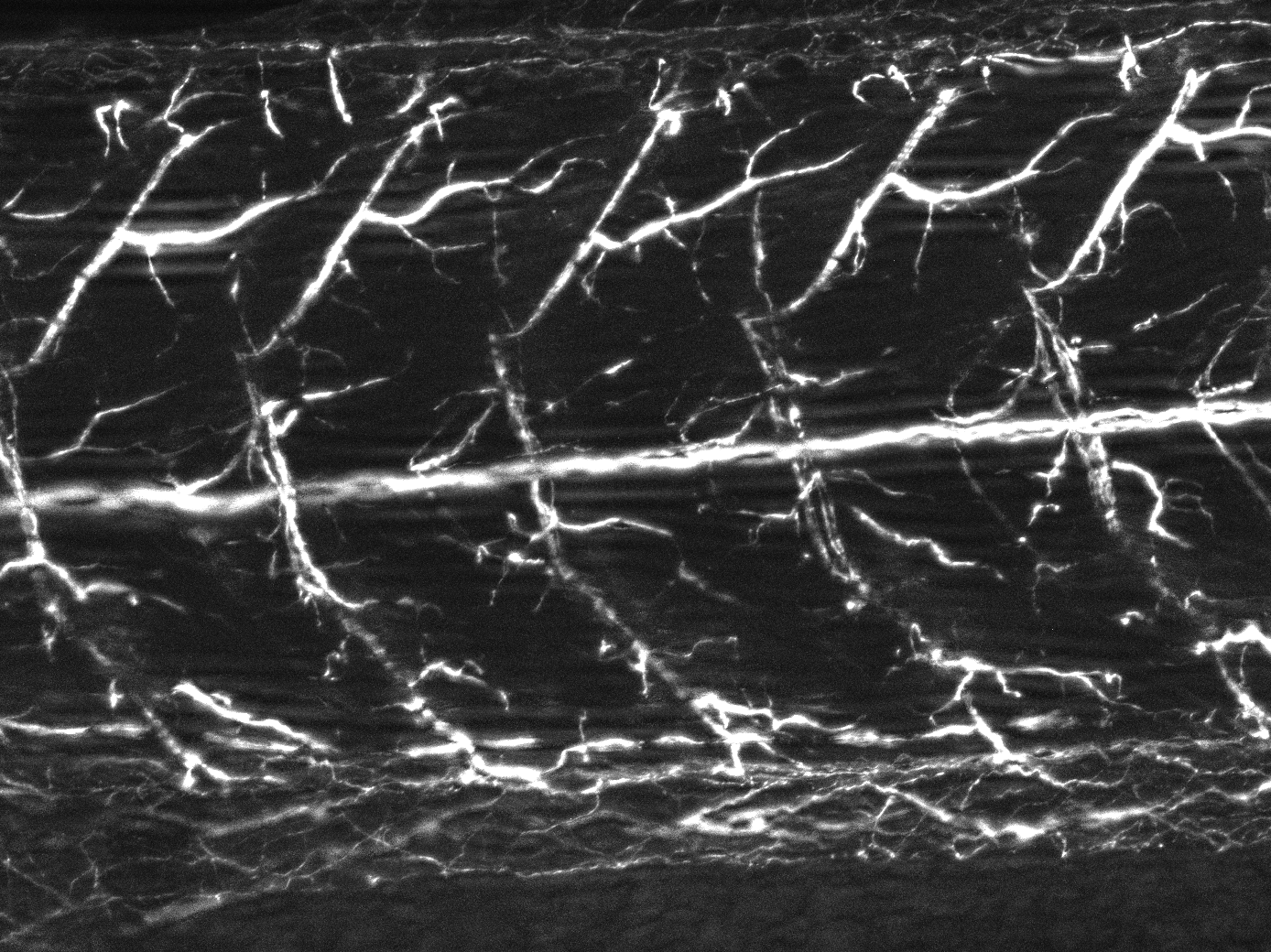

Supplement: Supplementary file 9 — Source data Fig. 4 [file 44318_2024_307_MOESM9_ESM.zip › EMBOJ-2024-116734_sourcedata_Fig 4/Fig.4A_GT335_72hpf.tif]

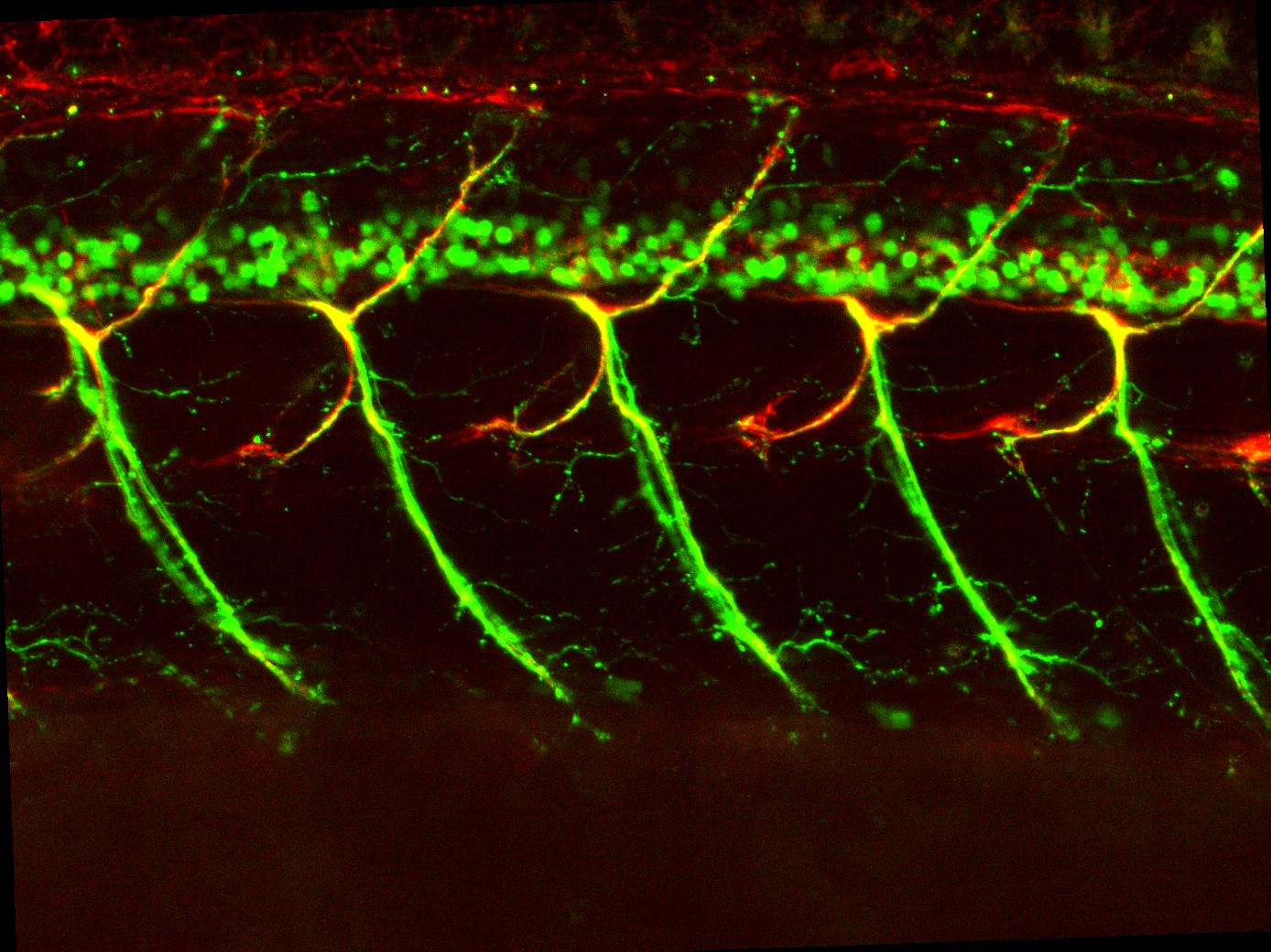

Supplement: Supplementary file 9 — Source data Fig. 4 [file 44318_2024_307_MOESM9_ESM.zip › EMBOJ-2024-116734_sourcedata_Fig 4/Fig4.D_MOCTL_smnzn5GFP.tif]

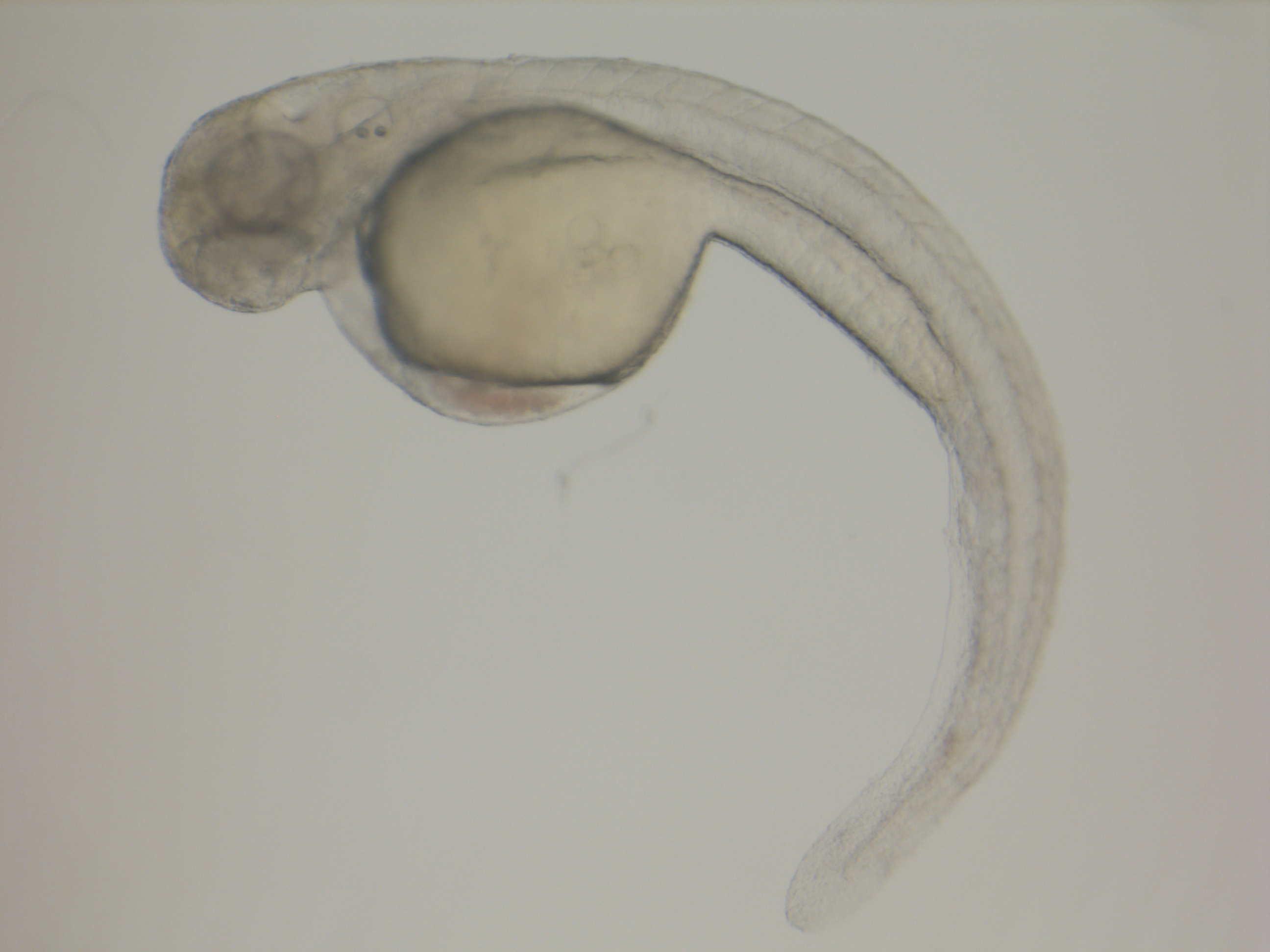

Supplement: Supplementary file 9 — Source data Fig. 4 [file 44318_2024_307_MOESM9_ESM.zip › EMBOJ-2024-116734_sourcedata_Fig 4/Fig4.DMOTTLL11.tif]

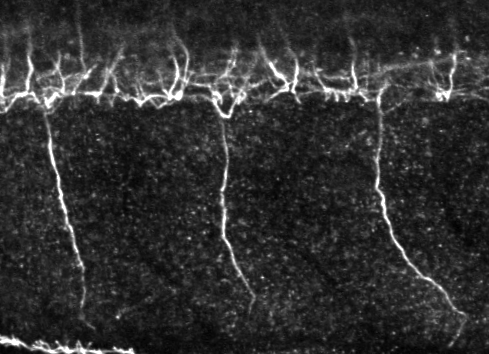

Supplement: Supplementary file 9 — Source data Fig. 4 [file 44318_2024_307_MOESM9_ESM.zip › EMBOJ-2024-116734_sourcedata_Fig 4/Fig4.B_MOCTL_polyE.tif]

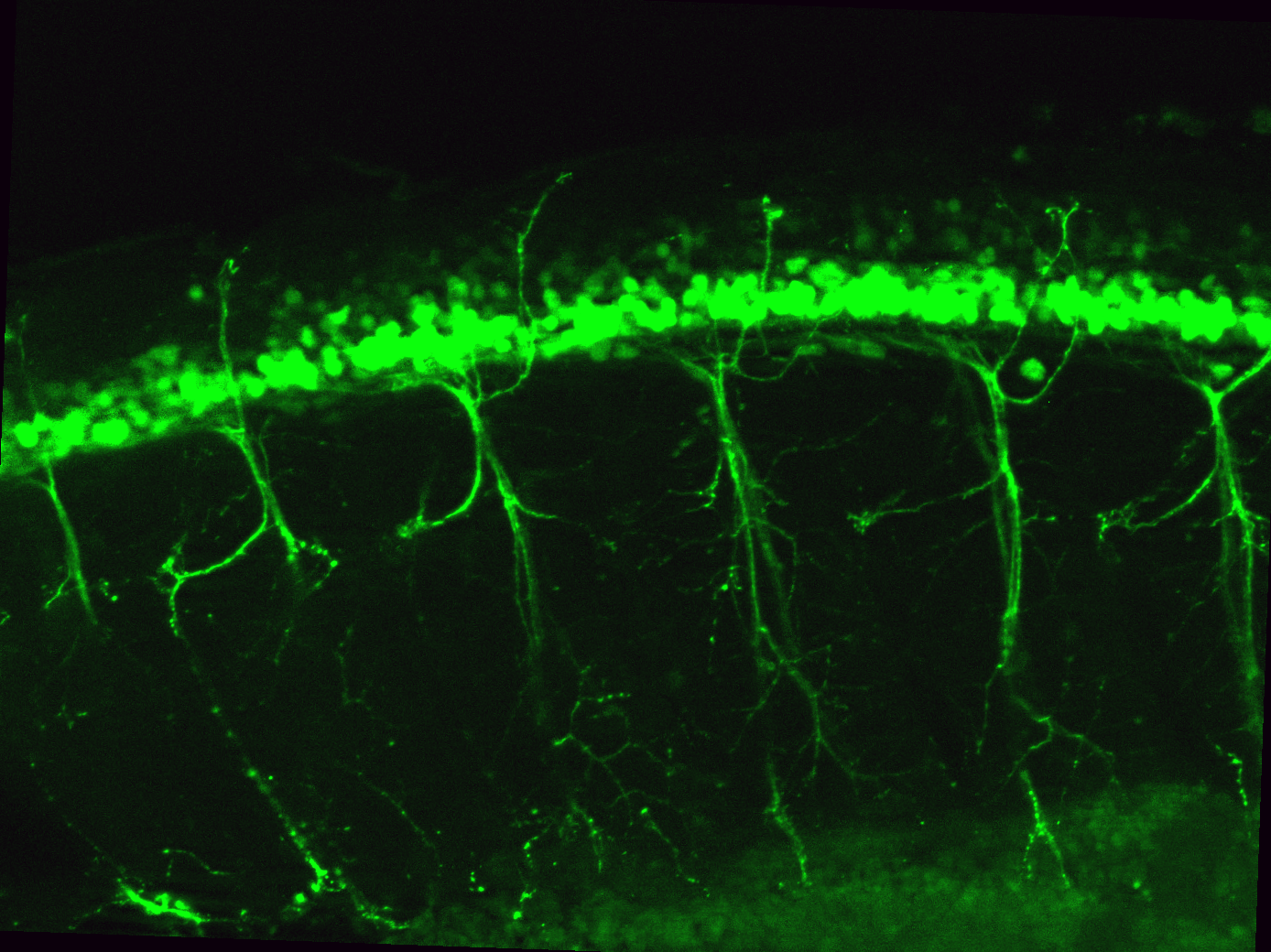

Supplement: Supplementary file 9 — Source data Fig. 4 [file 44318_2024_307_MOESM9_ESM.zip › EMBOJ-2024-116734_sourcedata_Fig 4/Fig.4D_MOTTLL6_smnsorting_GFP.tif]

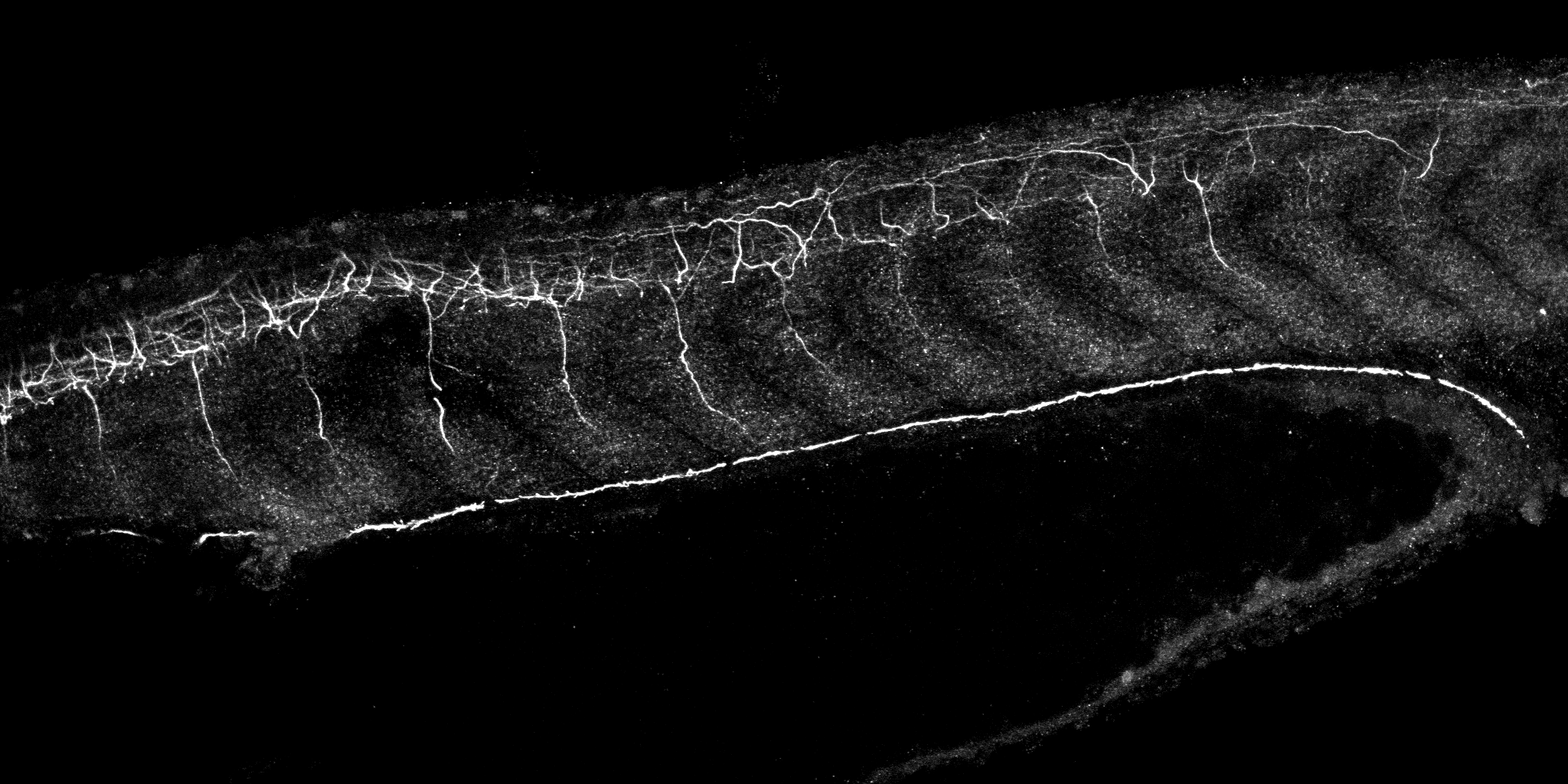

Supplement: Supplementary file 9 — Source data Fig. 4 [file 44318_2024_307_MOESM9_ESM.zip › EMBOJ-2024-116734_sourcedata_Fig 4/Fig.4A_polyE_26hpf.tif]

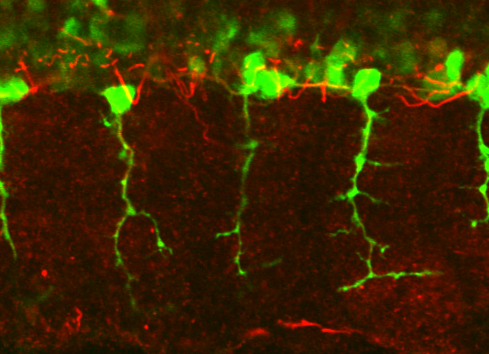

Supplement: Supplementary file 9 — Source data Fig. 4 [file 44318_2024_307_MOESM9_ESM.zip › EMBOJ-2024-116734_sourcedata_Fig 4/Fig4.B_MOTTLL6_polyEGFP.tif]

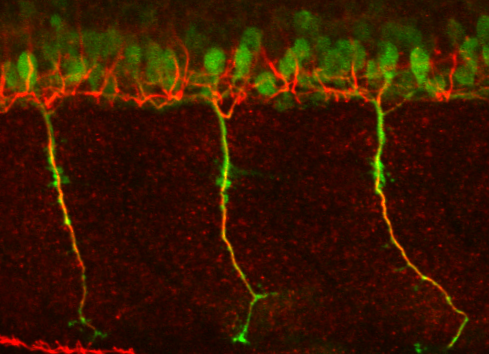

Supplement: Supplementary file 9 — Source data Fig. 4 [file 44318_2024_307_MOESM9_ESM.zip › EMBOJ-2024-116734_sourcedata_Fig 4/Fig4.B_MOCTL_polyEGFP.tif]

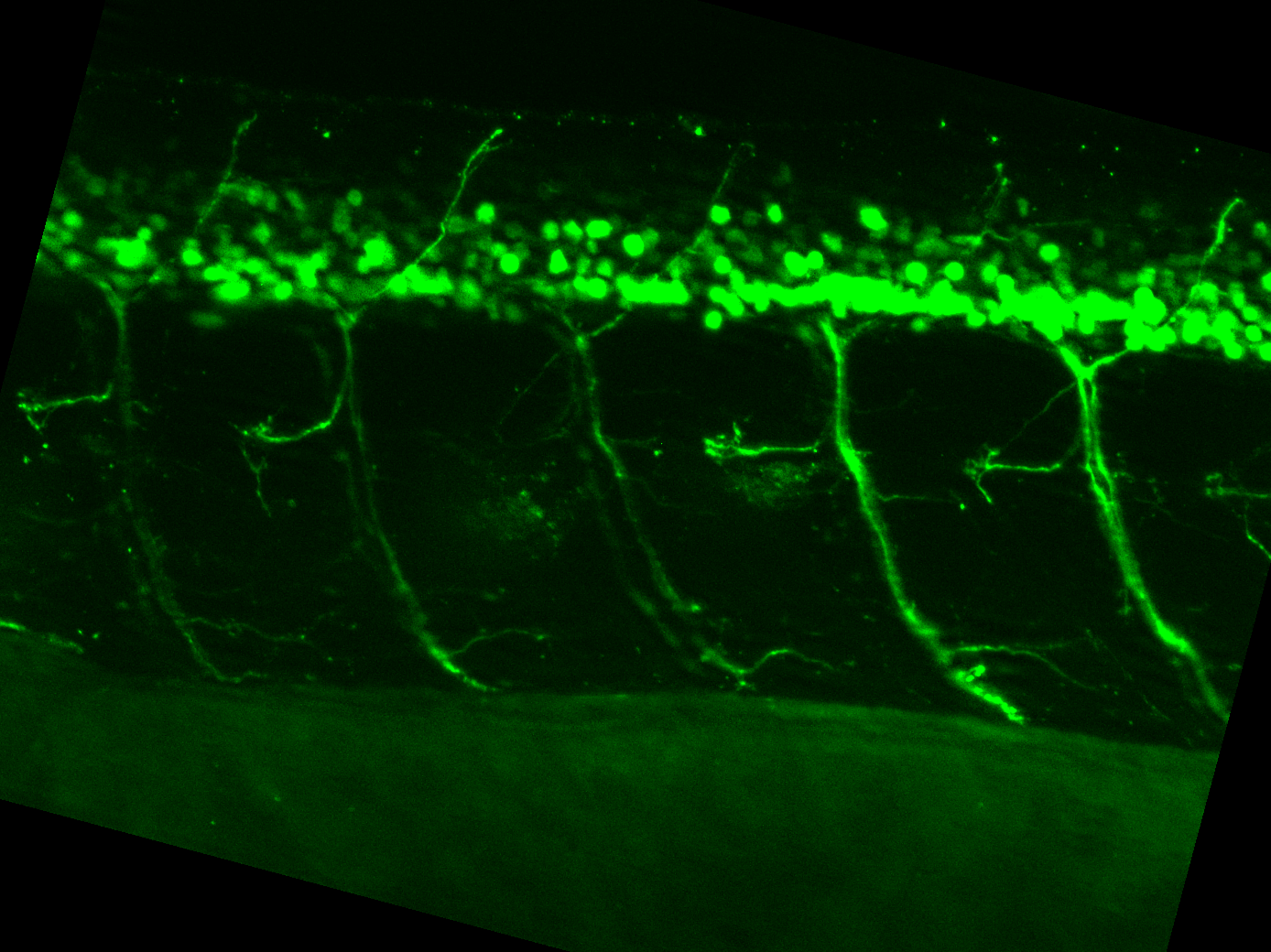

Supplement: Supplementary file 9 — Source data Fig. 4 [file 44318_2024_307_MOESM9_ESM.zip › EMBOJ-2024-116734_sourcedata_Fig 4/Fig.4D_MOCTL_smnsorting_GFP.tif]

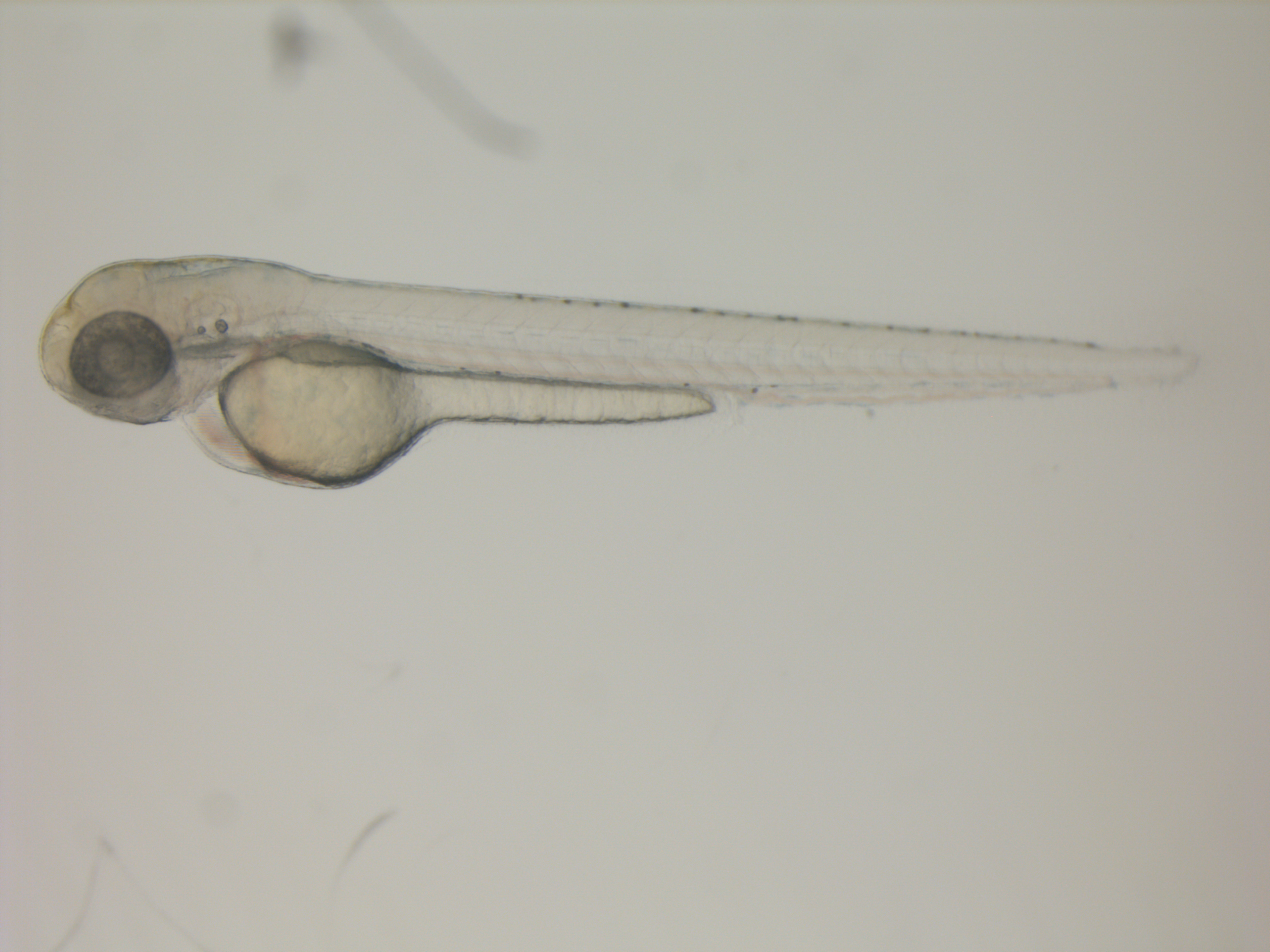

Supplement: Supplementary file 9 — Source data Fig. 4 [file 44318_2024_307_MOESM9_ESM.zip › EMBOJ-2024-116734_sourcedata_Fig 4/Fig4.D_MOCTL.tif]

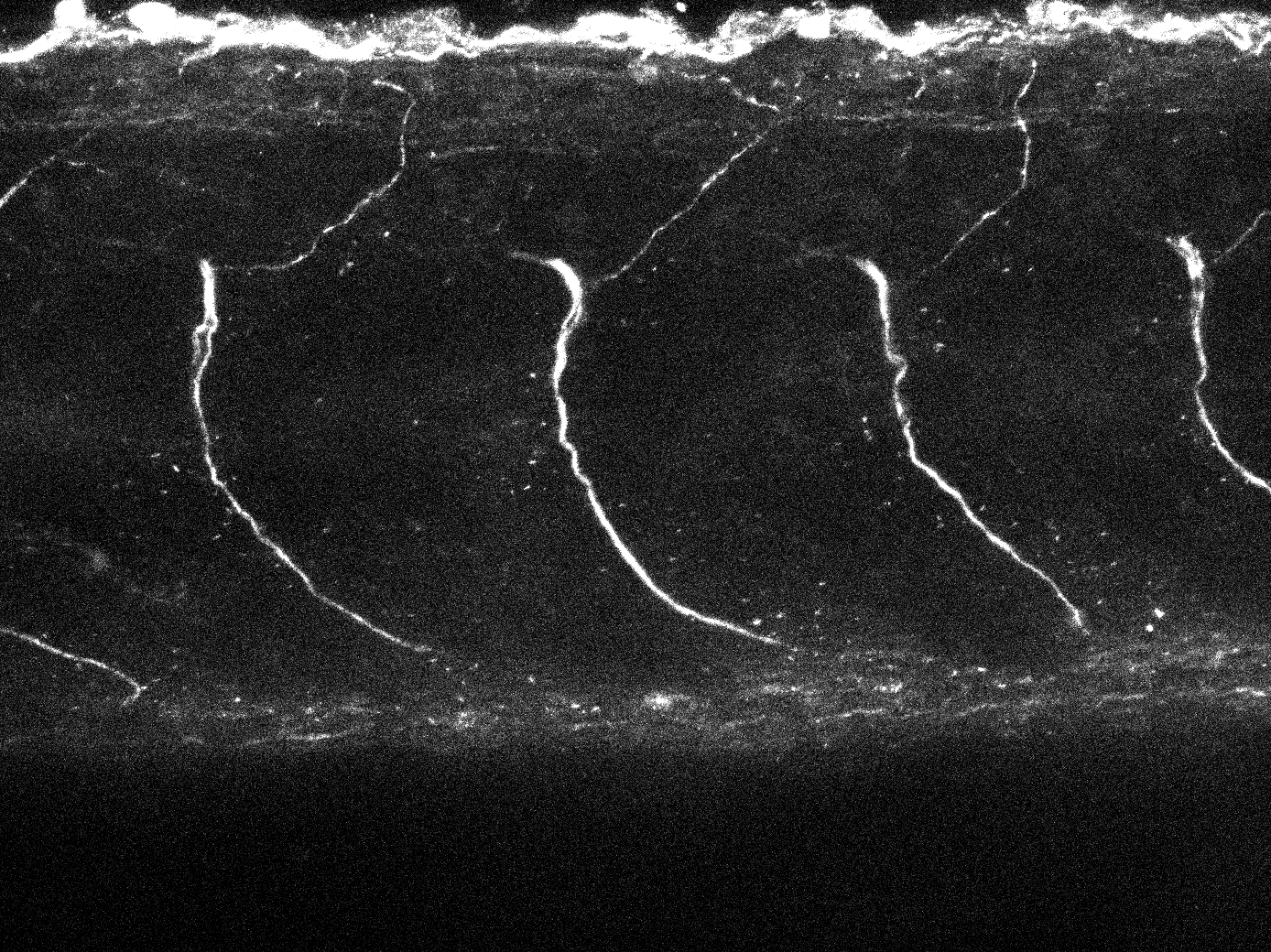

Supplement: Supplementary file 9 — Source data Fig. 4 [file 44318_2024_307_MOESM9_ESM.zip › EMBOJ-2024-116734_sourcedata_Fig 4/Fig.4A_GT335_26hpf.tif]

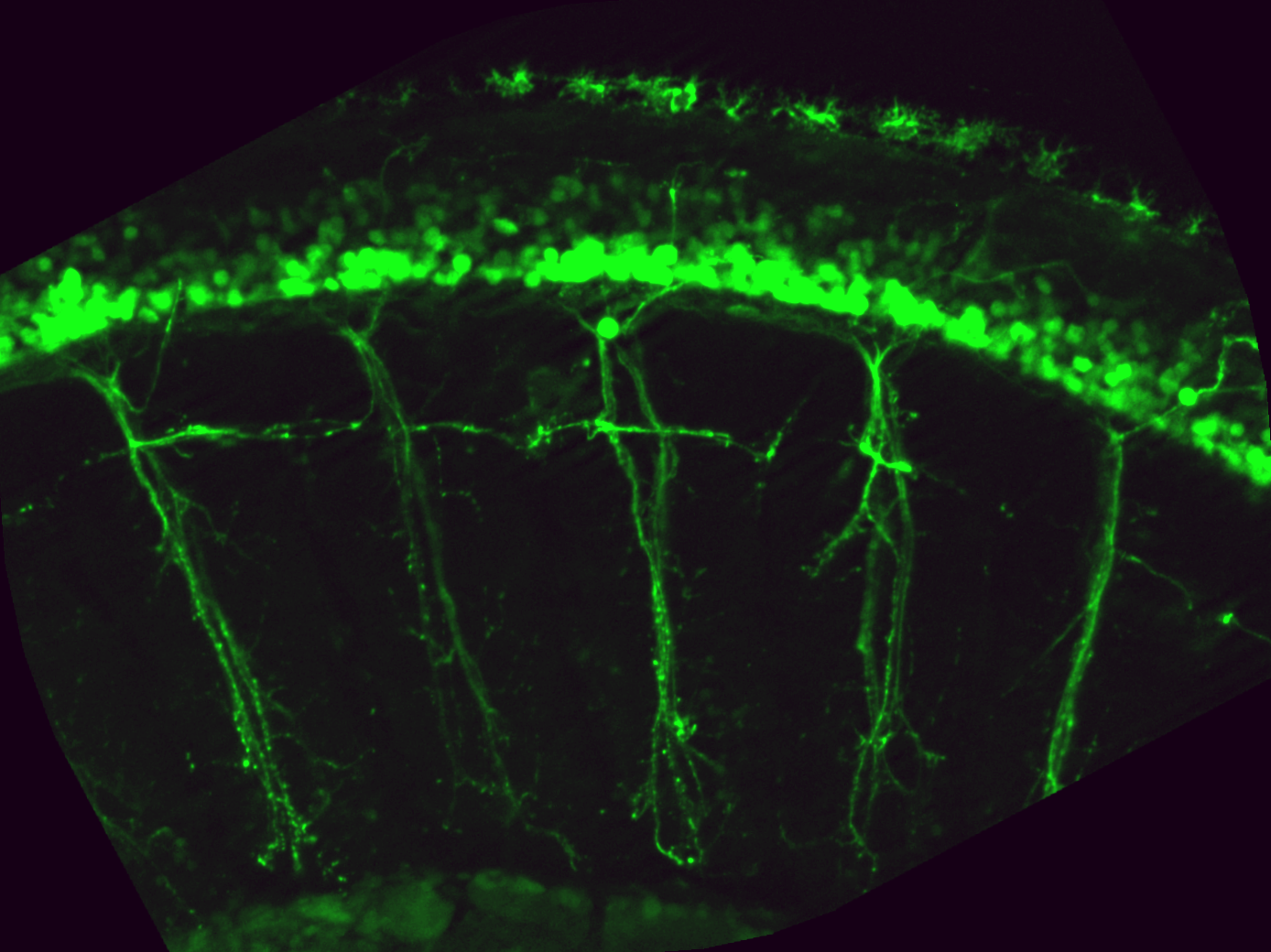

Supplement: Supplementary file 9 — Source data Fig. 4 [file 44318_2024_307_MOESM9_ESM.zip › EMBOJ-2024-116734_sourcedata_Fig 4/Fig.4D_MOTTLL11_smnsorting_GFP.tif]

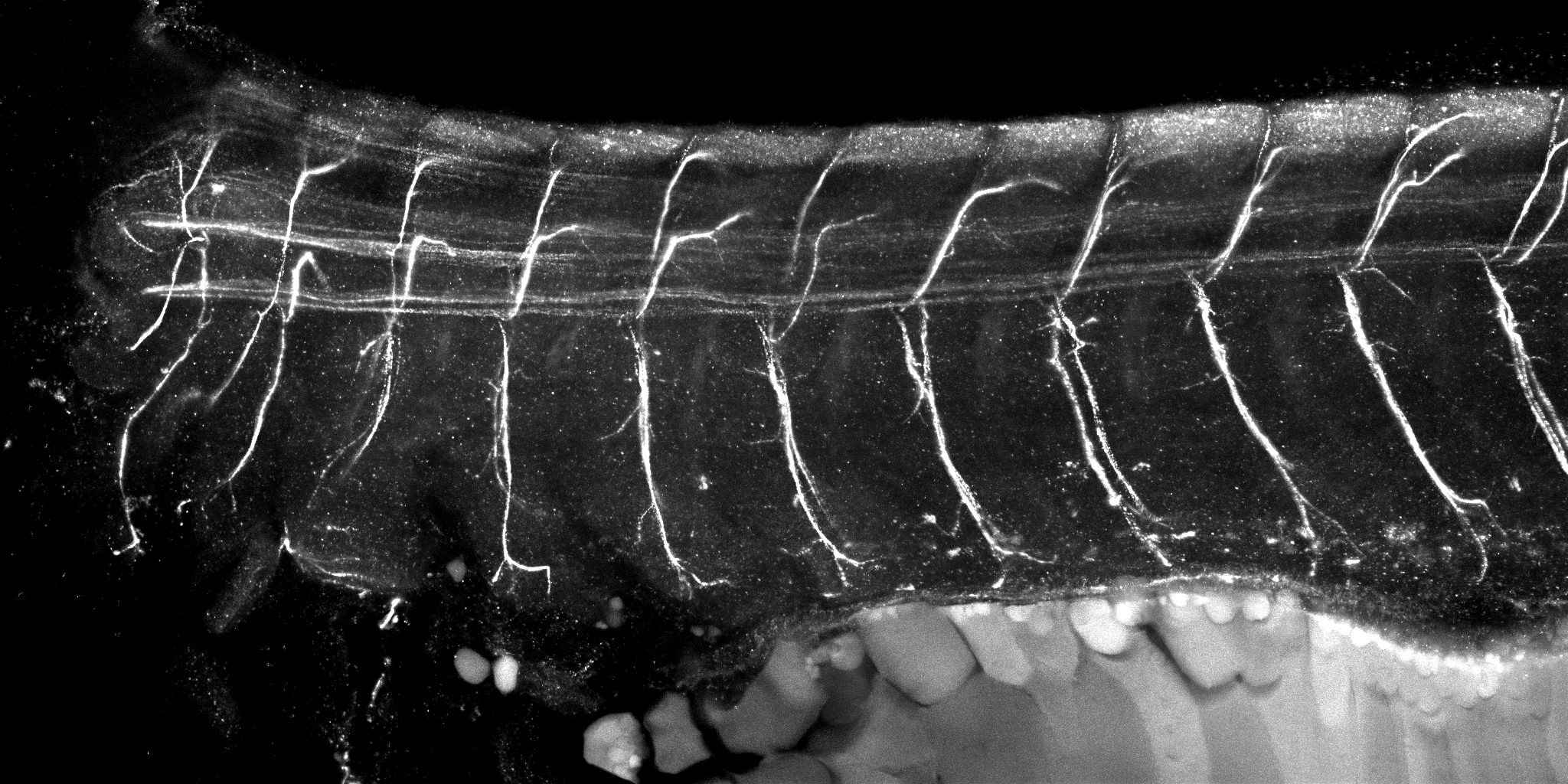

Supplement: Supplementary file 9 — Source data Fig. 4 [file 44318_2024_307_MOESM9_ESM.zip › EMBOJ-2024-116734_sourcedata_Fig 4/Fig.4A_polyE_72hpf.tif]

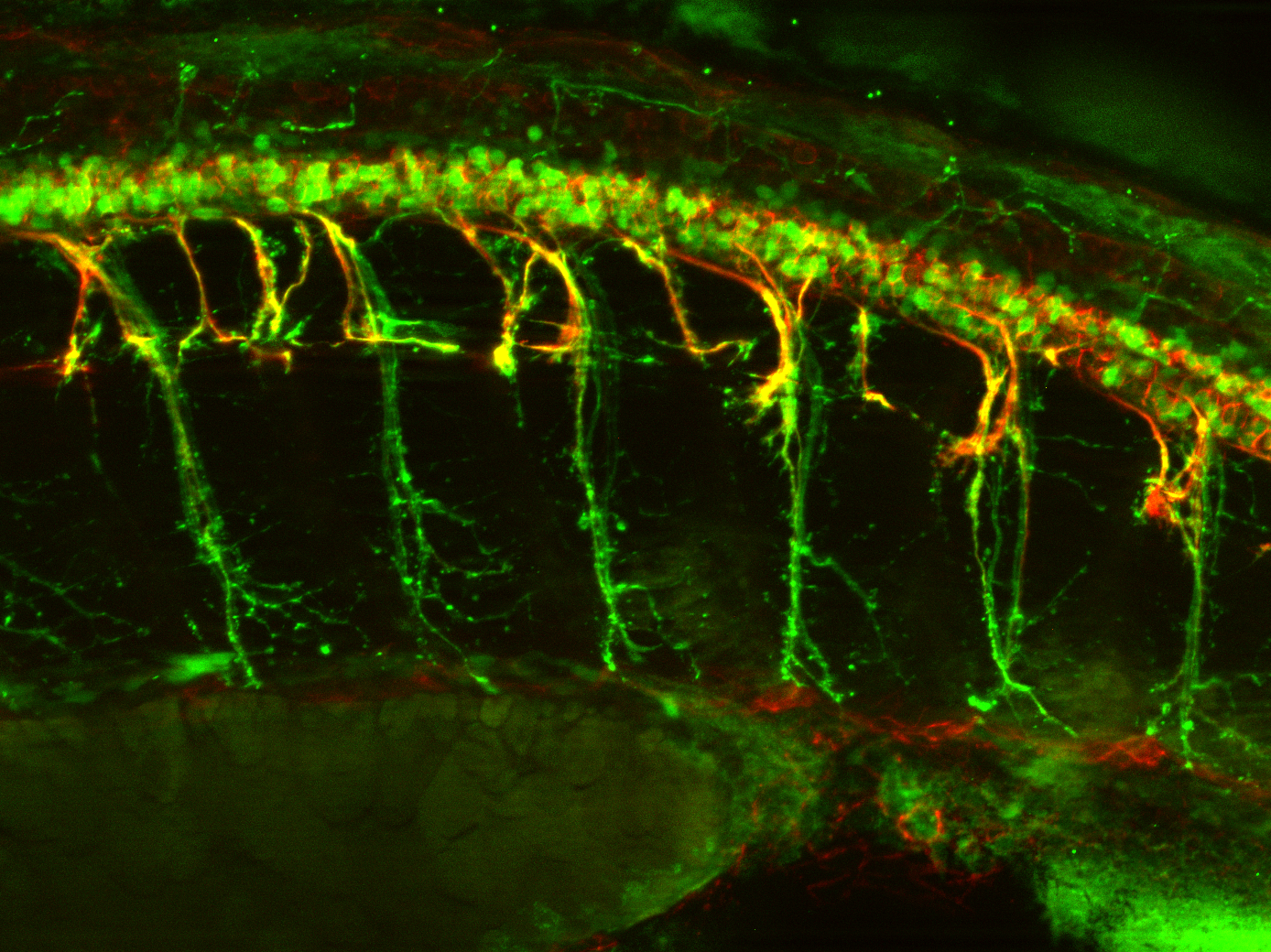

Supplement: Supplementary file 9 — Source data Fig. 4 [file 44318_2024_307_MOESM9_ESM.zip › EMBOJ-2024-116734_sourcedata_Fig 4/Fig.4D_MOTTLL11smnzn5GFP.tif]

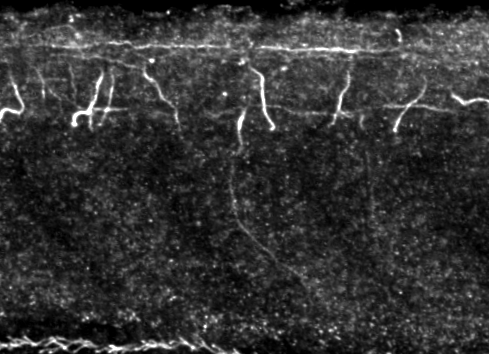

Supplement: Supplementary file 9 — Source data Fig. 4 [file 44318_2024_307_MOESM9_ESM.zip › EMBOJ-2024-116734_sourcedata_Fig 4/Fig4.B_MOTTLL11_polyE.tif]

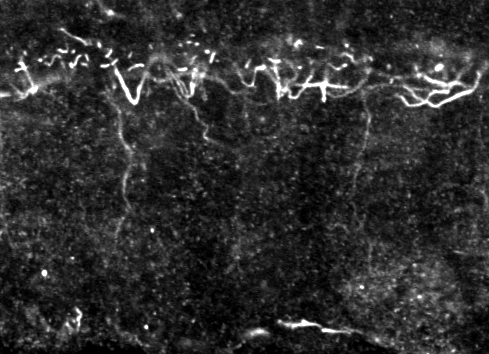

Supplement: Supplementary file 9 — Source data Fig. 4 [file 44318_2024_307_MOESM9_ESM.zip › EMBOJ-2024-116734_sourcedata_Fig 4/Fig4.B_MOTTLL6_polyE.tif]

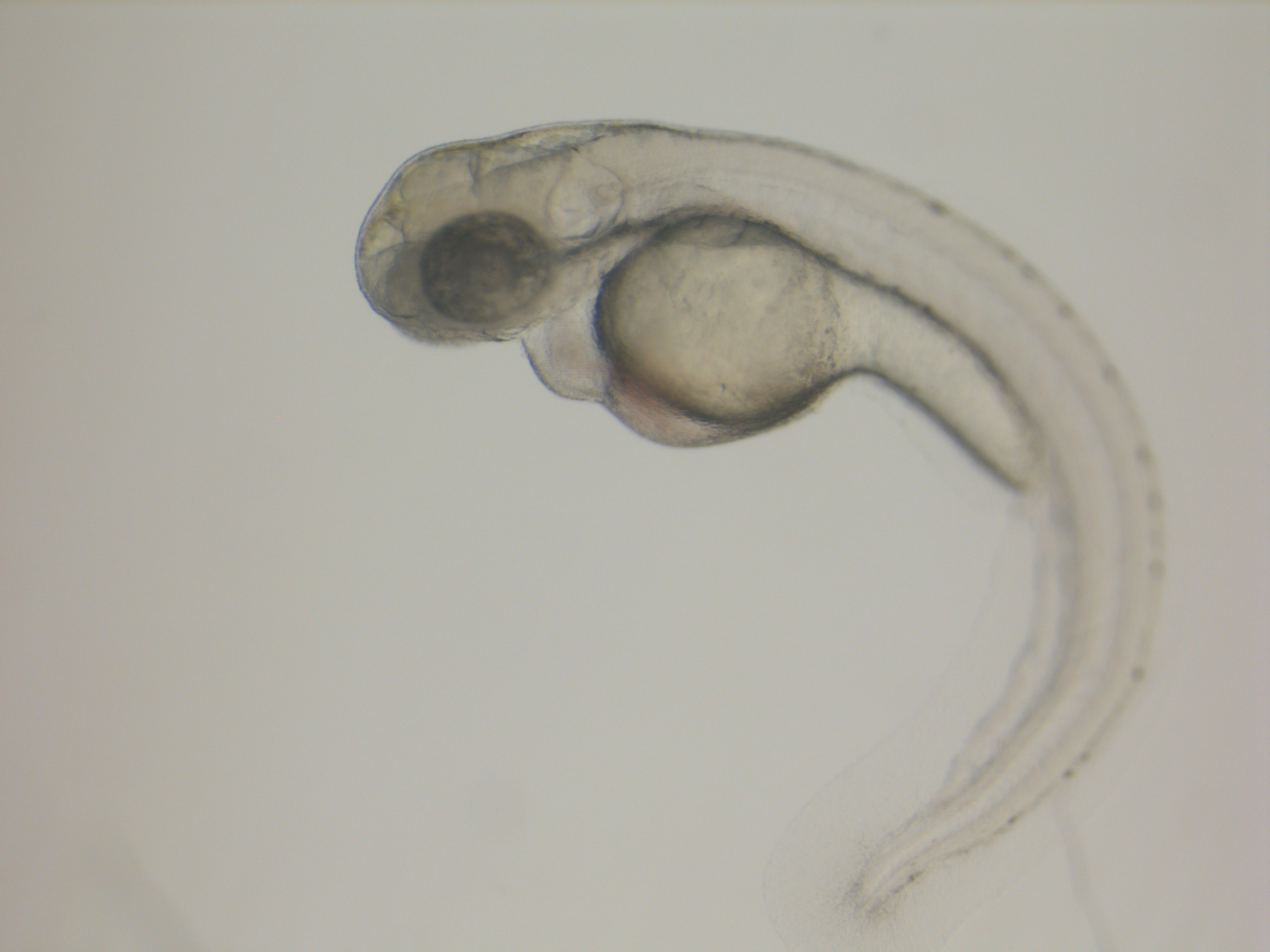

Supplement: Supplementary file 9 — Source data Fig. 4 [file 44318_2024_307_MOESM9_ESM.zip › EMBOJ-2024-116734_sourcedata_Fig 4/Fig4.D_MOTTLL6.tif]

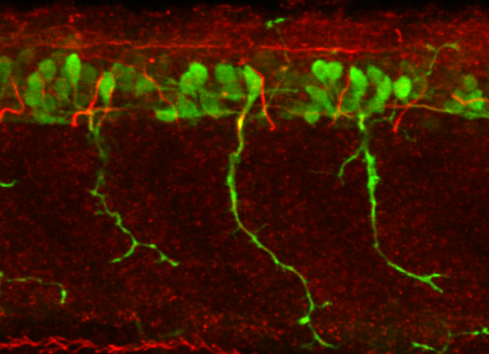

Supplement: Supplementary file 9 — Source data Fig. 4 [file 44318_2024_307_MOESM9_ESM.zip › EMBOJ-2024-116734_sourcedata_Fig 4/Fig4.B_MOTTLL11_polyEGFP.tif]

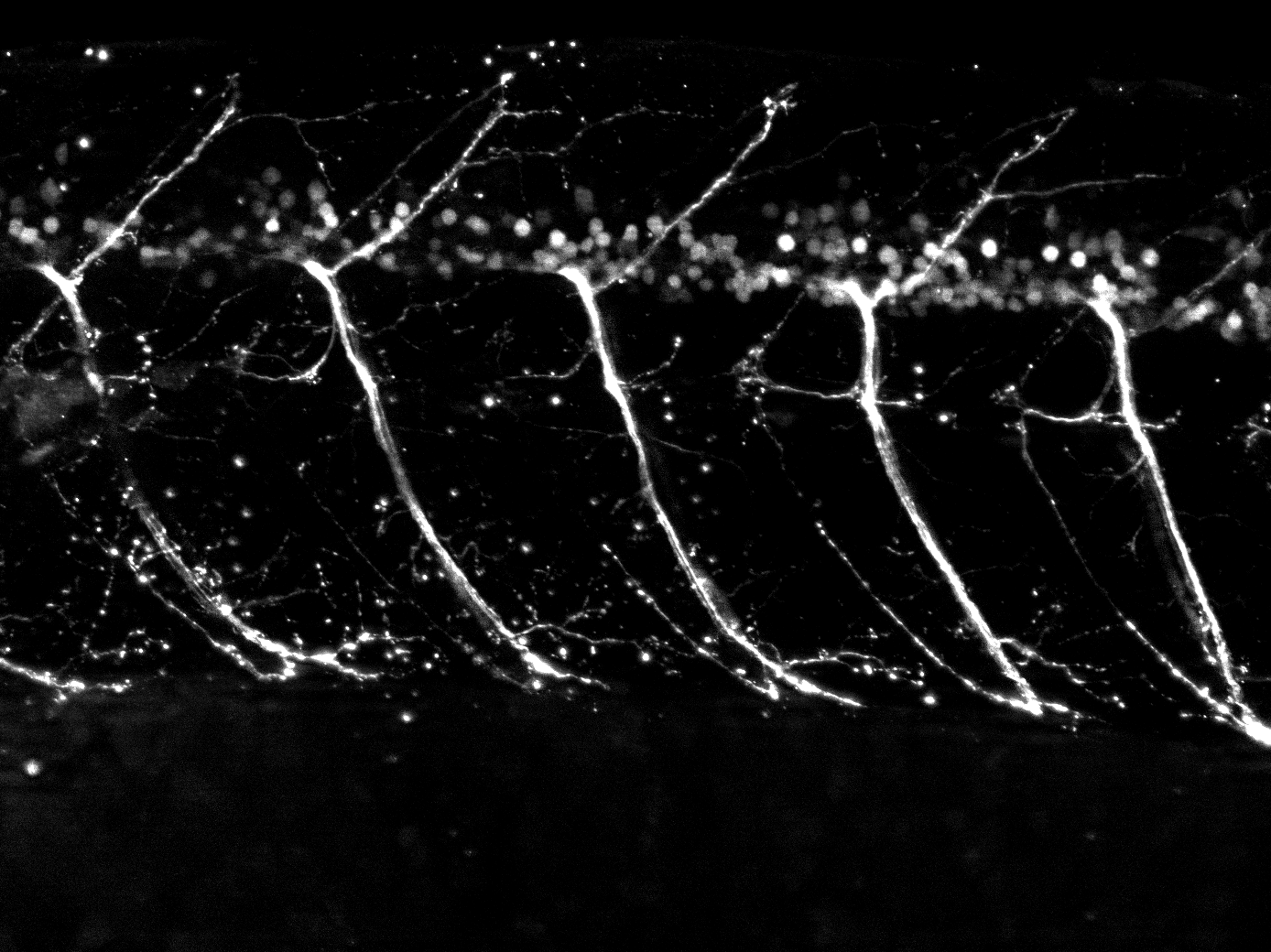

Supplement: Supplementary file 10 — Source data Fig. 5 [file 44318_2024_307_MOESM10_ESM.zip › EMBOJ-2024-116734_sourcedata_Fig 5/Fig5_panelB_MoCTL_sMN_zn5GFP.tif]

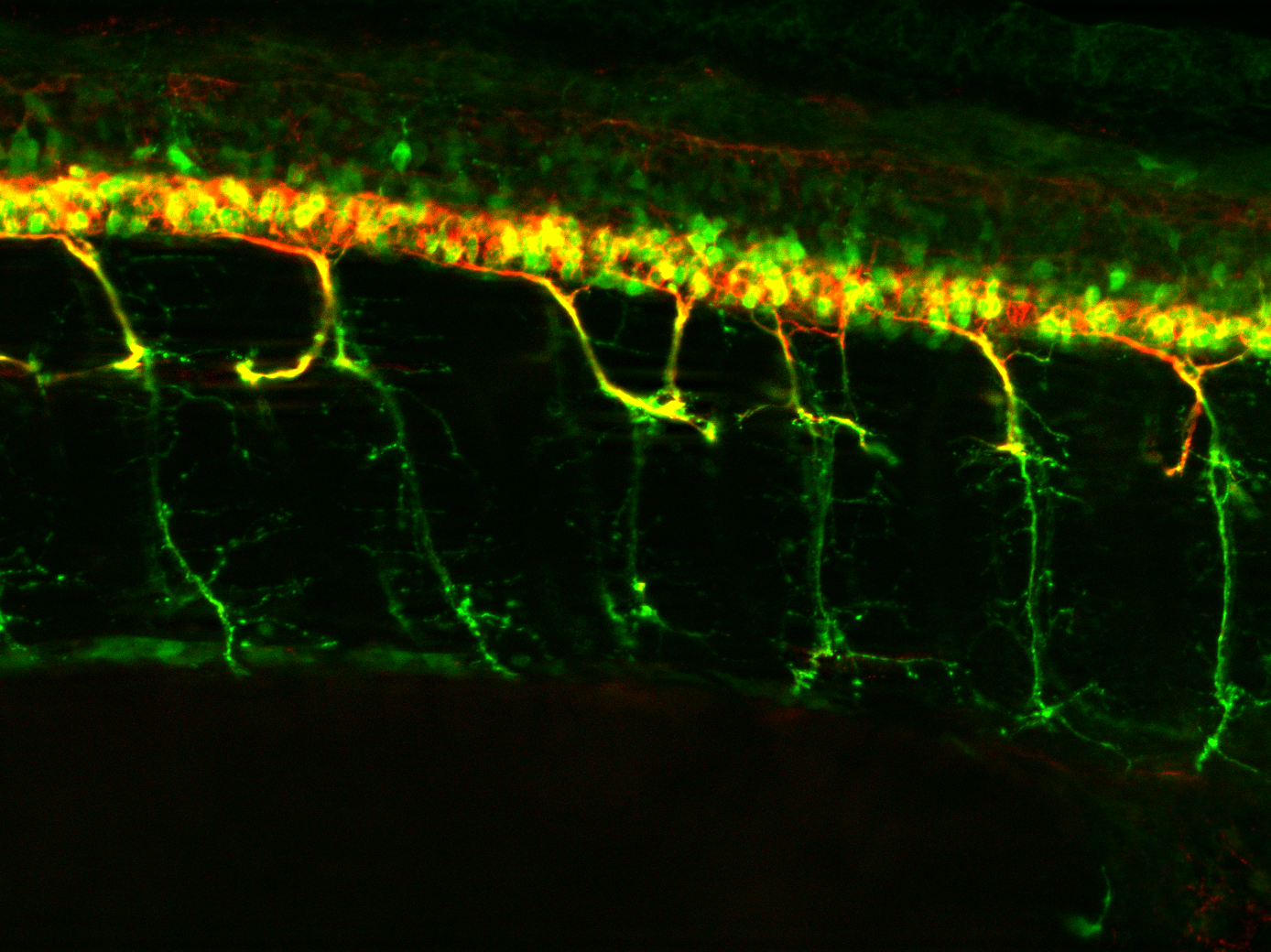

Supplement: Supplementary file 10 — Source data Fig. 5 [file 44318_2024_307_MOESM10_ESM.zip › EMBOJ-2024-116734_sourcedata_Fig 5/Fig5_panelB_MoTTLL11andTTLL6mRNA_sMN_zn5GFP.tif]

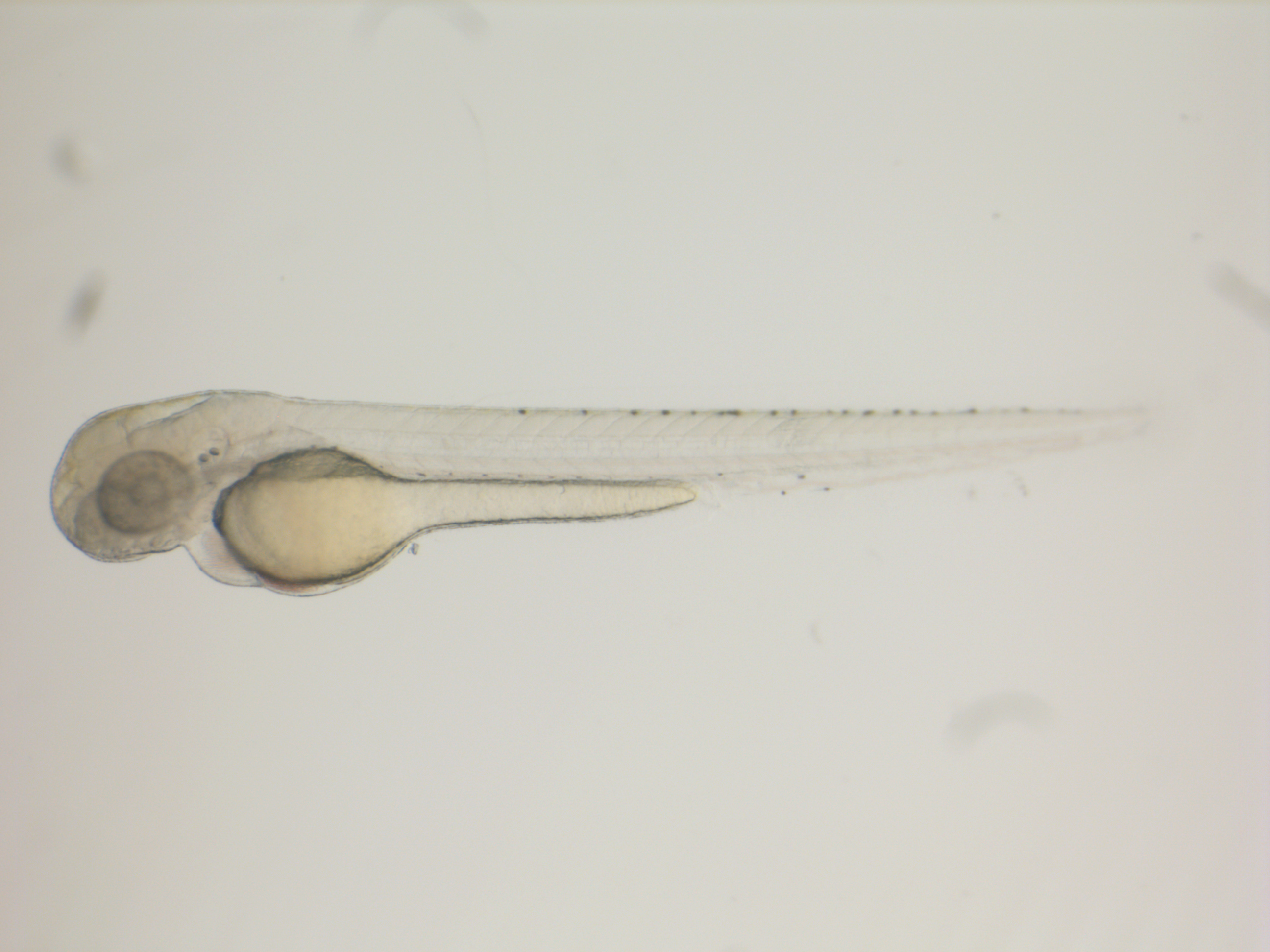

Supplement: Supplementary file 10 — Source data Fig. 5 [file 44318_2024_307_MOESM10_ESM.zip › EMBOJ-2024-116734_sourcedata_Fig 5/Fig5_panelB_MoCTL_morpho.tif]

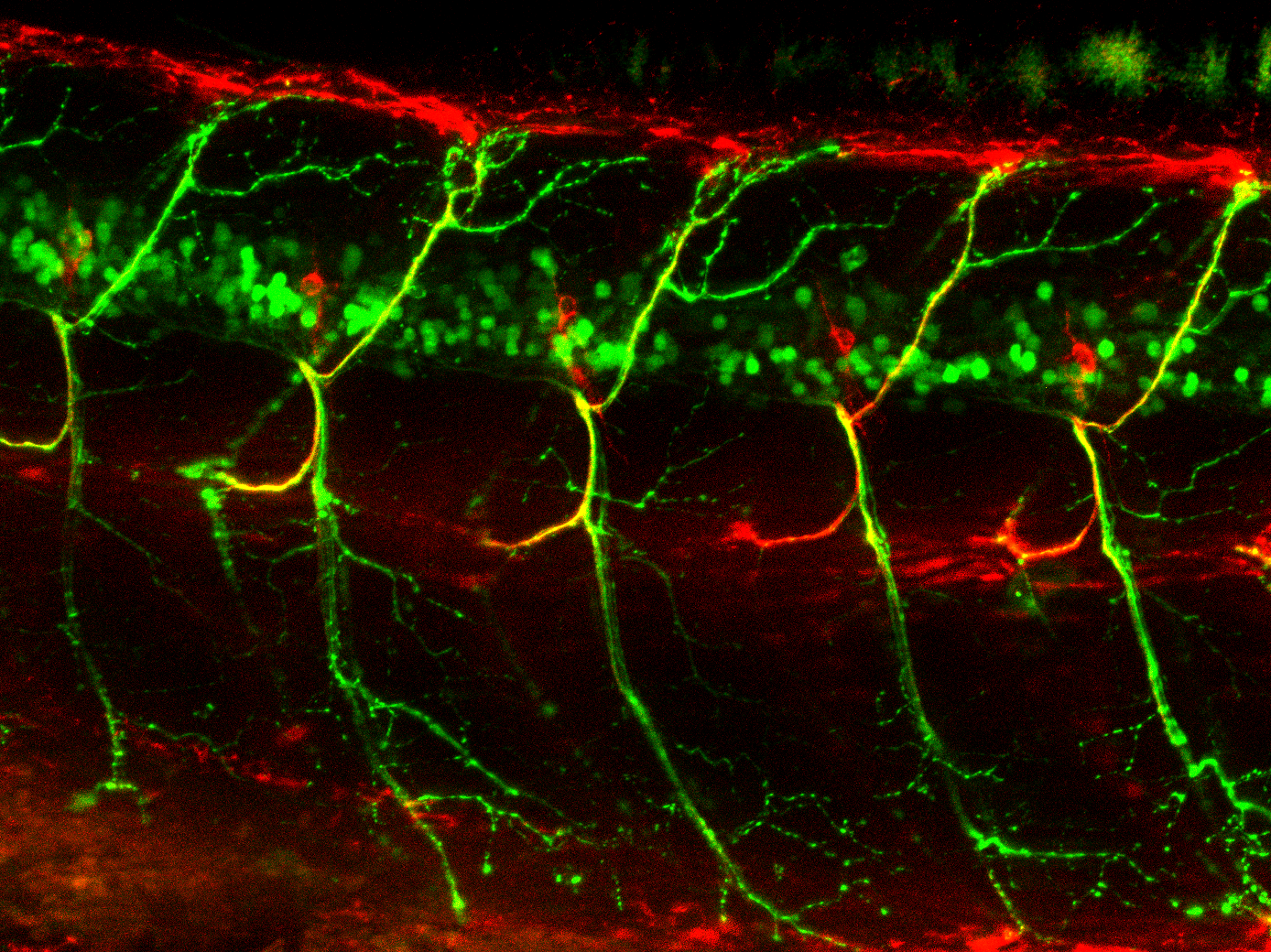

Supplement: Supplementary file 10 — Source data Fig. 5 [file 44318_2024_307_MOESM10_ESM.zip › EMBOJ-2024-116734_sourcedata_Fig 5/Fig5_panelB_MoTTLL11andTTLL11mRNA_sMN_zn5GFP.tif]

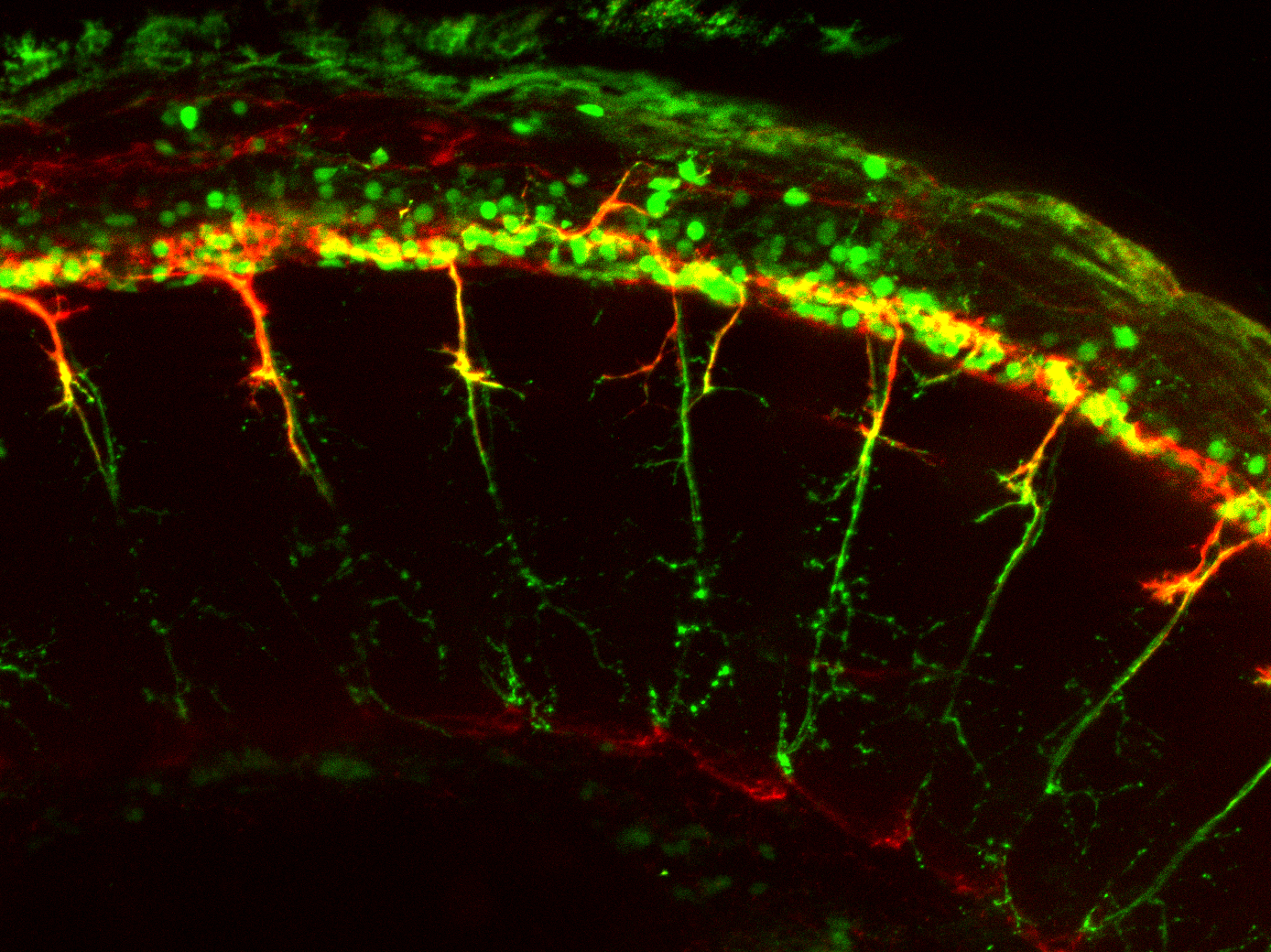

Supplement: Supplementary file 10 — Source data Fig. 5 [file 44318_2024_307_MOESM10_ESM.zip › EMBOJ-2024-116734_sourcedata_Fig 5/Fig5_panelB_MoTTLL11_sMN_zn5GFP.tif]

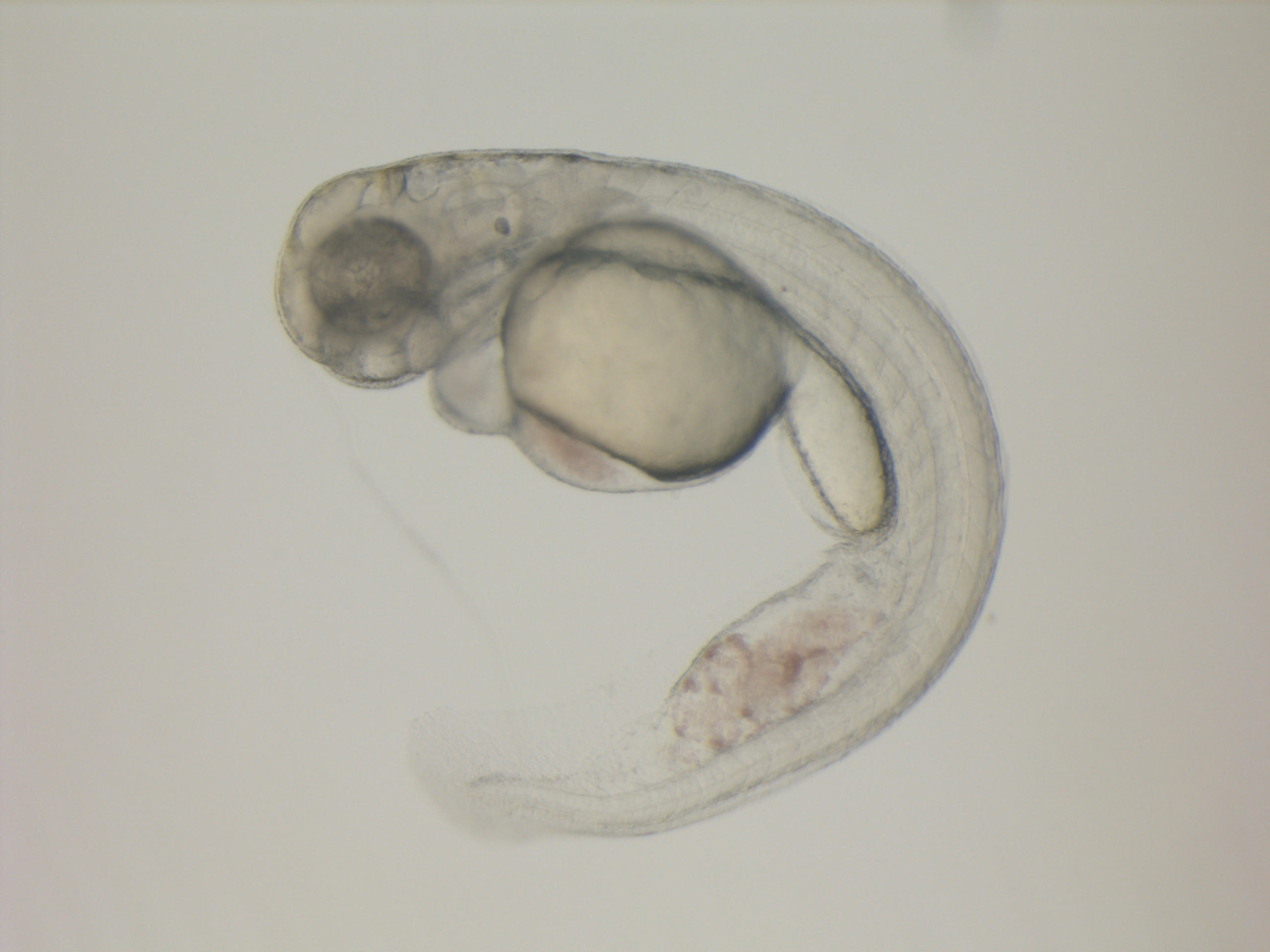

Supplement: Supplementary file 10 — Source data Fig. 5 [file 44318_2024_307_MOESM10_ESM.zip › EMBOJ-2024-116734_sourcedata_Fig 5/Fig5_panelB_MoTTLL11andTTLL6mRNA_morpho.tif]

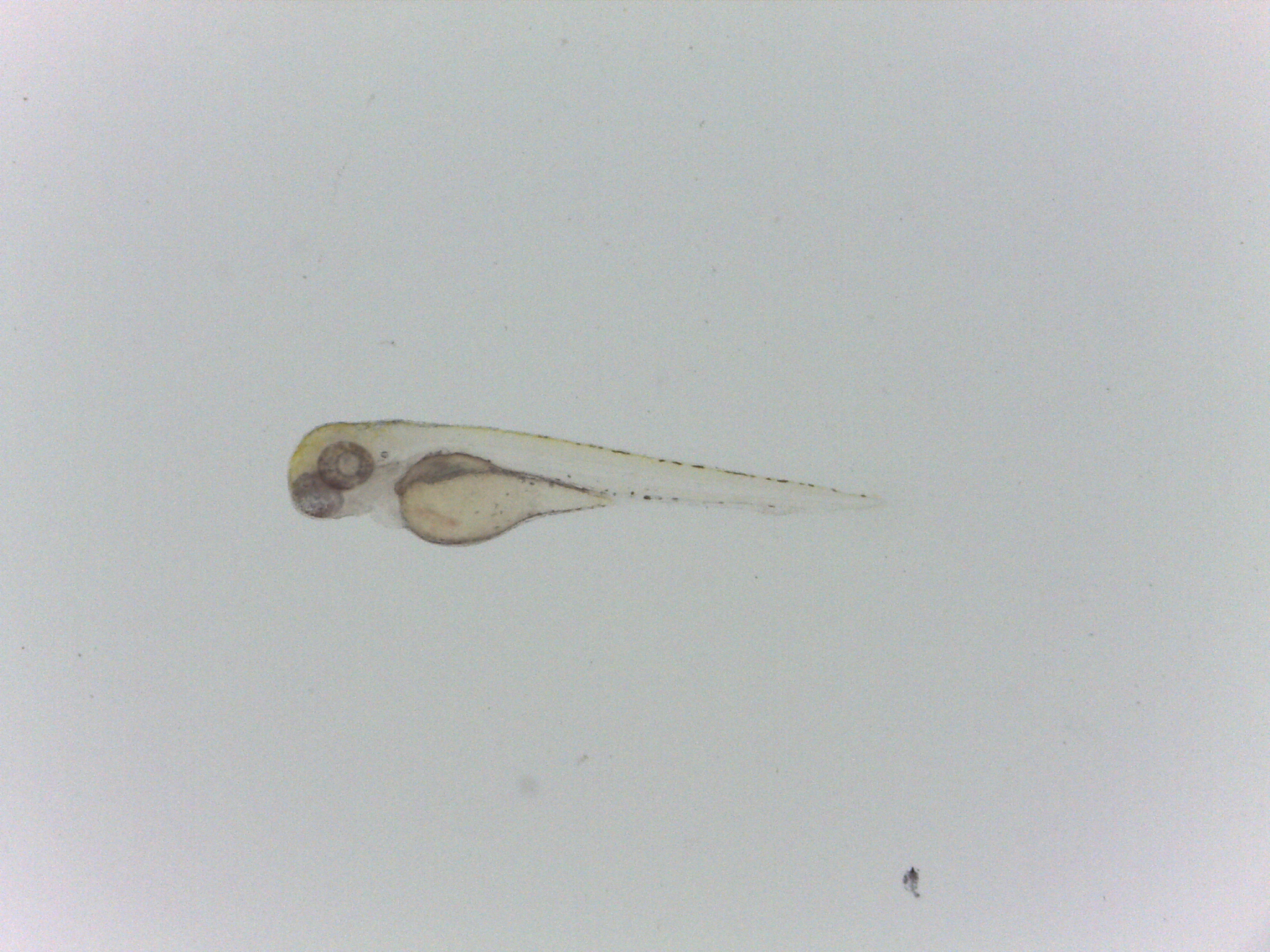

Supplement: Supplementary file 10 — Source data Fig. 5 [file 44318_2024_307_MOESM10_ESM.zip › EMBOJ-2024-116734_sourcedata_Fig 5/Fig5_panelA_MoTTLL6andTTLL6mRNA_morpho.tif]

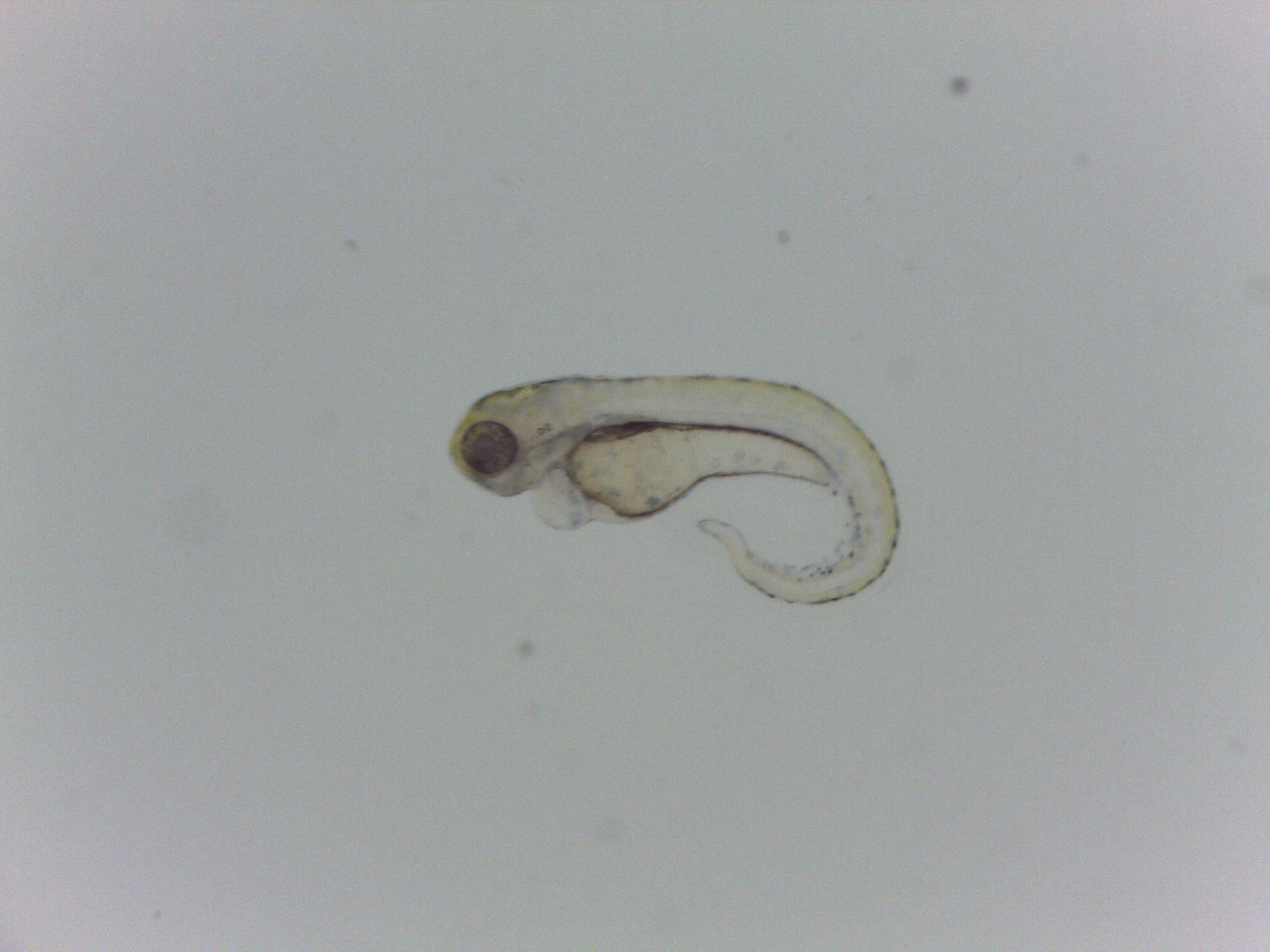

Supplement: Supplementary file 10 — Source data Fig. 5 [file 44318_2024_307_MOESM10_ESM.zip › EMBOJ-2024-116734_sourcedata_Fig 5/Fig5_panelB_MoTTLL11_morpho.tif]

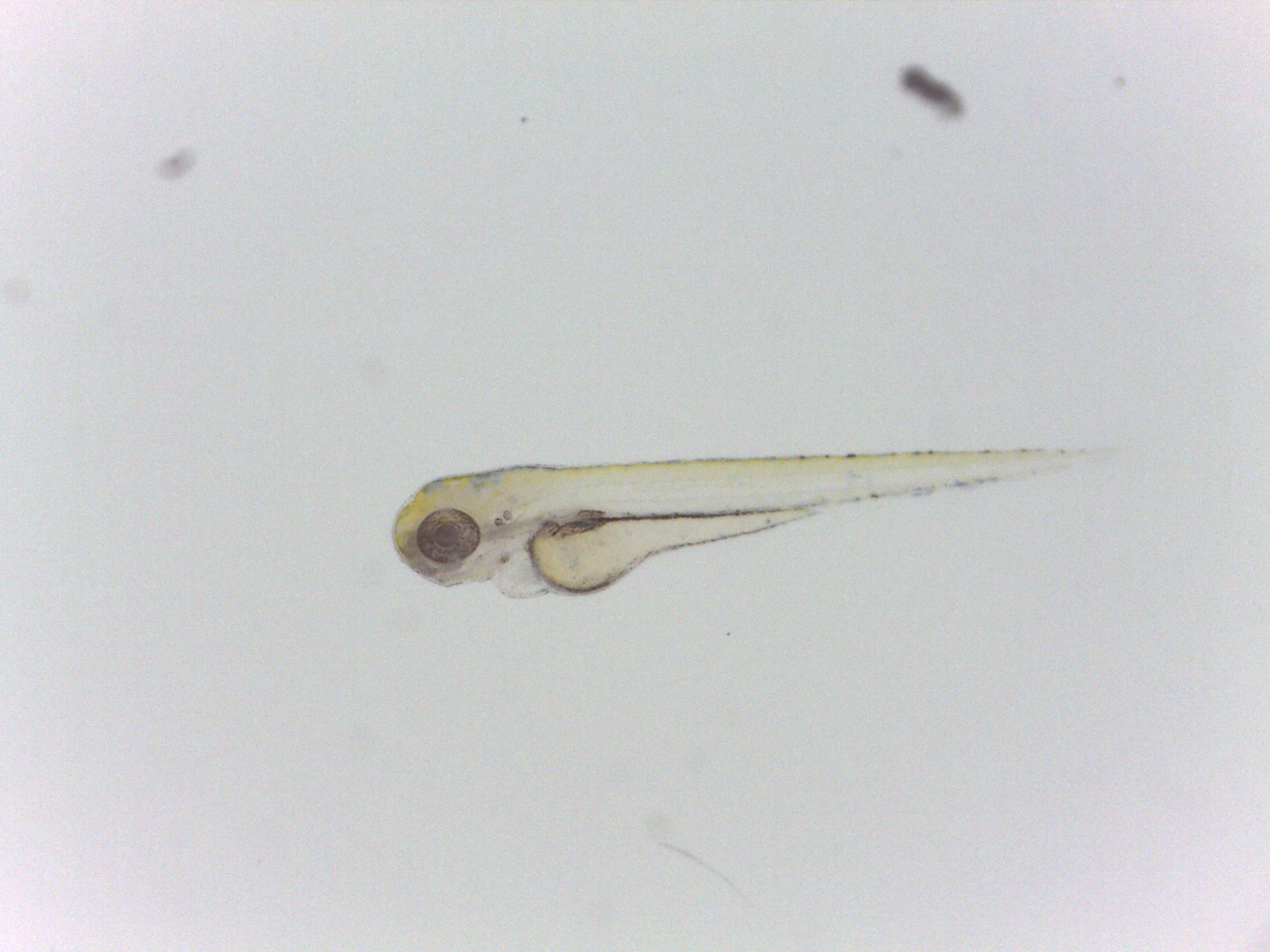

Supplement: Supplementary file 10 — Source data Fig. 5 [file 44318_2024_307_MOESM10_ESM.zip › EMBOJ-2024-116734_sourcedata_Fig 5/Fig5_panelB_MoTTLL11andTTLL11mRNA_morpho.tif]

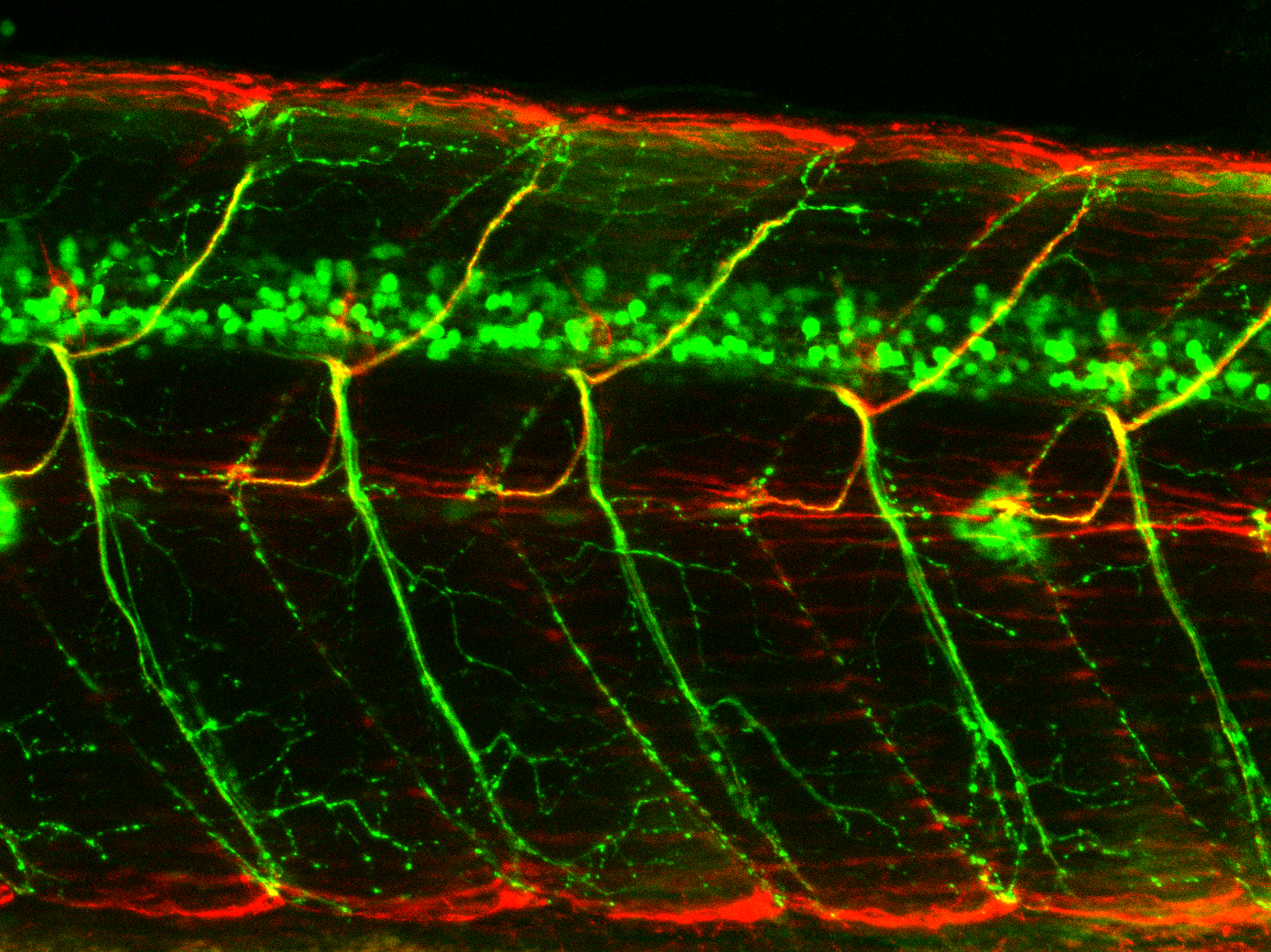

Supplement: Supplementary file 10 — Source data Fig. 5 [file 44318_2024_307_MOESM10_ESM.zip › EMBOJ-2024-116734_sourcedata_Fig 5/Fig5_panelA_MoCtlsMN_zn5GFP.tif]

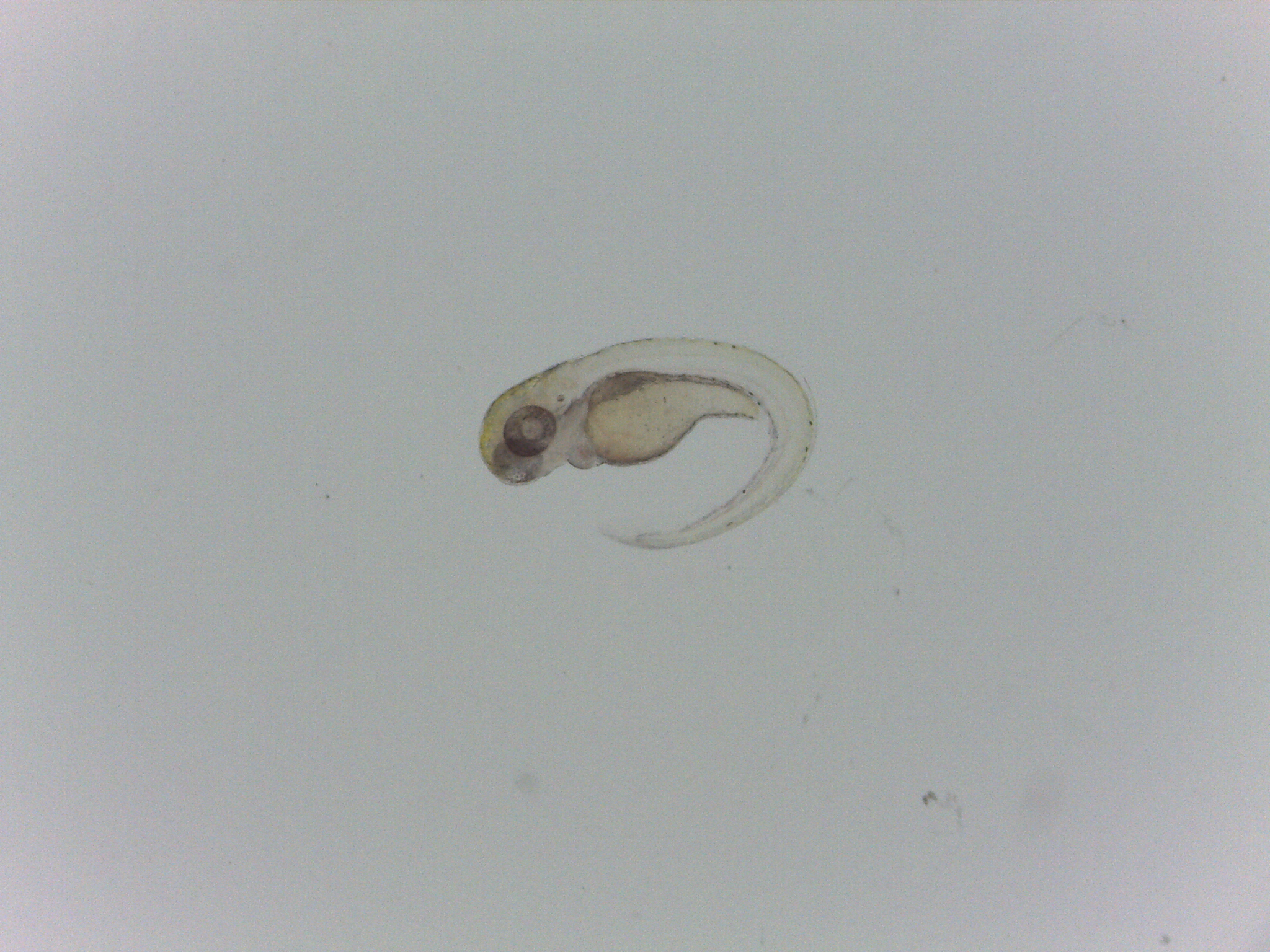

Supplement: Supplementary file 10 — Source data Fig. 5 [file 44318_2024_307_MOESM10_ESM.zip › EMBOJ-2024-116734_sourcedata_Fig 5/Fig5_panelA_MoTTLL6_morpho.tif]

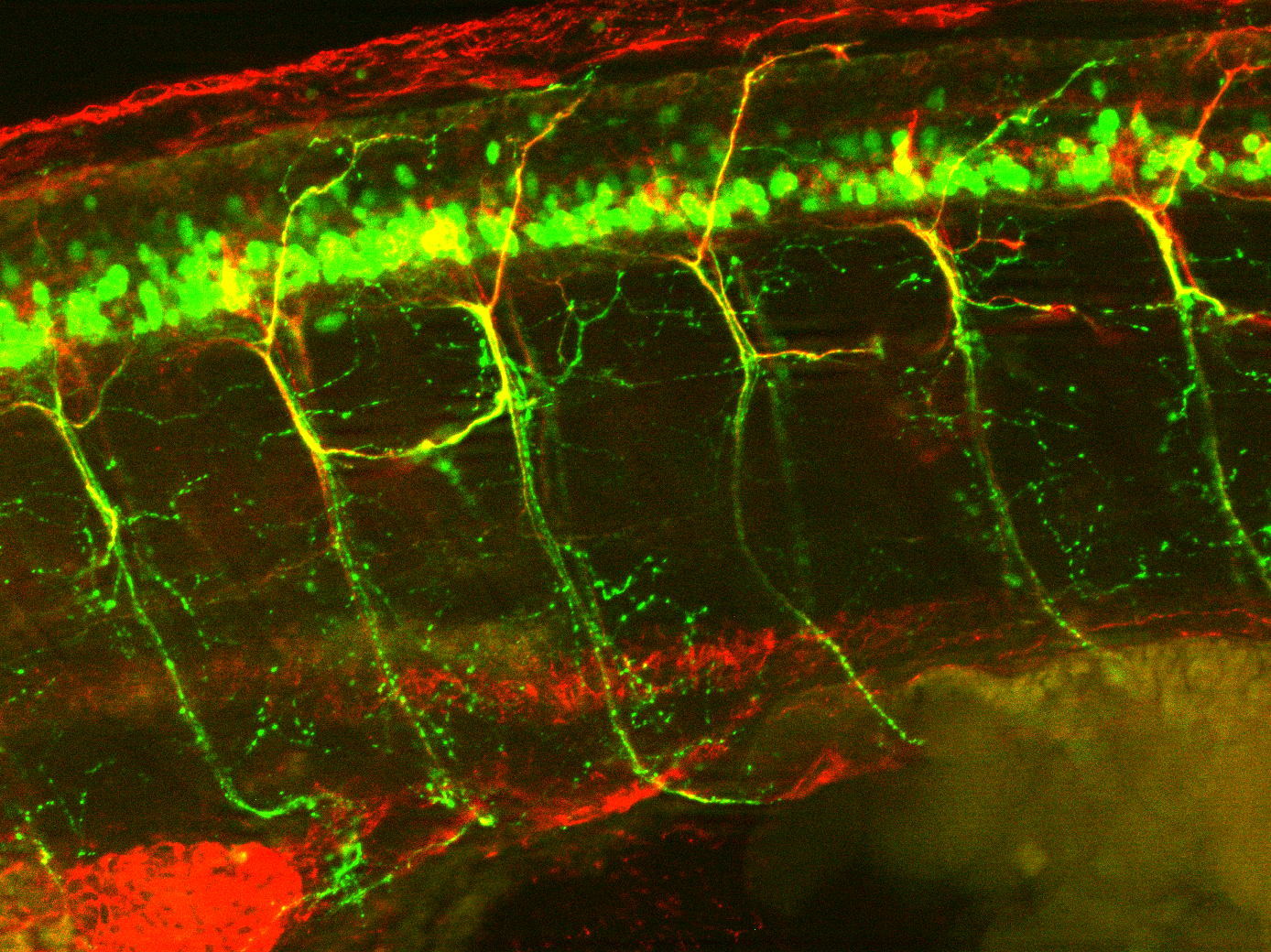

Supplement: Supplementary file 10 — Source data Fig. 5 [file 44318_2024_307_MOESM10_ESM.zip › EMBOJ-2024-116734_sourcedata_Fig 5/Fig5_panelA_MoTTLL6_sMN_zn5GFP.tif]

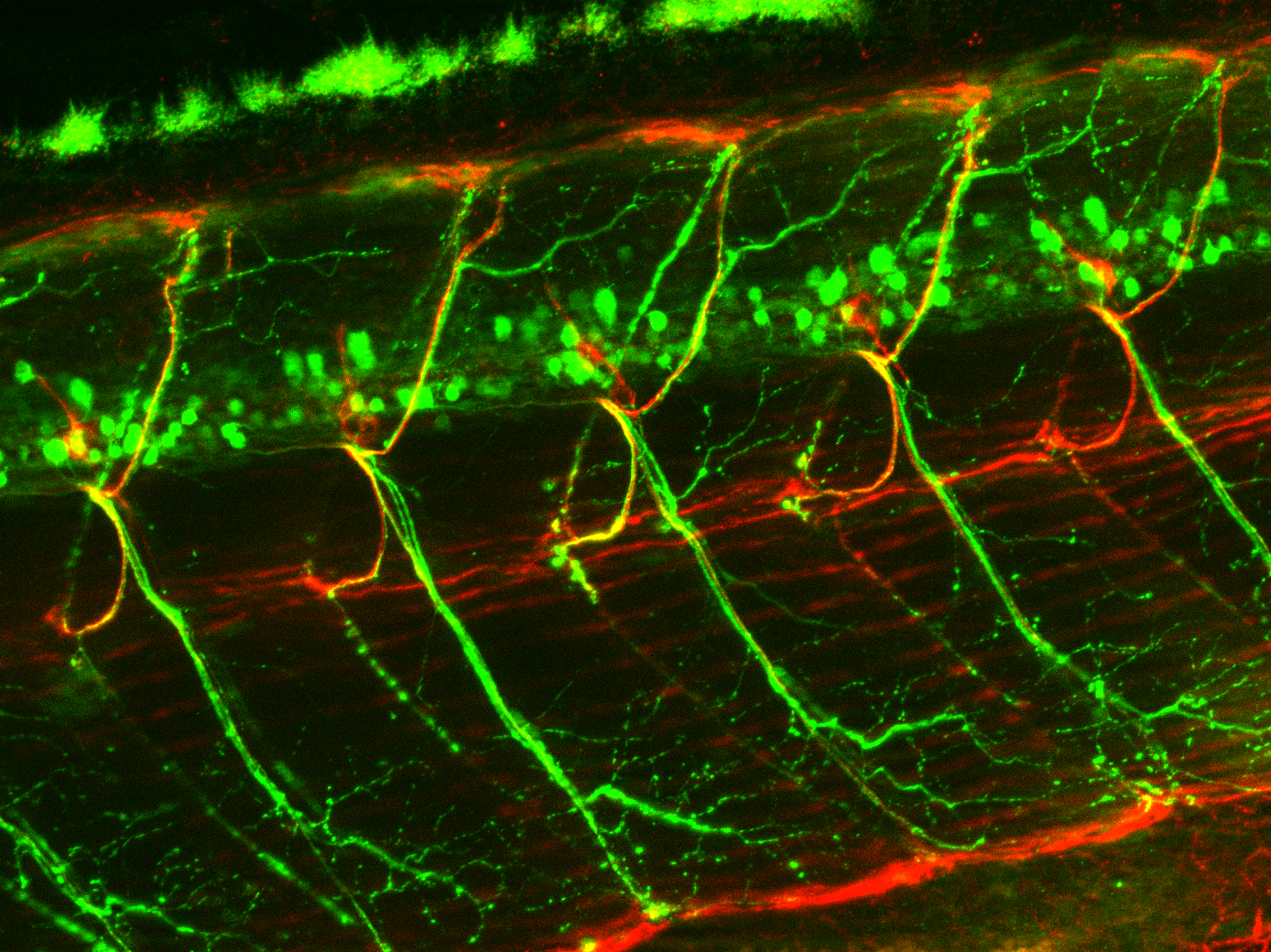

Supplement: Supplementary file 10 — Source data Fig. 5 [file 44318_2024_307_MOESM10_ESM.zip › EMBOJ-2024-116734_sourcedata_Fig 5/Fig5_panelA_MoTTLL6andTTLL6mRNA_sMN_zn5GFP.tif]

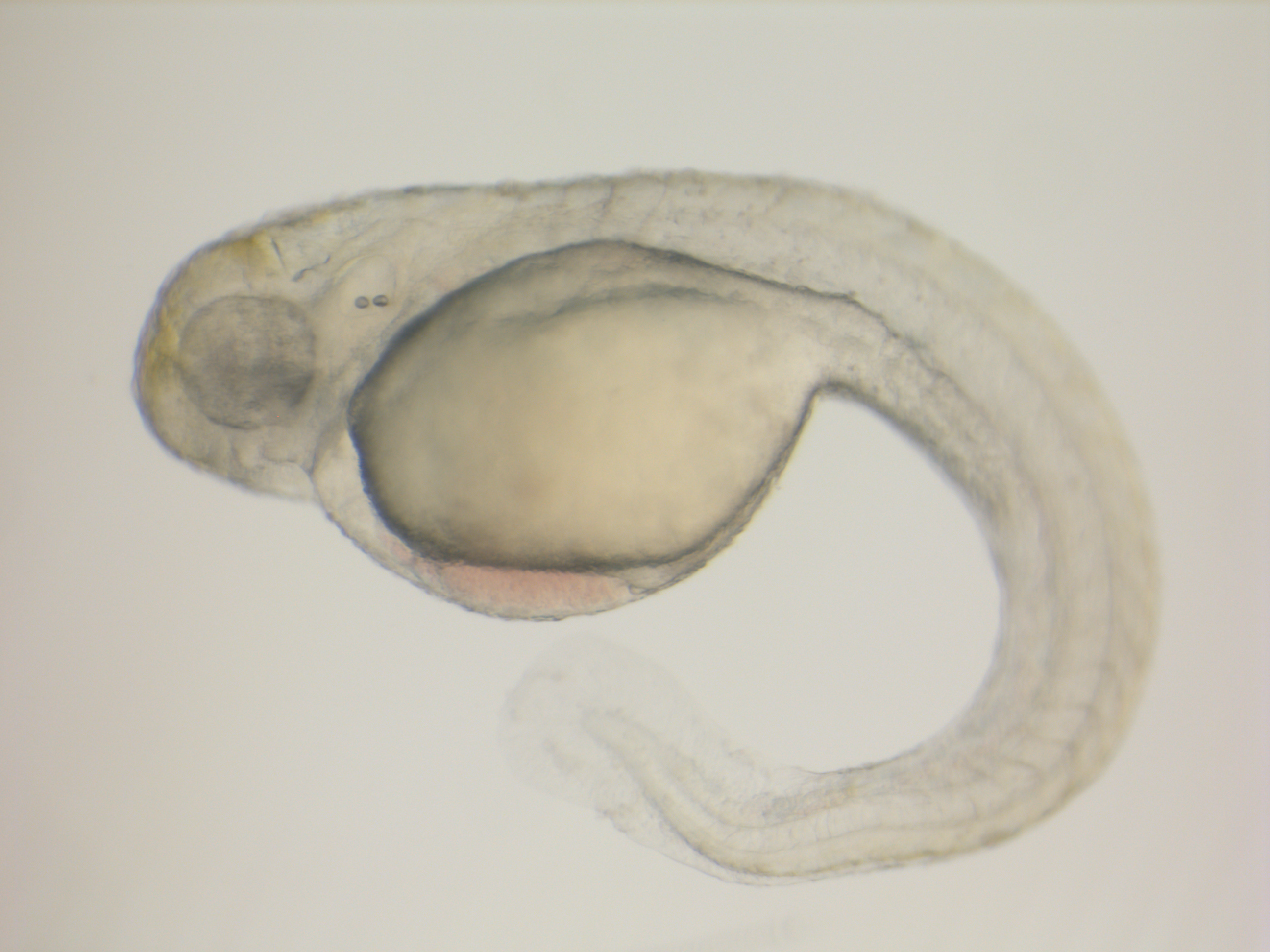

Supplement: Supplementary file 10 — Source data Fig. 5 [file 44318_2024_307_MOESM10_ESM.zip › EMBOJ-2024-116734_sourcedata_Fig 5/Fig5_panelA_MoTTLL6andTTLL11mRNA_morpho.tif]

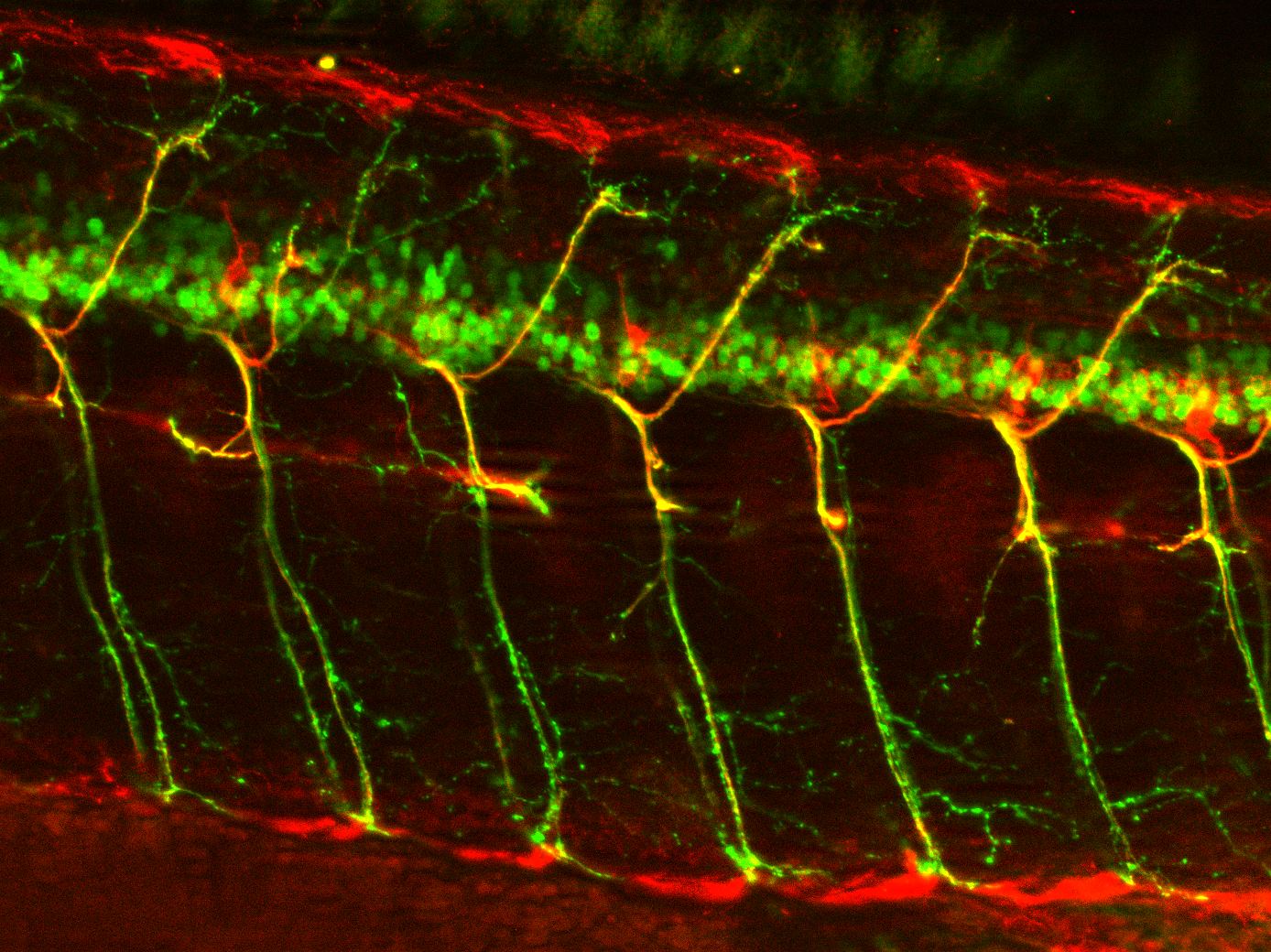

Supplement: Supplementary file 10 — Source data Fig. 5 [file 44318_2024_307_MOESM10_ESM.zip › EMBOJ-2024-116734_sourcedata_Fig 5/Fig5_panelA_MoTTLL6andTTLL11mRNA_sMN_zn5GFP.tif]

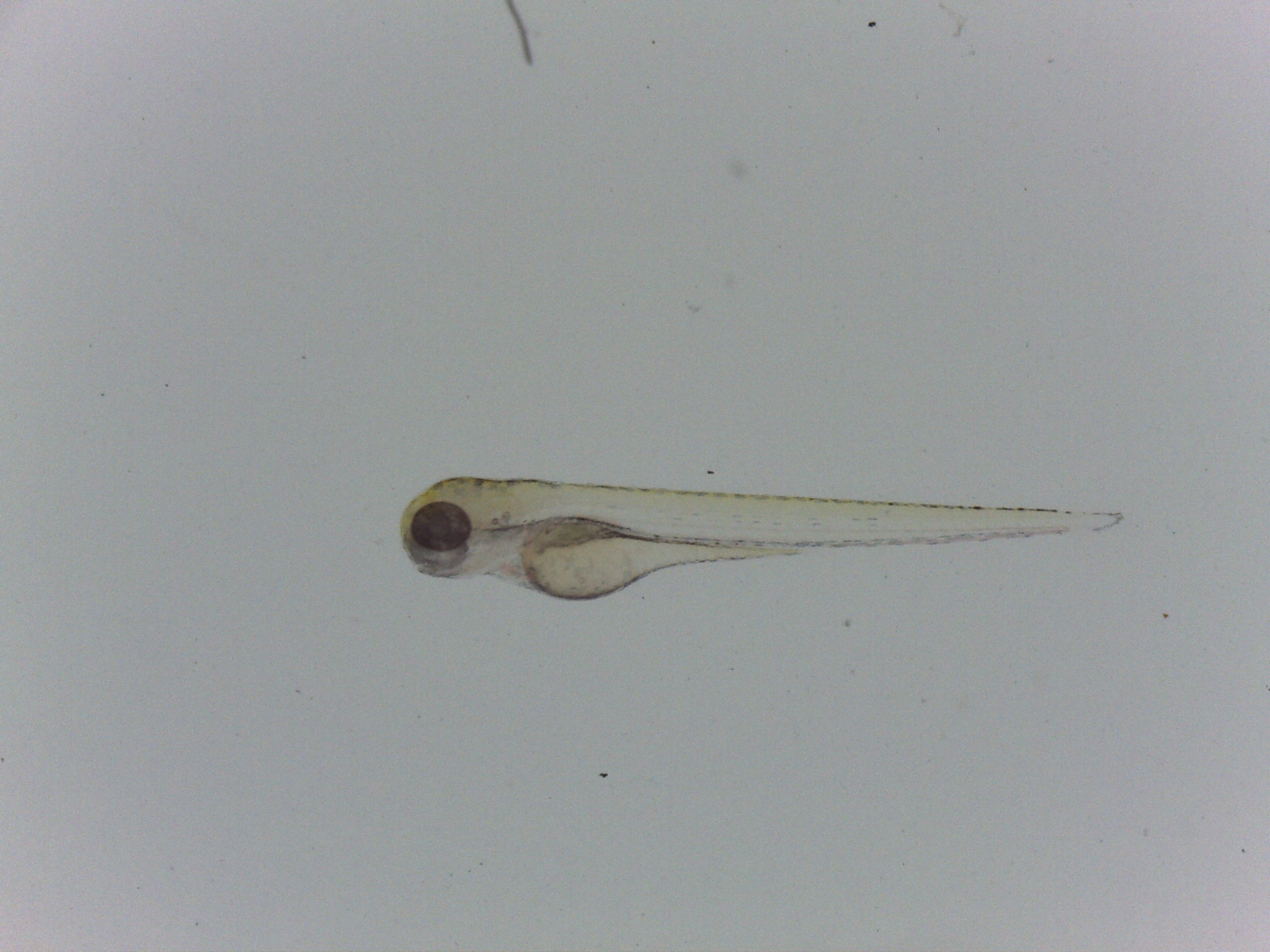

Supplement: Supplementary file 10 — Source data Fig. 5 [file 44318_2024_307_MOESM10_ESM.zip › EMBOJ-2024-116734_sourcedata_Fig 5/Fig5_panelA_MoCTL_morpho.tif]

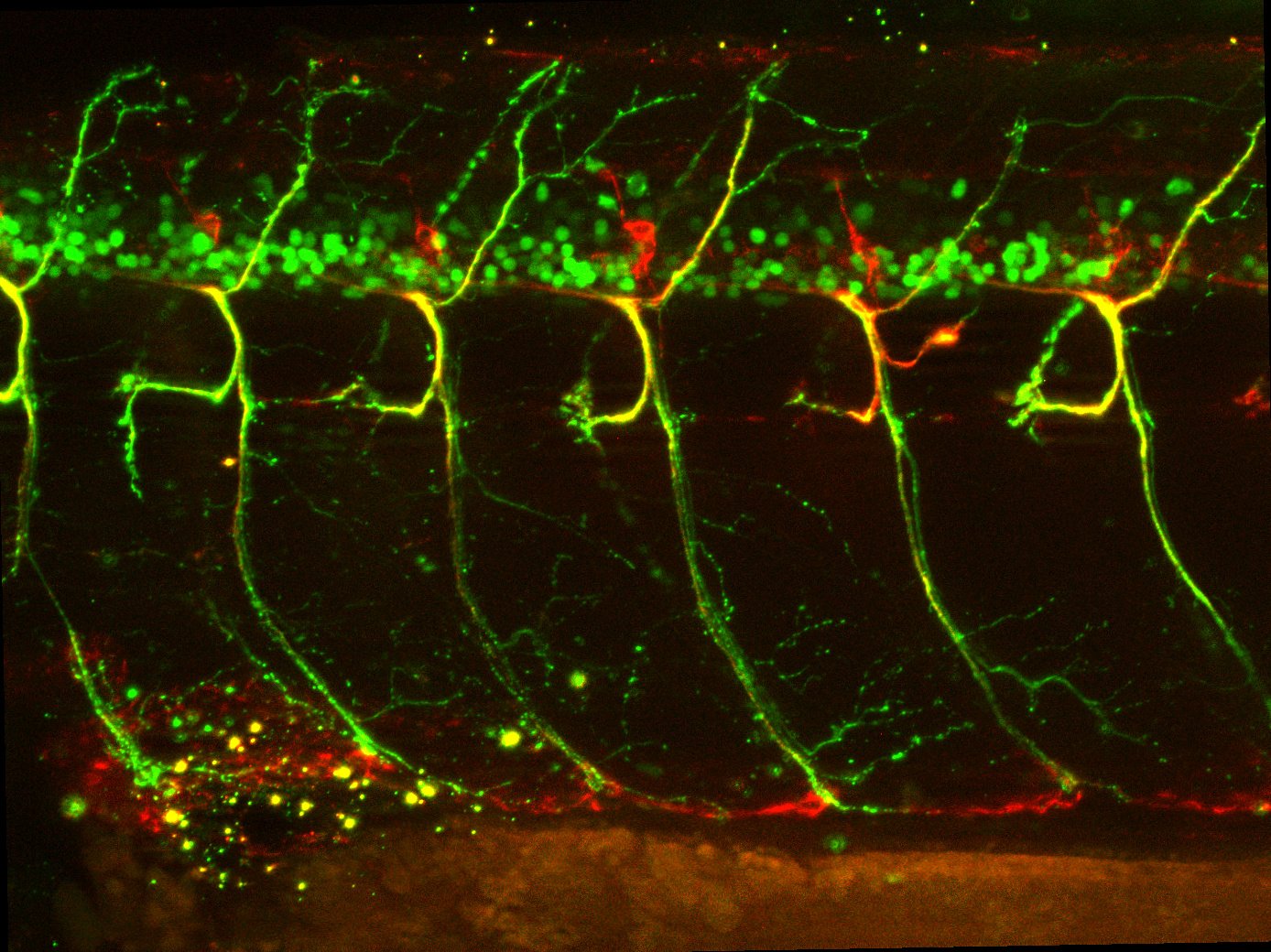

Supplement: Supplementary file 11 — Source data Fig. 6 [file 44318_2024_307_MOESM11_ESM.zip › EMBOJ-2024-116734_sourcedata_Fig 6/Fig 6_panelA_sMN_zn5GFP_MokatandTTLL6mRNA.tif]

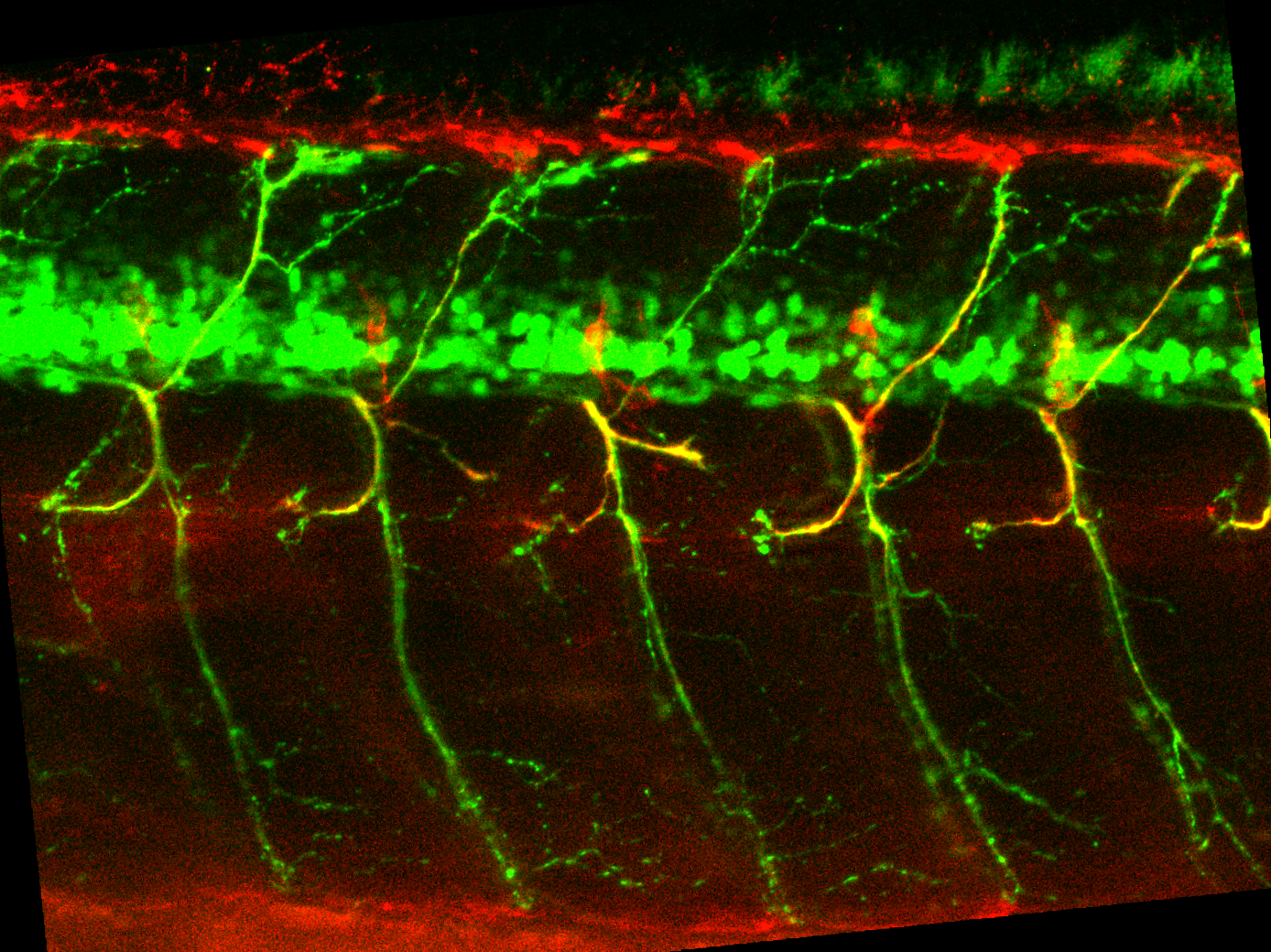

Supplement: Supplementary file 11 — Source data Fig. 6 [file 44318_2024_307_MOESM11_ESM.zip › EMBOJ-2024-116734_sourcedata_Fig 6/Fig 6_panelA_sMN_zn5GFP_MokatandTTLL11mRNA.tif]

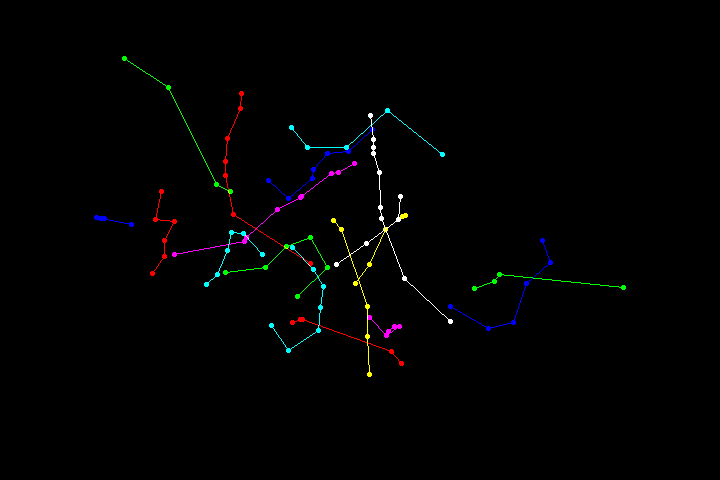

Supplement: Supplementary file 11 — Source data Fig. 6 [file 44318_2024_307_MOESM11_ESM.zip › EMBOJ-2024-116734_sourcedata_Fig 6/Fig 6_panellA_dots and lines Mokat.tif]

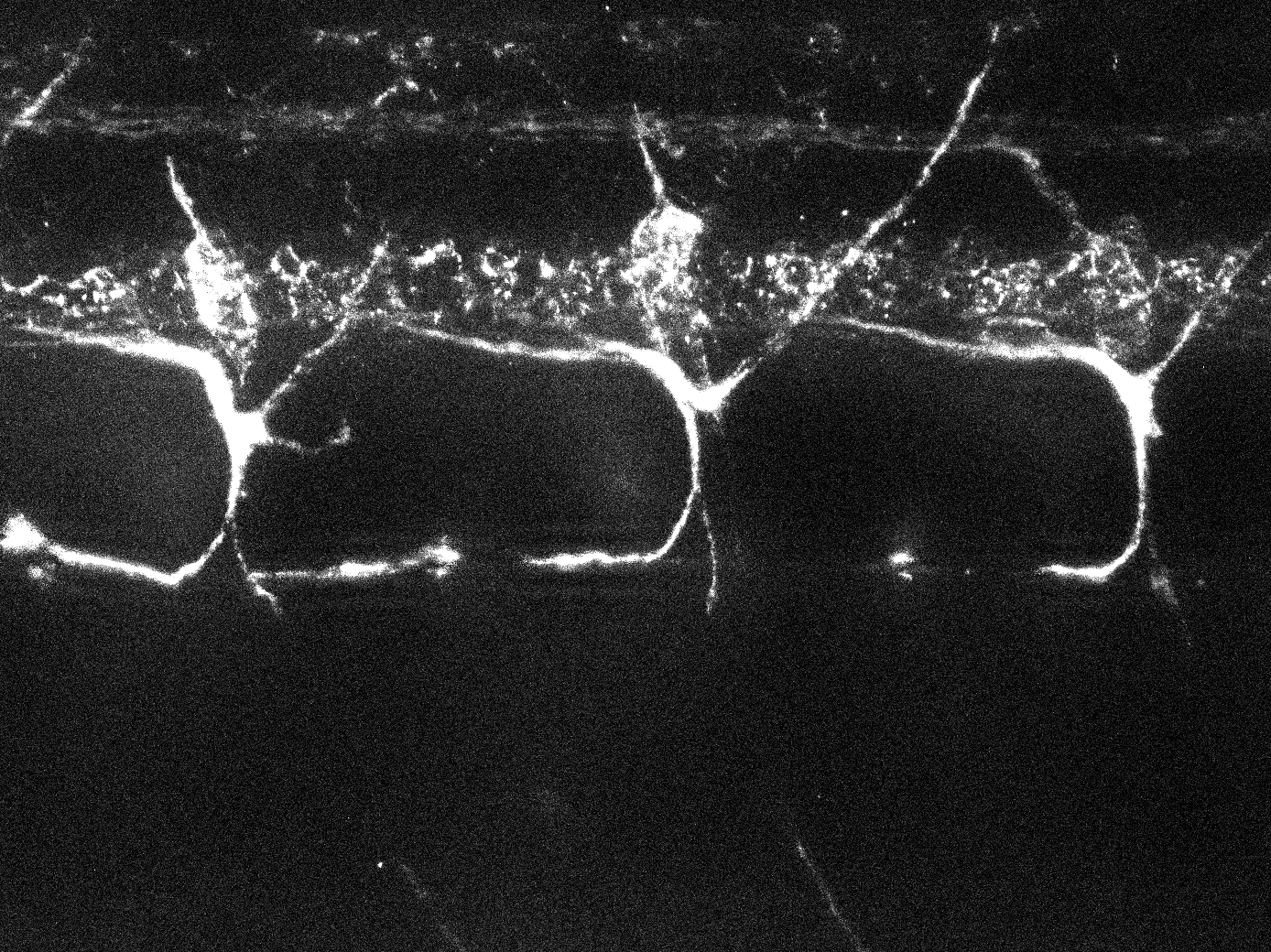

Supplement: Supplementary file 11 — Source data Fig. 6 [file 44318_2024_307_MOESM11_ESM.zip › EMBOJ-2024-116734_sourcedata_Fig 6/Fig 6_panelF_Katna1KOmz_sMN_zn5.tif]

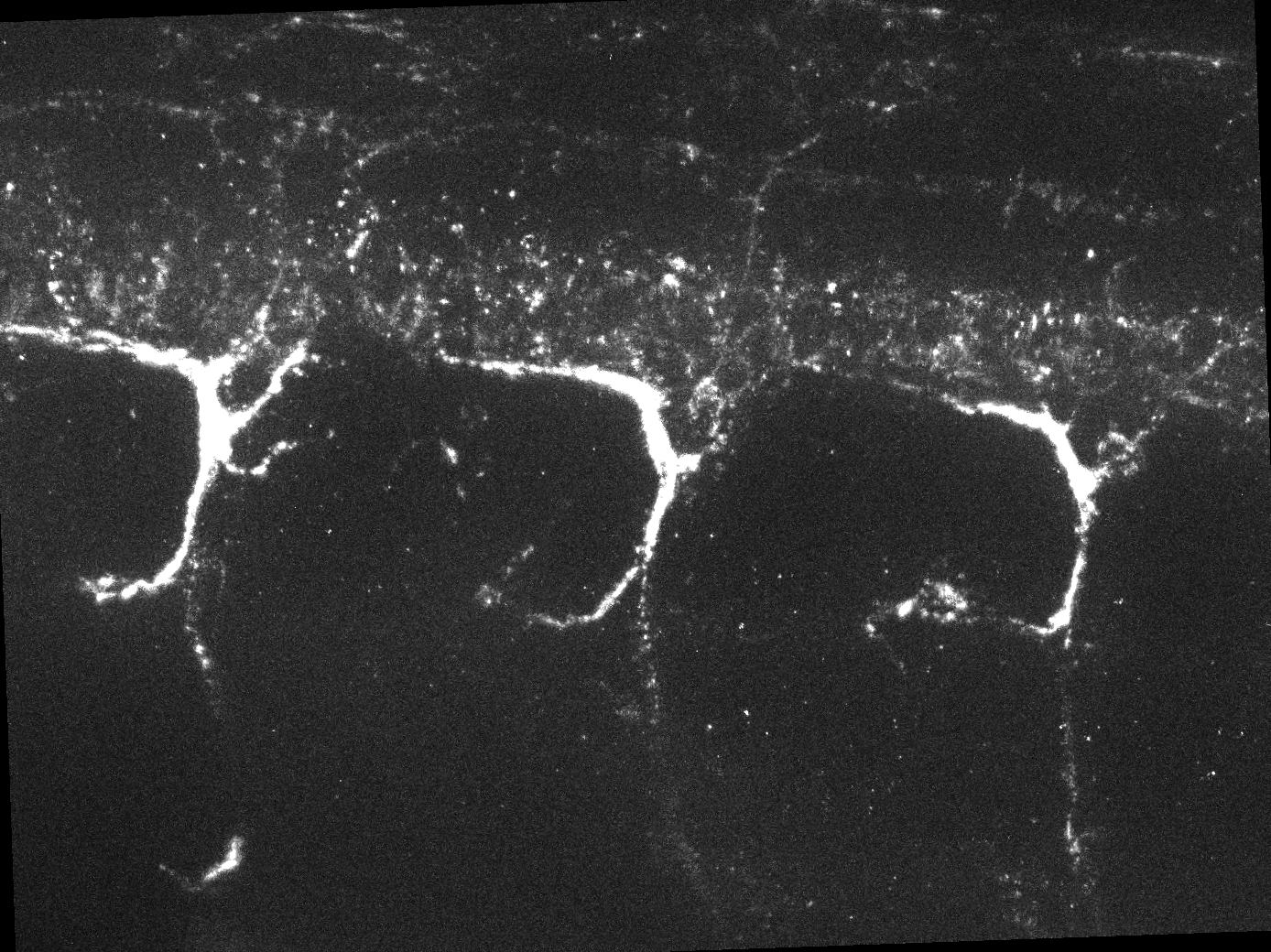

Supplement: Supplementary file 11 — Source data Fig. 6 [file 44318_2024_307_MOESM11_ESM.zip › EMBOJ-2024-116734_sourcedata_Fig 6/Fig 6_panelF_Katna1KOmzandTTLL6mRNA_sMN_zn5.tif]

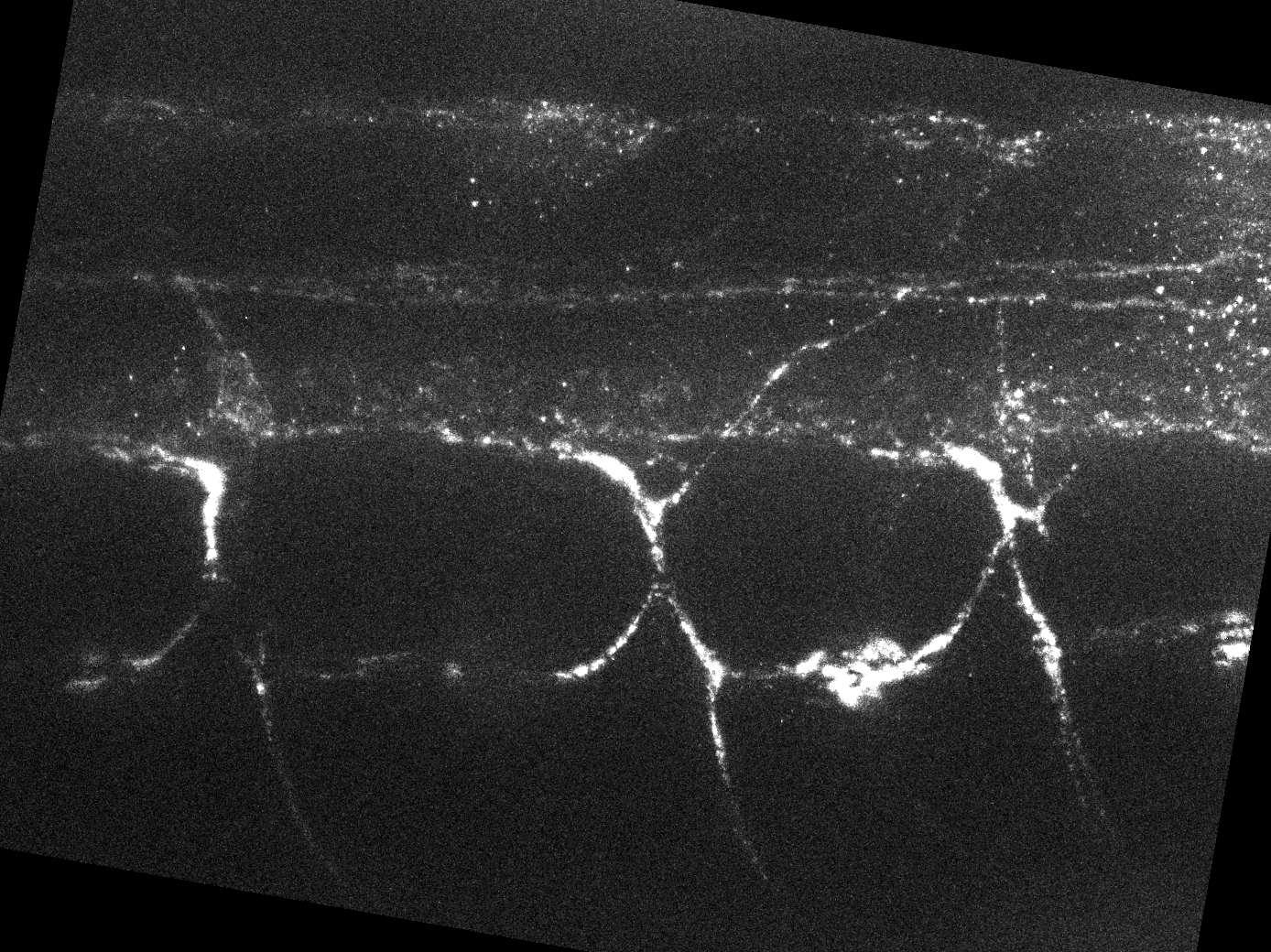

Supplement: Supplementary file 11 — Source data Fig. 6 [file 44318_2024_307_MOESM11_ESM.zip › EMBOJ-2024-116734_sourcedata_Fig 6/Fig 6_panelF_Katna1KOmzandTTLL11mRNA_sMN_zn5.tif]

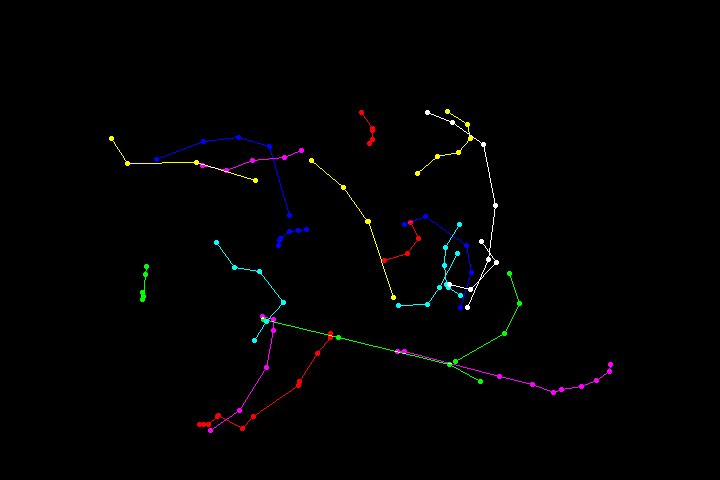

Supplement: Supplementary file 11 — Source data Fig. 6 [file 44318_2024_307_MOESM11_ESM.zip › EMBOJ-2024-116734_sourcedata_Fig 6/Fig 6_panellA_dots and lines MokatandTTLL11mRNA.tif]

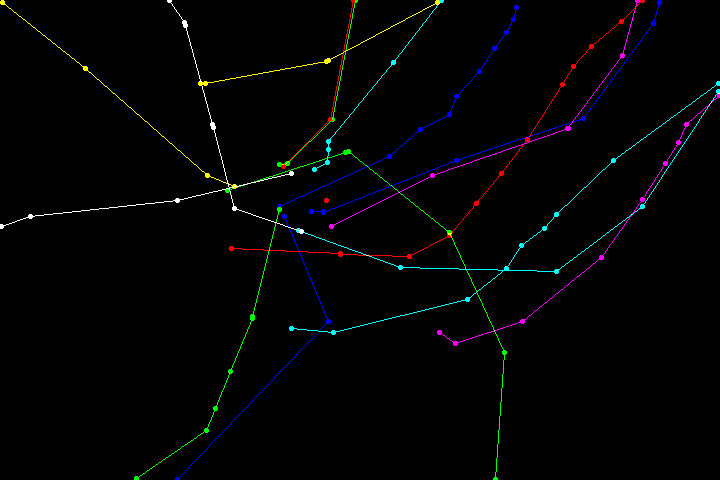

Supplement: Supplementary file 11 — Source data Fig. 6 [file 44318_2024_307_MOESM11_ESM.zip › EMBOJ-2024-116734_sourcedata_Fig 6/Fig 6_panelA_dots and lines MoCtl.tif]

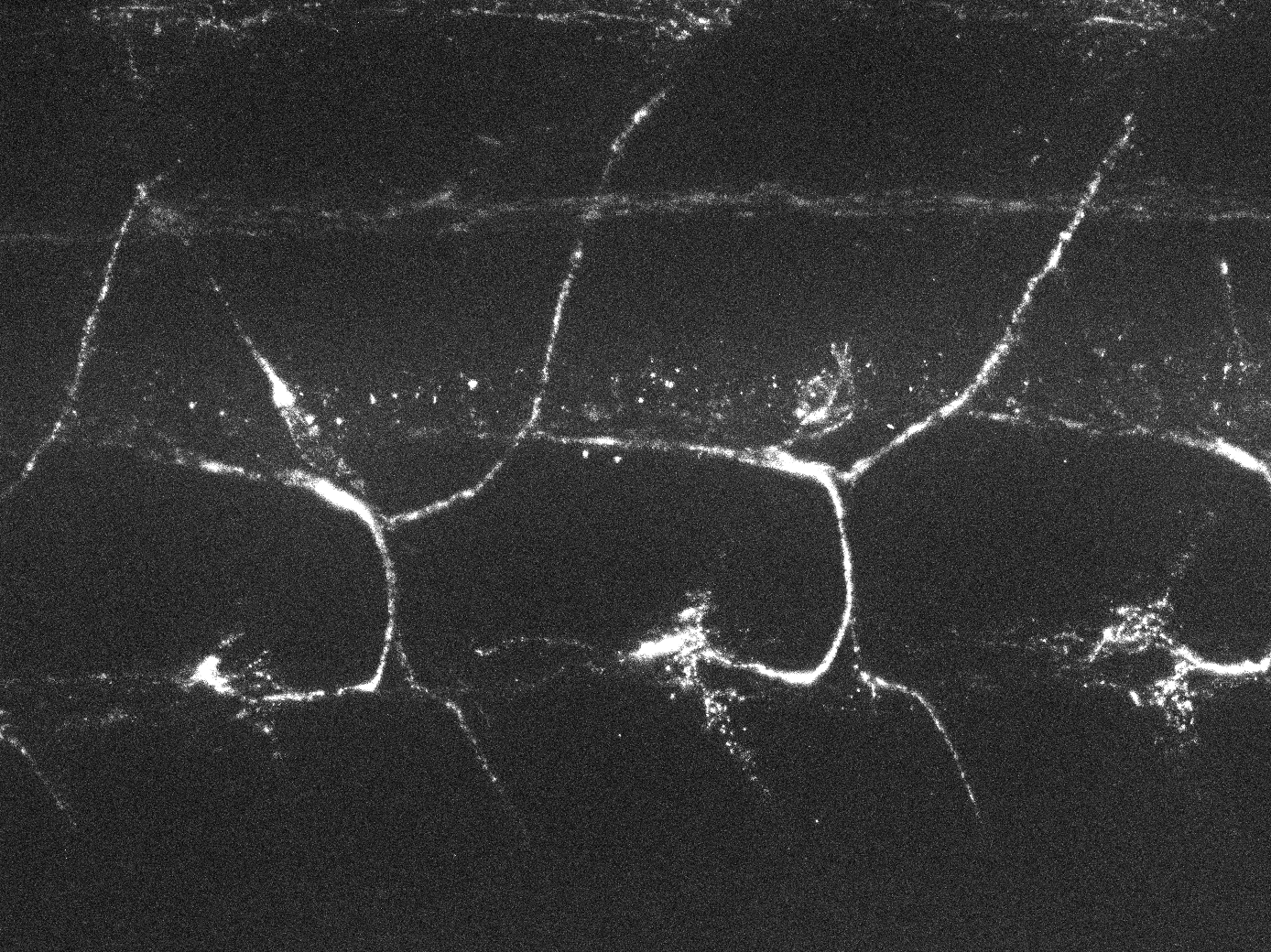

Supplement: Supplementary file 11 — Source data Fig. 6 [file 44318_2024_307_MOESM11_ESM.zip › EMBOJ-2024-116734_sourcedata_Fig 6/Fig 6_panelF_Katna1WT_sMN_zn5.tif]

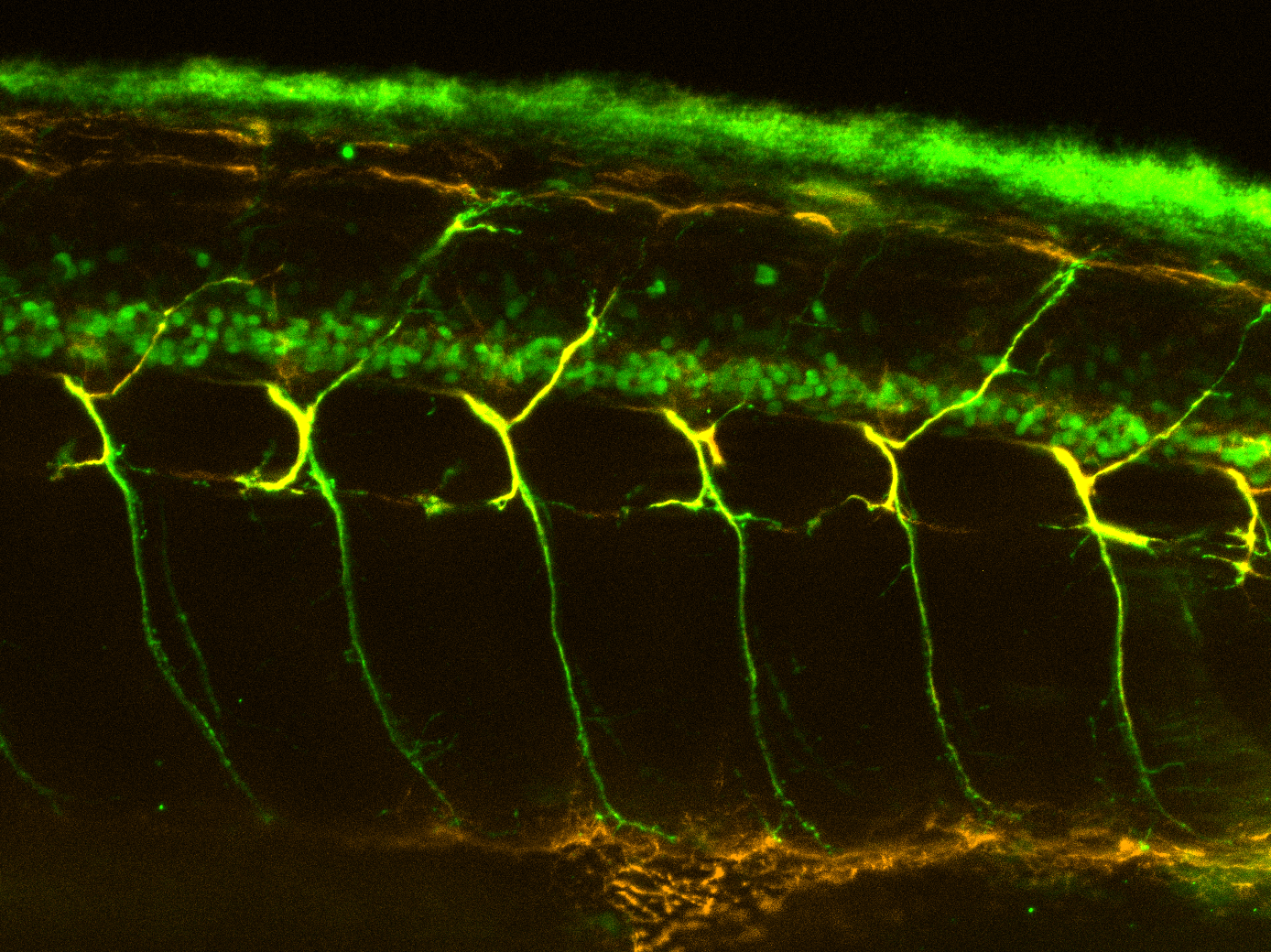

Supplement: Supplementary file 11 — Source data Fig. 6 [file 44318_2024_307_MOESM11_ESM.zip › EMBOJ-2024-116734_sourcedata_Fig 6/Fig 6_panelA_sMN_zn5GFP_MokatandTTLL11mRNA_caudal projection.tif]

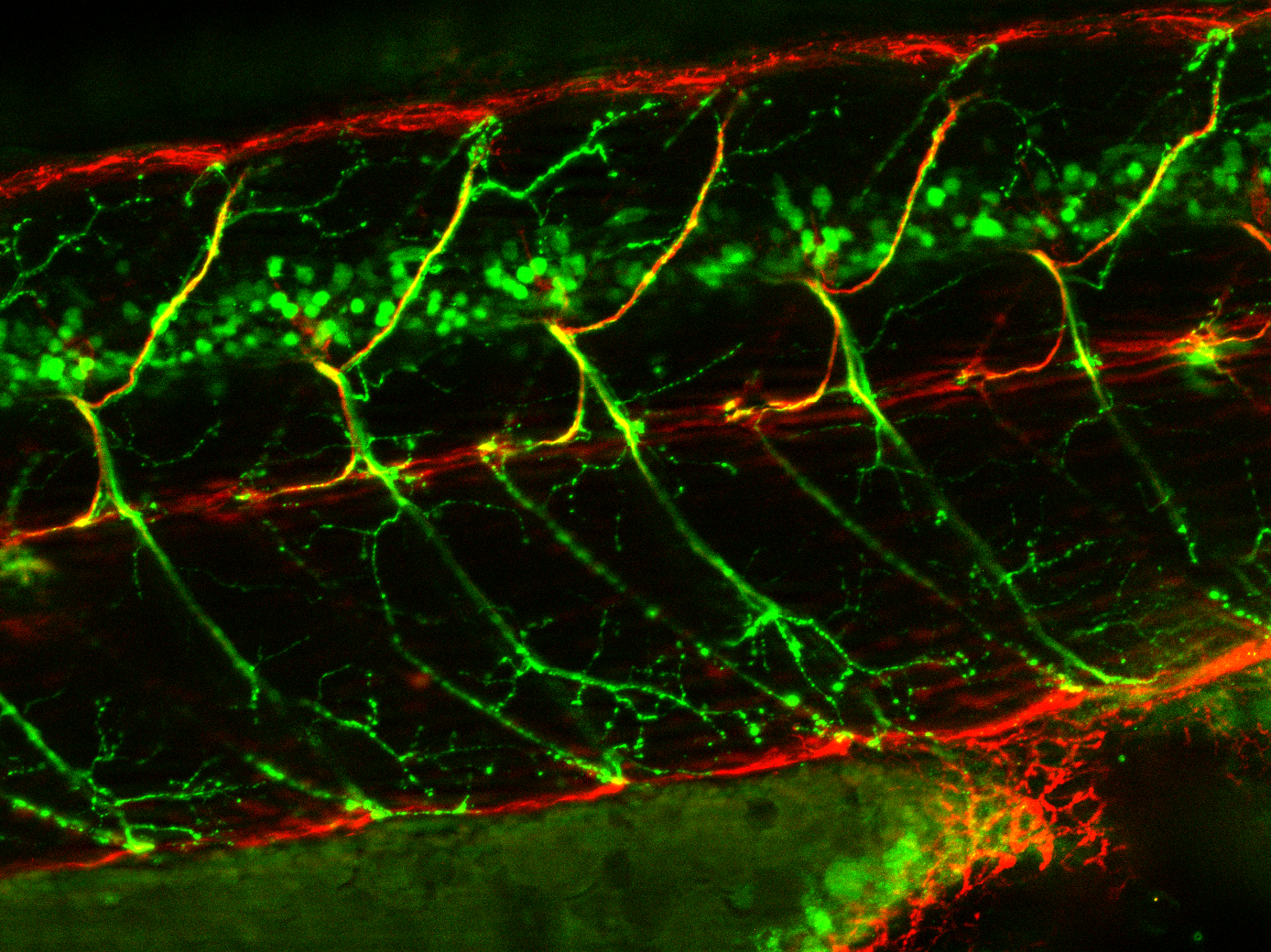

Supplement: Supplementary file 11 — Source data Fig. 6 [file 44318_2024_307_MOESM11_ESM.zip › EMBOJ-2024-116734_sourcedata_Fig 6/Fig 6_panelA_sMN_zn5GFP_MoCtl.tif]

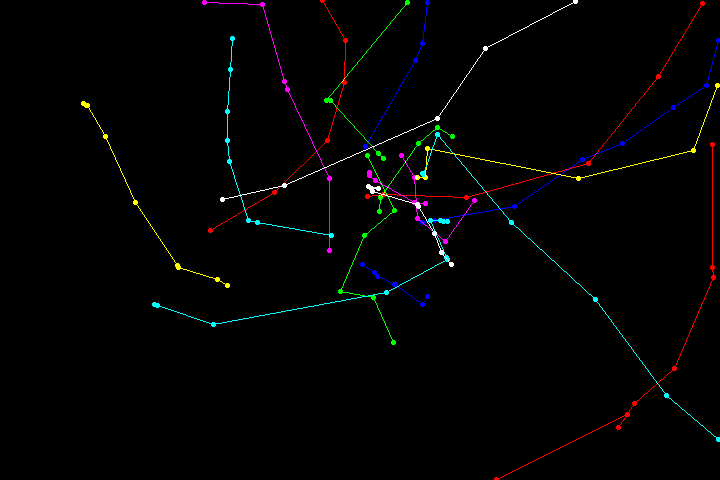

Supplement: Supplementary file 11 — Source data Fig. 6 [file 44318_2024_307_MOESM11_ESM.zip › EMBOJ-2024-116734_sourcedata_Fig 6/Fig 6_panellA_dots and lines MokatandTTLL6mRNA.tif]

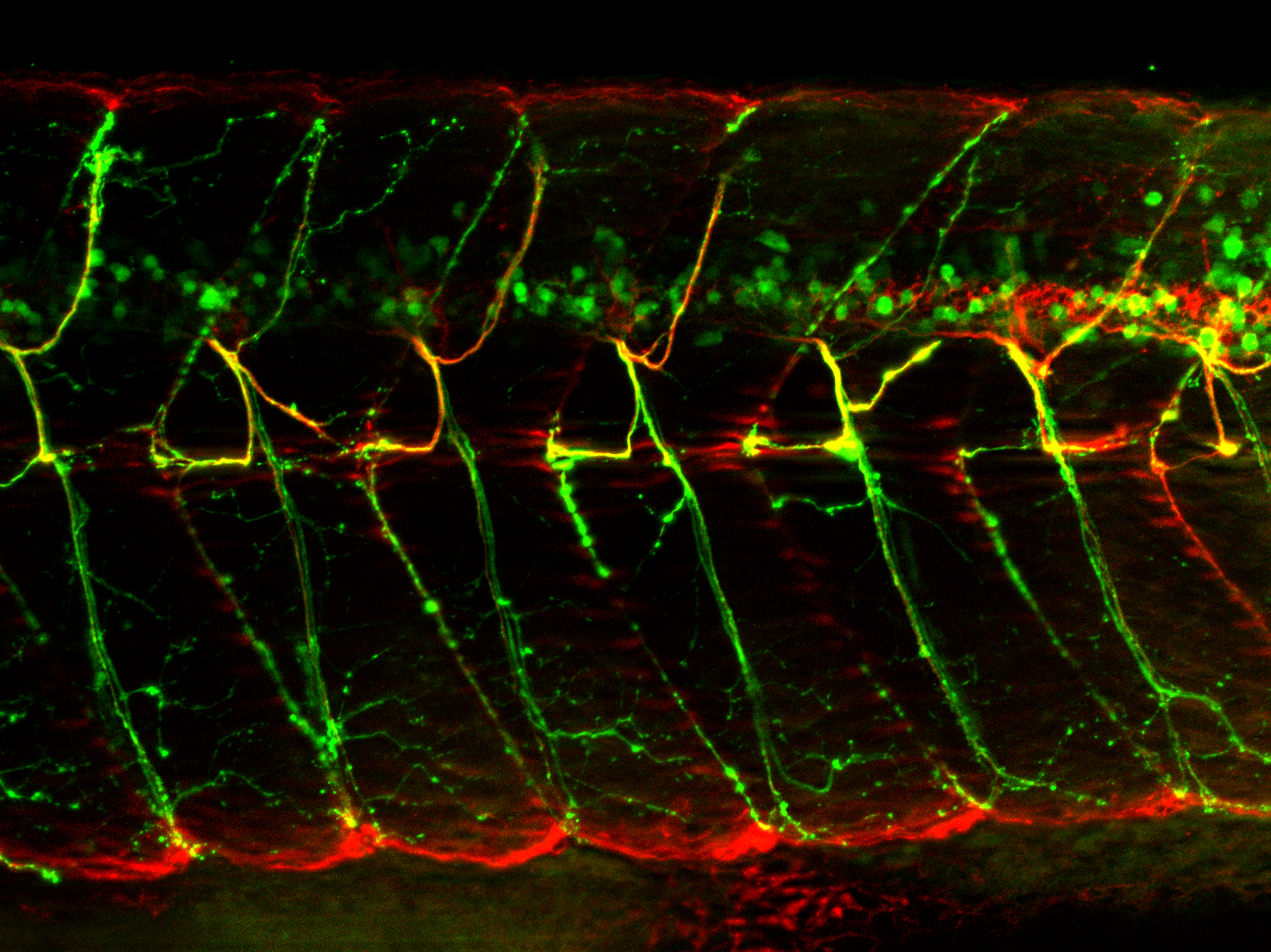

Supplement: Supplementary file 11 — Source data Fig. 6 [file 44318_2024_307_MOESM11_ESM.zip › EMBOJ-2024-116734_sourcedata_Fig 6/Fig 6_panelA_sMN_zn5GFP_MoKat.tif]

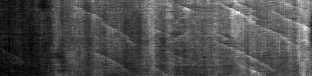

Supplement: Supplementary file 12 — Source data Fig. 7 [file 44318_2024_307_MOESM12_ESM.zip › EMBOJ-2024-116734_sourcedata_Fig 7/Fig7_panelA_MOKat1.3_EB3-GFP_Kymo.tif]

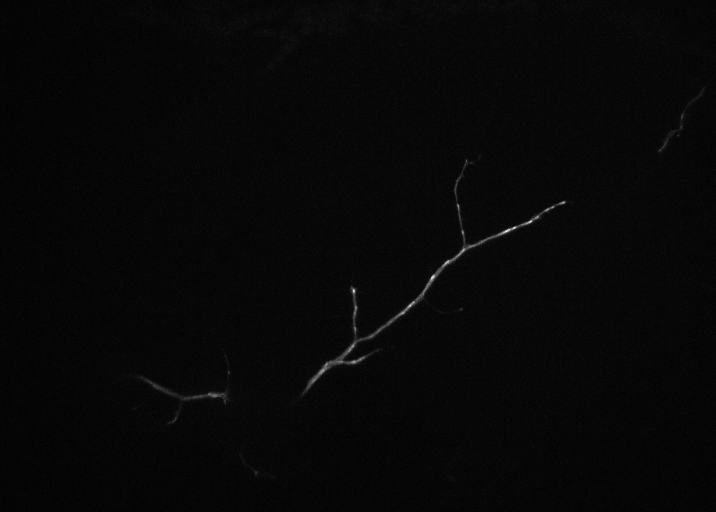

Supplement: Supplementary file 12 — Source data Fig. 7 [file 44318_2024_307_MOESM12_ESM.zip › EMBOJ-2024-116734_sourcedata_Fig 7/Fig7_panelA_MOCTL_EB3-GFP.tif]

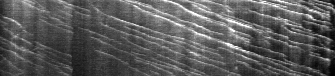

Supplement: Supplementary file 12 — Source data Fig. 7 [file 44318_2024_307_MOESM12_ESM.zip › EMBOJ-2024-116734_sourcedata_Fig 7/Fig7_panelA_MOCTL_EB3-GFP_Kymo.tif]

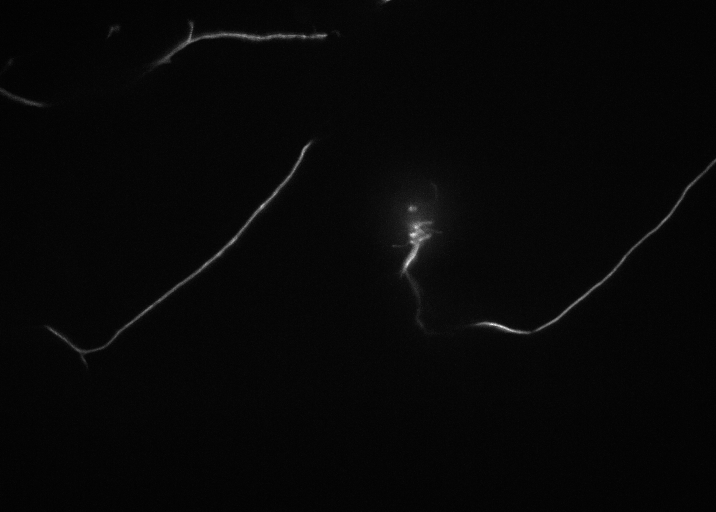

Supplement: Supplementary file 12 — Source data Fig. 7 [file 44318_2024_307_MOESM12_ESM.zip › EMBOJ-2024-116734_sourcedata_Fig 7/Fig7_panelA_MOKat1.3_EB3-GFP.tif]

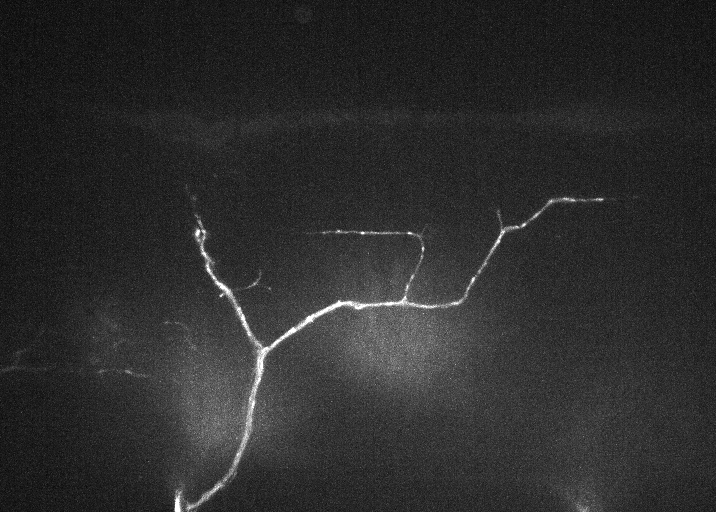

Supplement: Supplementary file 12 — Source data Fig. 7 [file 44318_2024_307_MOESM12_ESM.zip › EMBOJ-2024-116734_sourcedata_Fig 7/Fig7_panelA_MOKat1.3andTTLL6mRNA_EB3-GFPzoom.tif]

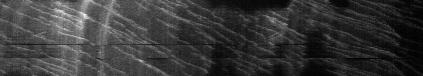

Supplement: Supplementary file 12 — Source data Fig. 7 [file 44318_2024_307_MOESM12_ESM.zip › EMBOJ-2024-116734_sourcedata_Fig 7/Fig7_panelA_MOKat1.3andTTLL6mRNA_EB3-GFP_Kymo.tif]

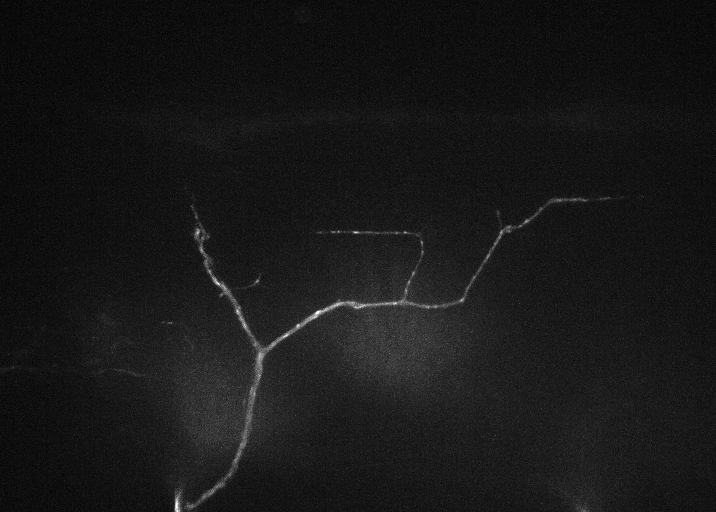

Supplement: Supplementary file 12 — Source data Fig. 7 [file 44318_2024_307_MOESM12_ESM.zip › EMBOJ-2024-116734_sourcedata_Fig 7/Fig7_panelA_MOKat1.3andTTLL6mRNA_EB3-GFP.tif]

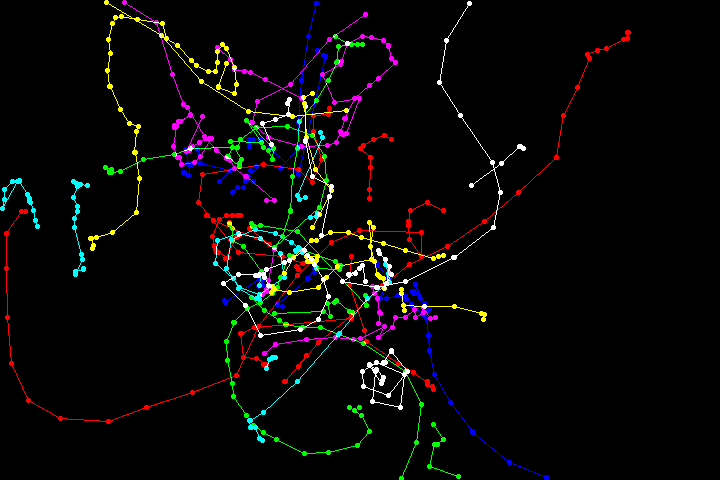

Supplement: Supplementary file 13 — Source data Fig. 8 [file 44318_2024_307_MOESM13_ESM.zip › EMBOJ-2024-116734_sourcedata_Fig 8/Fig8_panelA_MOspATG and TTLL6mRNA_dotsandlines.tif]

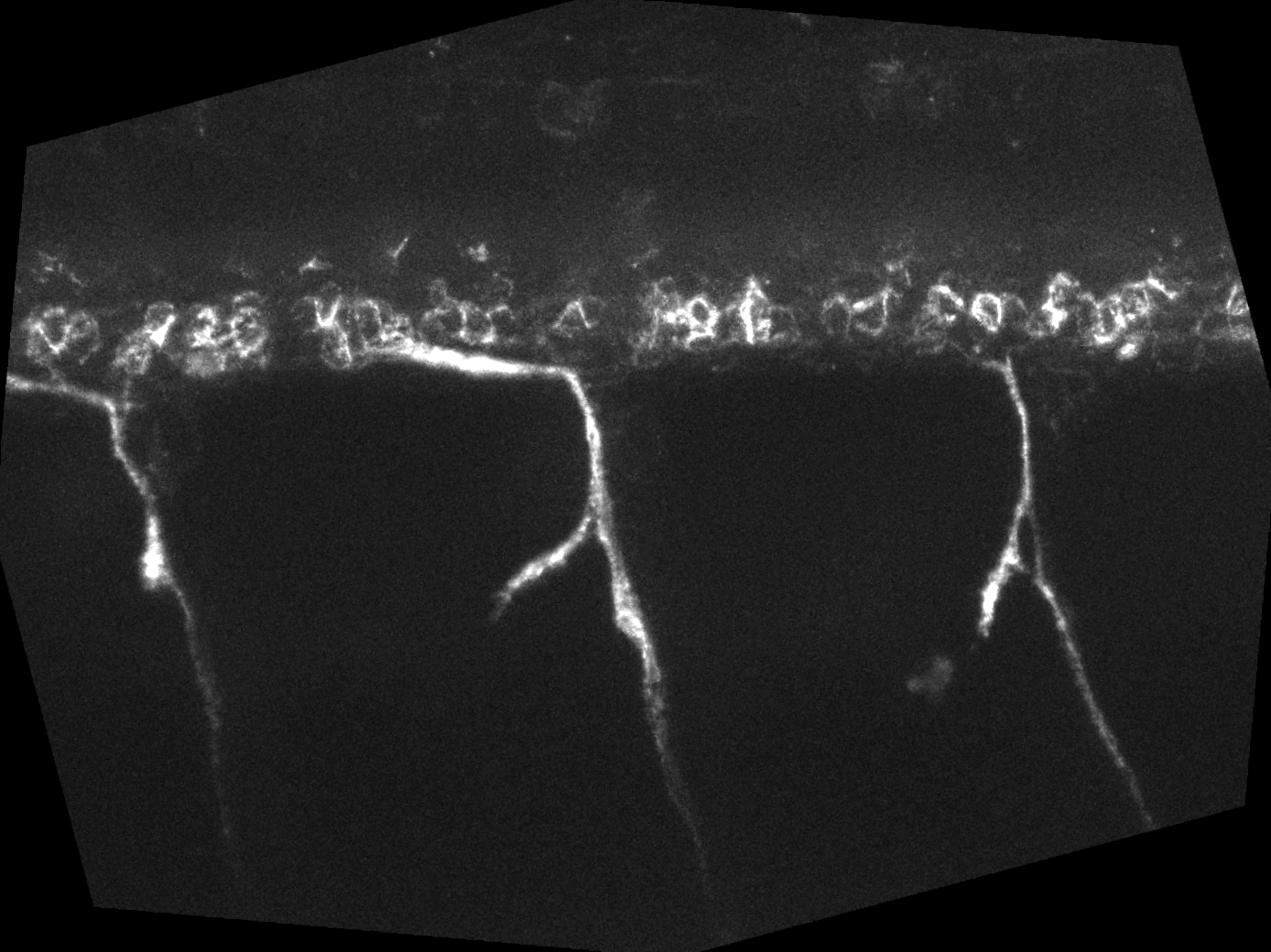

Supplement: Supplementary file 13 — Source data Fig. 8 [file 44318_2024_307_MOESM13_ESM.zip › EMBOJ-2024-116734_sourcedata_Fig 8/Fig8_panelA_MOspATG and TTLL6mRNA_sMN_zn5.tif]

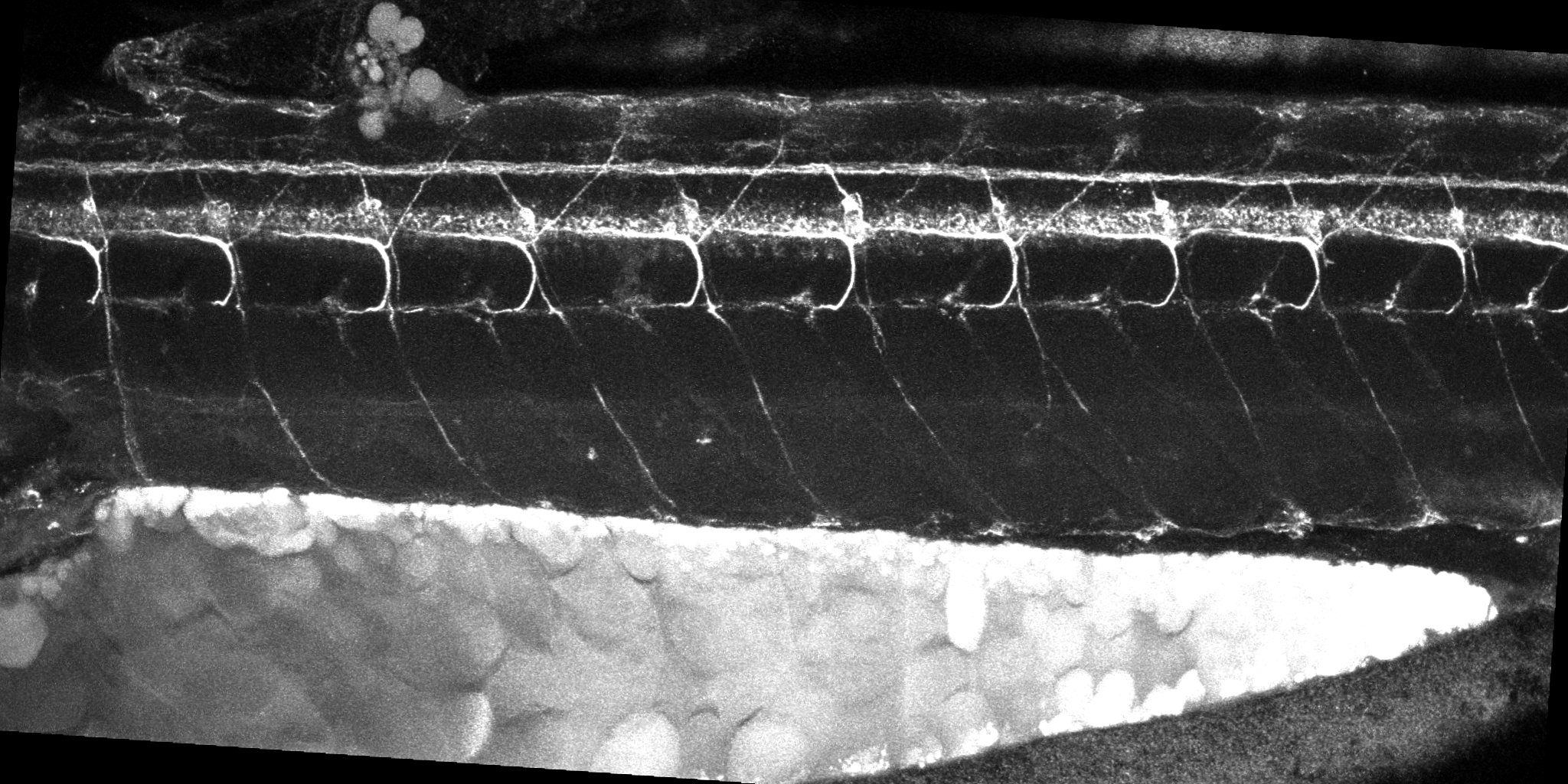

Supplement: Supplementary file 13 — Source data Fig. 8 [file 44318_2024_307_MOESM13_ESM.zip › EMBOJ-2024-116734_sourcedata_Fig 8/Fig8_panelE_sp++_sMN_zn5.tif]

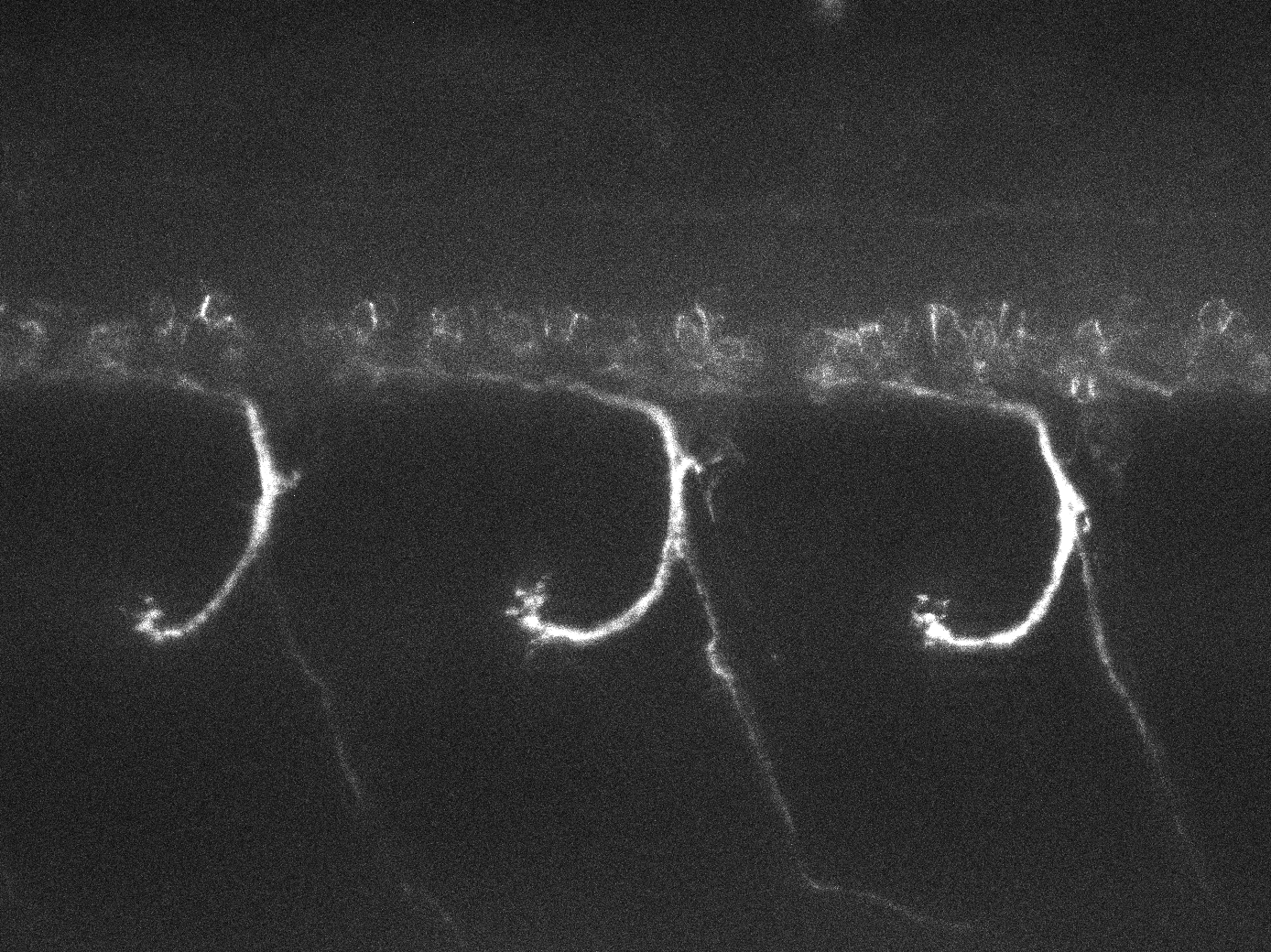

Supplement: Supplementary file 13 — Source data Fig. 8 [file 44318_2024_307_MOESM13_ESM.zip › EMBOJ-2024-116734_sourcedata_Fig 8/Fig8_panelA_MOspATG and TTLL11mRNA_sMN_zn5.tif]

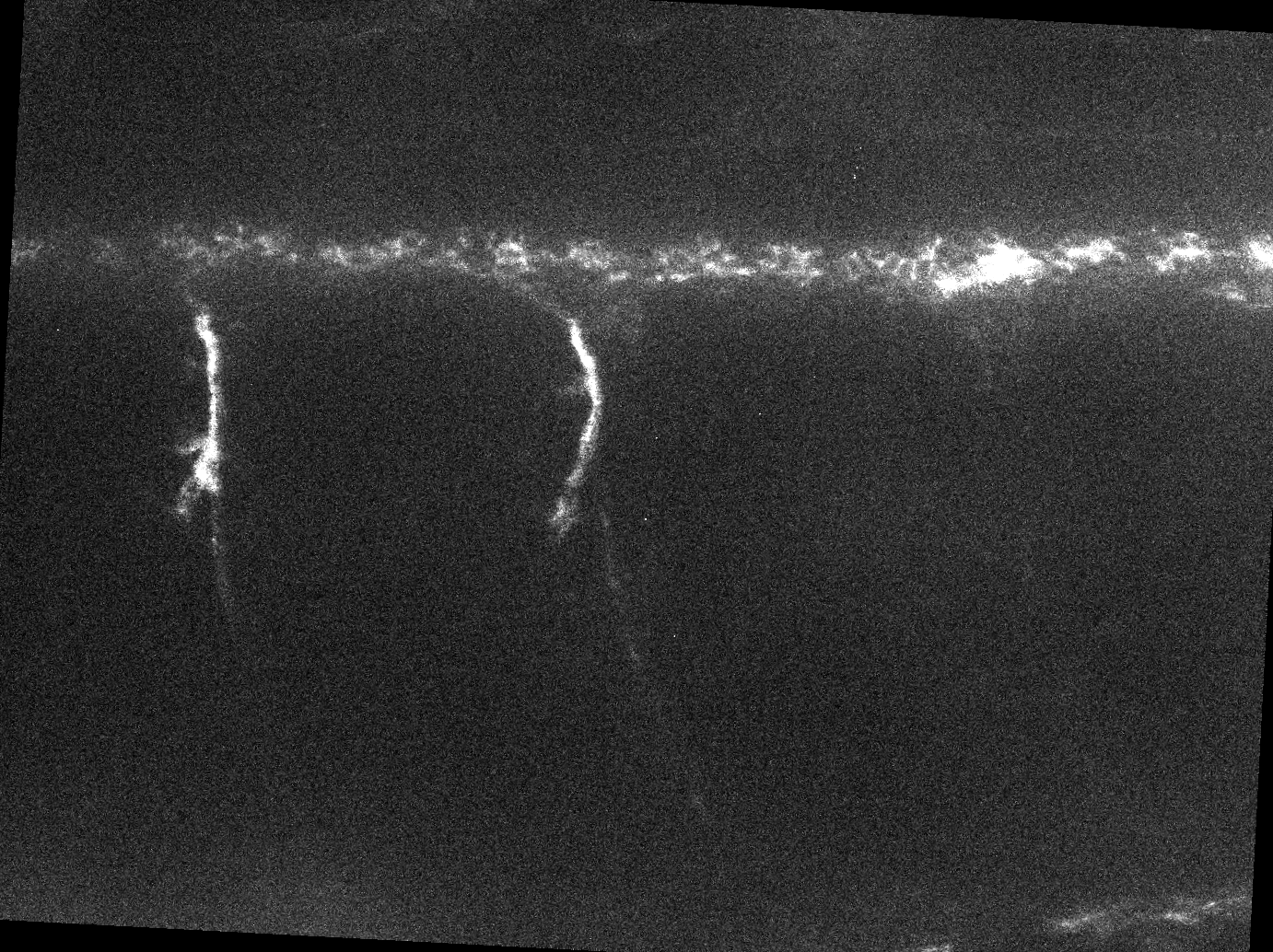

Supplement: Supplementary file 13 — Source data Fig. 8 [file 44318_2024_307_MOESM13_ESM.zip › EMBOJ-2024-116734_sourcedata_Fig 8/Fig8_panelA_MOspATG_sMN_zn5.tif]

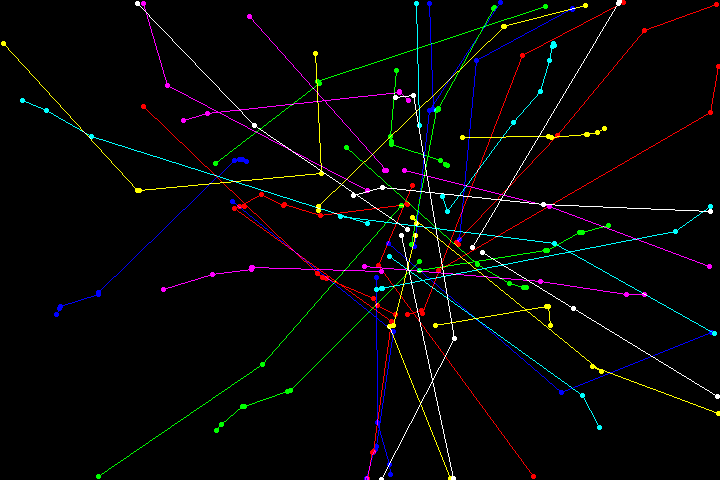

Supplement: Supplementary file 13 — Source data Fig. 8 [file 44318_2024_307_MOESM13_ESM.zip › EMBOJ-2024-116734_sourcedata_Fig 8/Fig8_panelA_MOCTL_dotsandlines.tif]

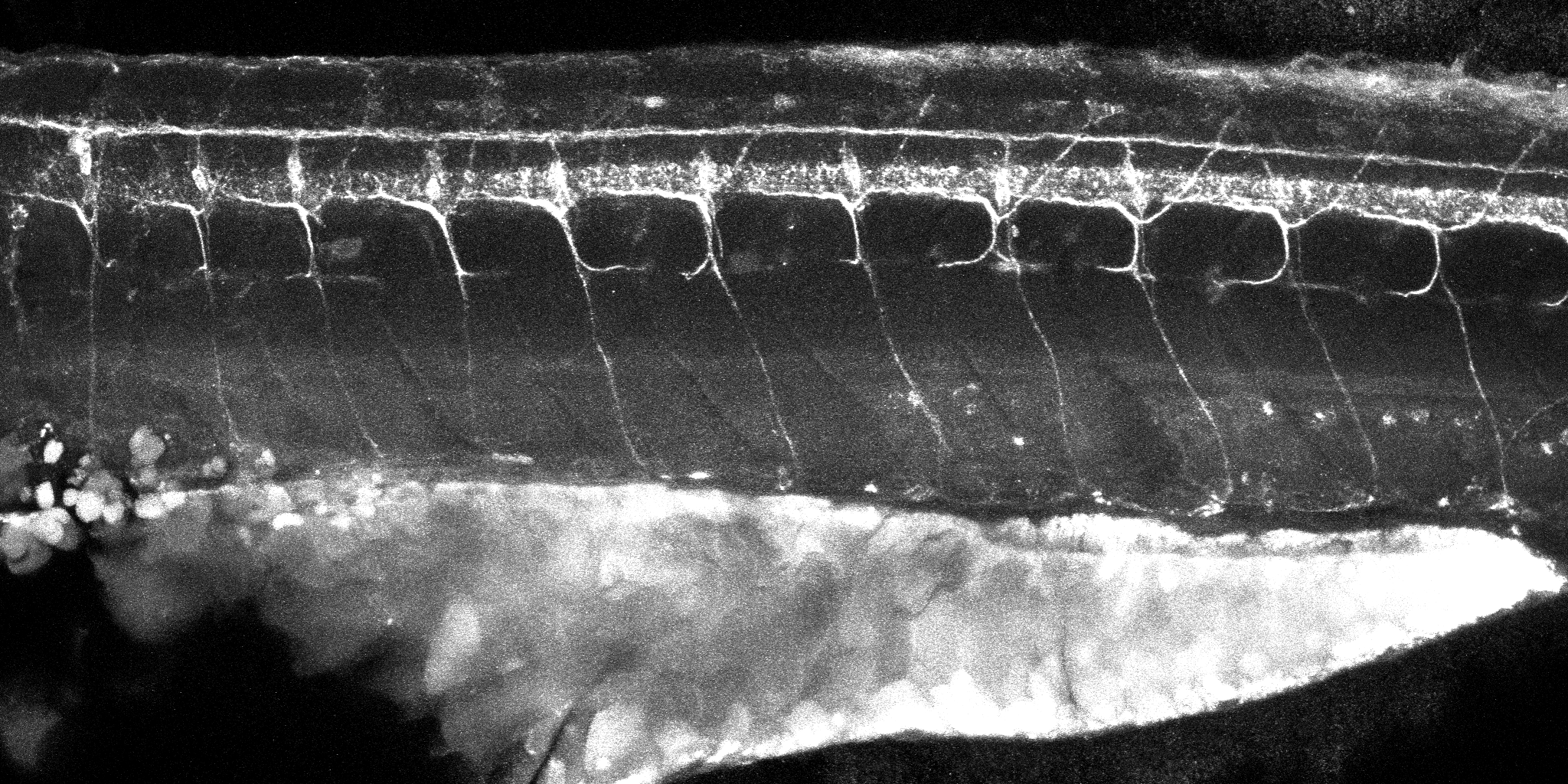

Supplement: Supplementary file 13 — Source data Fig. 8 [file 44318_2024_307_MOESM13_ESM.zip › EMBOJ-2024-116734_sourcedata_Fig 8/Fig8_panelE_spC68XC68Xand TTLL6mRNA_sMN_zn5.tif]

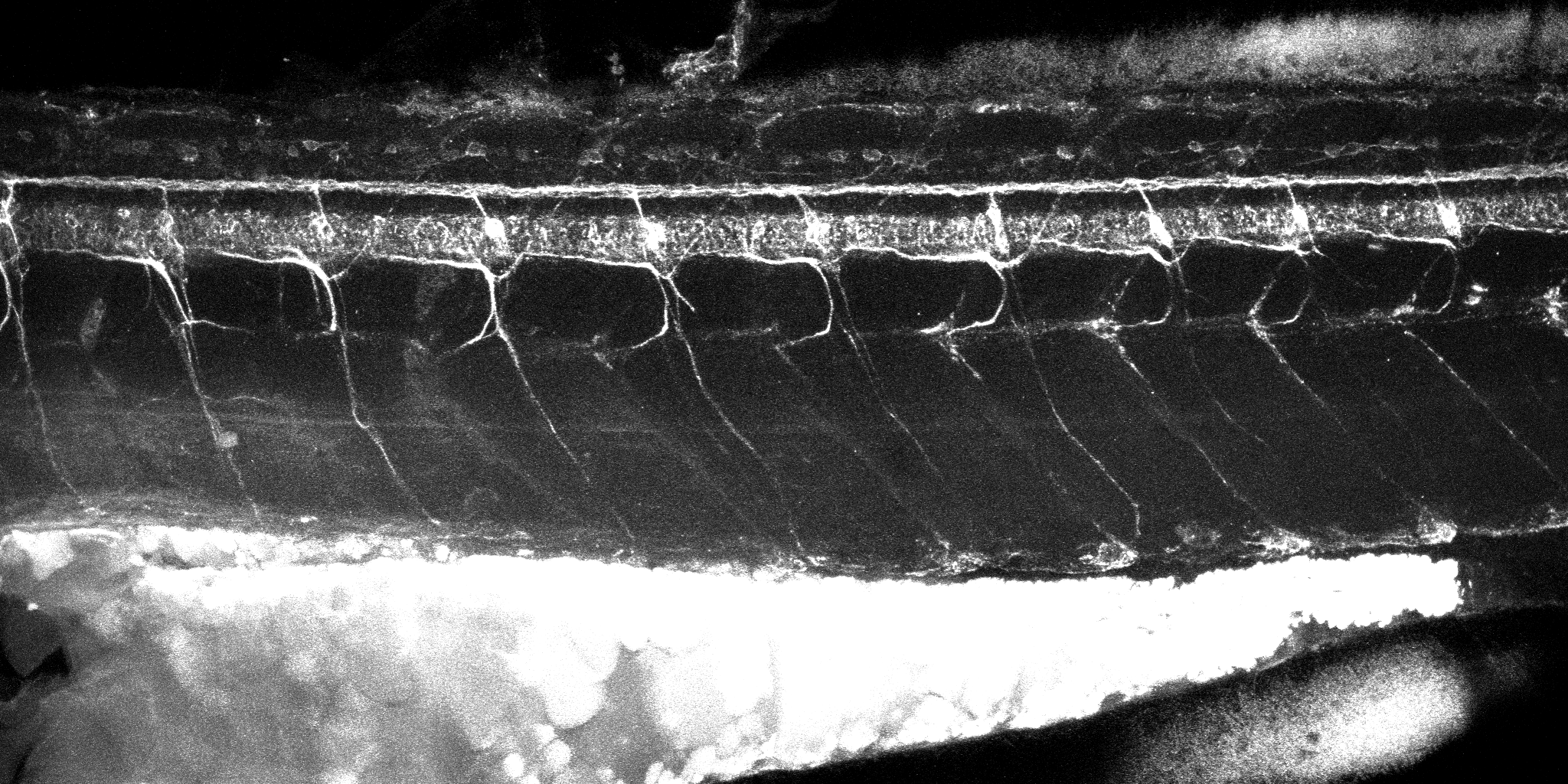

Supplement: Supplementary file 13 — Source data Fig. 8 [file 44318_2024_307_MOESM13_ESM.zip › EMBOJ-2024-116734_sourcedata_Fig 8/Fig8_panelE_spC68XC68X_sMN_zn5.tif]

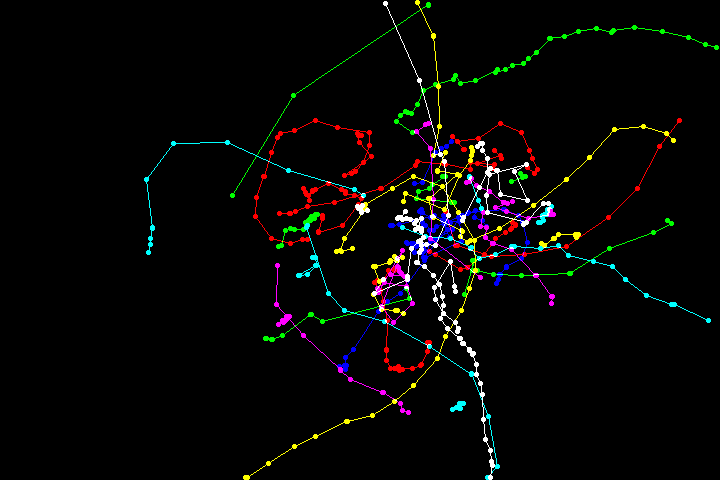

Supplement: Supplementary file 13 — Source data Fig. 8 [file 44318_2024_307_MOESM13_ESM.zip › EMBOJ-2024-116734_sourcedata_Fig 8/Fig8_panelA_MOspATG_dotsandlines.tif]

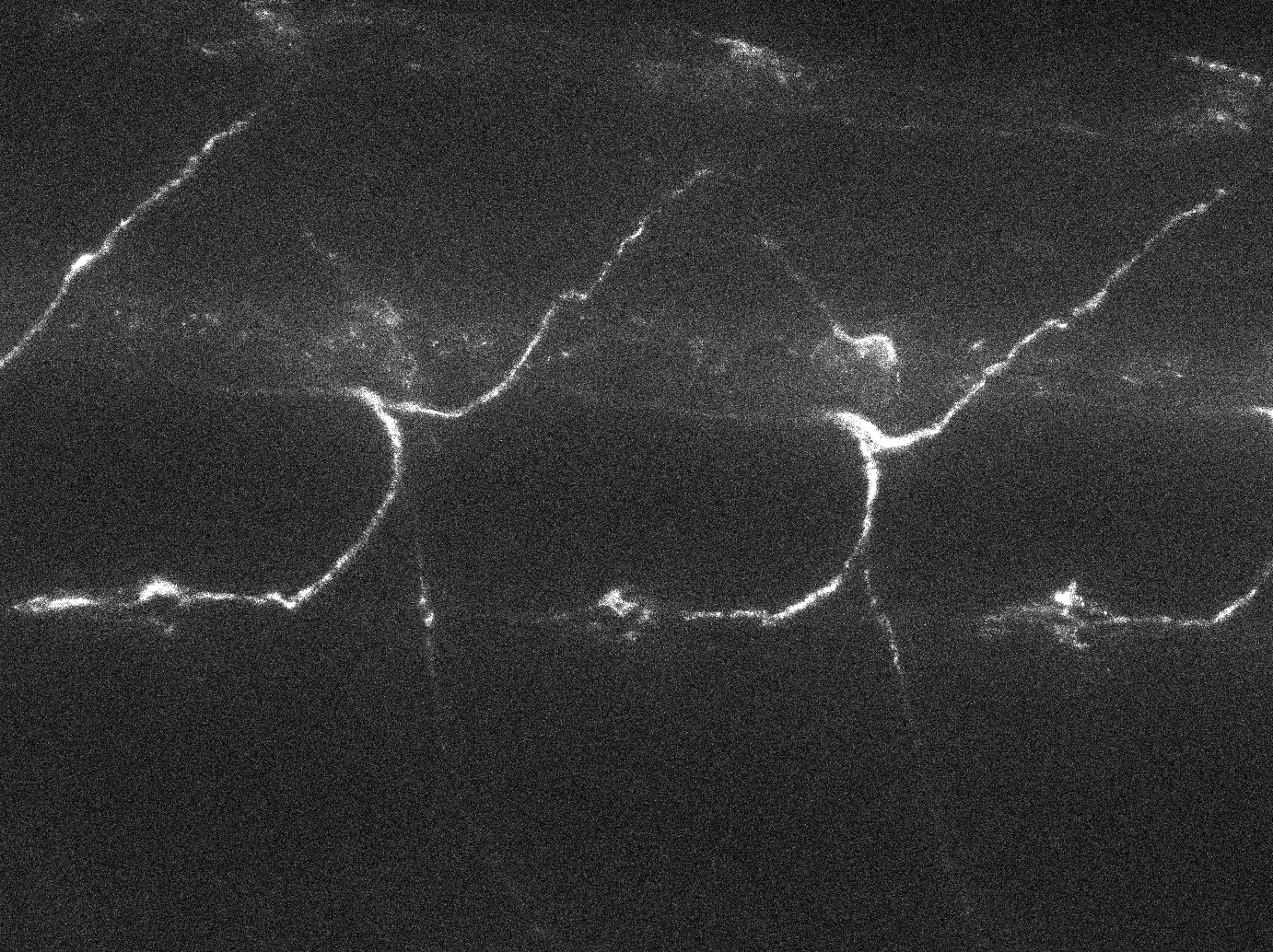

Supplement: Supplementary file 13 — Source data Fig. 8 [file 44318_2024_307_MOESM13_ESM.zip › EMBOJ-2024-116734_sourcedata_Fig 8/Fig8_panelA_MOCTL_sMN_zn5.tif]

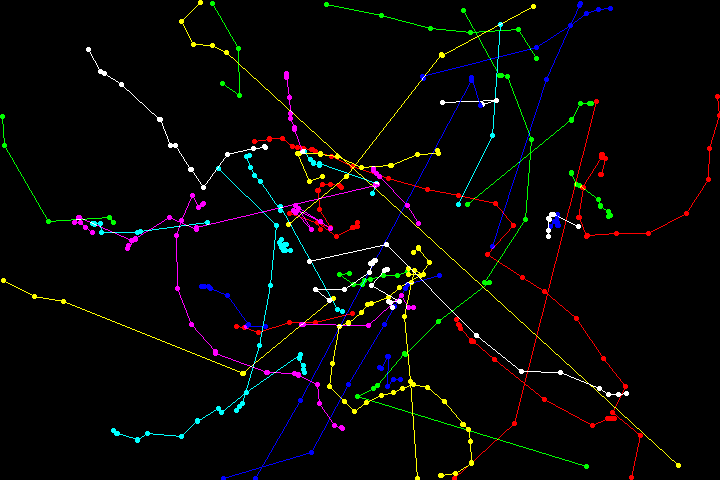

Supplement: Supplementary file 13 — Source data Fig. 8 [file 44318_2024_307_MOESM13_ESM.zip › EMBOJ-2024-116734_sourcedata_Fig 8/Fig8_panelA_MOspATG and TTLL11mRNA_dotsandlines.tif]

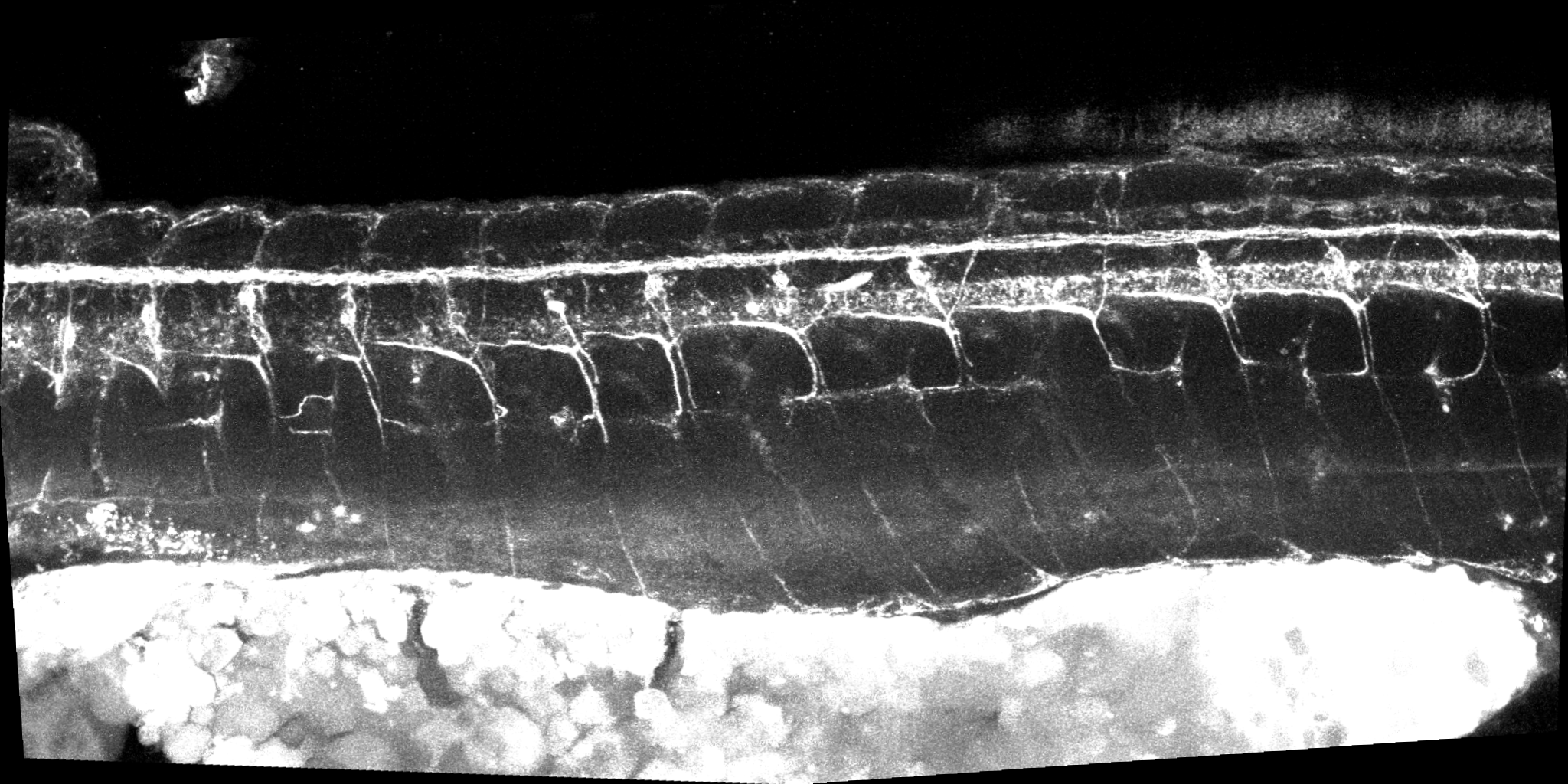

Supplement: Supplementary file 13 — Source data Fig. 8 [file 44318_2024_307_MOESM13_ESM.zip › EMBOJ-2024-116734_sourcedata_Fig 8/Fig8_panelE_spC68XC68Xand TTLL11mRNA_sMN_zn5.tif]

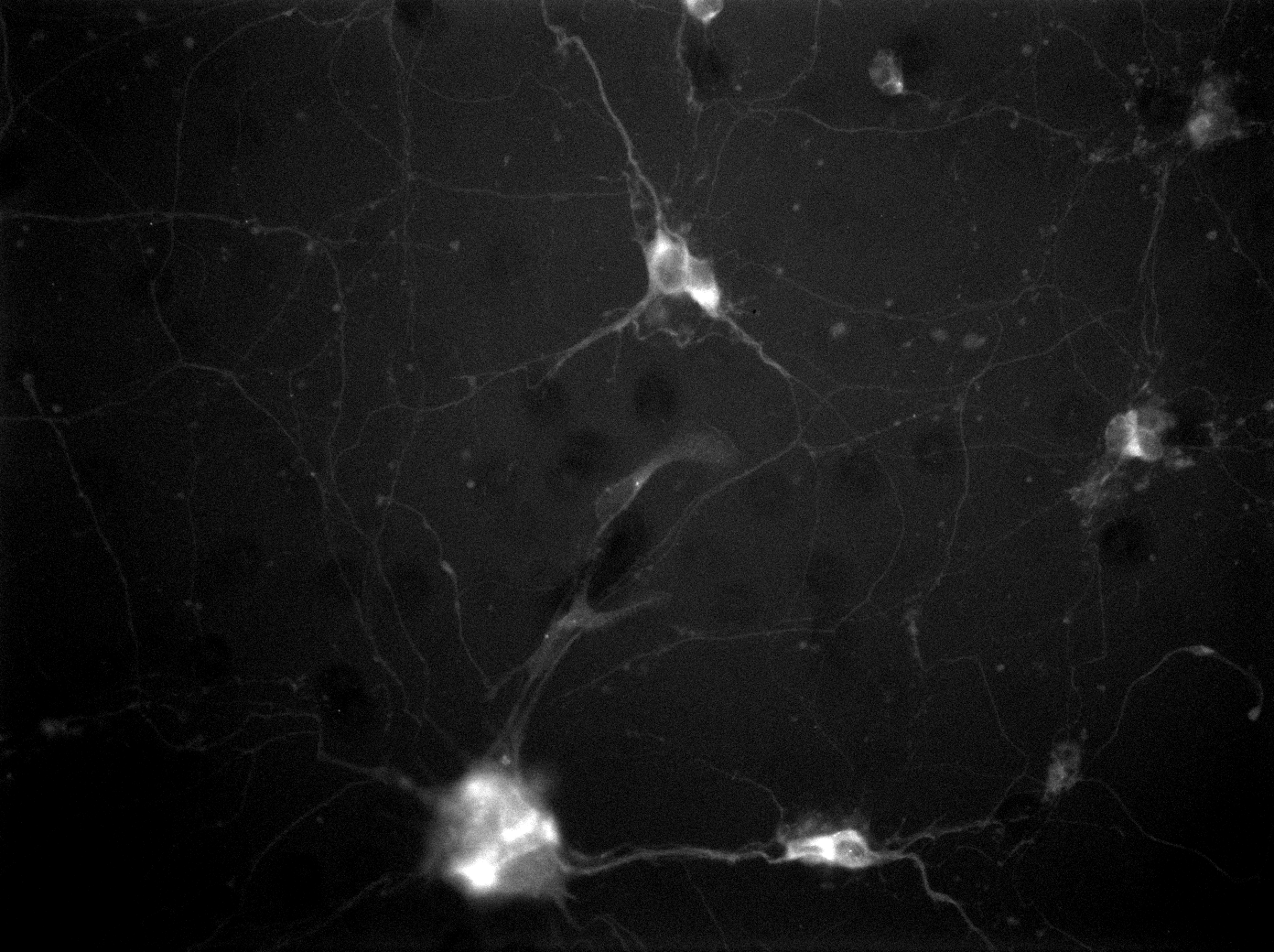

Supplement: Supplementary file 14 — Source data Fig. 9 [file 44318_2024_307_MOESM14_ESM.zip › EMBOJ-2024-116734_sourcedata_Fig 9/Fig 9_Sp--KO and TTLL6_cortico_GFP.tif]

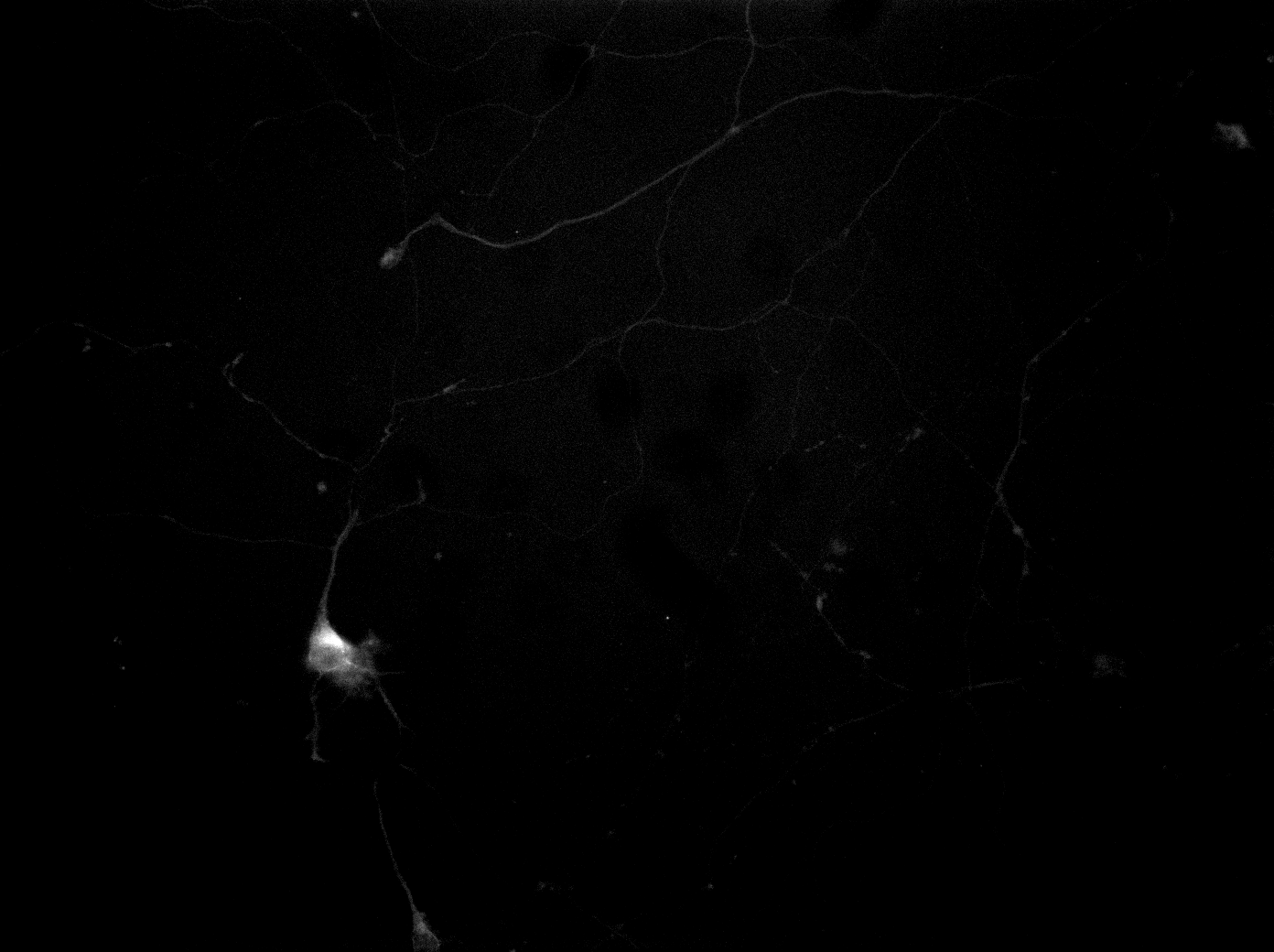

Supplement: Supplementary file 14 — Source data Fig. 9 [file 44318_2024_307_MOESM14_ESM.zip › EMBOJ-2024-116734_sourcedata_Fig 9/Fig 9_Sp+-heteroandTTLL6_cortico_GFP.tif]

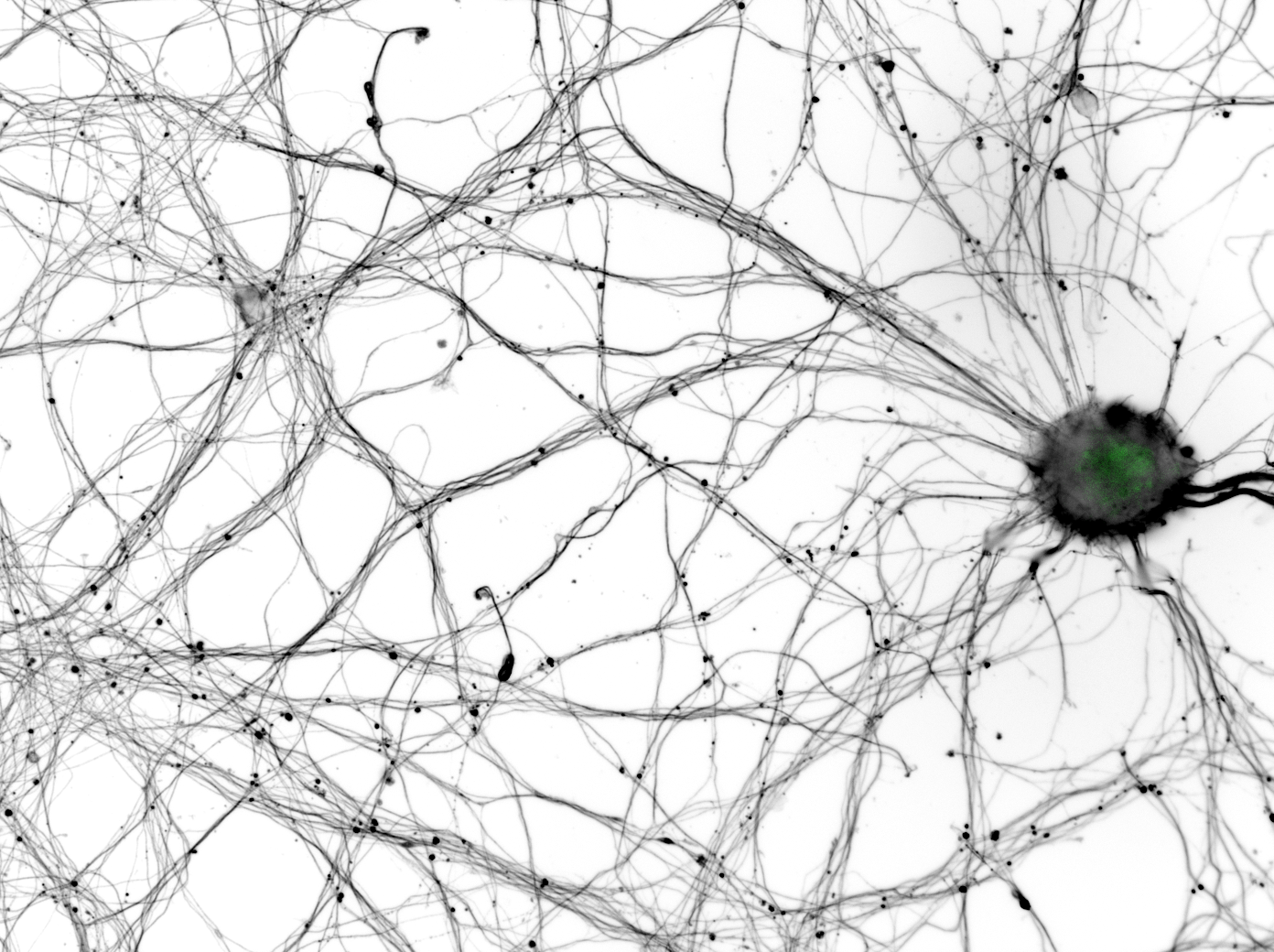

Supplement: Supplementary file 14 — Source data Fig. 9 [file 44318_2024_307_MOESM14_ESM.zip › EMBOJ-2024-116734_sourcedata_Fig 9/Fig 9_Sp--KO NT_cortico_tubulinGFP.tif]

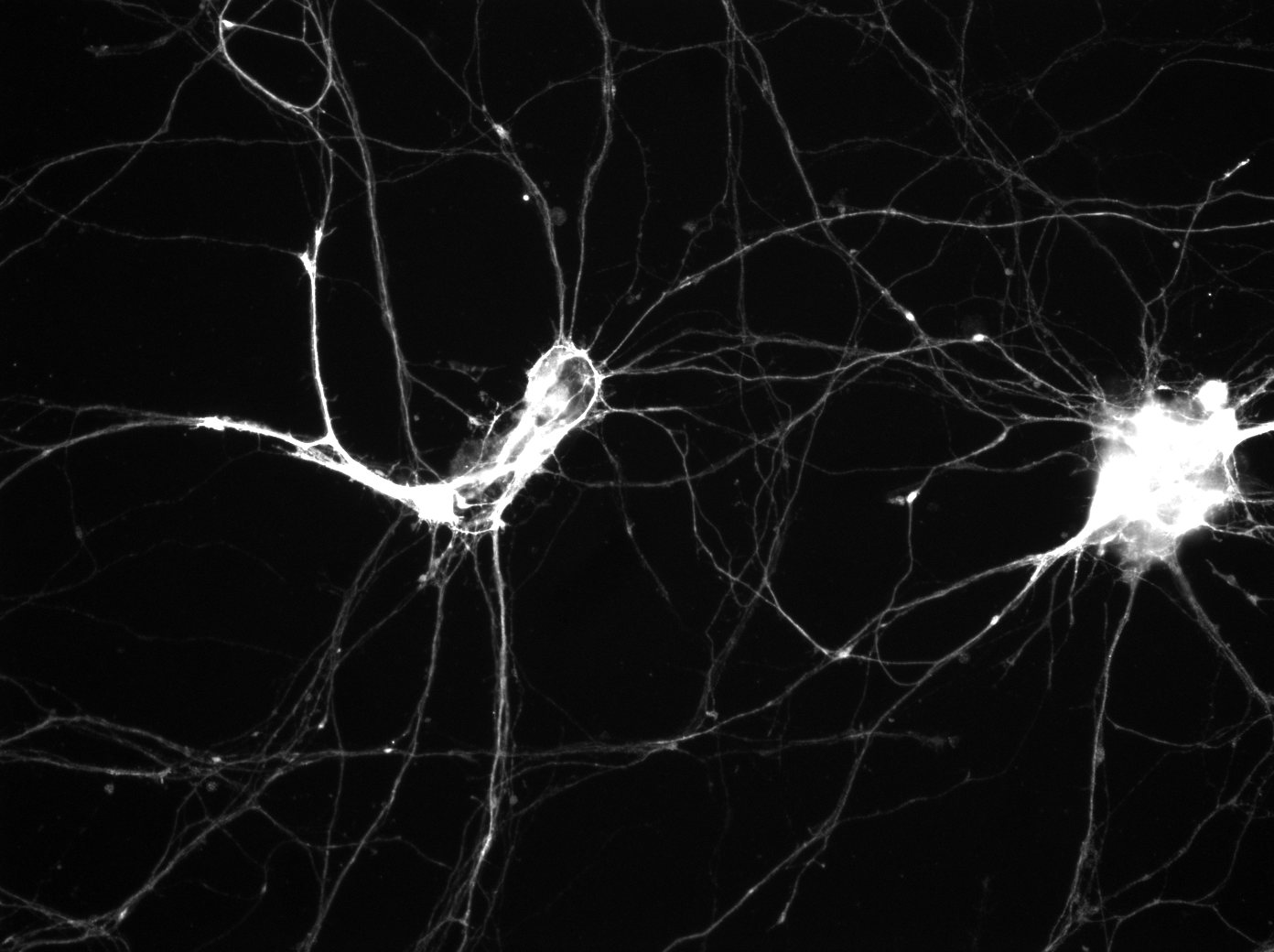

Supplement: Supplementary file 14 — Source data Fig. 9 [file 44318_2024_307_MOESM14_ESM.zip › EMBOJ-2024-116734_sourcedata_Fig 9/Fig 9_Sp--KO and TTLL11_cortico_GFP.tif]

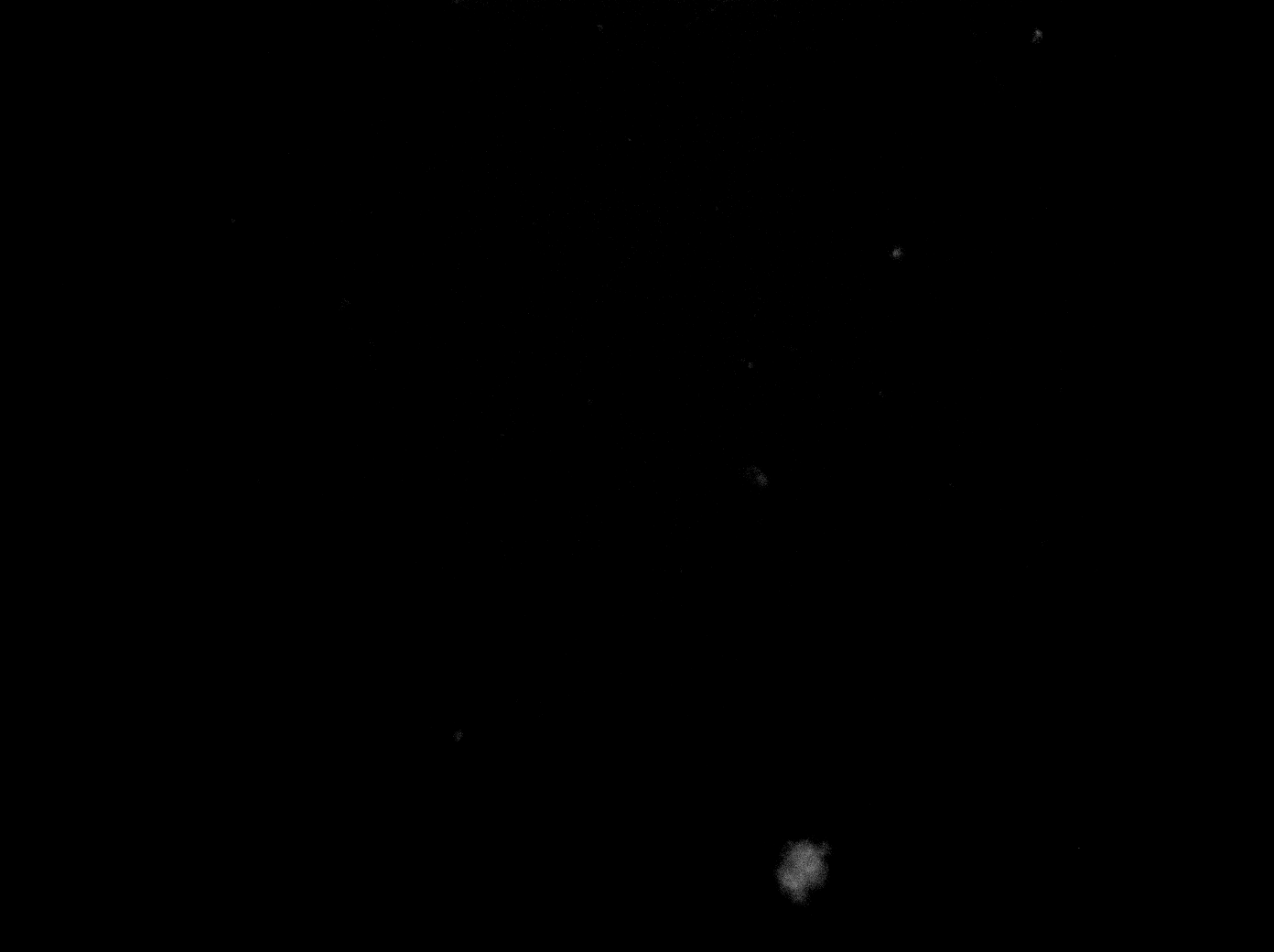

Supplement: Supplementary file 14 — Source data Fig. 9 [file 44318_2024_307_MOESM14_ESM.zip › EMBOJ-2024-116734_sourcedata_Fig 9/Fig 9_Sp++ NT_cortico_GFP.tif]

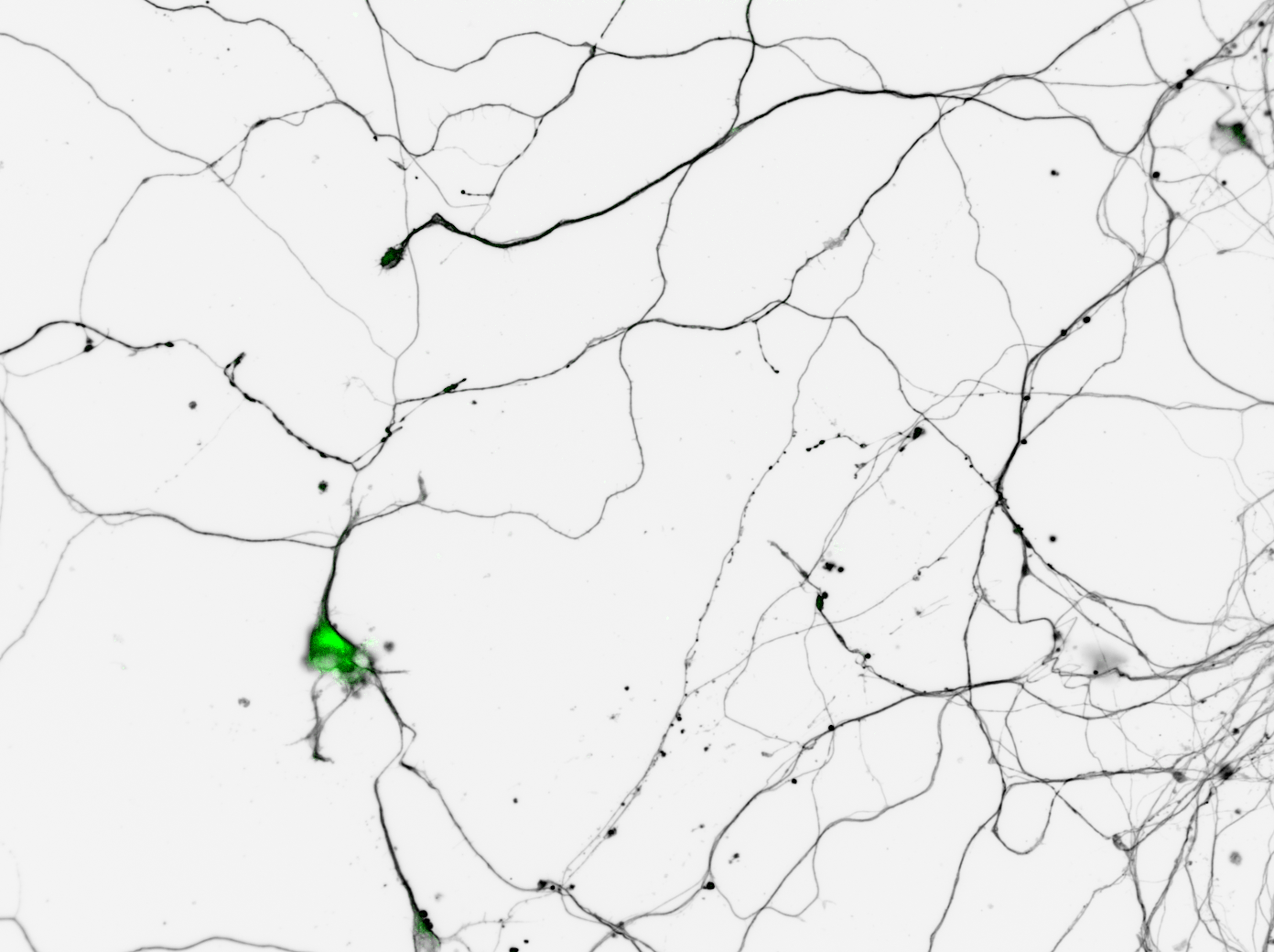

Supplement: Supplementary file 14 — Source data Fig. 9 [file 44318_2024_307_MOESM14_ESM.zip › EMBOJ-2024-116734_sourcedata_Fig 9/Fig 9_Sp+-heteroandTTLL6_cortico_tubulinGFP.tif]

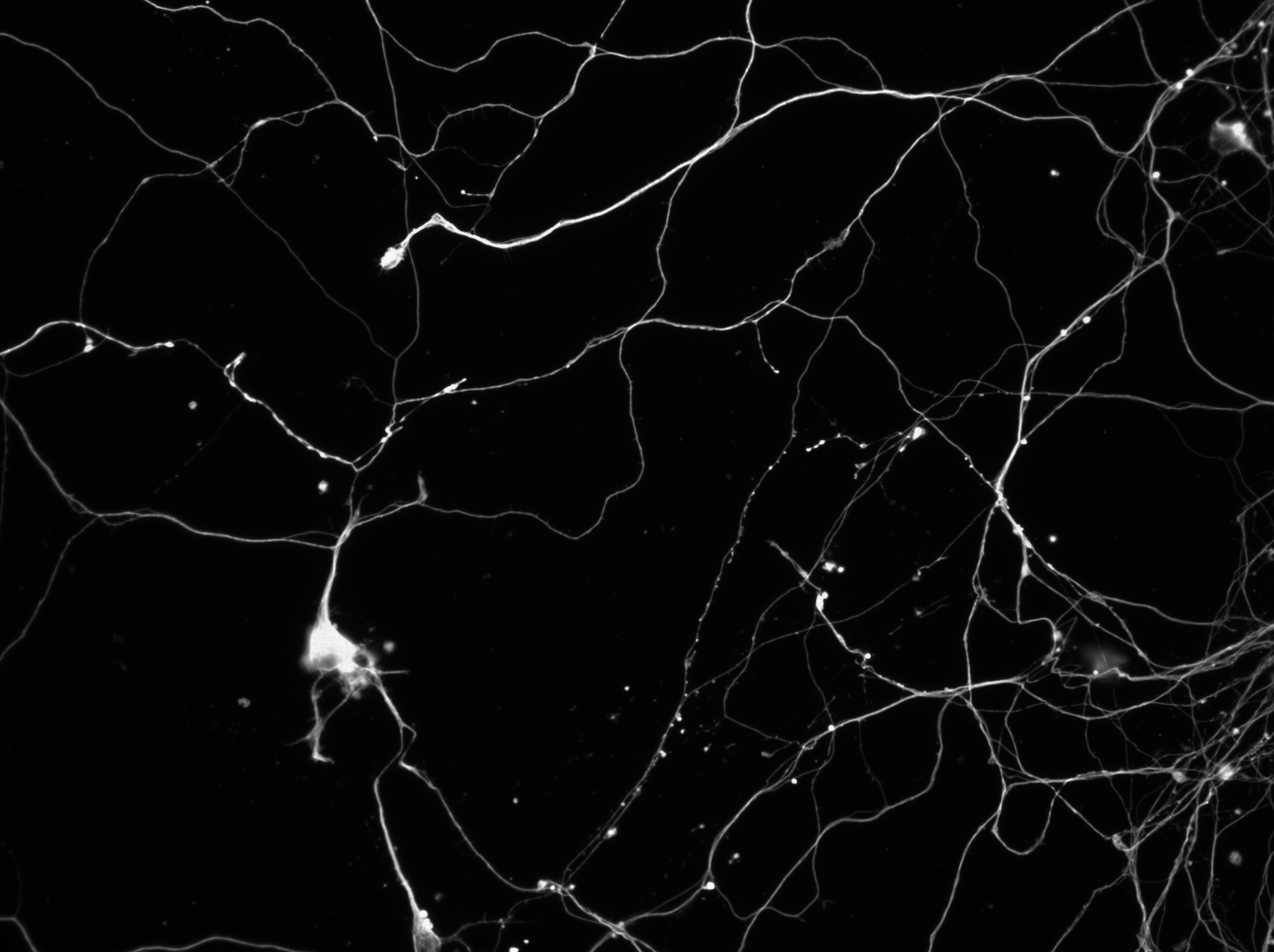

Supplement: Supplementary file 14 — Source data Fig. 9 [file 44318_2024_307_MOESM14_ESM.zip › EMBOJ-2024-116734_sourcedata_Fig 9/Fig 9_Sp+-heteroandTTLL6_cortico_tubulin.tif]

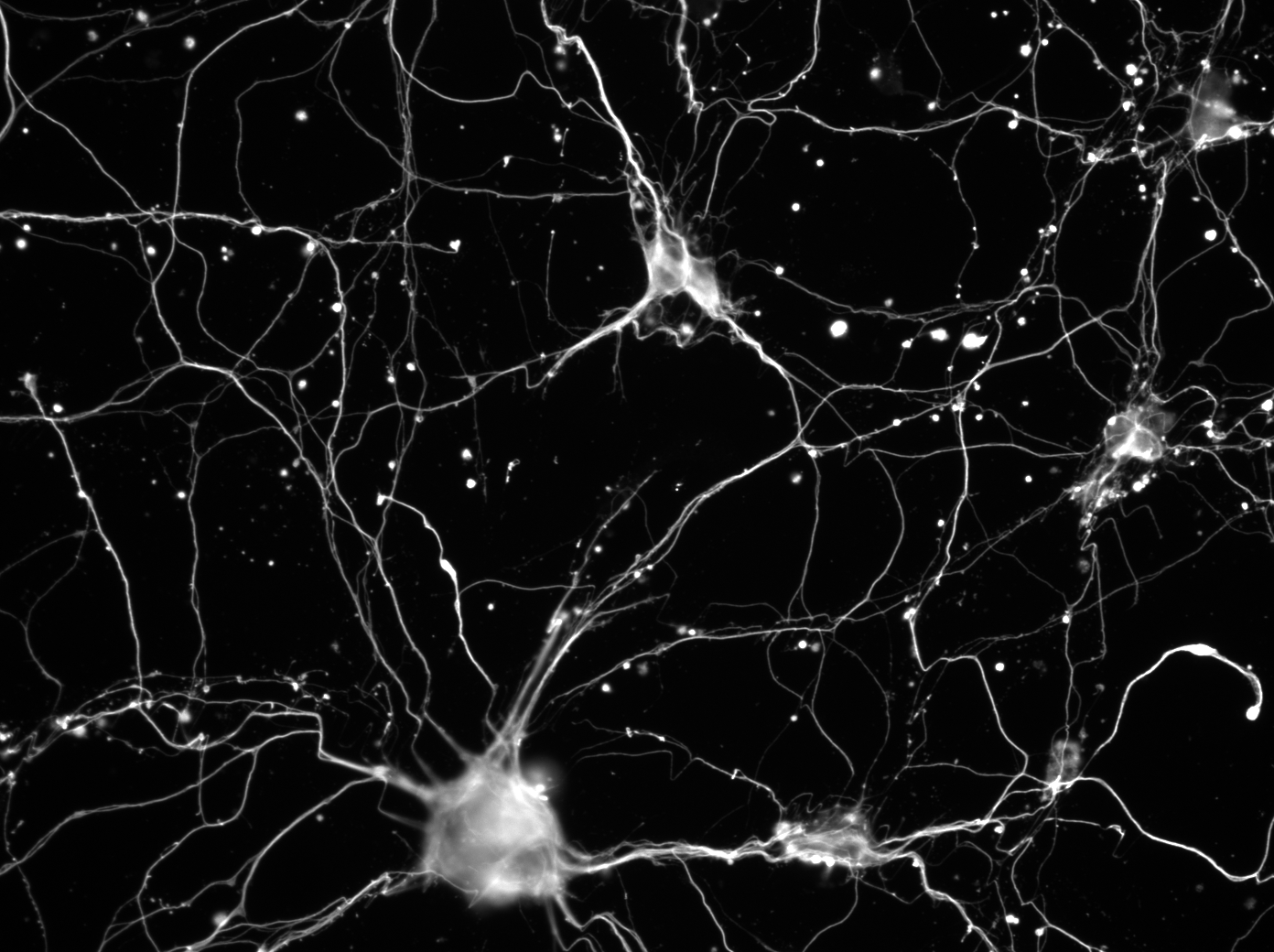

Supplement: Supplementary file 14 — Source data Fig. 9 [file 44318_2024_307_MOESM14_ESM.zip › EMBOJ-2024-116734_sourcedata_Fig 9/Fig 9_Sp--KO and TTLL6_cortico_tubulin.tif]

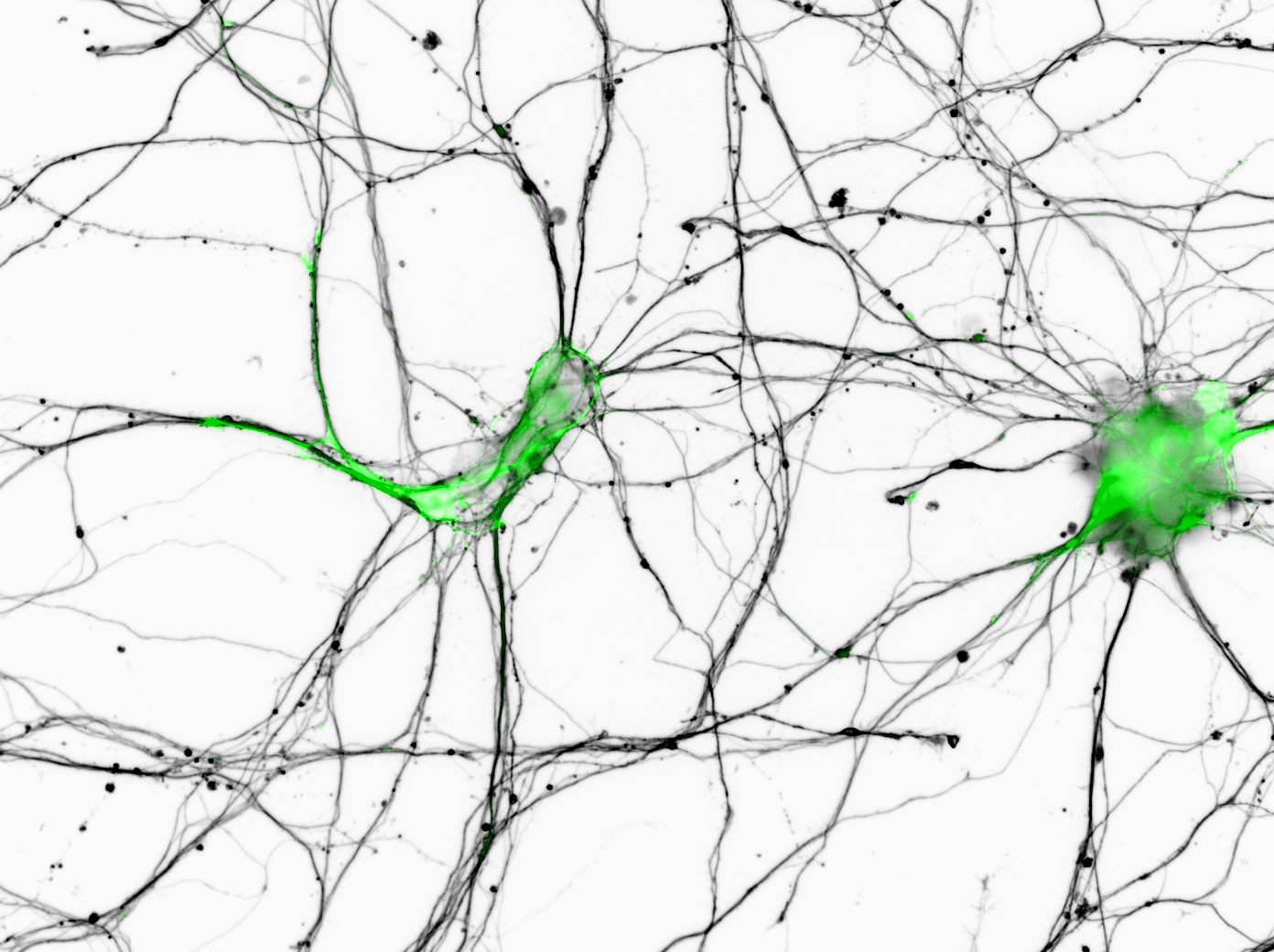

Supplement: Supplementary file 14 — Source data Fig. 9 [file 44318_2024_307_MOESM14_ESM.zip › EMBOJ-2024-116734_sourcedata_Fig 9/Fig 9_Sp--KO and TTLL11_cortico_tubulinGFP.tif]

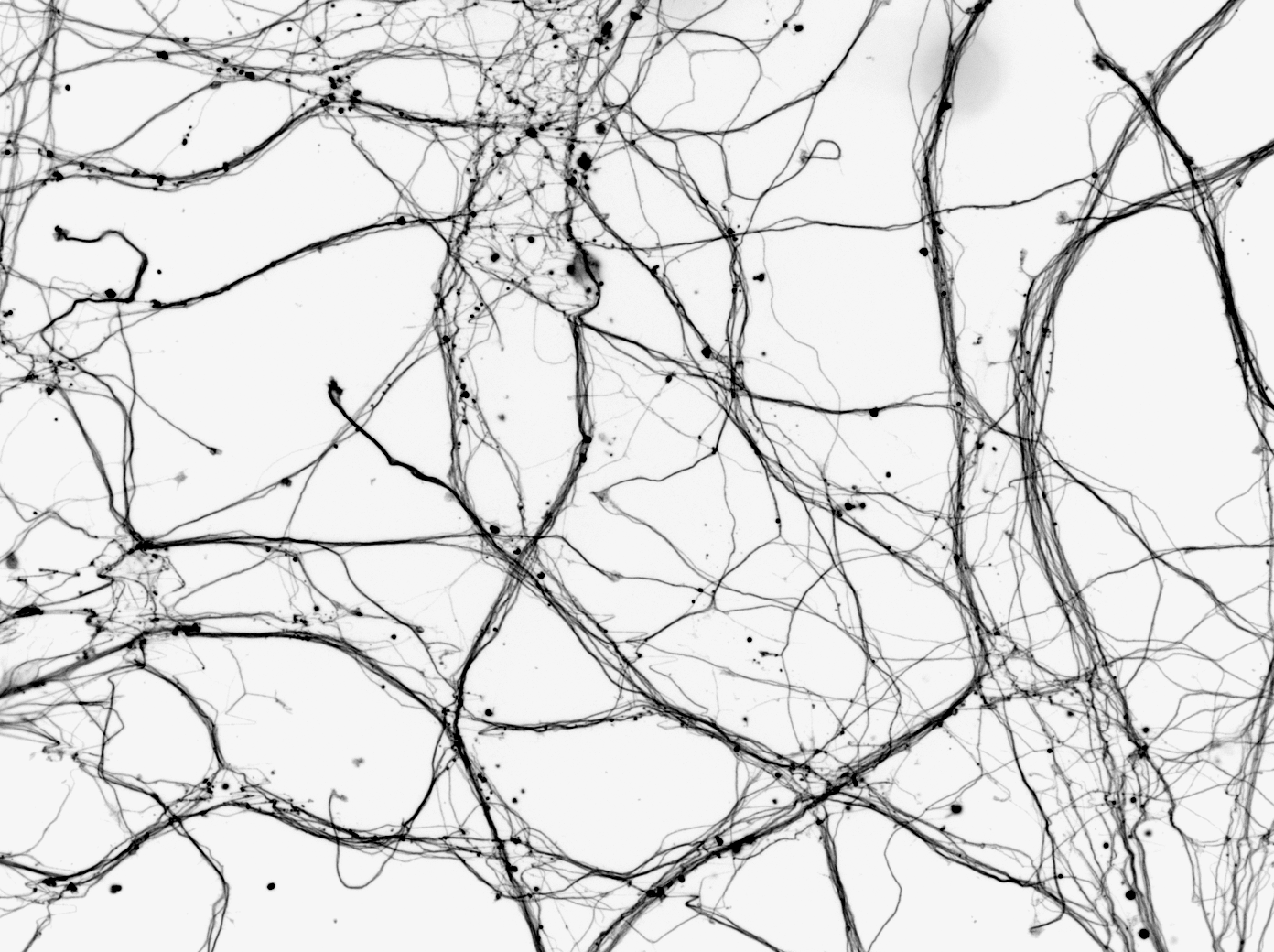

Supplement: Supplementary file 14 — Source data Fig. 9 [file 44318_2024_307_MOESM14_ESM.zip › EMBOJ-2024-116734_sourcedata_Fig 9/Fig 9_Sp+-hetero NT_cortico_tubulinGFP.tif]

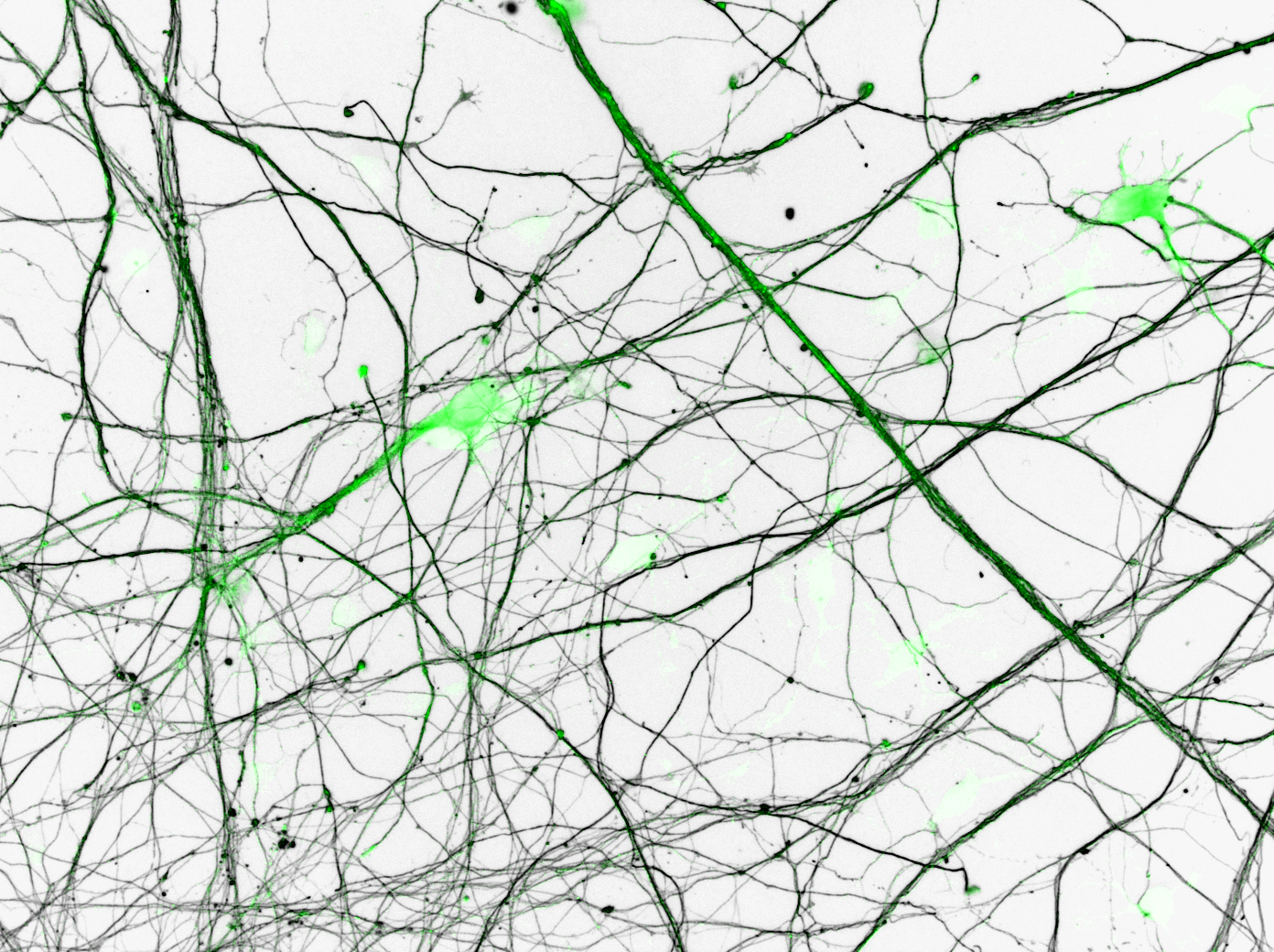

Supplement: Supplementary file 14 — Source data Fig. 9 [file 44318_2024_307_MOESM14_ESM.zip › EMBOJ-2024-116734_sourcedata_Fig 9/Fig 9_Sp+-heteroandTTLL11_cortico_tubulinGFP.tif]

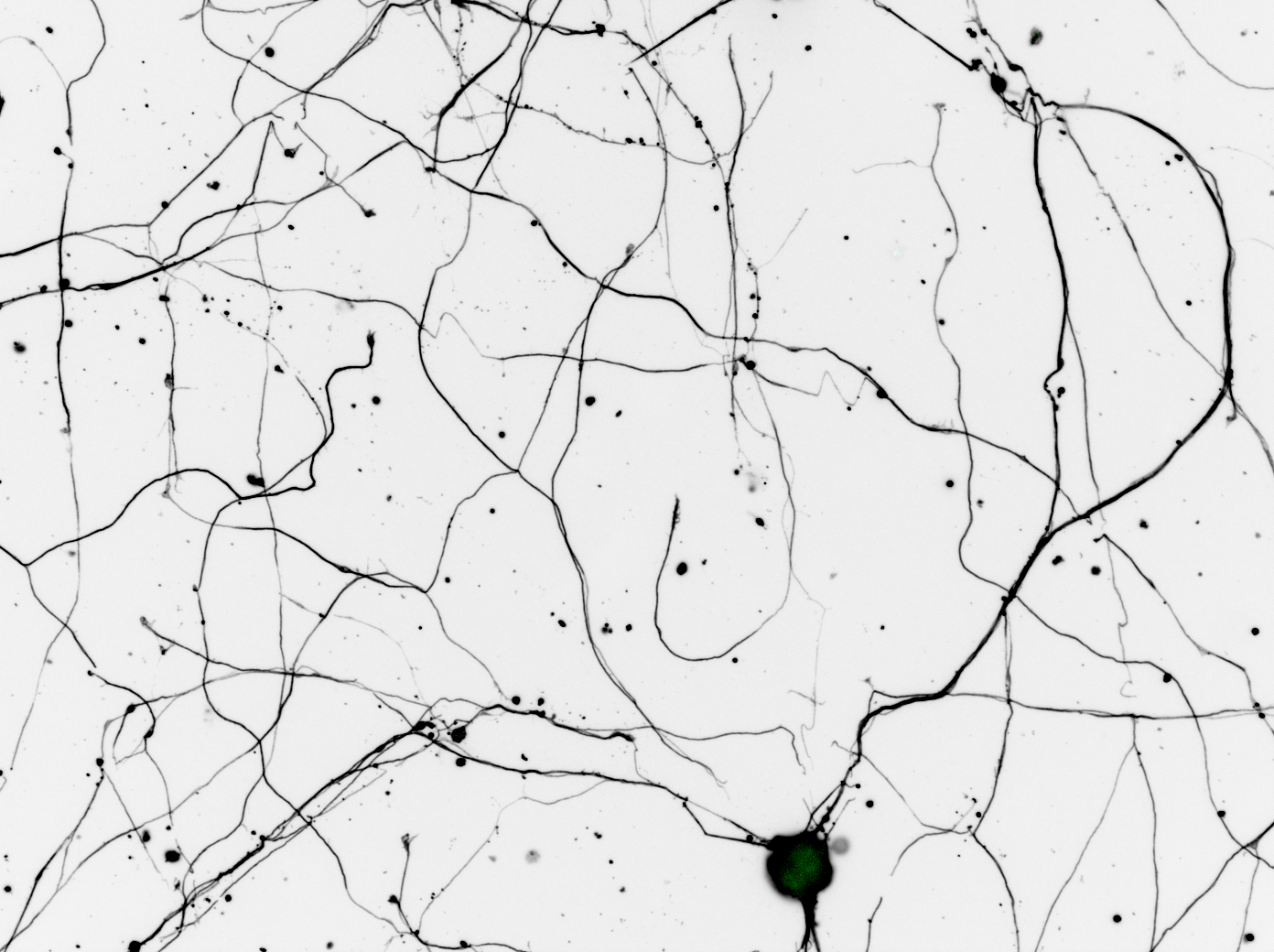

Supplement: Supplementary file 14 — Source data Fig. 9 [file 44318_2024_307_MOESM14_ESM.zip › EMBOJ-2024-116734_sourcedata_Fig 9/Fig 9_Sp++ NT_cortico_tubulinGFP.tif]

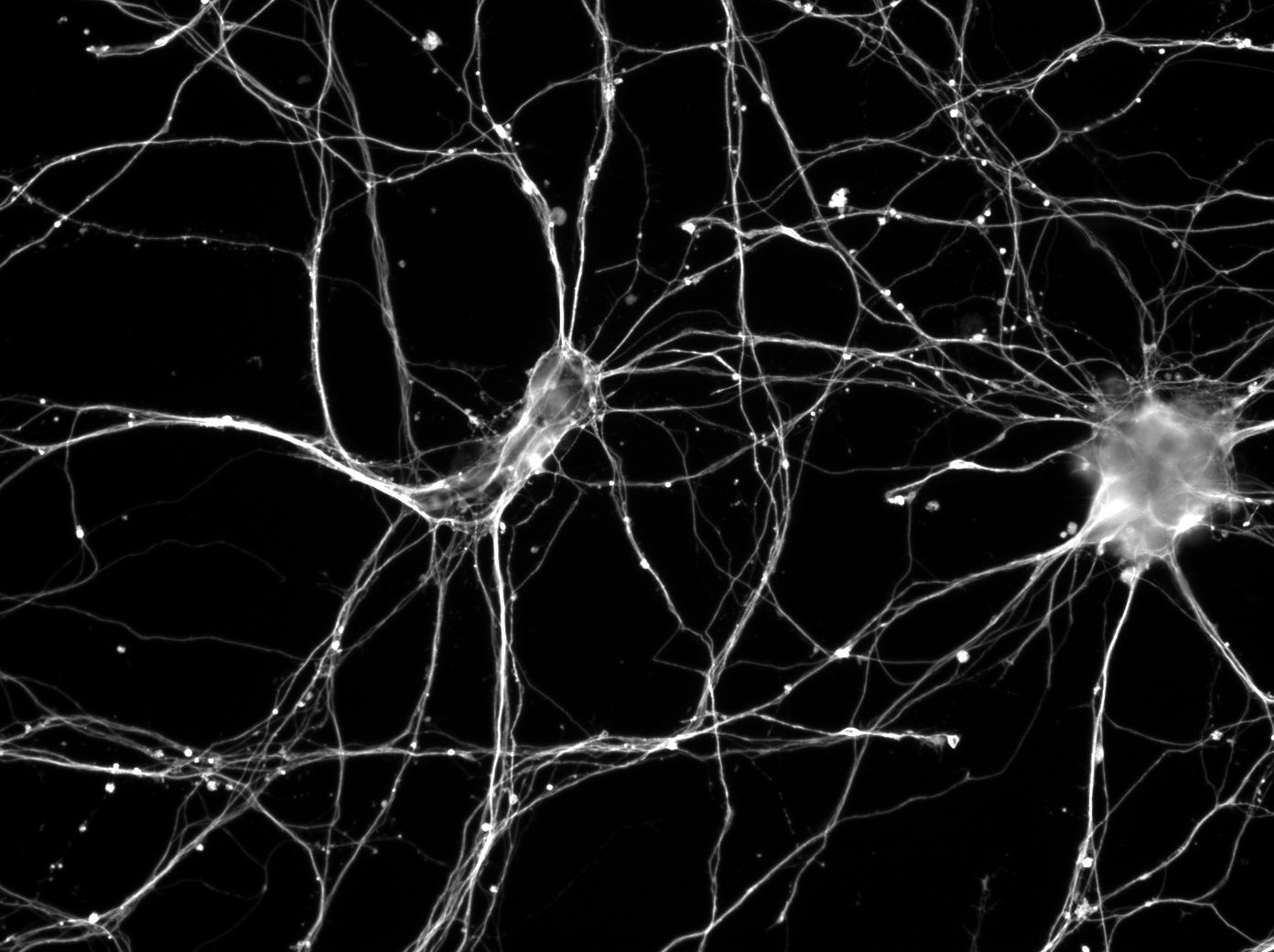

Supplement: Supplementary file 14 — Source data Fig. 9 [file 44318_2024_307_MOESM14_ESM.zip › EMBOJ-2024-116734_sourcedata_Fig 9/Fig 9_Sp--KO and TTLL11_cortico_tubulin.tif]
