# Supplementary figures and images for: Tubulin glutamylation regulates axon guidance via the selective tuning of microtubule-severing enzymes (part 2 of 2)
Source: EMBO J. 2024 Nov 29;44(1):107–40. doi: 10.1038/s44318-024-00307-x (PMC11695996; doi:10.1038/s44318-024-00307-x)

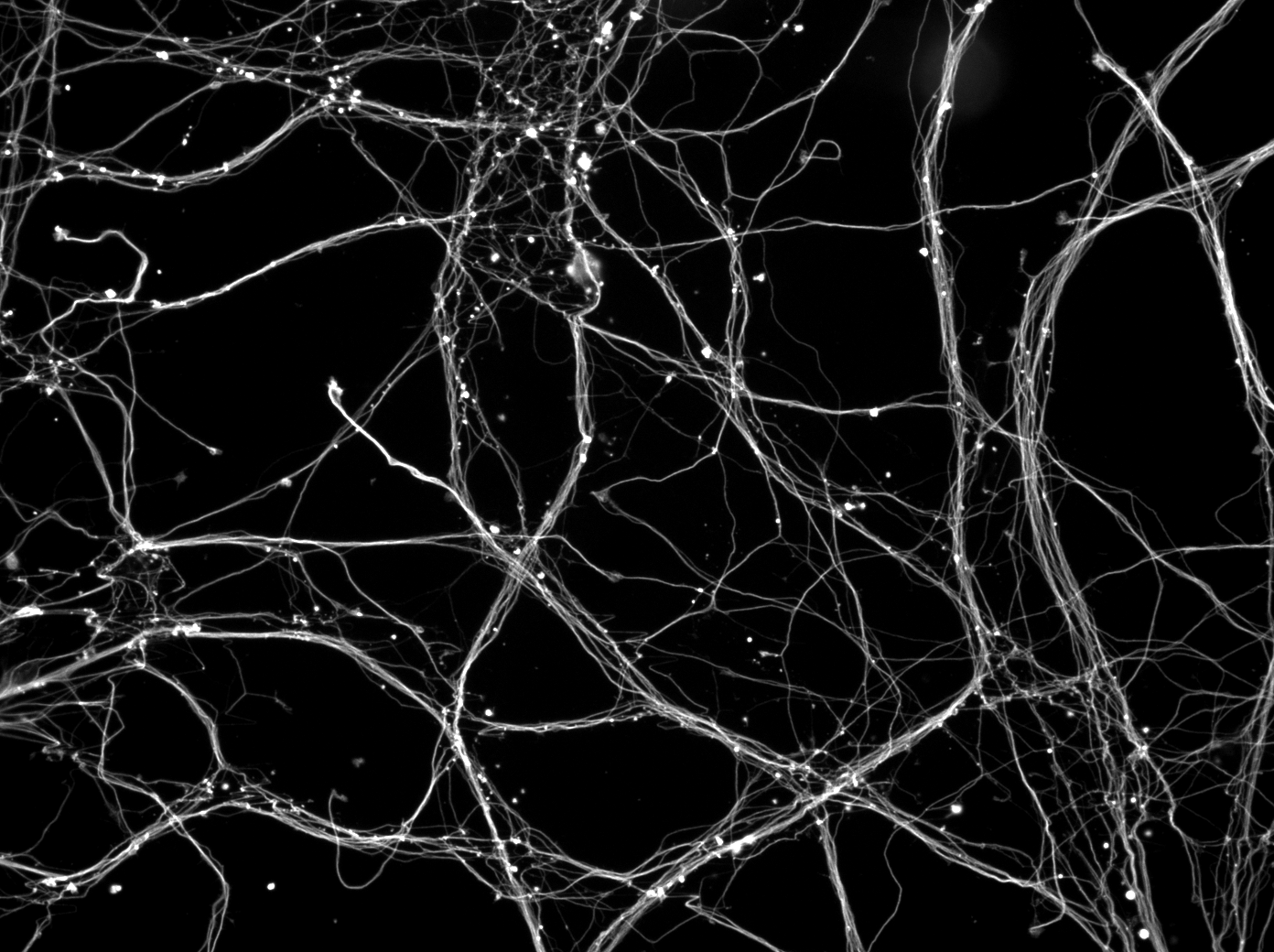

Supplement: Supplementary file 14 — Source data Fig. 9 [file 44318_2024_307_MOESM14_ESM.zip › EMBOJ-2024-116734_sourcedata_Fig 9/Fig 9_Sp+-hetero NT_cortico_tubulin.tif]

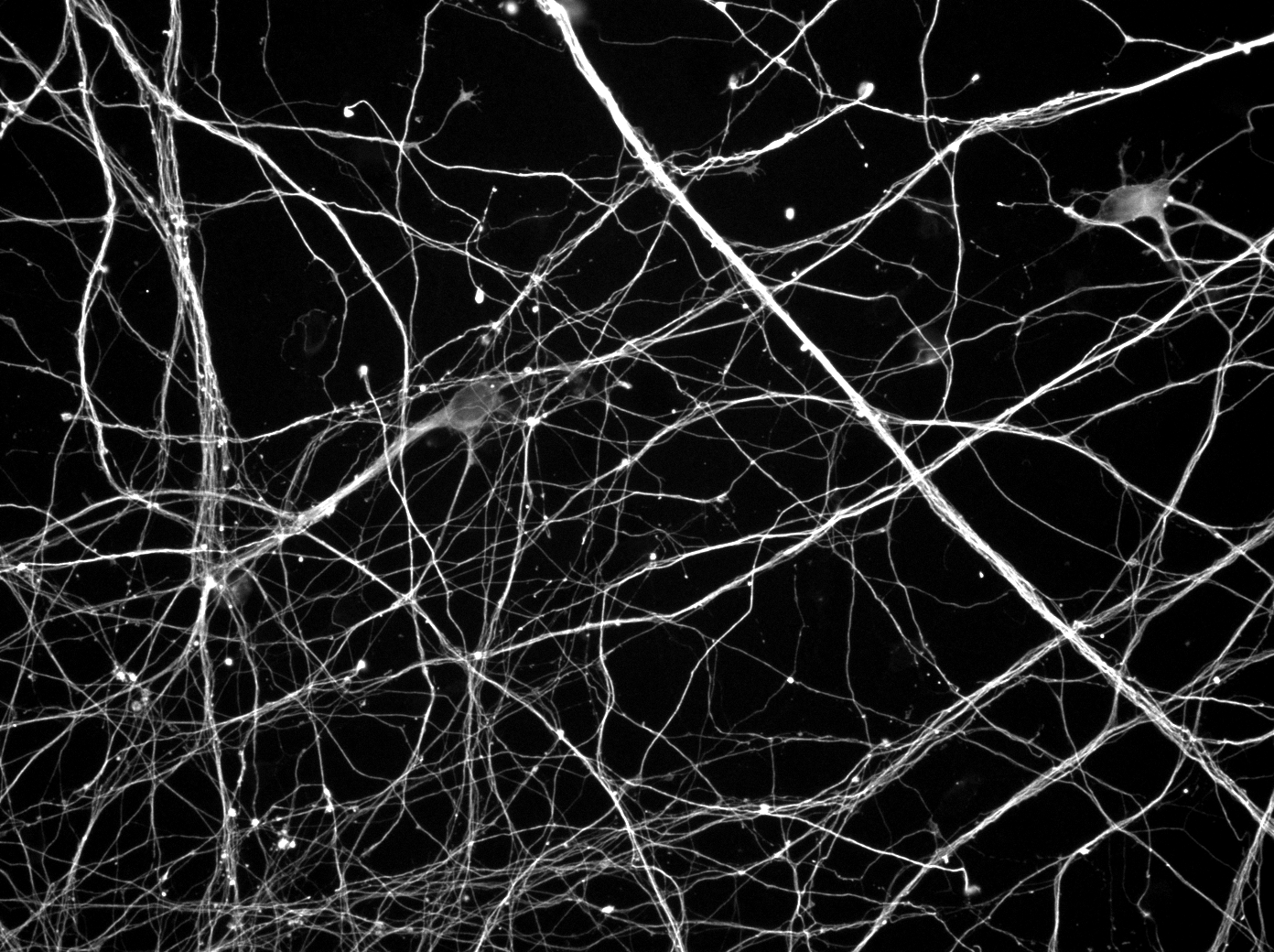

Supplement: Supplementary file 14 — Source data Fig. 9 [file 44318_2024_307_MOESM14_ESM.zip › EMBOJ-2024-116734_sourcedata_Fig 9/Fig 9_Sp+-heteroandTTLL11_cortico_tubulin.tif]

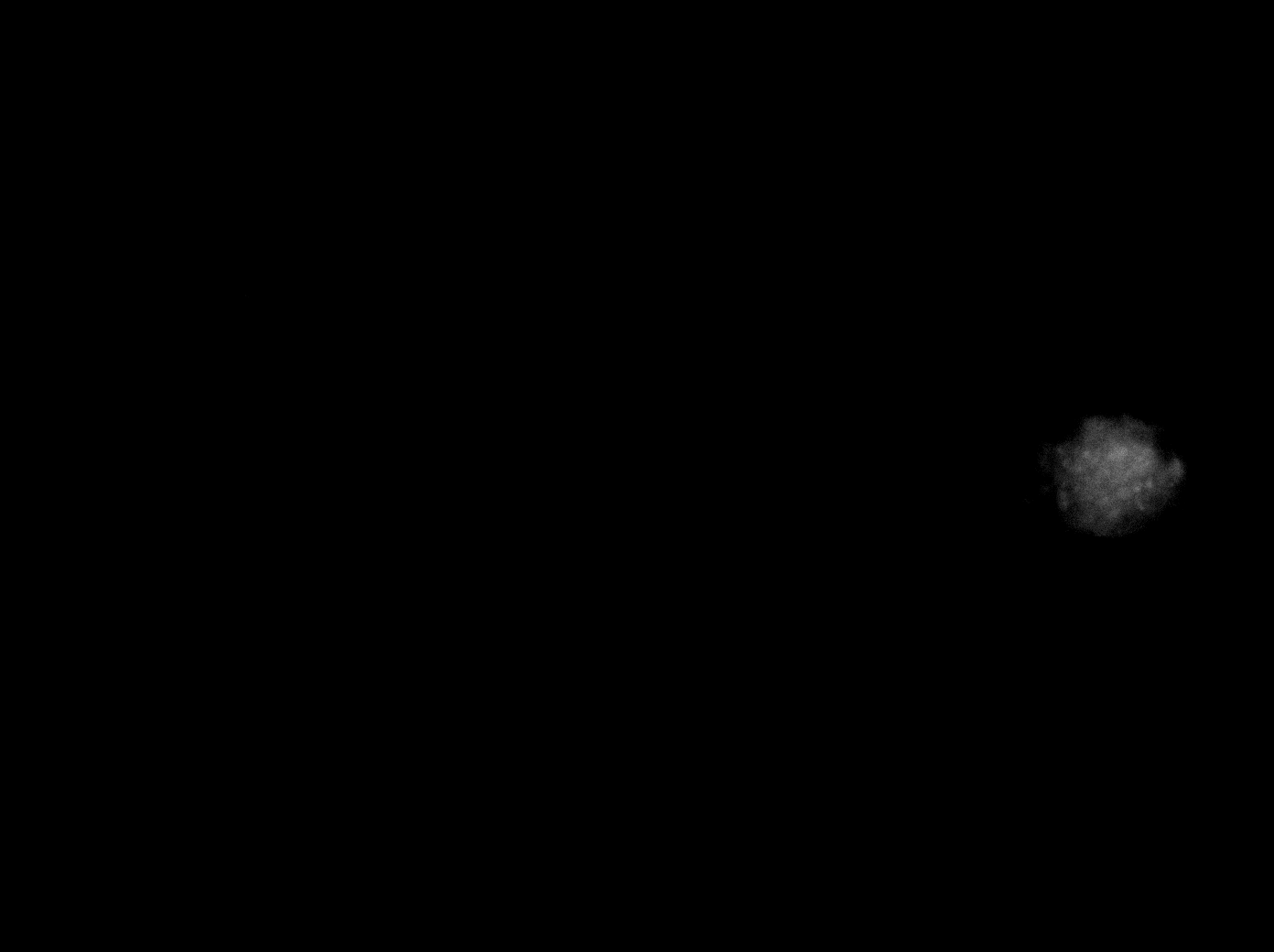

Supplement: Supplementary file 14 — Source data Fig. 9 [file 44318_2024_307_MOESM14_ESM.zip › EMBOJ-2024-116734_sourcedata_Fig 9/Fig 9_Sp--KO NT_cortico_GFP.tif]

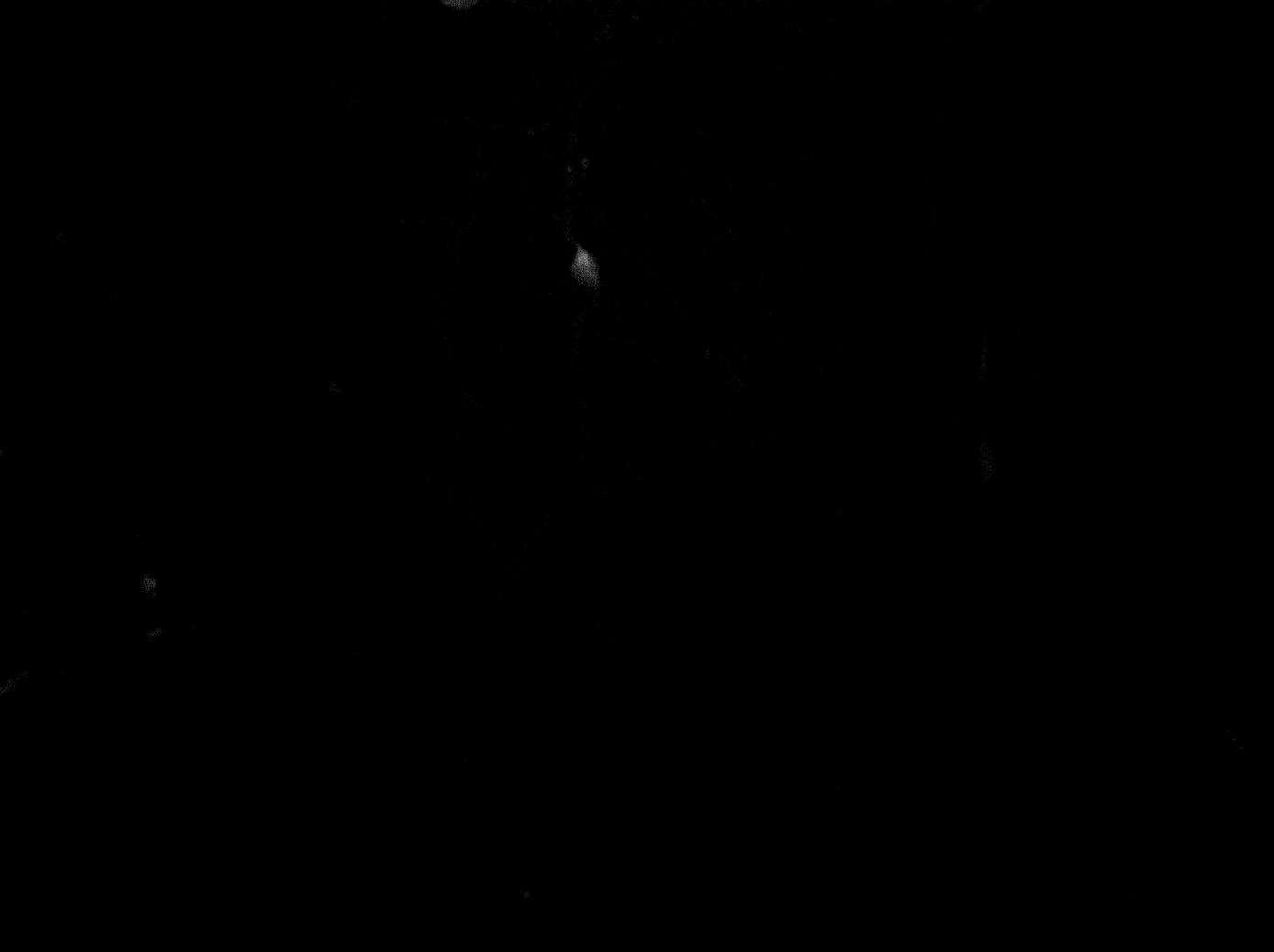

Supplement: Supplementary file 14 — Source data Fig. 9 [file 44318_2024_307_MOESM14_ESM.zip › EMBOJ-2024-116734_sourcedata_Fig 9/Fig 9_Sp+-hetero NT_cortico_GFP.tif]

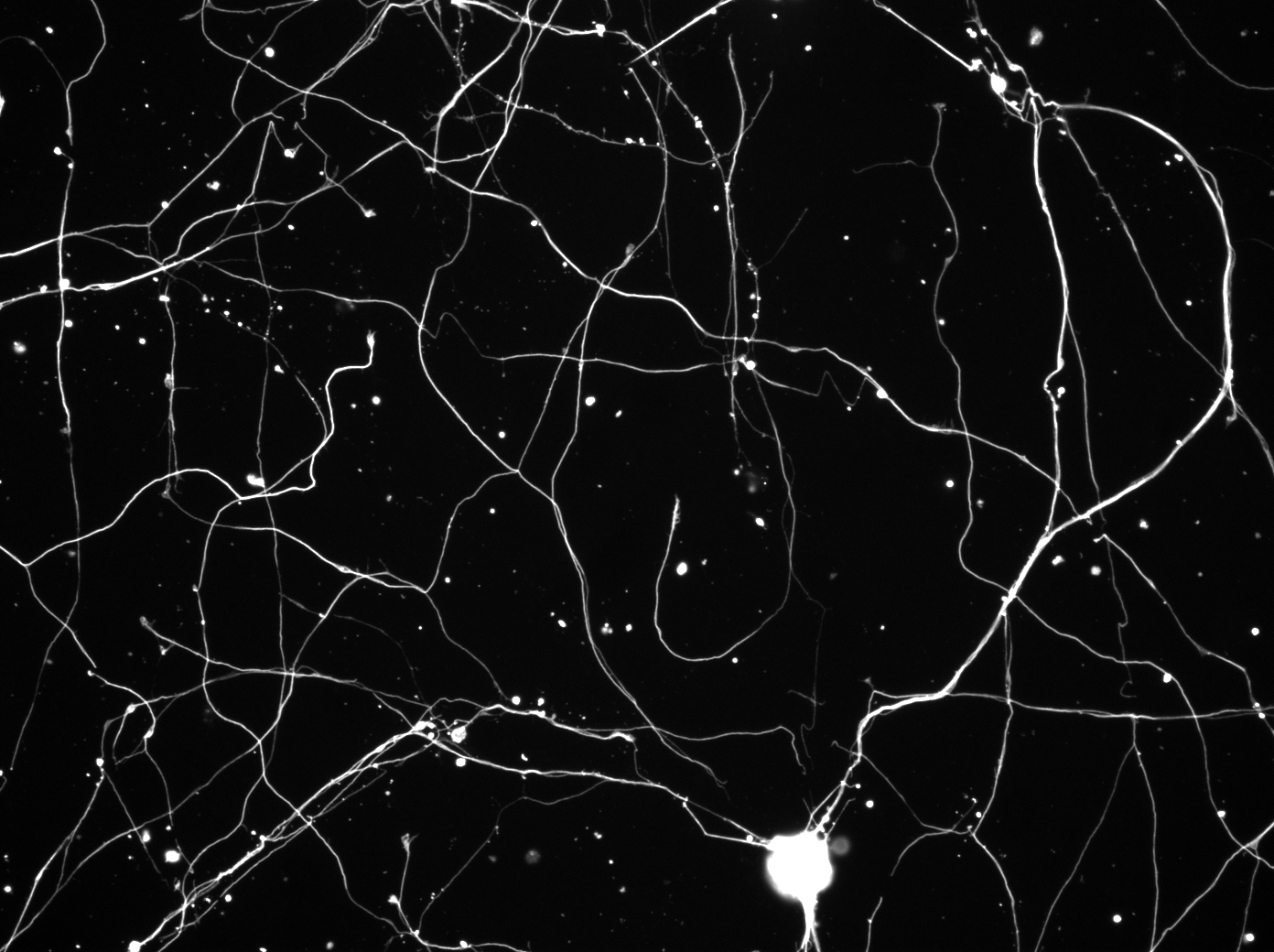

Supplement: Supplementary file 14 — Source data Fig. 9 [file 44318_2024_307_MOESM14_ESM.zip › EMBOJ-2024-116734_sourcedata_Fig 9/Fig 9_Sp++ NT_cortico_tubulin.tif]

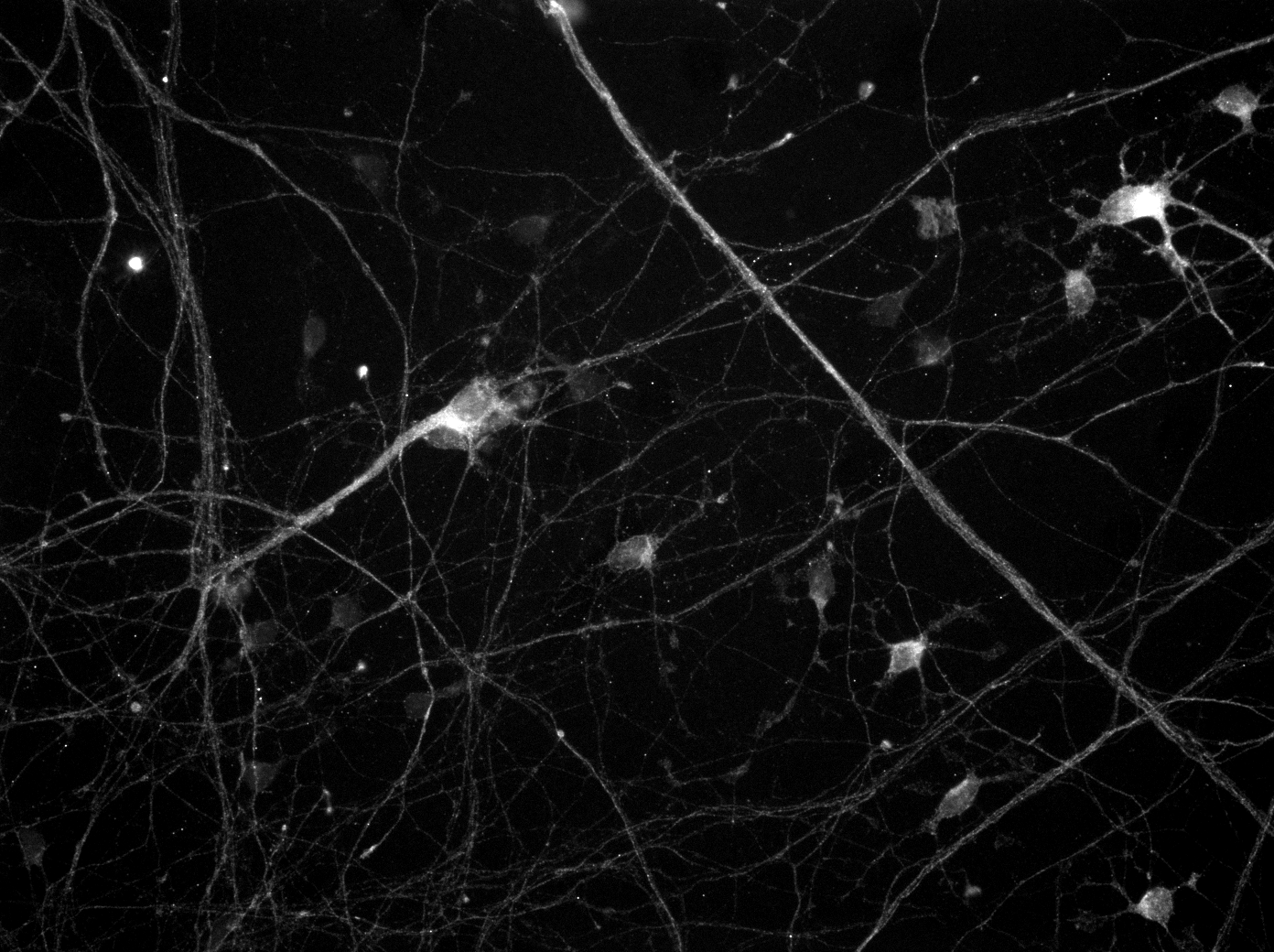

Supplement: Supplementary file 14 — Source data Fig. 9 [file 44318_2024_307_MOESM14_ESM.zip › EMBOJ-2024-116734_sourcedata_Fig 9/Fig 9_Sp+-heteroandTTLL11_cortico_GFP.tif]

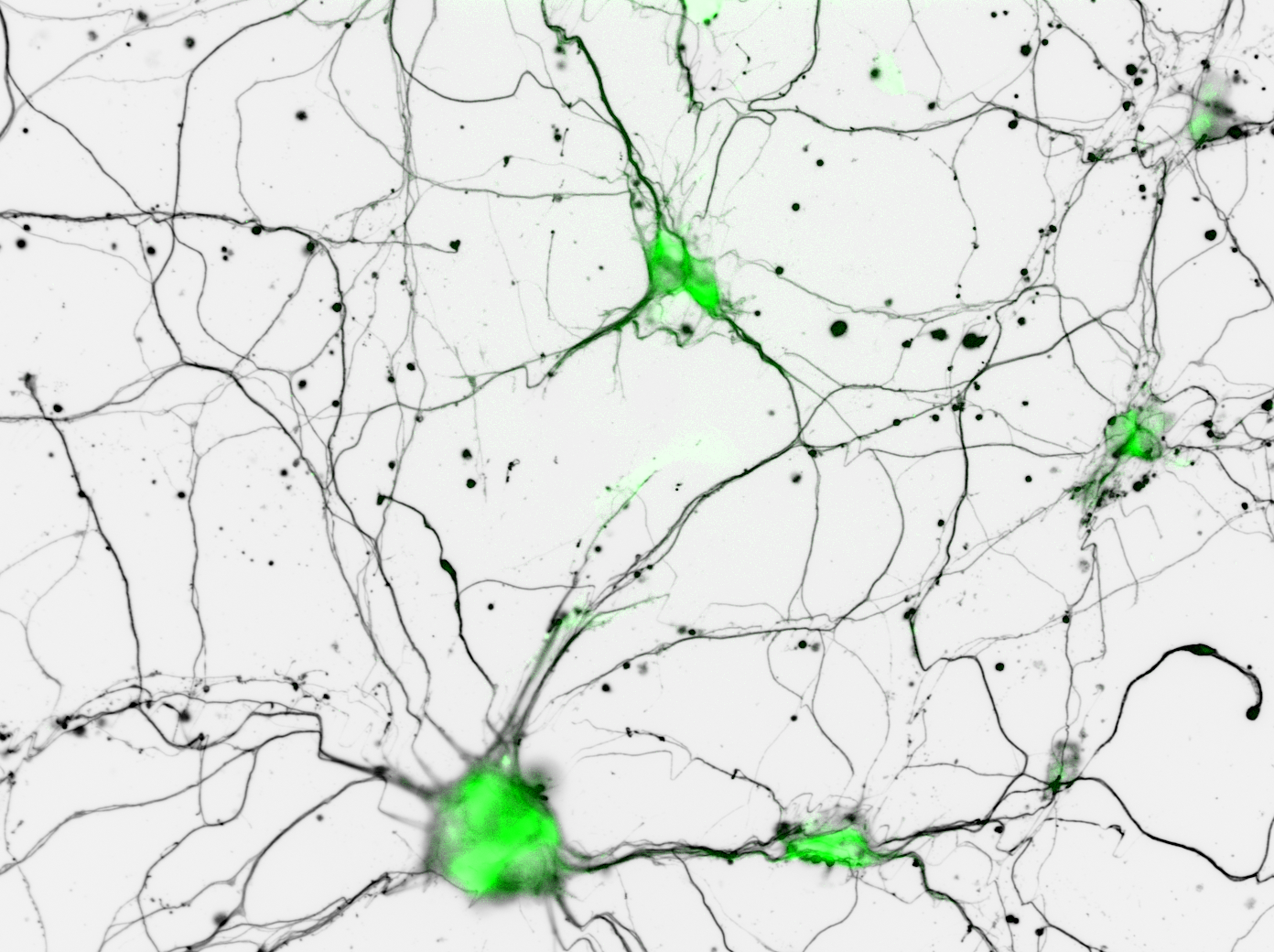

Supplement: Supplementary file 14 — Source data Fig. 9 [file 44318_2024_307_MOESM14_ESM.zip › EMBOJ-2024-116734_sourcedata_Fig 9/Fig 9_Sp--KO and TTLL6_cortico_tubulinGFP.tif]

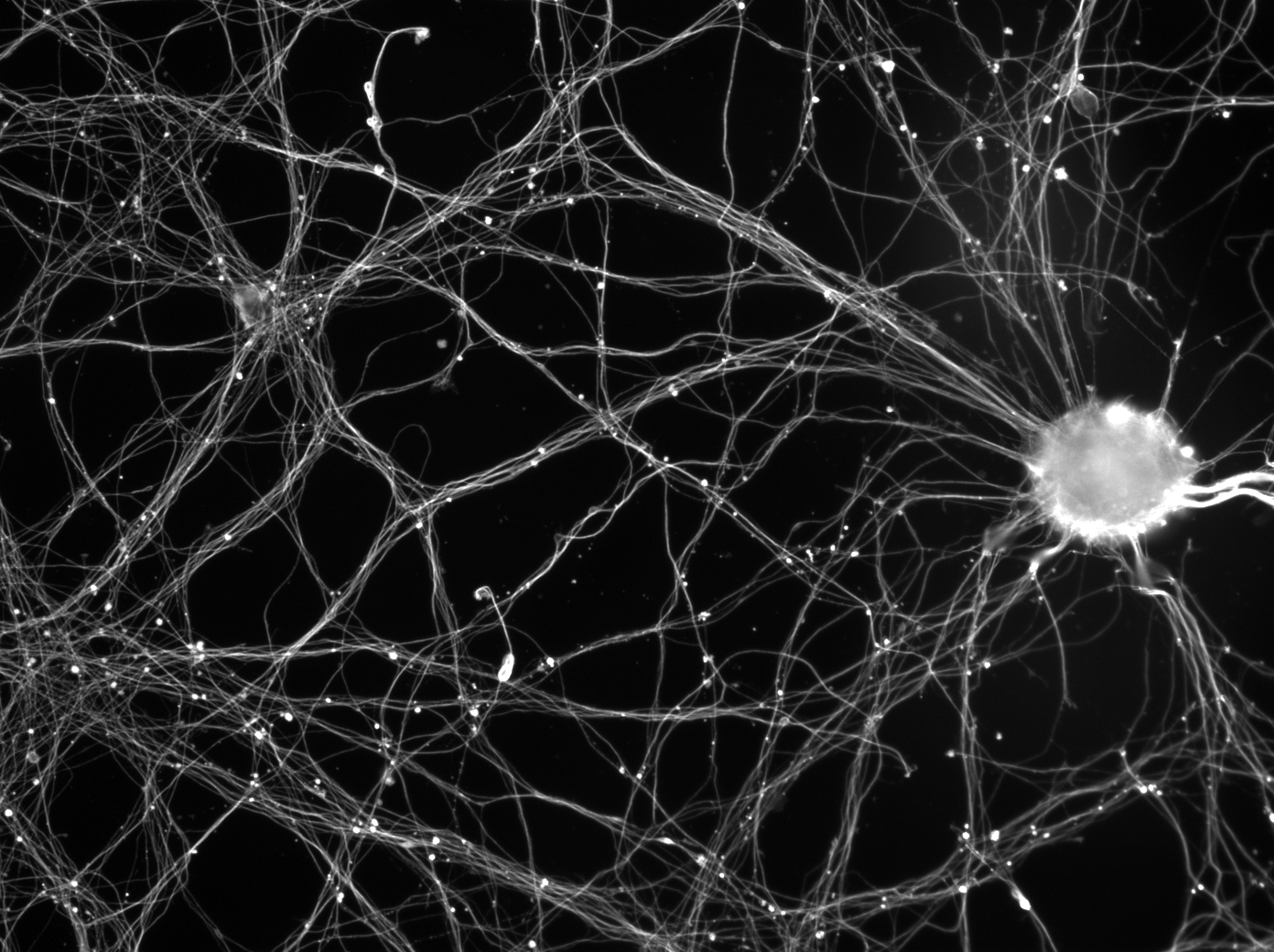

Supplement: Supplementary file 14 — Source data Fig. 9 [file 44318_2024_307_MOESM14_ESM.zip › EMBOJ-2024-116734_sourcedata_Fig 9/Fig 9_Sp--KO NT_cortico_tubulin.tif]

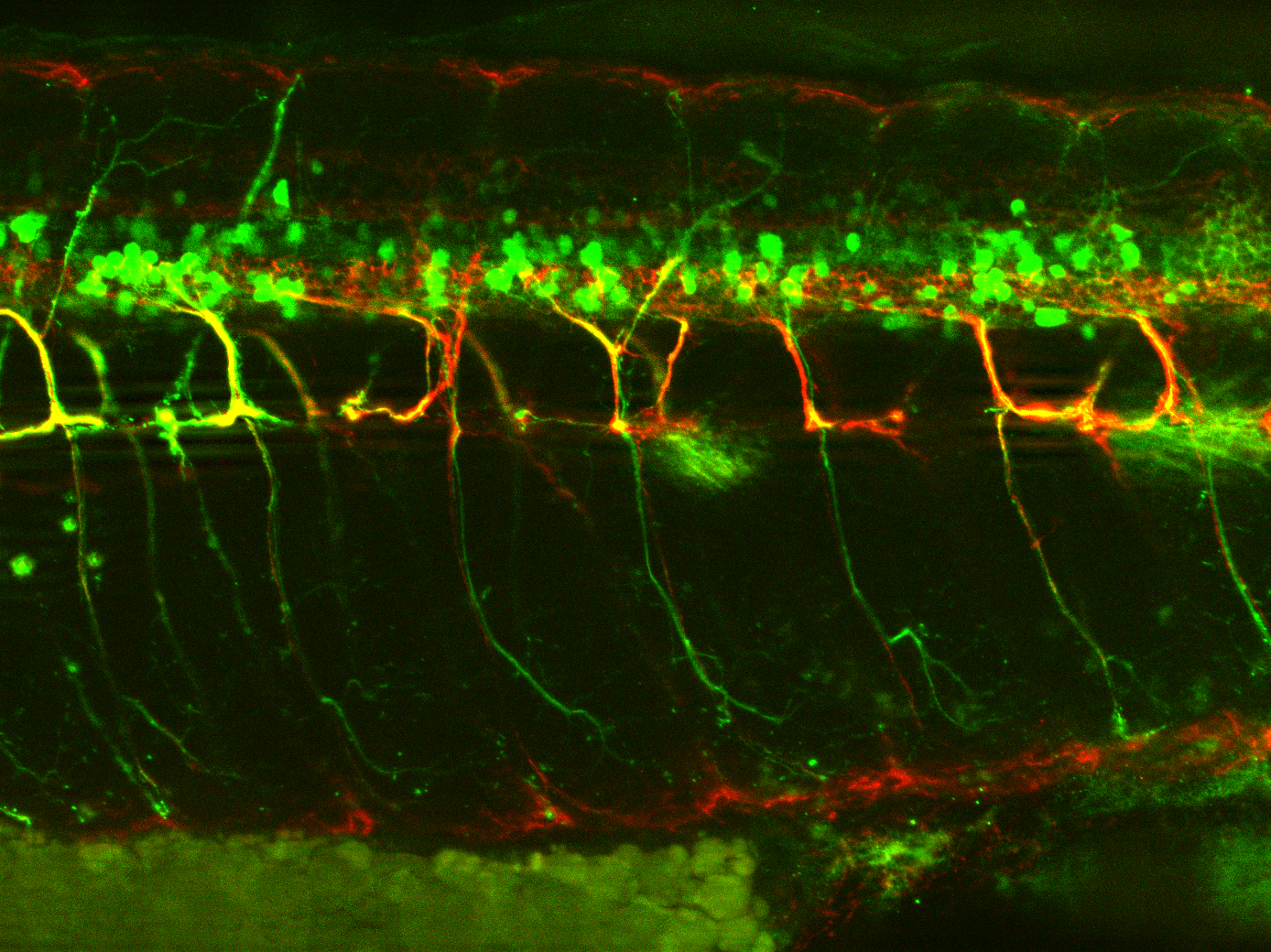

Supplement: Supplementary file 15 — EV Figure Source Data [file 44318_2024_307_MOESM15_ESM.zip › EMBOJ-2024-116734_sourcedataforexpandedviews/Fig EV1/FigEV1_panelA_MOkatna1aug1_smN_zn5GFP.tif]

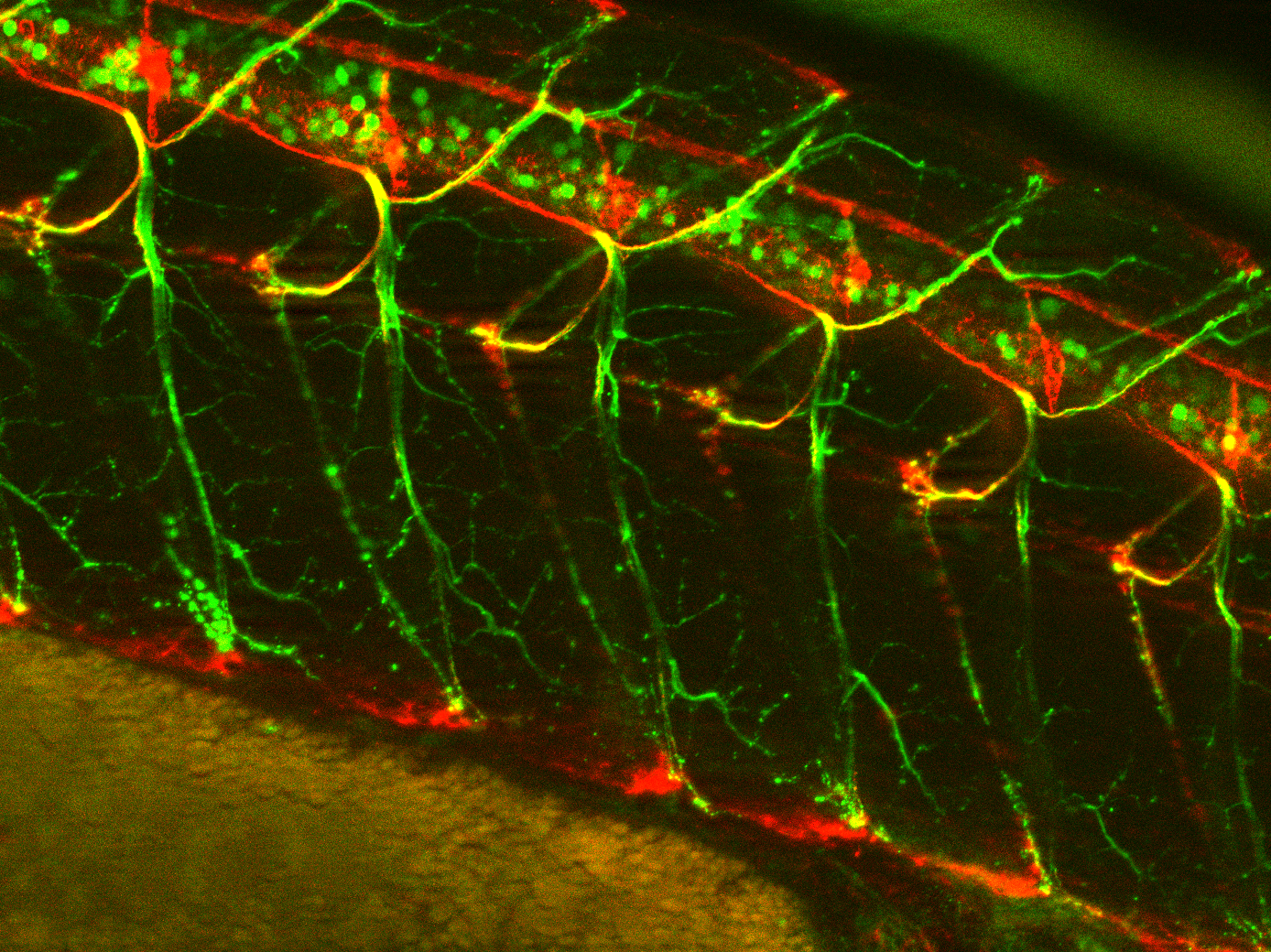

Supplement: Supplementary file 15 — EV Figure Source Data [file 44318_2024_307_MOESM15_ESM.zip › EMBOJ-2024-116734_sourcedataforexpandedviews/Fig EV1/FigEV1_panelA_MOCTL_smN_zn5GFP.tif]

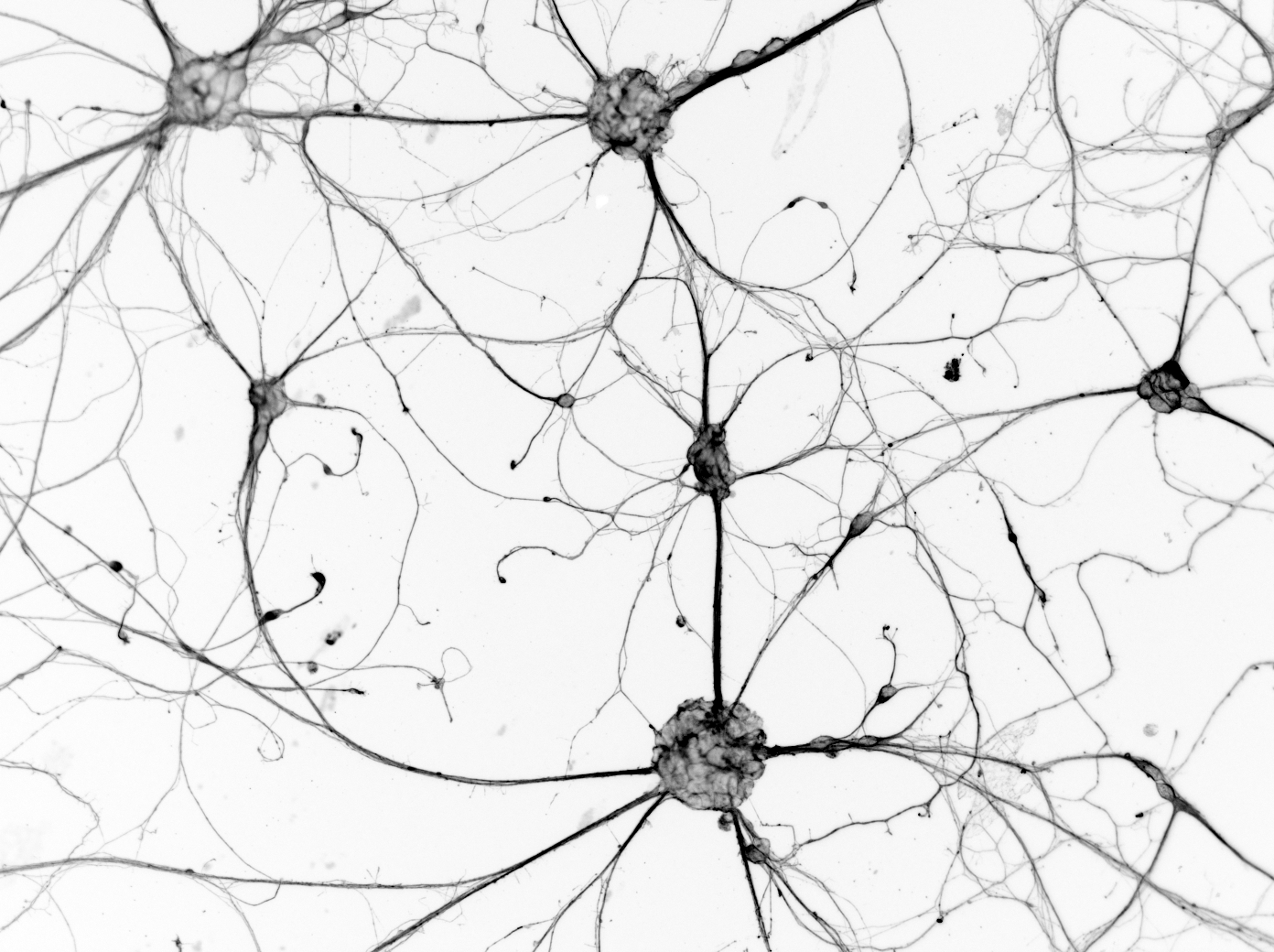

Supplement: Supplementary file 15 — EV Figure Source Data [file 44318_2024_307_MOESM15_ESM.zip › EMBOJ-2024-116734_sourcedataforexpandedviews/Fig EV6/Fig EV6_panelA_Sp--KO_DIV6_inverted.tif]

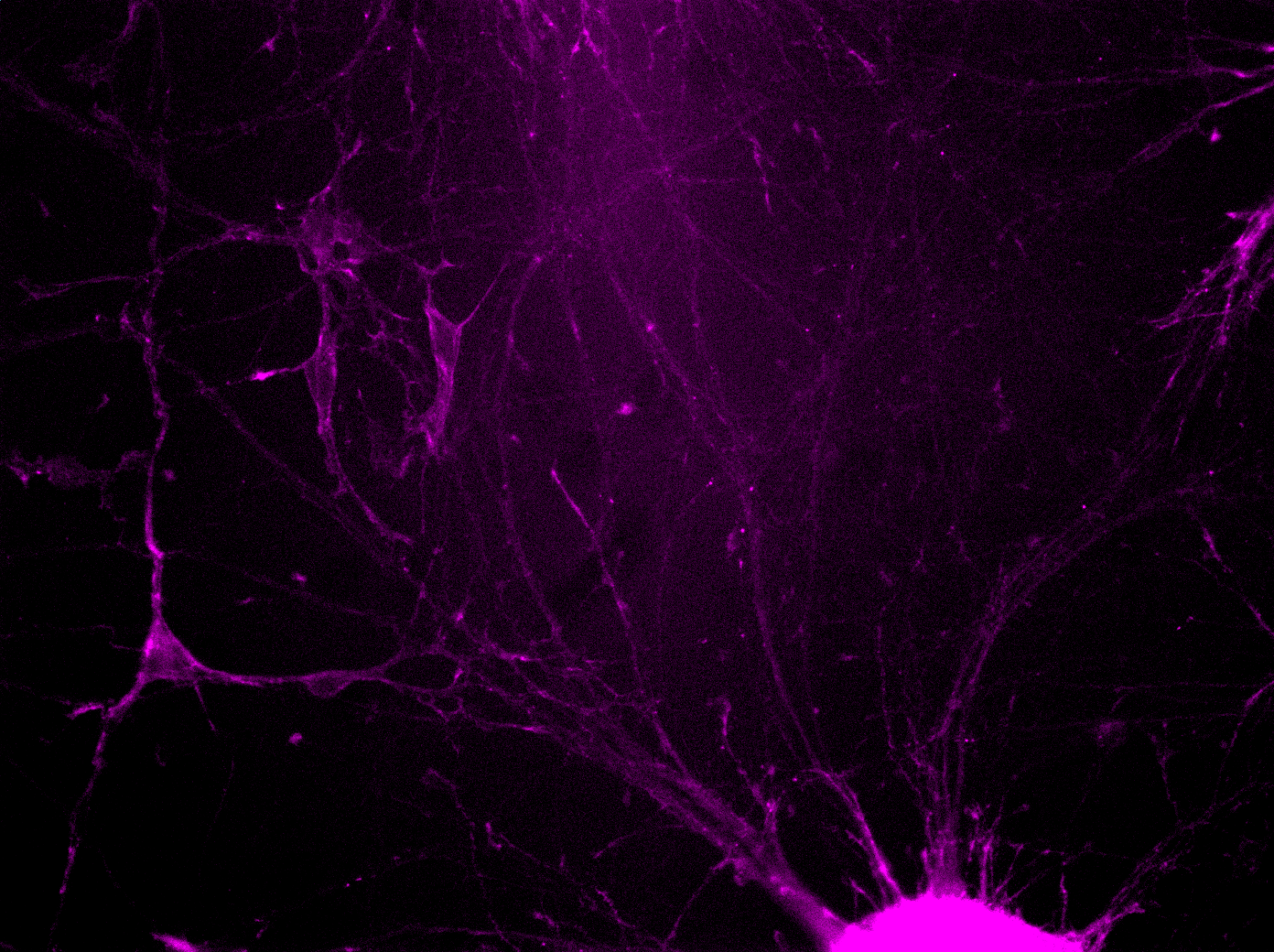

Supplement: Supplementary file 15 — EV Figure Source Data [file 44318_2024_307_MOESM15_ESM.zip › EMBOJ-2024-116734_sourcedataforexpandedviews/Fig EV6/Fig EV6_panelD_Sp-+hetero_DIV9_phalloidin.tif]

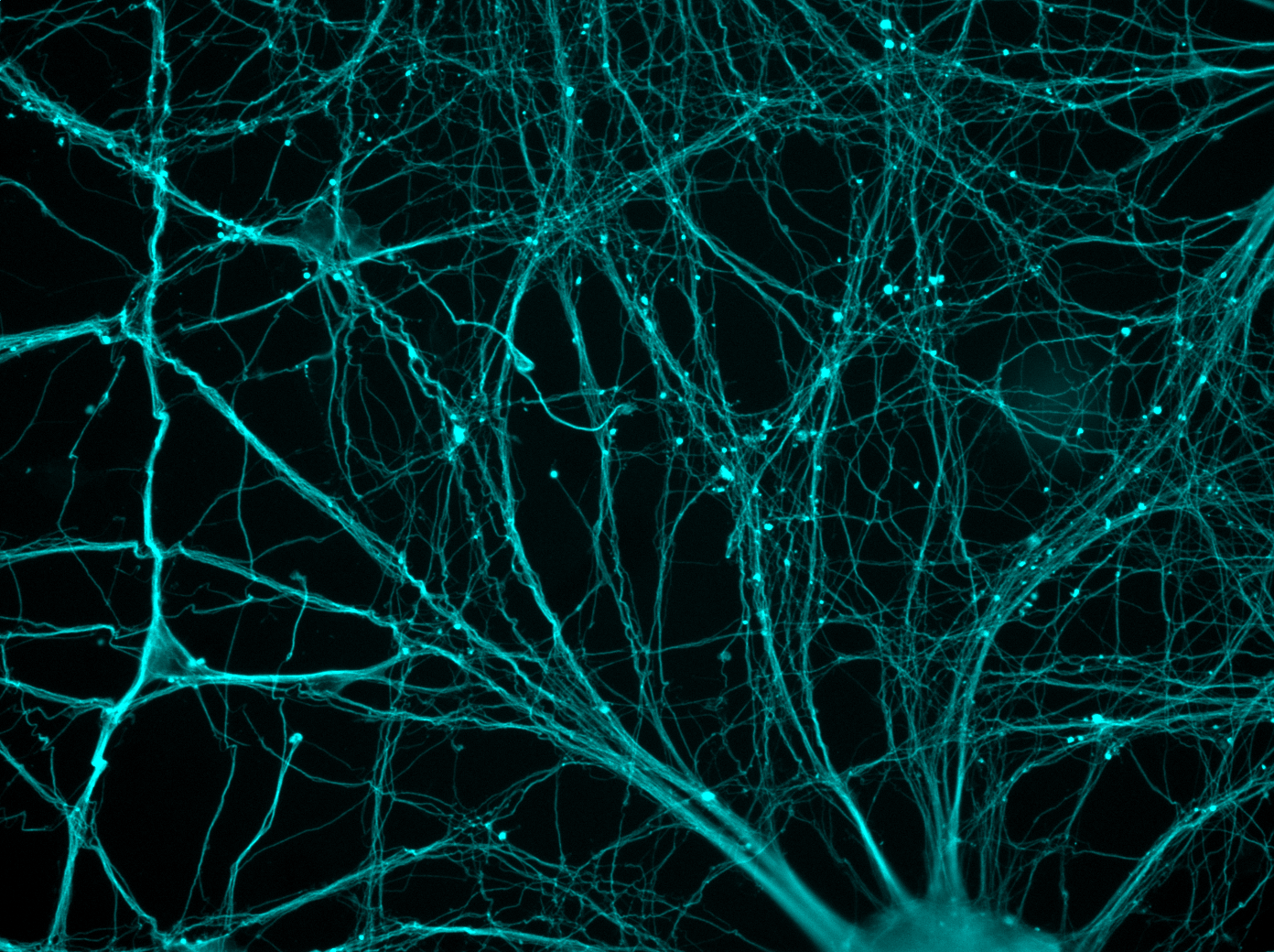

Supplement: Supplementary file 15 — EV Figure Source Data [file 44318_2024_307_MOESM15_ESM.zip › EMBOJ-2024-116734_sourcedataforexpandedviews/Fig EV6/Fig EV6_panelD_Sp-+hetero_DIV9_betaIIItubulin.tif]

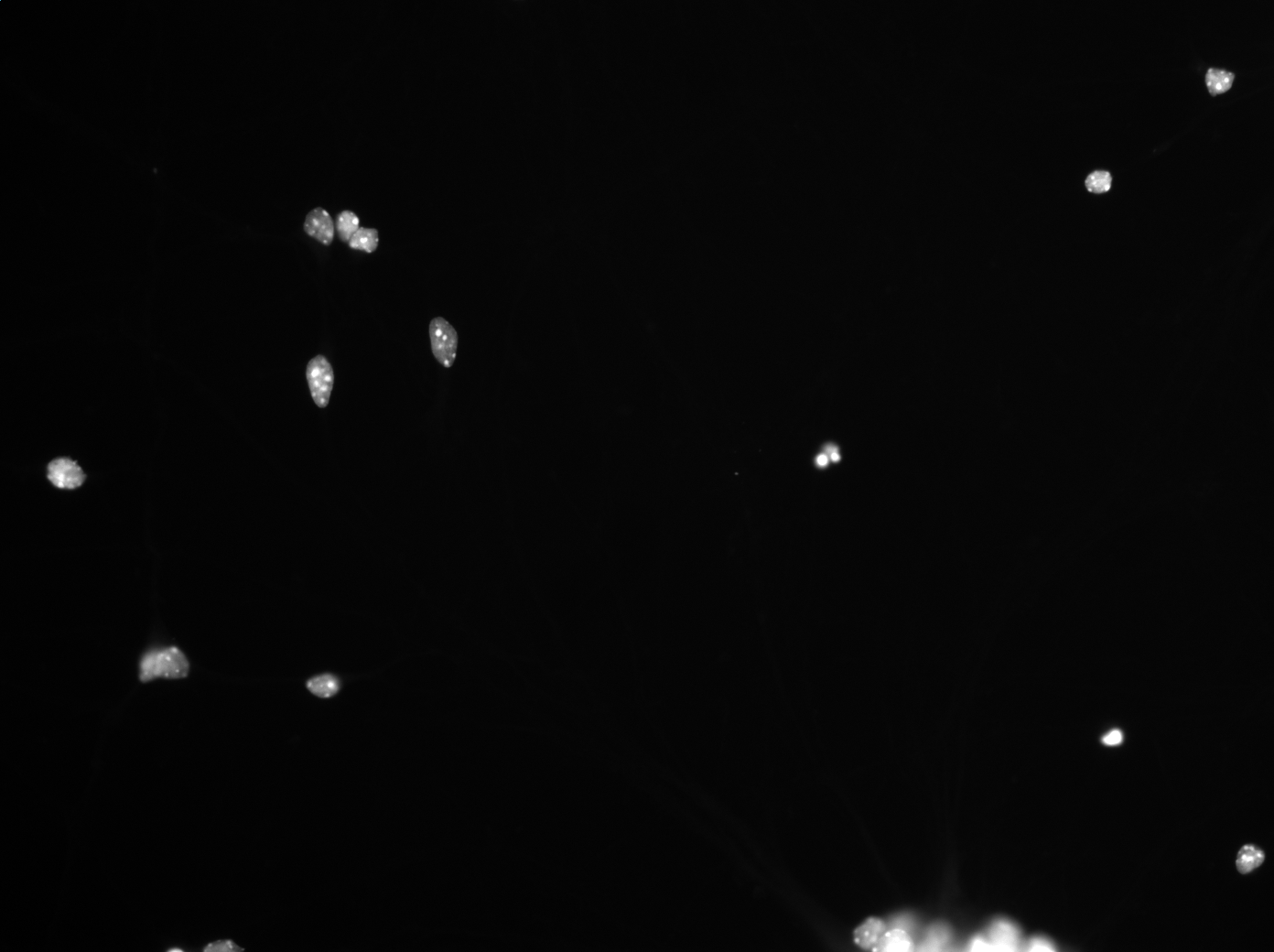

Supplement: Supplementary file 15 — EV Figure Source Data [file 44318_2024_307_MOESM15_ESM.zip › EMBOJ-2024-116734_sourcedataforexpandedviews/Fig EV6/Fig EV6_panelD_Sp-+hetero_DIV9_dapi.tif]

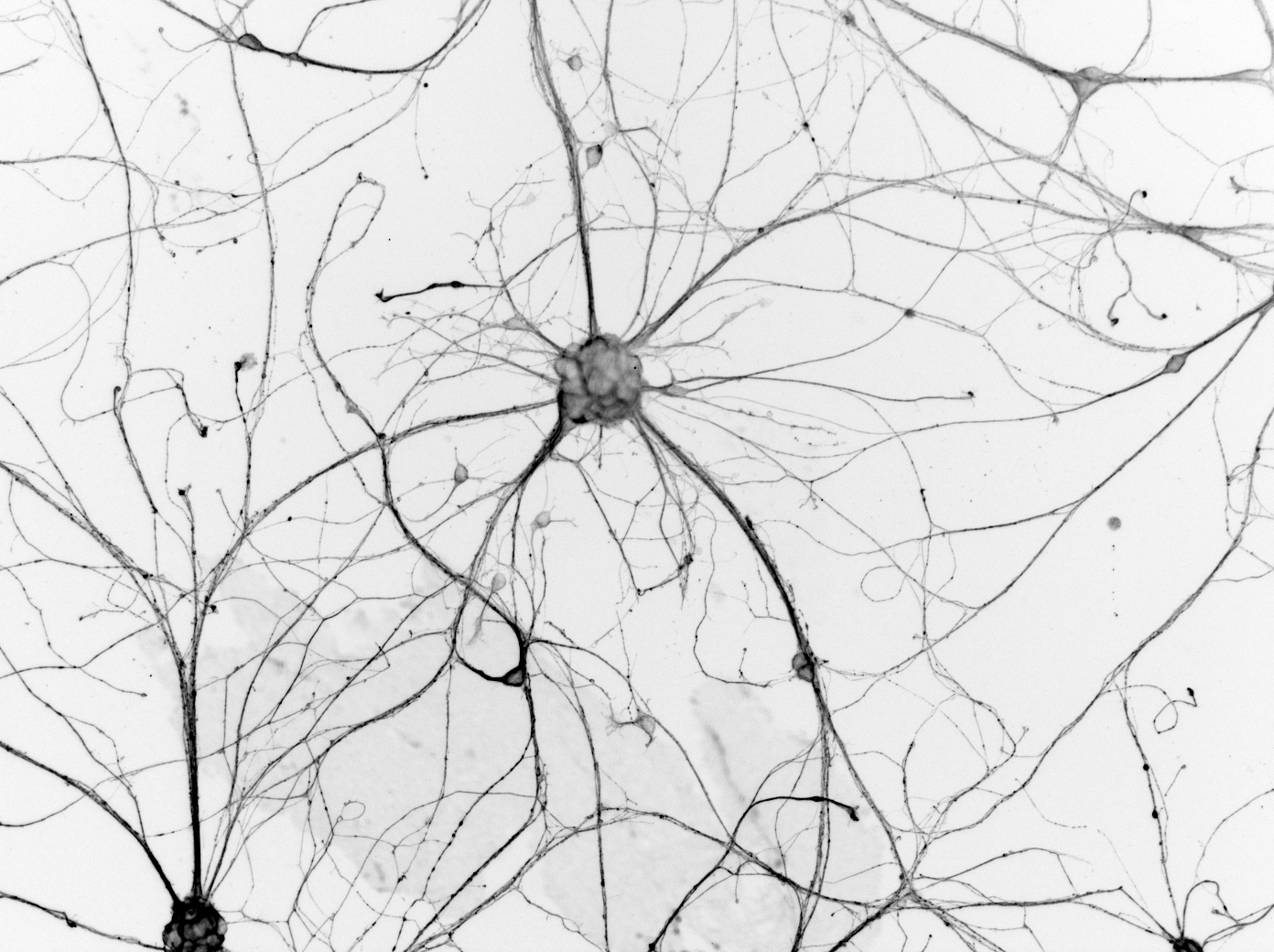

Supplement: Supplementary file 15 — EV Figure Source Data [file 44318_2024_307_MOESM15_ESM.zip › EMBOJ-2024-116734_sourcedataforexpandedviews/Fig EV6/Fig EV6_panelA_Sp-+hetero_DIV6_inverted.tif]

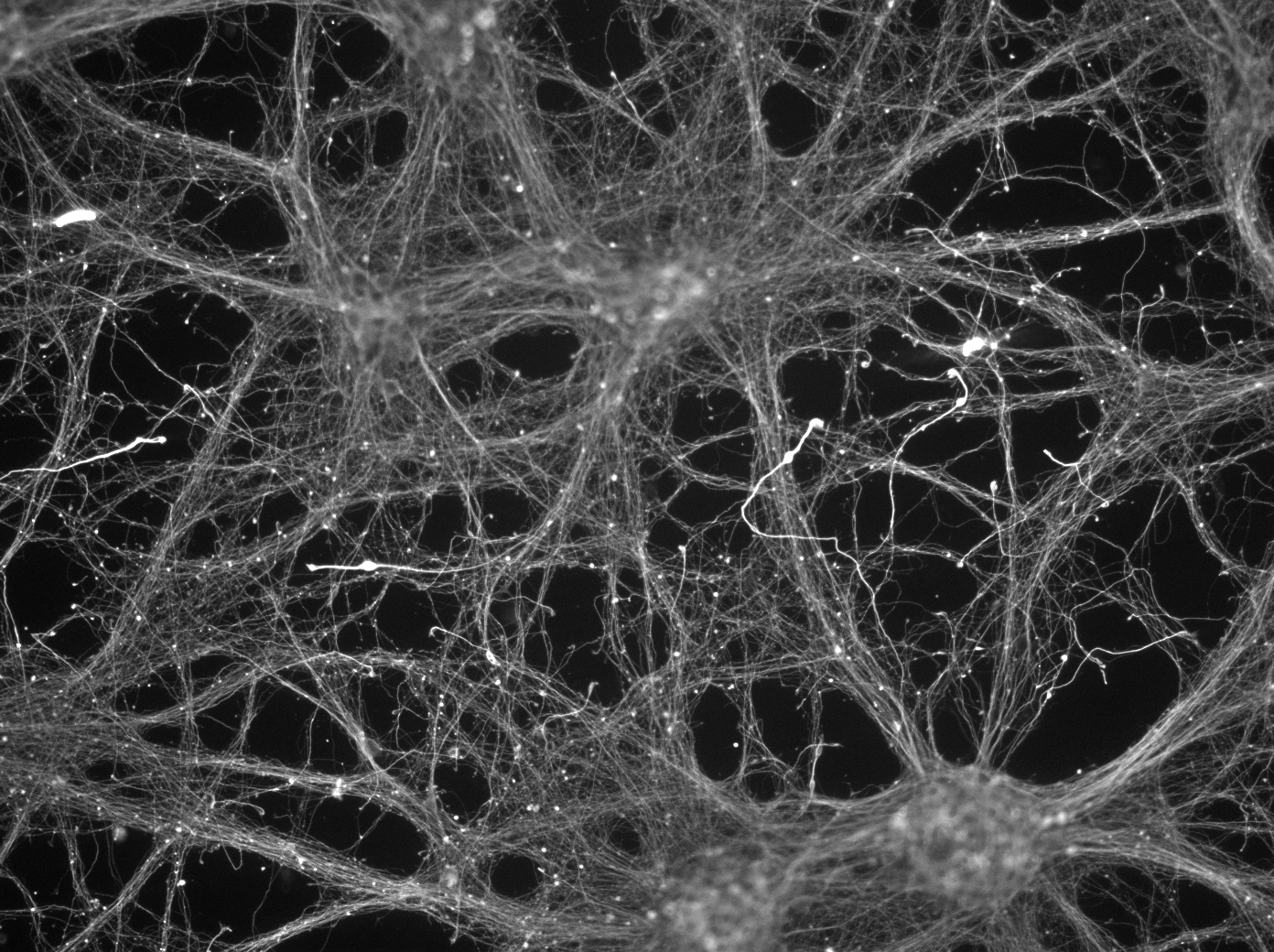

Supplement: Supplementary file 15 — EV Figure Source Data [file 44318_2024_307_MOESM15_ESM.zip › EMBOJ-2024-116734_sourcedataforexpandedviews/Fig EV6/Fig EV6_panelA_Sp-+hetero_DIV9_original.tif]

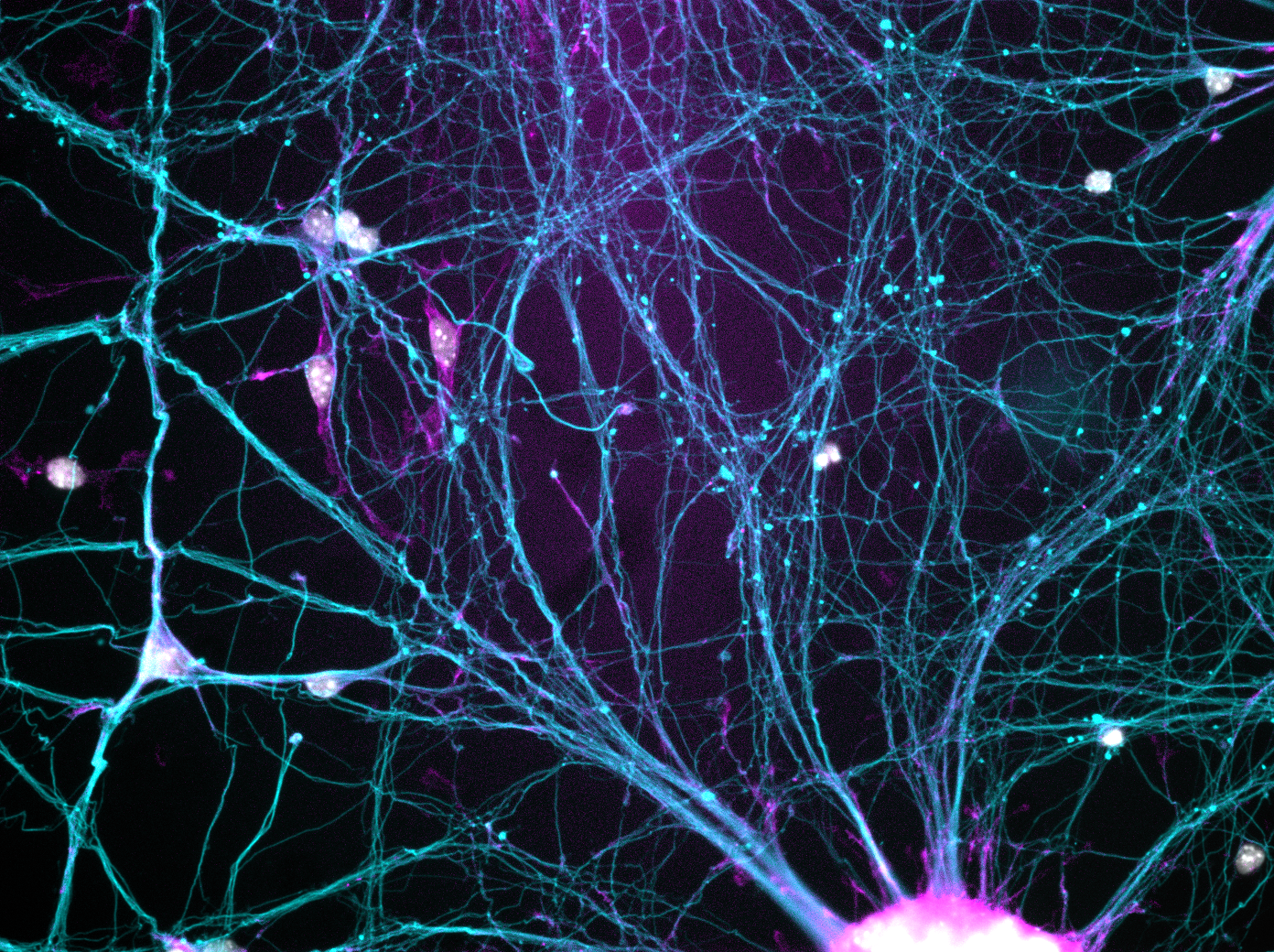

Supplement: Supplementary file 15 — EV Figure Source Data [file 44318_2024_307_MOESM15_ESM.zip › EMBOJ-2024-116734_sourcedataforexpandedviews/Fig EV6/Fig EV6_panelD_Sp-+hetero_DIV9_merge.tif]

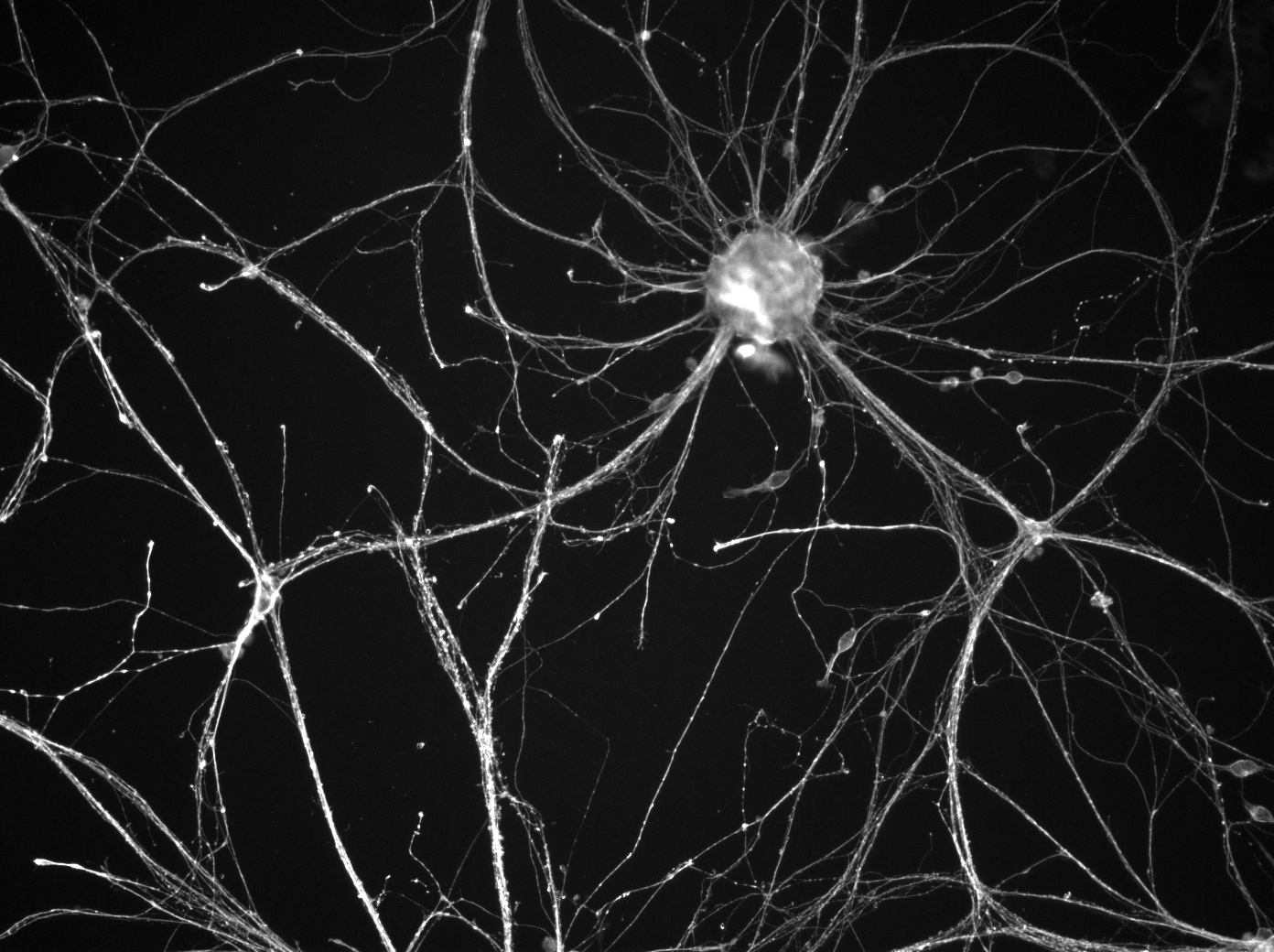

Supplement: Supplementary file 15 — EV Figure Source Data [file 44318_2024_307_MOESM15_ESM.zip › EMBOJ-2024-116734_sourcedataforexpandedviews/Fig EV6/Fig EV6_panelA_Sp++_DIV6_original.tif]

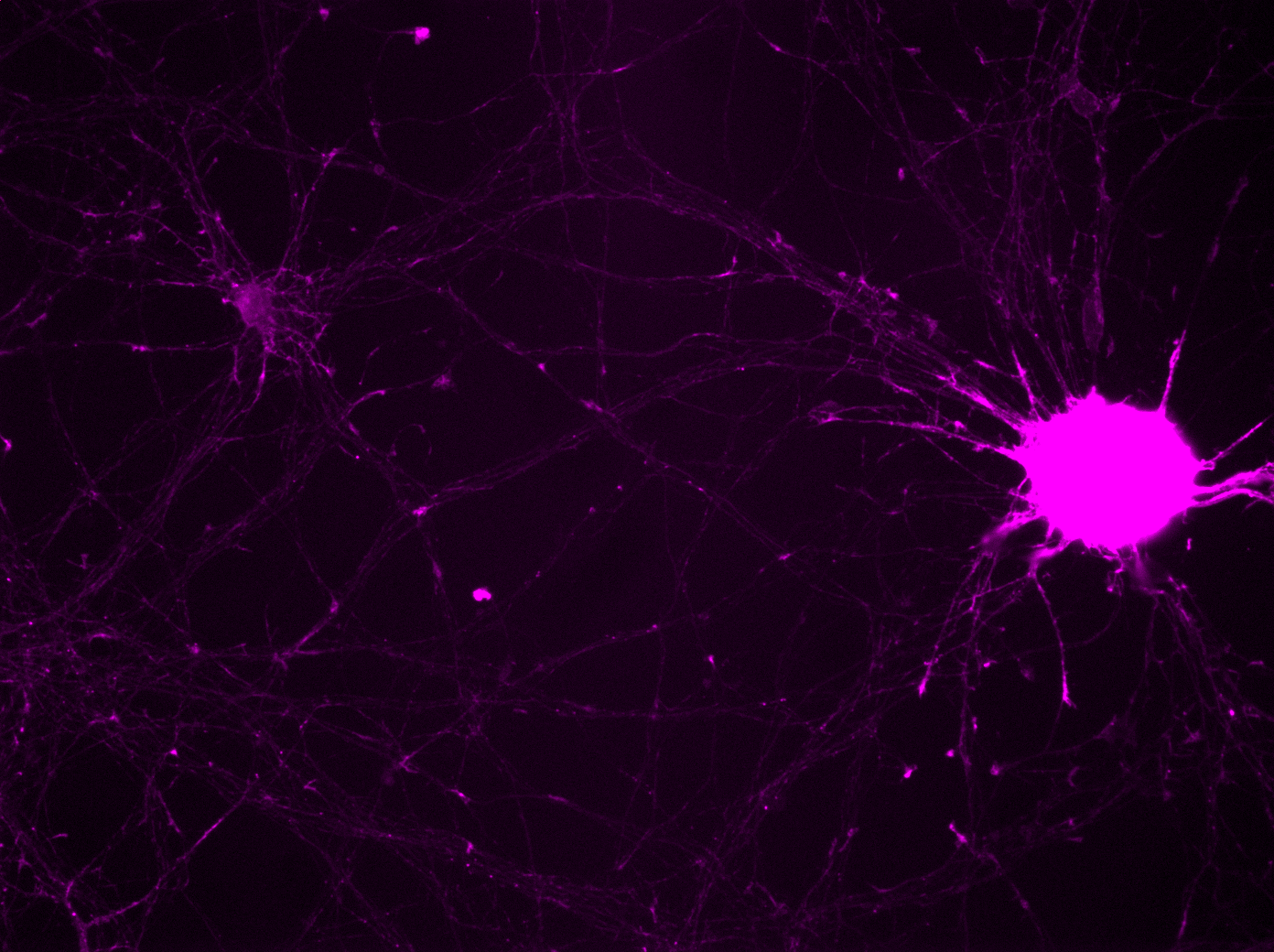

Supplement: Supplementary file 15 — EV Figure Source Data [file 44318_2024_307_MOESM15_ESM.zip › EMBOJ-2024-116734_sourcedataforexpandedviews/Fig EV6/Fig EV6_panelD_Sp--KO_DIV9_phalloidin.tif]

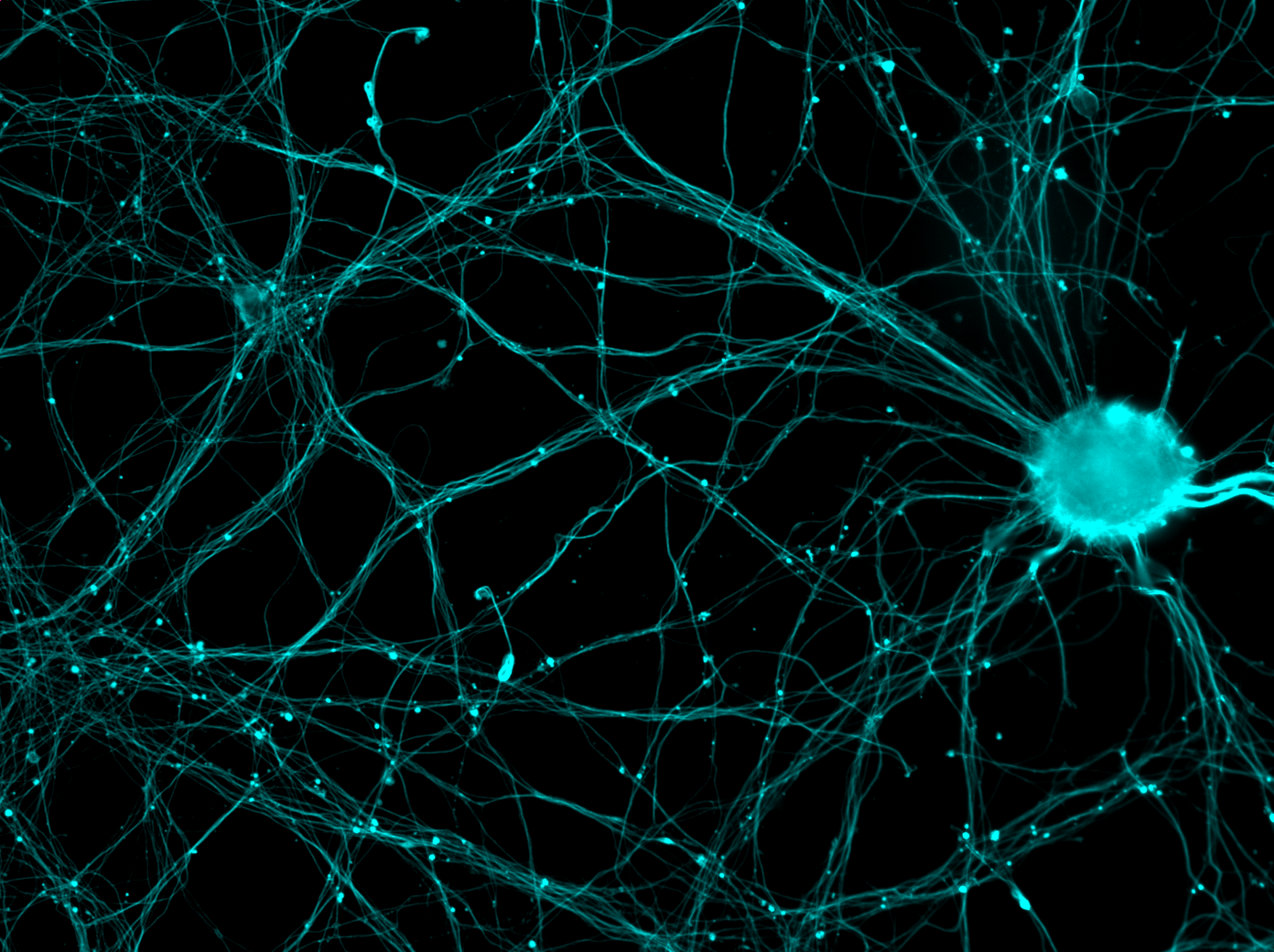

Supplement: Supplementary file 15 — EV Figure Source Data [file 44318_2024_307_MOESM15_ESM.zip › EMBOJ-2024-116734_sourcedataforexpandedviews/Fig EV6/Fig EV6_panelD_Sp--KO_DIV9_betaIIItubulin.tif]

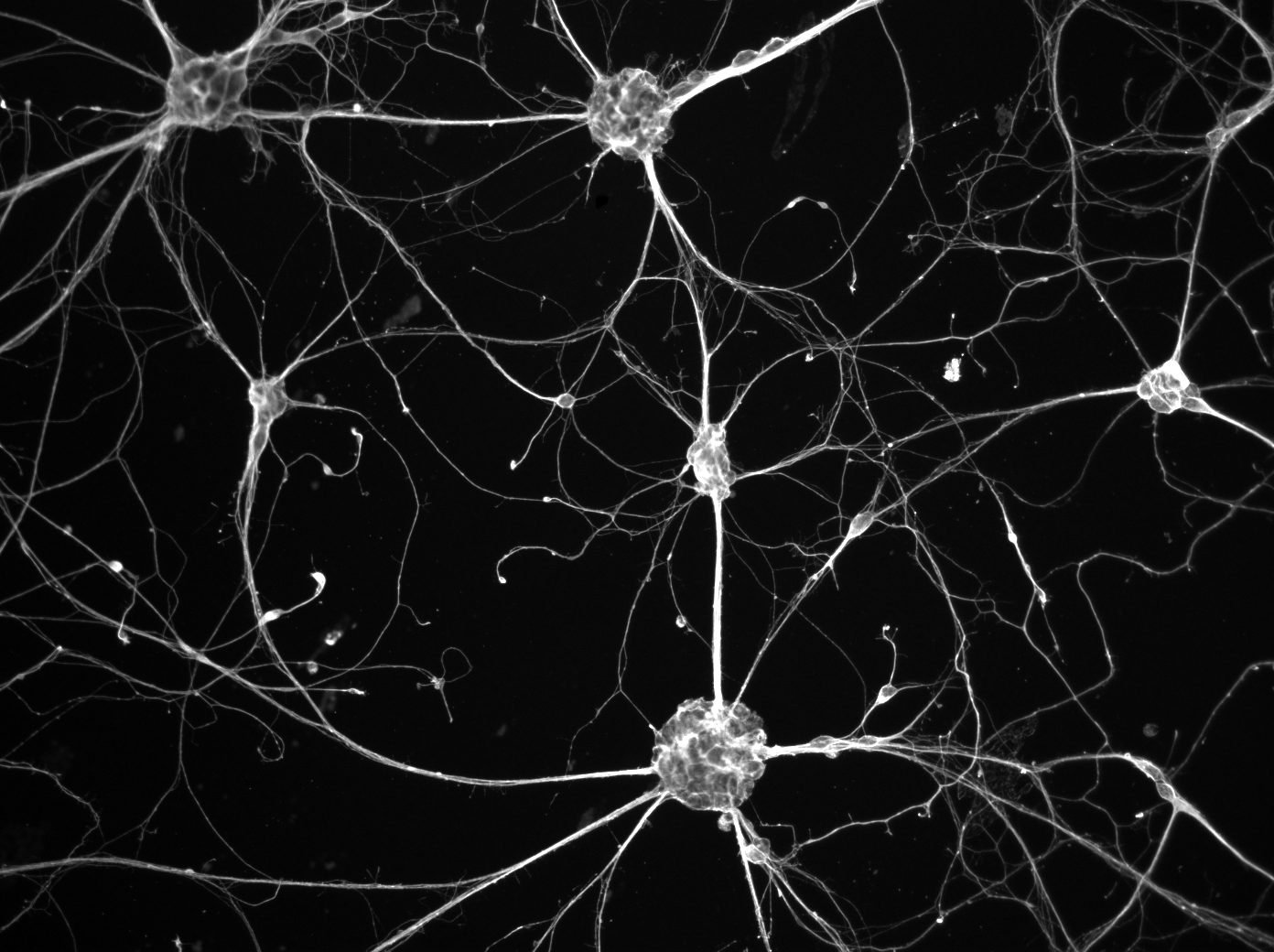

Supplement: Supplementary file 15 — EV Figure Source Data [file 44318_2024_307_MOESM15_ESM.zip › EMBOJ-2024-116734_sourcedataforexpandedviews/Fig EV6/Fig EV6_panelA_Sp--KO_DIV6_original.tif]

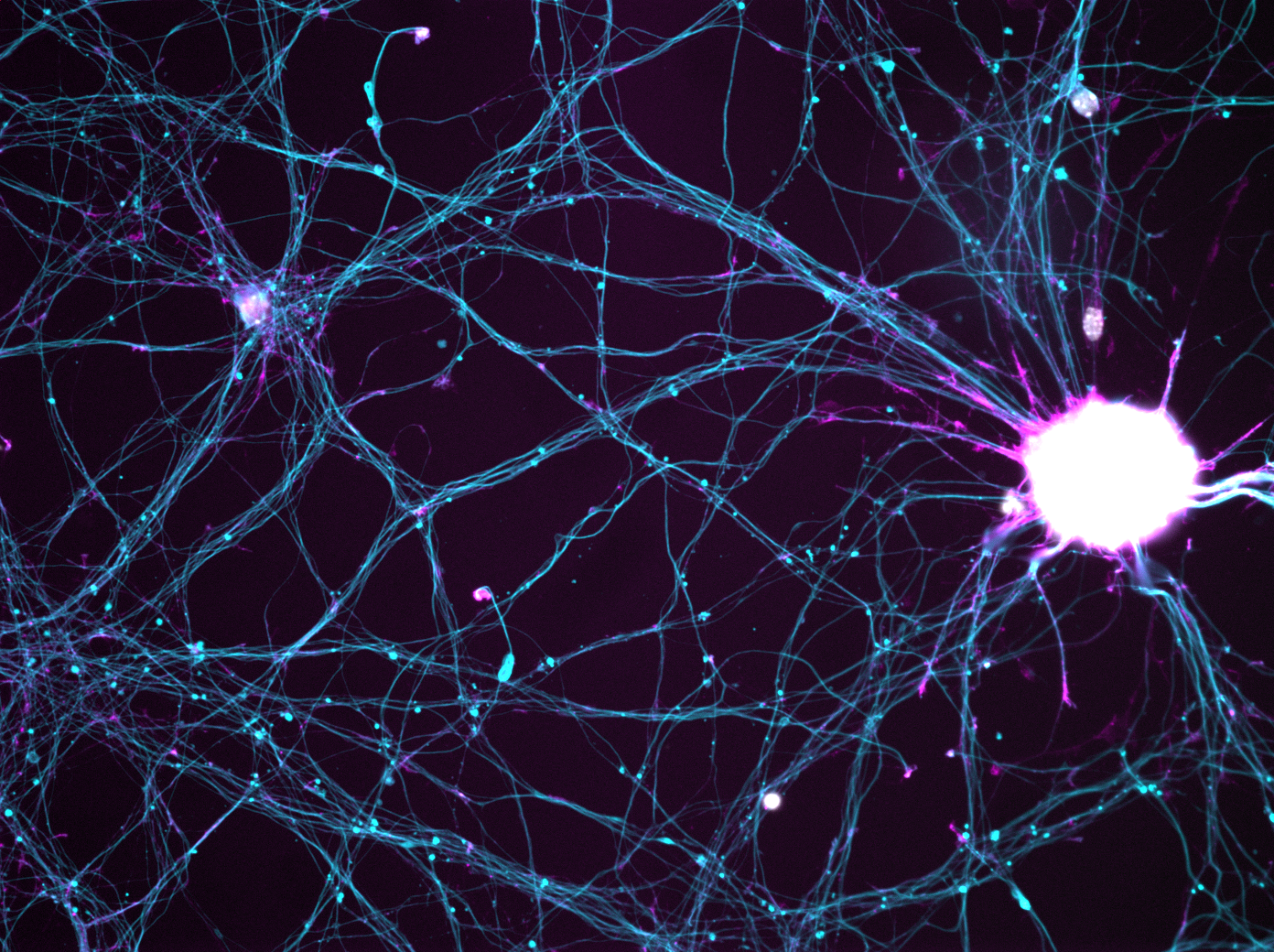

Supplement: Supplementary file 15 — EV Figure Source Data [file 44318_2024_307_MOESM15_ESM.zip › EMBOJ-2024-116734_sourcedataforexpandedviews/Fig EV6/Fig EV6_panelD_Sp--KO_DIV9_merge.tif]

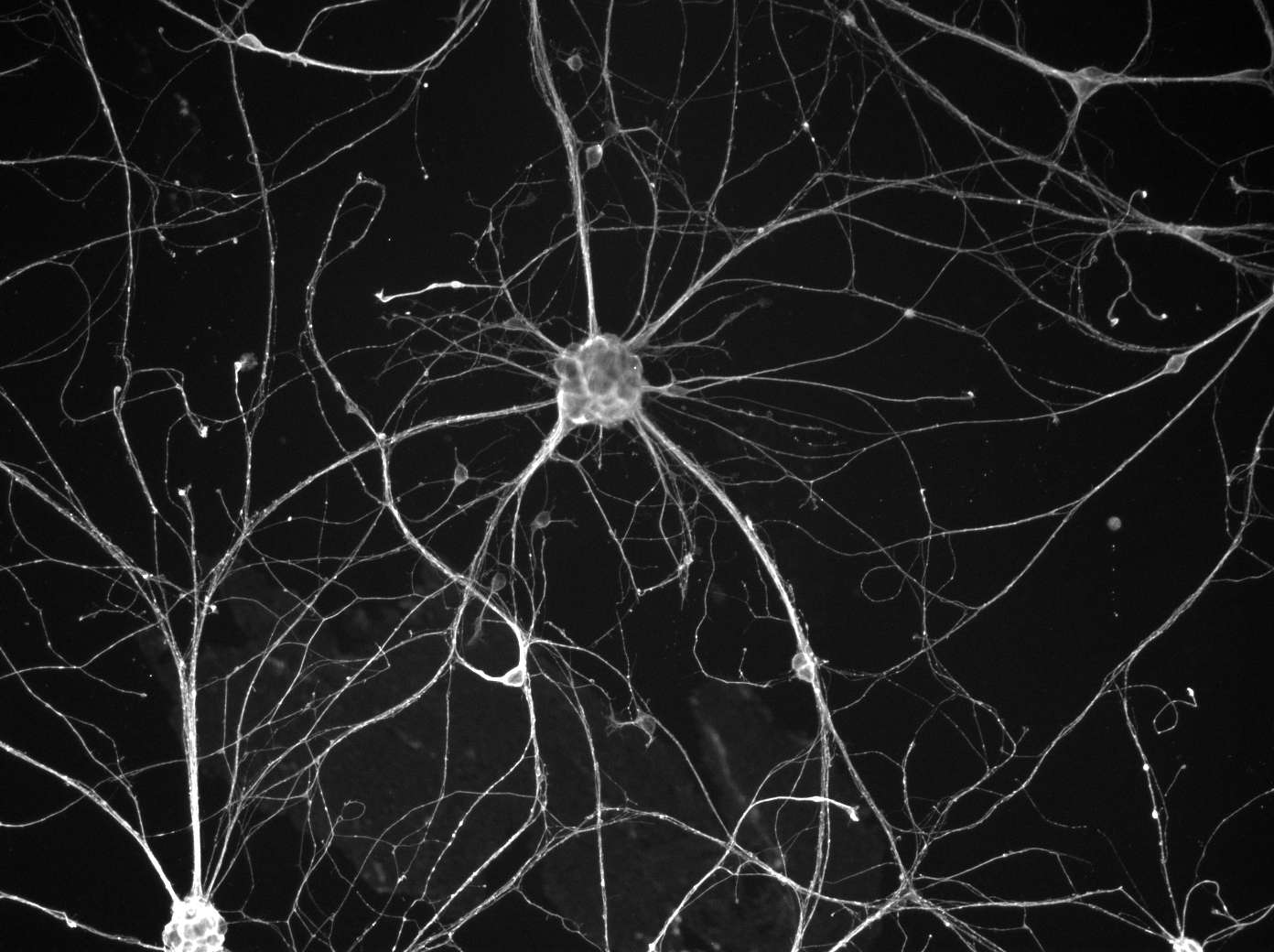

Supplement: Supplementary file 15 — EV Figure Source Data [file 44318_2024_307_MOESM15_ESM.zip › EMBOJ-2024-116734_sourcedataforexpandedviews/Fig EV6/Fig EV6_panelA_Sp-+hetero_DIV6_original.tif]

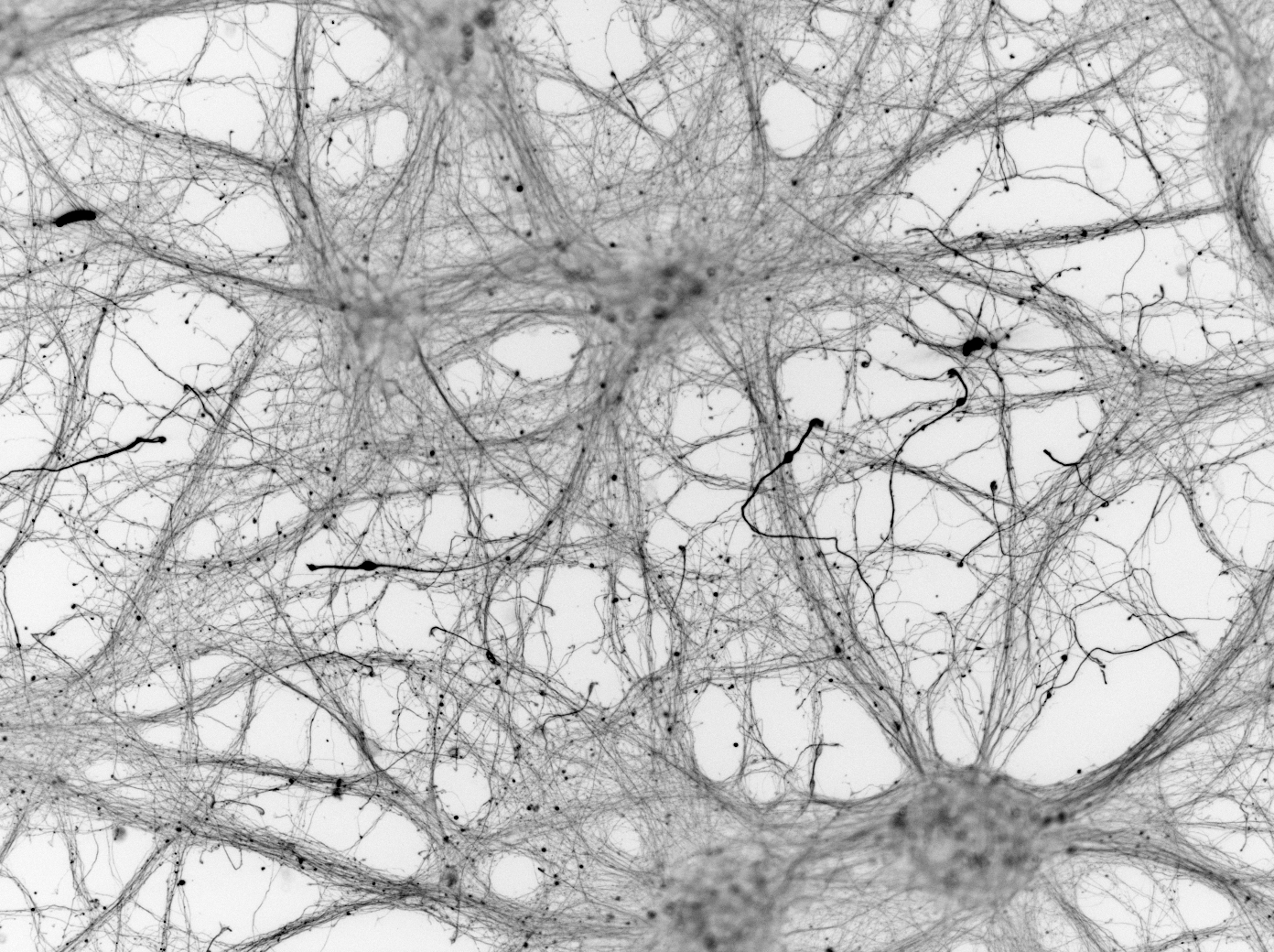

Supplement: Supplementary file 15 — EV Figure Source Data [file 44318_2024_307_MOESM15_ESM.zip › EMBOJ-2024-116734_sourcedataforexpandedviews/Fig EV6/Fig EV6_panelA_Sp-+hetero_DIV9_inverted.tif]

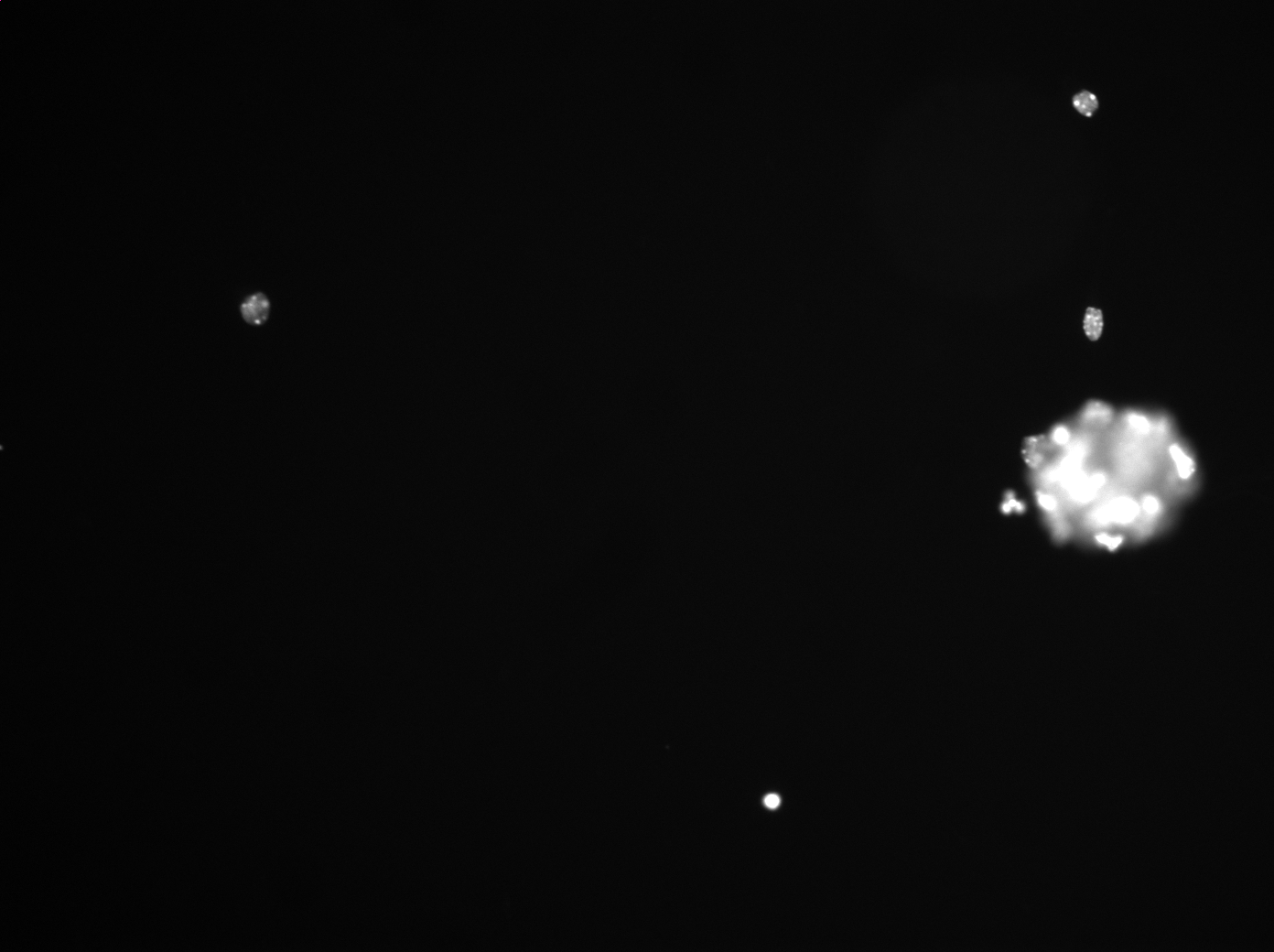

Supplement: Supplementary file 15 — EV Figure Source Data [file 44318_2024_307_MOESM15_ESM.zip › EMBOJ-2024-116734_sourcedataforexpandedviews/Fig EV6/Fig EV6_panelD_Sp--KO_DIV9_Dapi.tif]

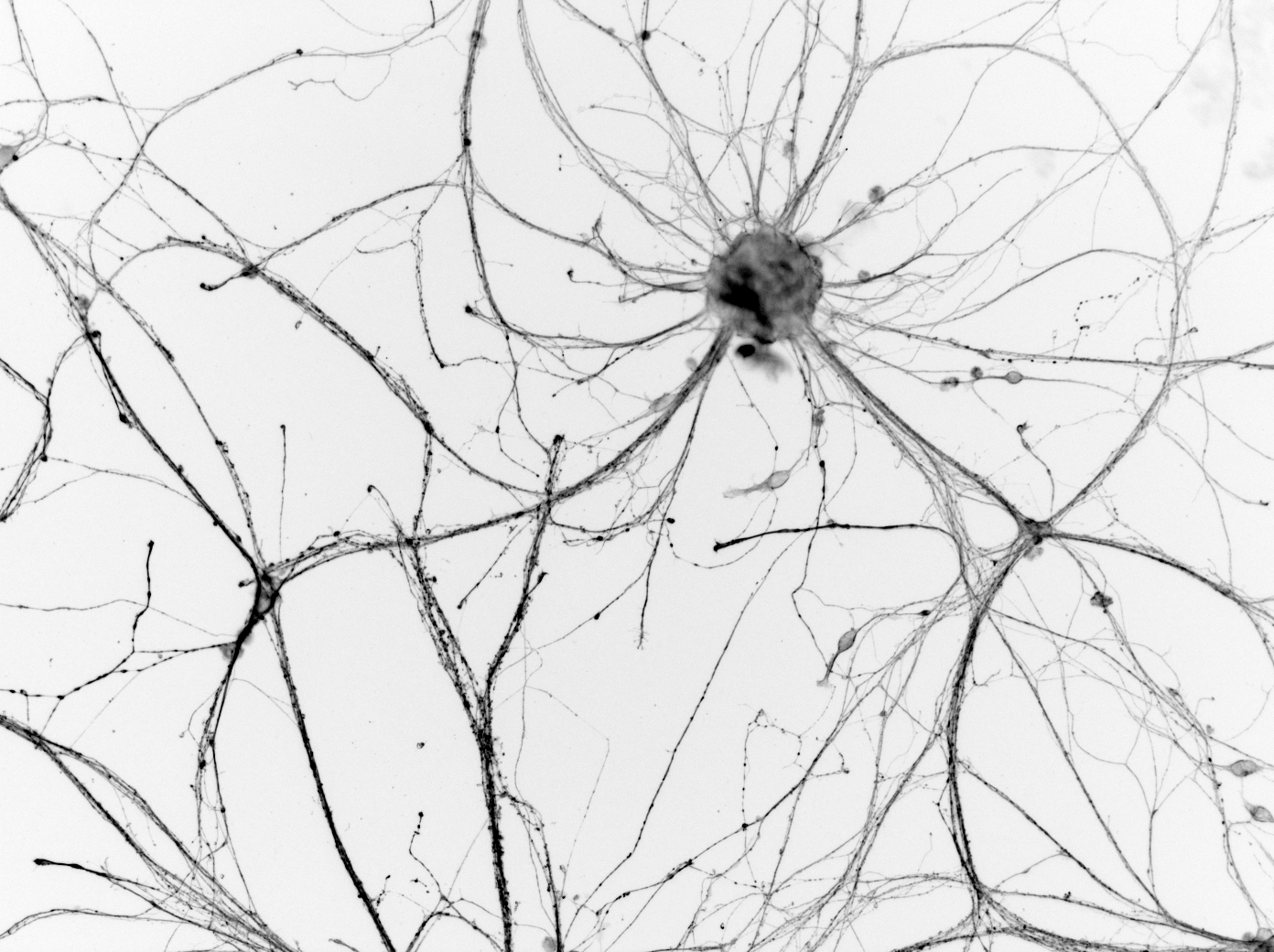

Supplement: Supplementary file 15 — EV Figure Source Data [file 44318_2024_307_MOESM15_ESM.zip › EMBOJ-2024-116734_sourcedataforexpandedviews/Fig EV6/Fig EV6_panelA_Sp++_DIV6_inverted.tif]

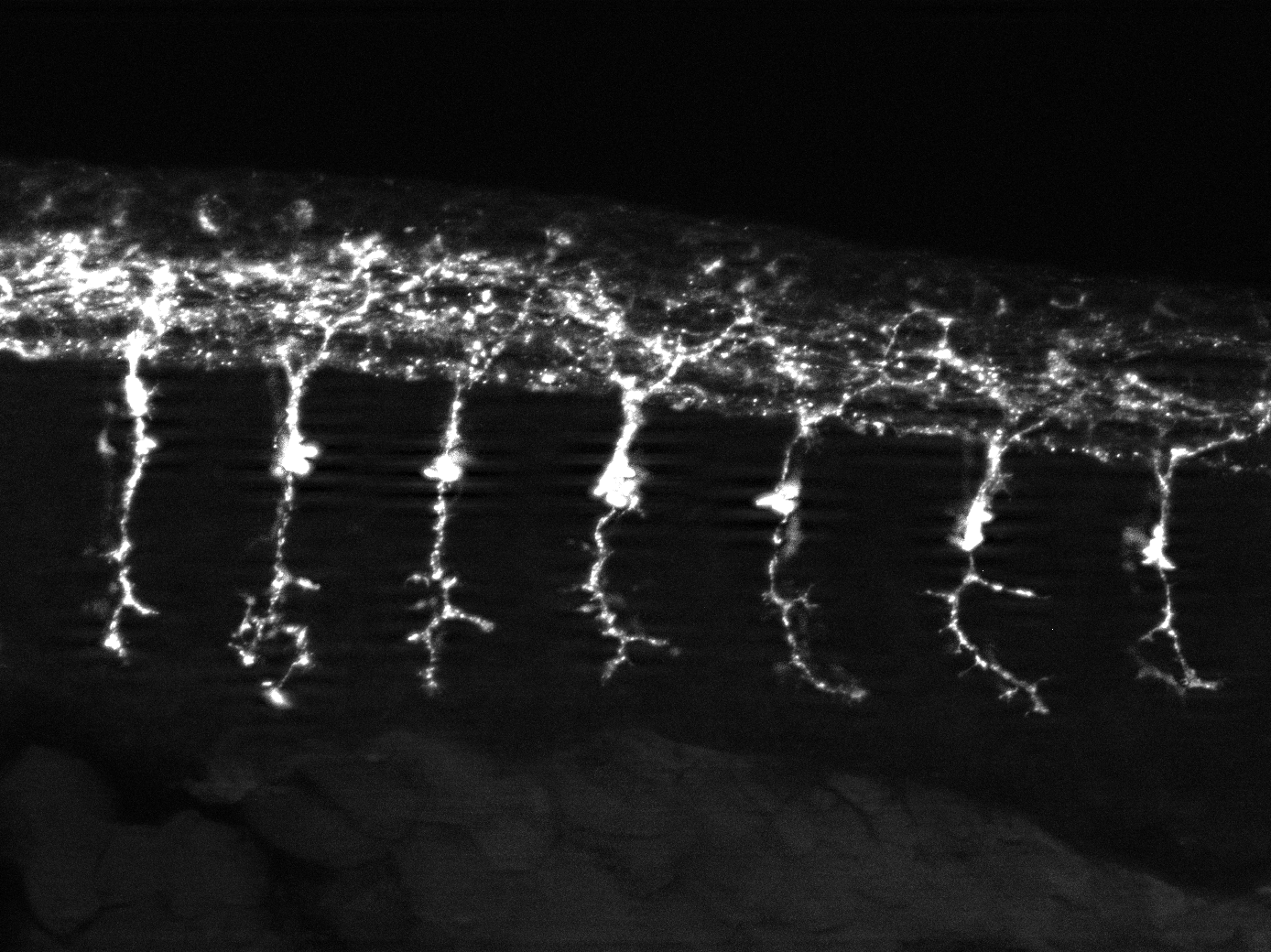

Supplement: Supplementary file 15 — EV Figure Source Data [file 44318_2024_307_MOESM15_ESM.zip › EMBOJ-2024-116734_sourcedataforexpandedviews/Fig EV5/Fig.EV5F_MOKat1.3_pmn_znp1.tif]

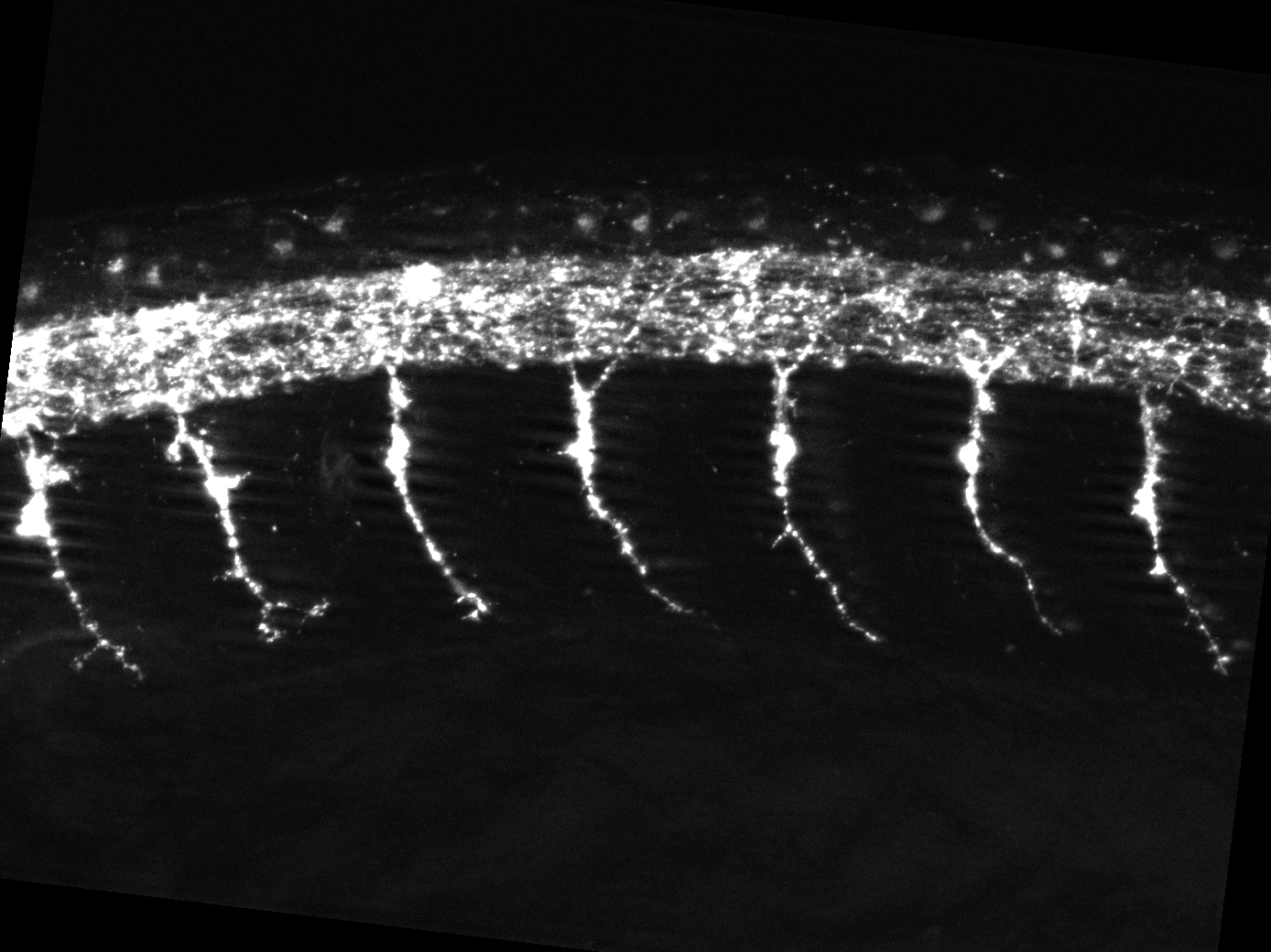

Supplement: Supplementary file 15 — EV Figure Source Data [file 44318_2024_307_MOESM15_ESM.zip › EMBOJ-2024-116734_sourcedataforexpandedviews/Fig EV5/Fig.EV5C_MOTTLL6andTTLL6mRNA_pmn.tif]

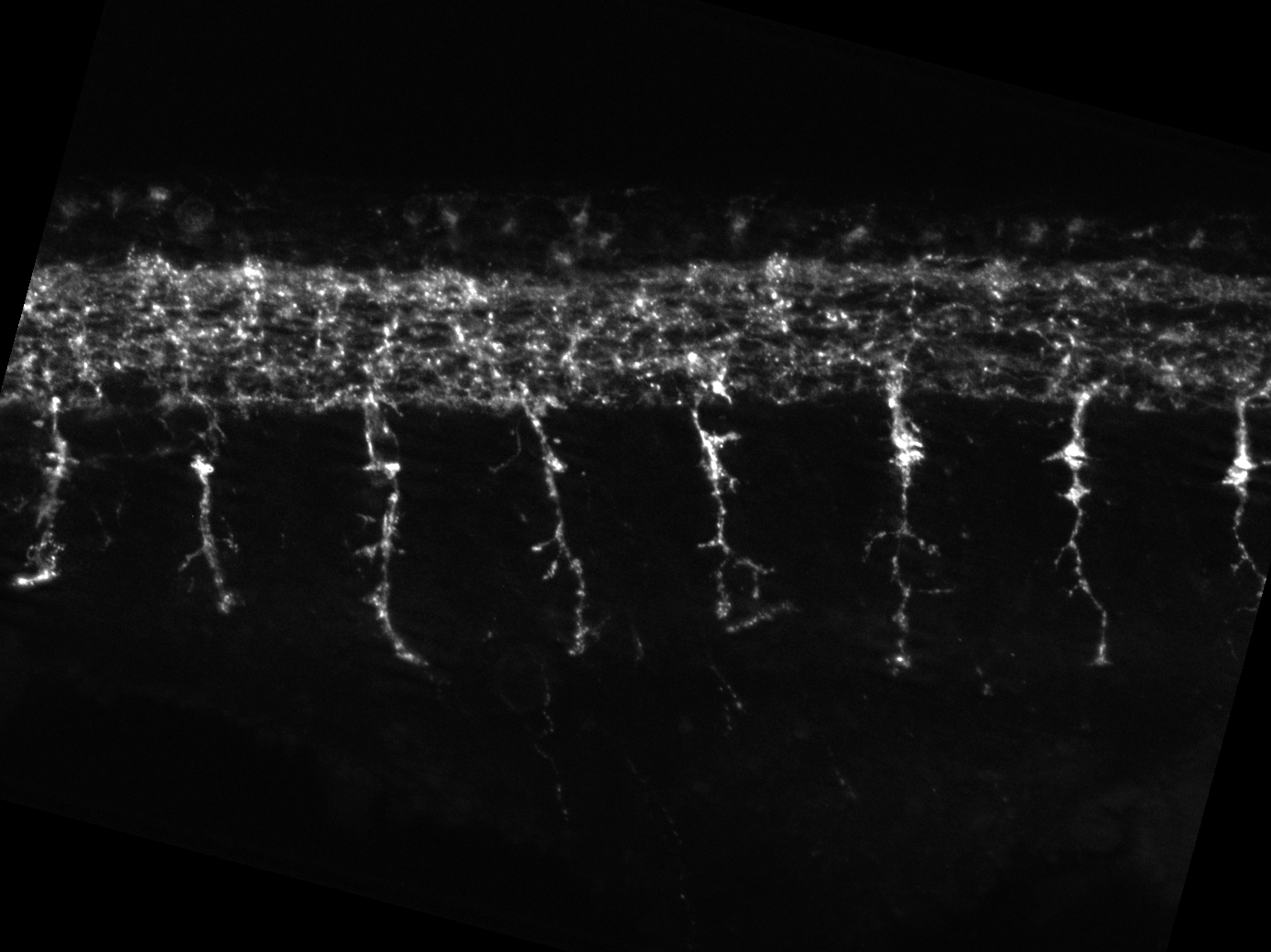

Supplement: Supplementary file 15 — EV Figure Source Data [file 44318_2024_307_MOESM15_ESM.zip › EMBOJ-2024-116734_sourcedataforexpandedviews/Fig EV5/Fig.EV5C_MOTTLL6pmn.tif]

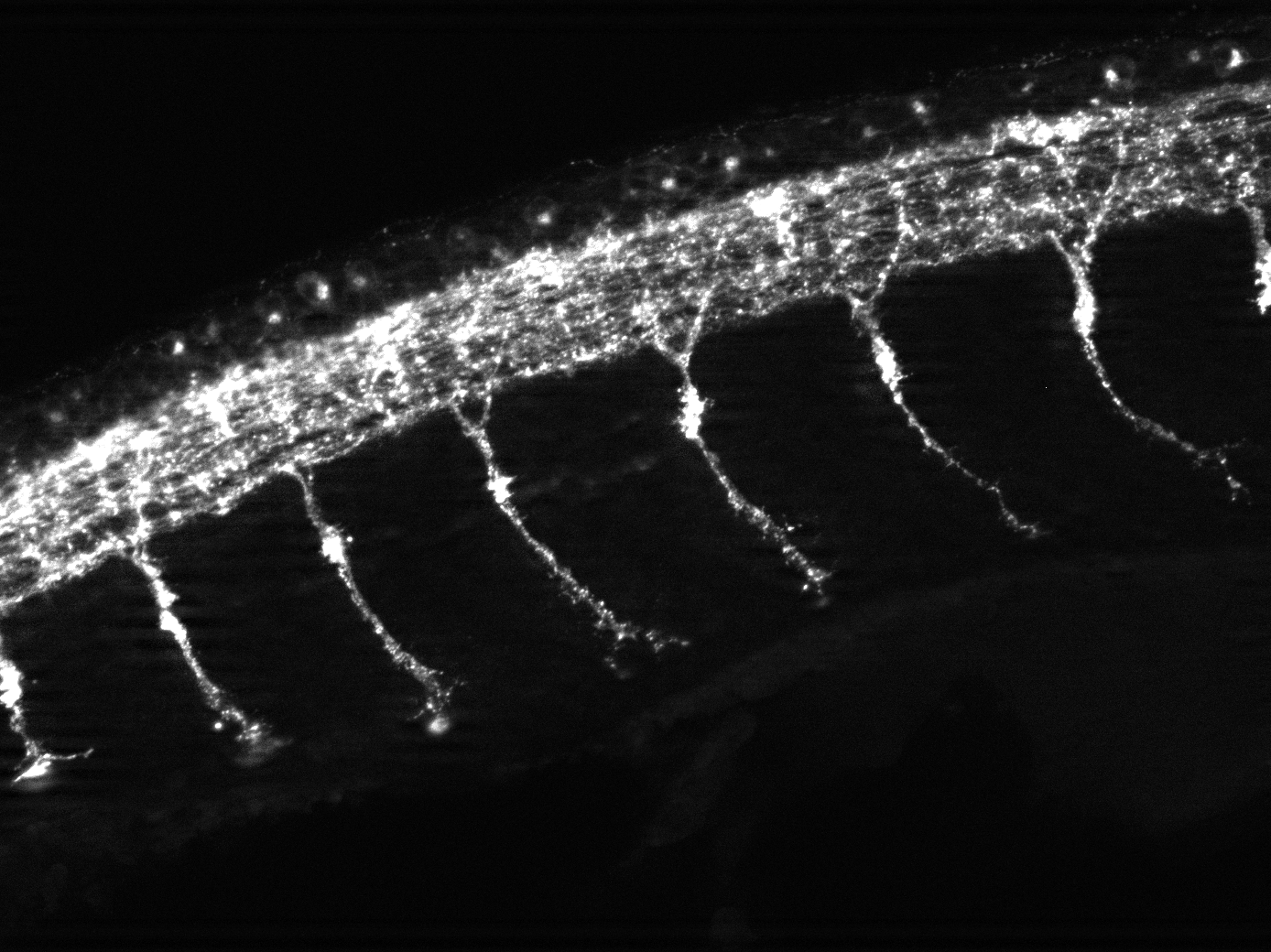

Supplement: Supplementary file 15 — EV Figure Source Data [file 44318_2024_307_MOESM15_ESM.zip › EMBOJ-2024-116734_sourcedataforexpandedviews/Fig EV5/Fig.EV5F_MOCTL_pmn_znp1.tif]

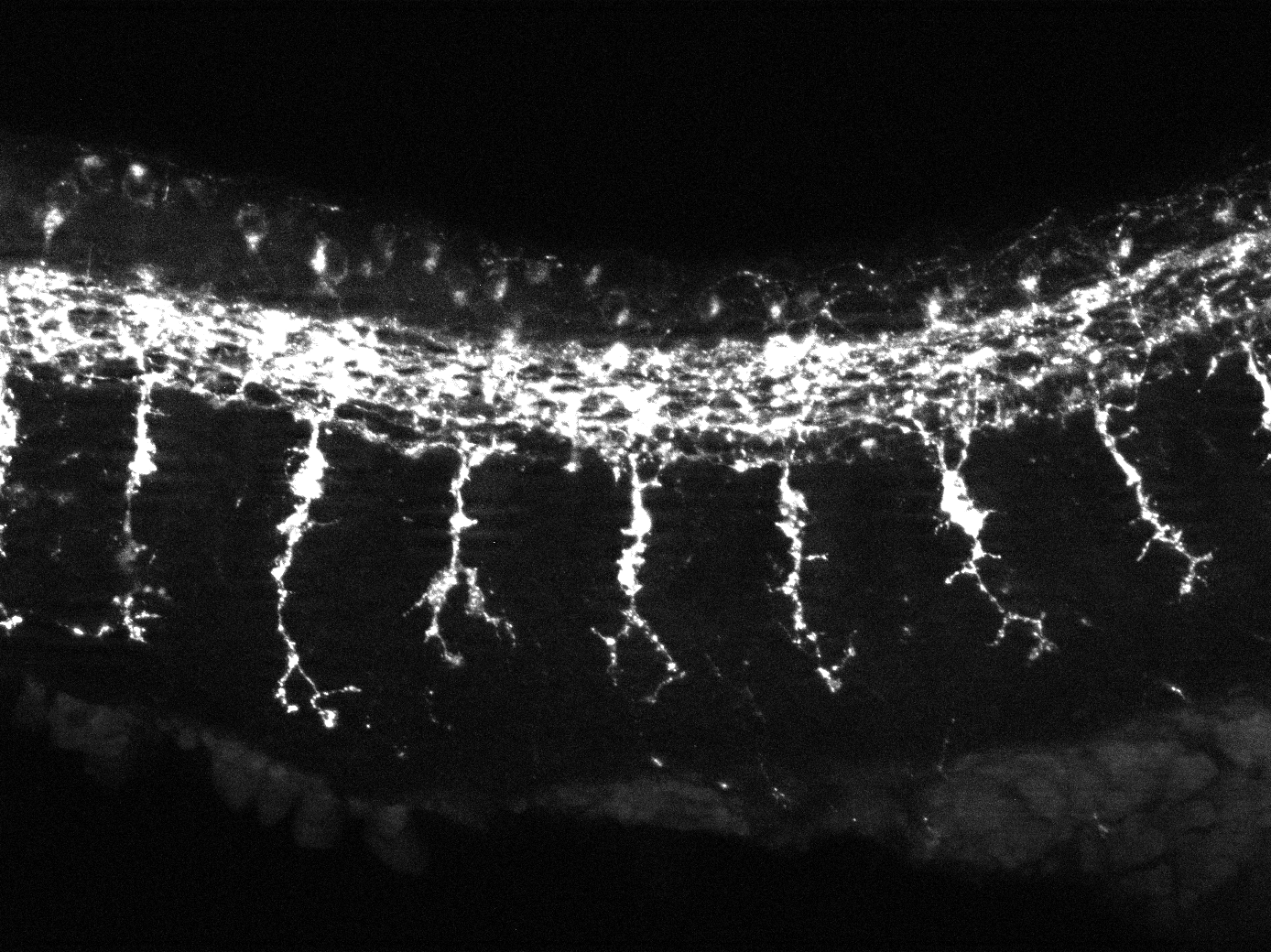

Supplement: Supplementary file 15 — EV Figure Source Data [file 44318_2024_307_MOESM15_ESM.zip › EMBOJ-2024-116734_sourcedataforexpandedviews/Fig EV5/Fig.EV5C_MOTTLL6andTTLL11mRNA_pmn.tif]

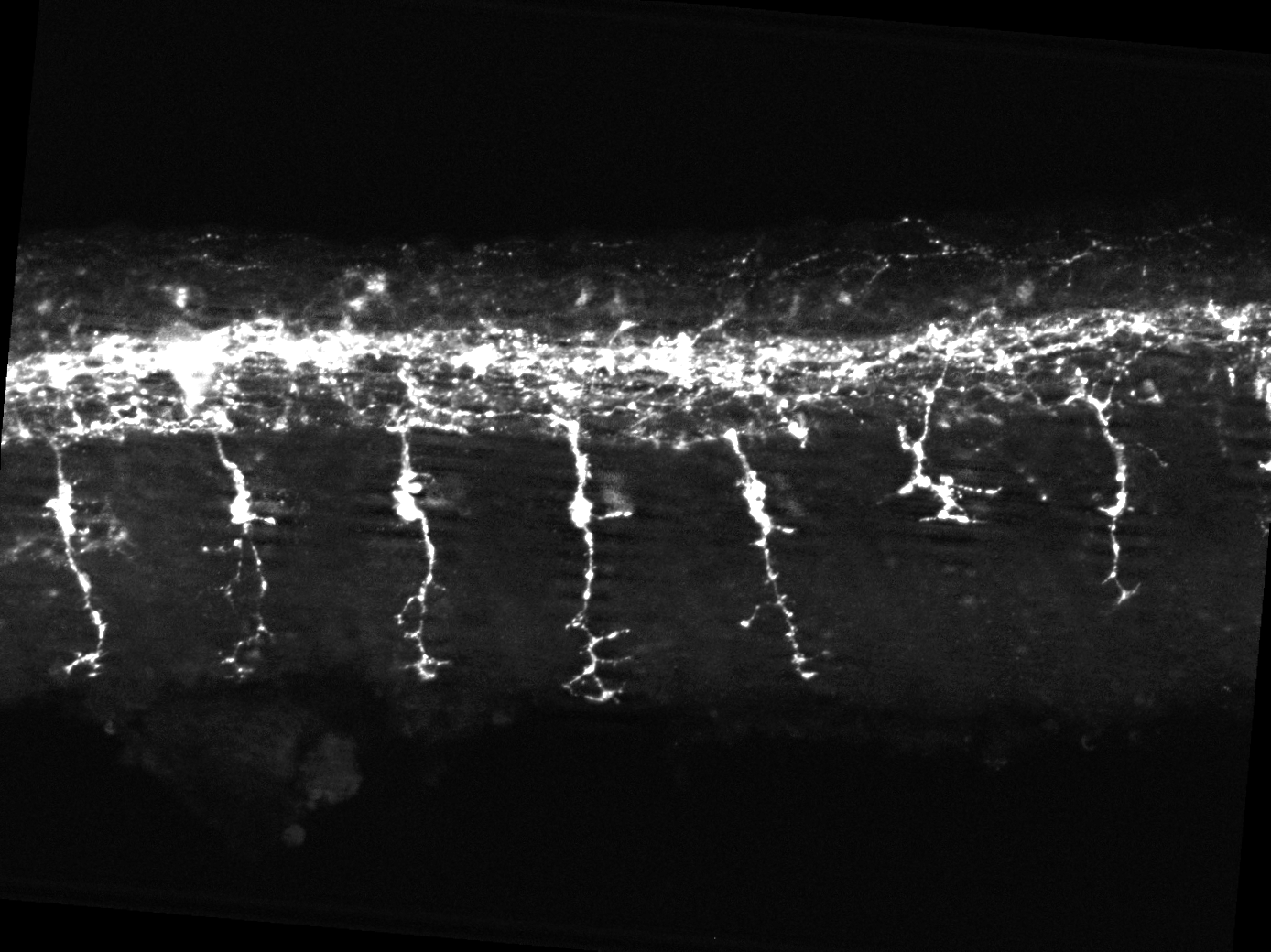

Supplement: Supplementary file 15 — EV Figure Source Data [file 44318_2024_307_MOESM15_ESM.zip › EMBOJ-2024-116734_sourcedataforexpandedviews/Fig EV5/Fig.EVC_MOTTLL11andTTLL6mRNA_pmn.tif]

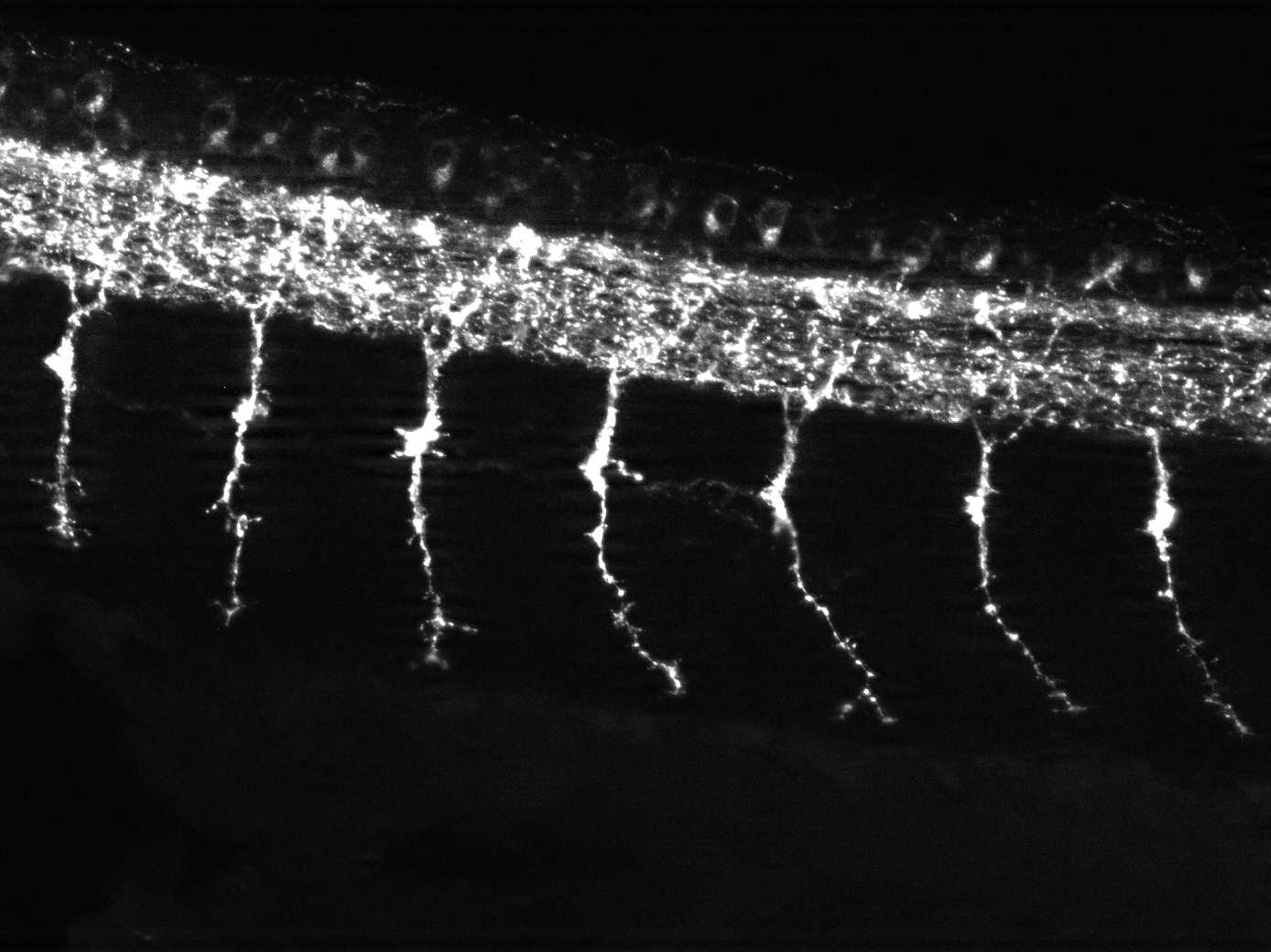

Supplement: Supplementary file 15 — EV Figure Source Data [file 44318_2024_307_MOESM15_ESM.zip › EMBOJ-2024-116734_sourcedataforexpandedviews/Fig EV5/Fig.EV5F_MOKat1.3 and TTLL6mRNA_pmn_znp1.tif]

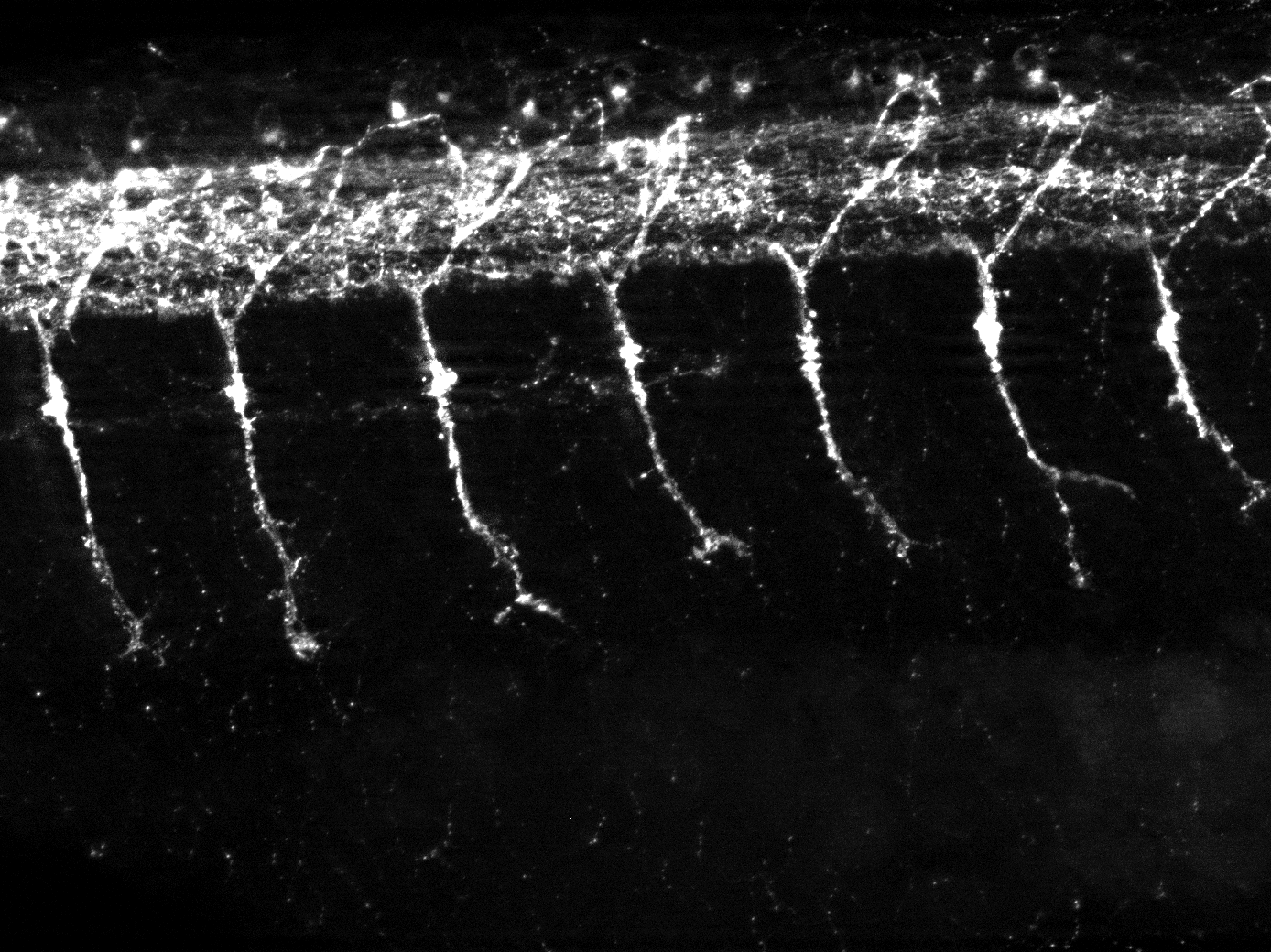

Supplement: Supplementary file 15 — EV Figure Source Data [file 44318_2024_307_MOESM15_ESM.zip › EMBOJ-2024-116734_sourcedataforexpandedviews/Fig EV5/Fig.EV5A_MOKat1.3 and KATNA1mRNA_pmn_znp1.tif]

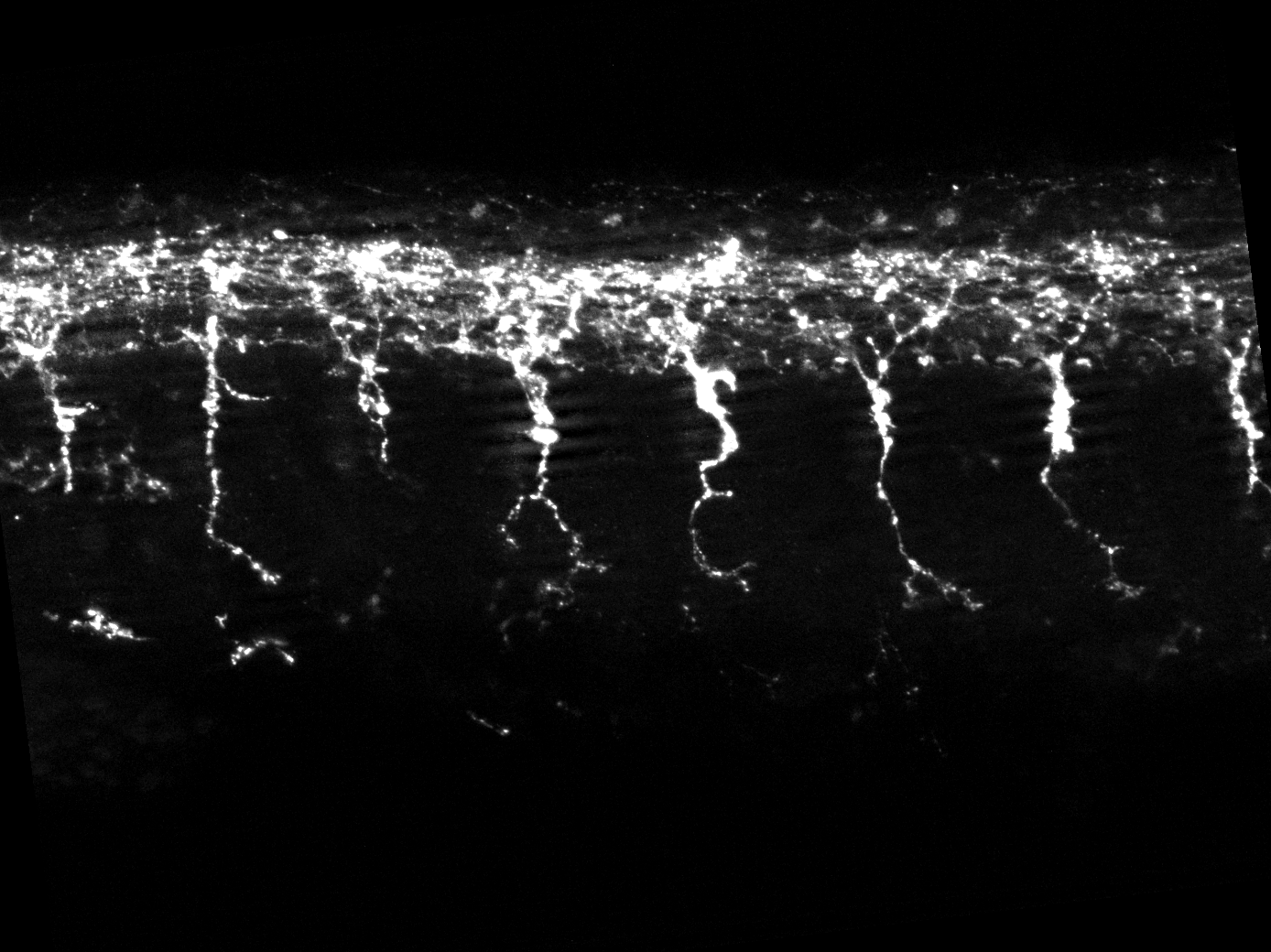

Supplement: Supplementary file 15 — EV Figure Source Data [file 44318_2024_307_MOESM15_ESM.zip › EMBOJ-2024-116734_sourcedataforexpandedviews/Fig EV5/Fig.EVC_MOTTLL11pmn.tif]

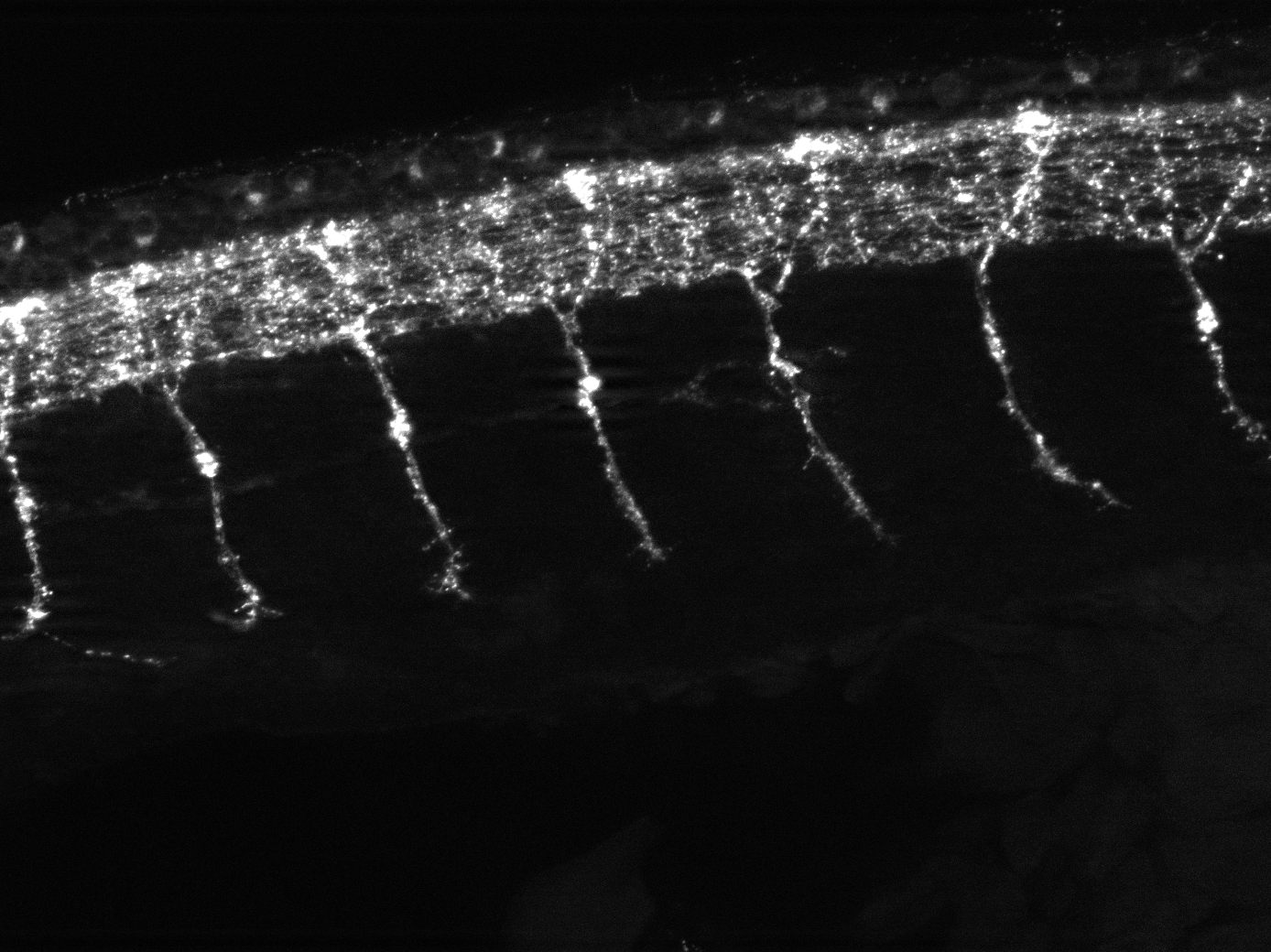

Supplement: Supplementary file 15 — EV Figure Source Data [file 44318_2024_307_MOESM15_ESM.zip › EMBOJ-2024-116734_sourcedataforexpandedviews/Fig EV5/Fig.EV5A_MOCTL_pmn_znp1.tif]

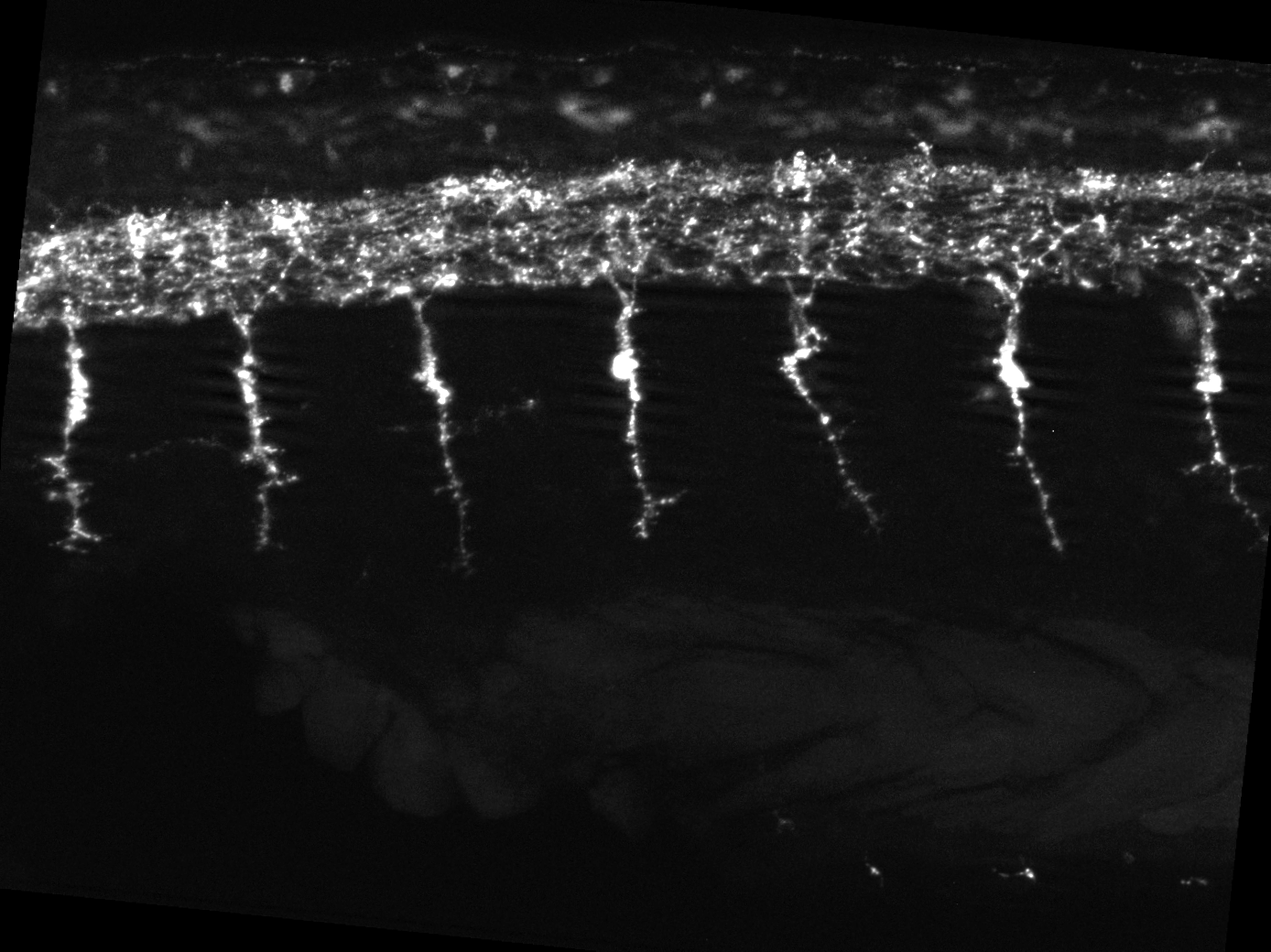

Supplement: Supplementary file 15 — EV Figure Source Data [file 44318_2024_307_MOESM15_ESM.zip › EMBOJ-2024-116734_sourcedataforexpandedviews/Fig EV5/Fig.EVC_MOTTLL11andTTLL11mRNA_pmn.tif]

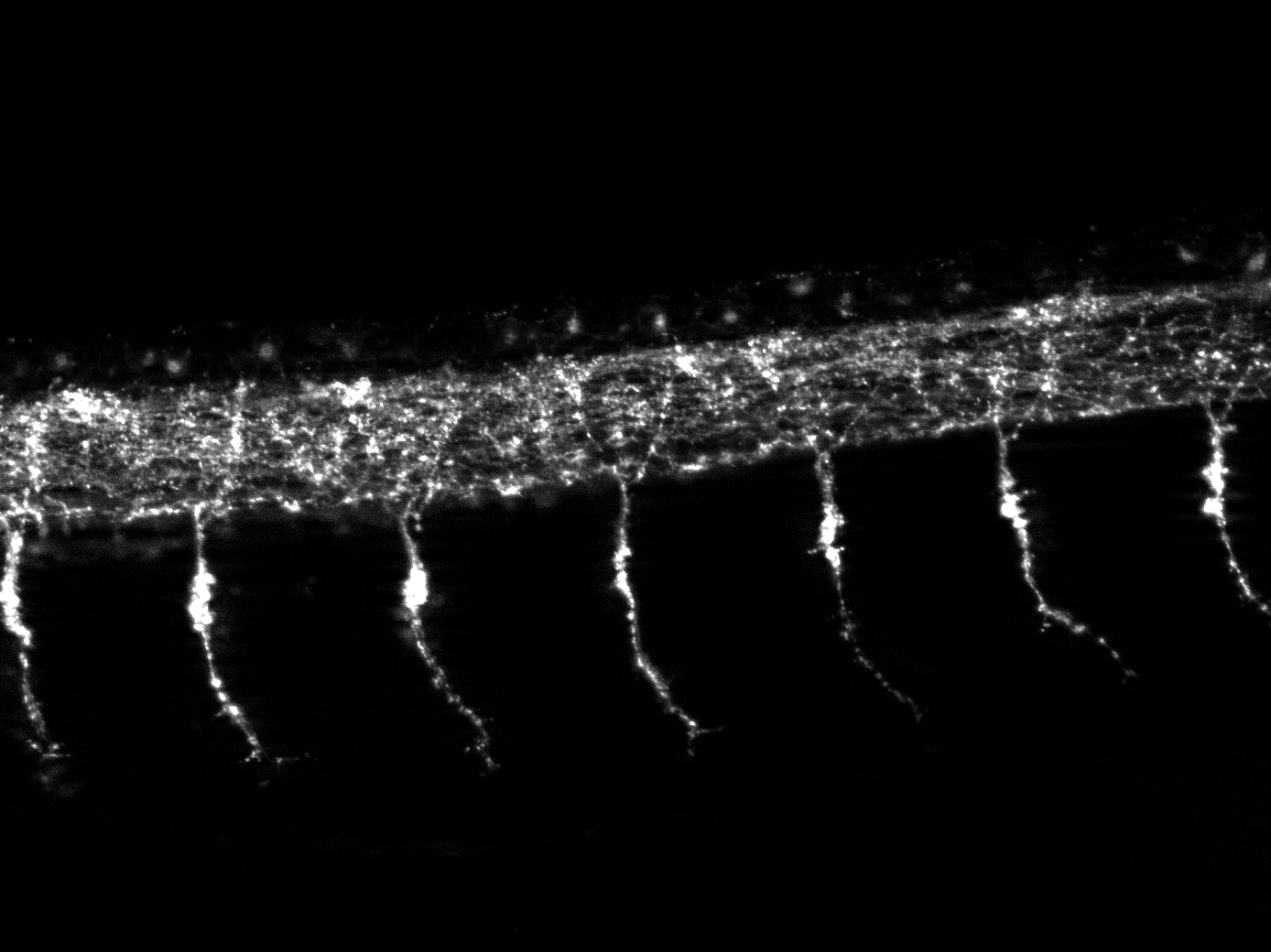

Supplement: Supplementary file 15 — EV Figure Source Data [file 44318_2024_307_MOESM15_ESM.zip › EMBOJ-2024-116734_sourcedataforexpandedviews/Fig EV5/Fig.EV5C_Ctlpmn.tif]

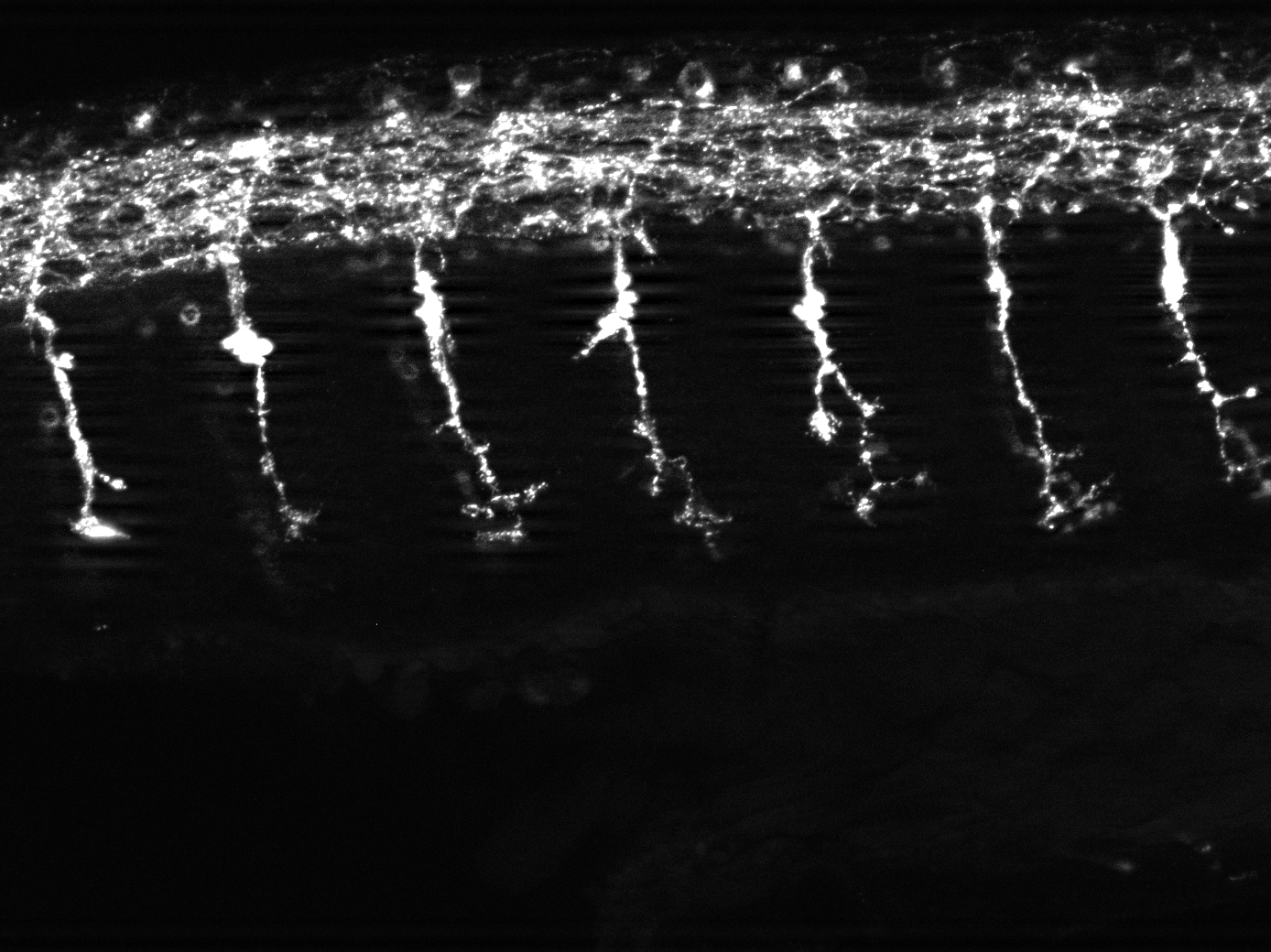

Supplement: Supplementary file 15 — EV Figure Source Data [file 44318_2024_307_MOESM15_ESM.zip › EMBOJ-2024-116734_sourcedataforexpandedviews/Fig EV5/Fig.EV5F_MOKat1.3 and TTLL11mRNA_pmn_znp1.tif]

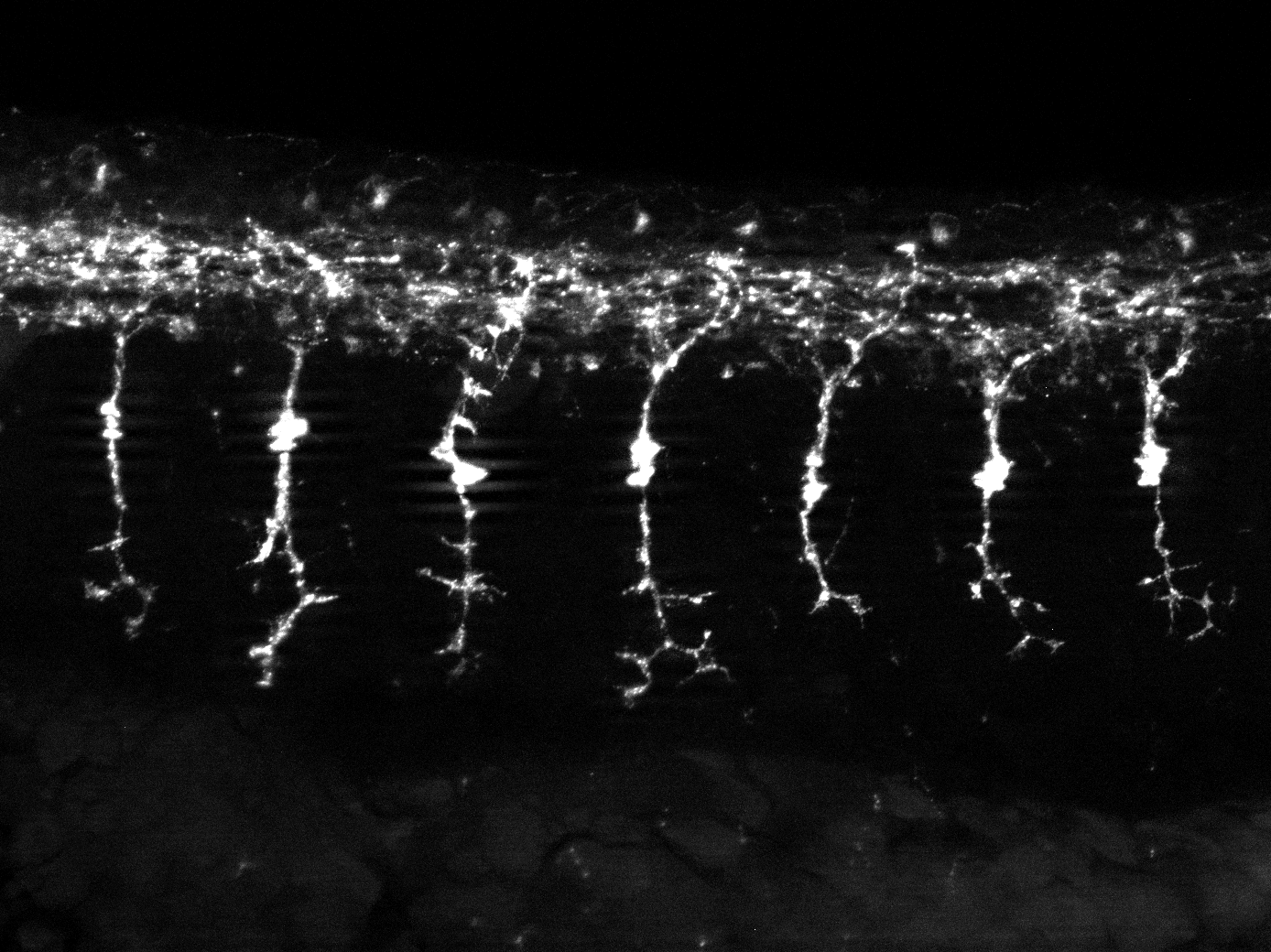

Supplement: Supplementary file 15 — EV Figure Source Data [file 44318_2024_307_MOESM15_ESM.zip › EMBOJ-2024-116734_sourcedataforexpandedviews/Fig EV5/Fig.EV5A_MOKat1.3_pmn_znp1.tif]

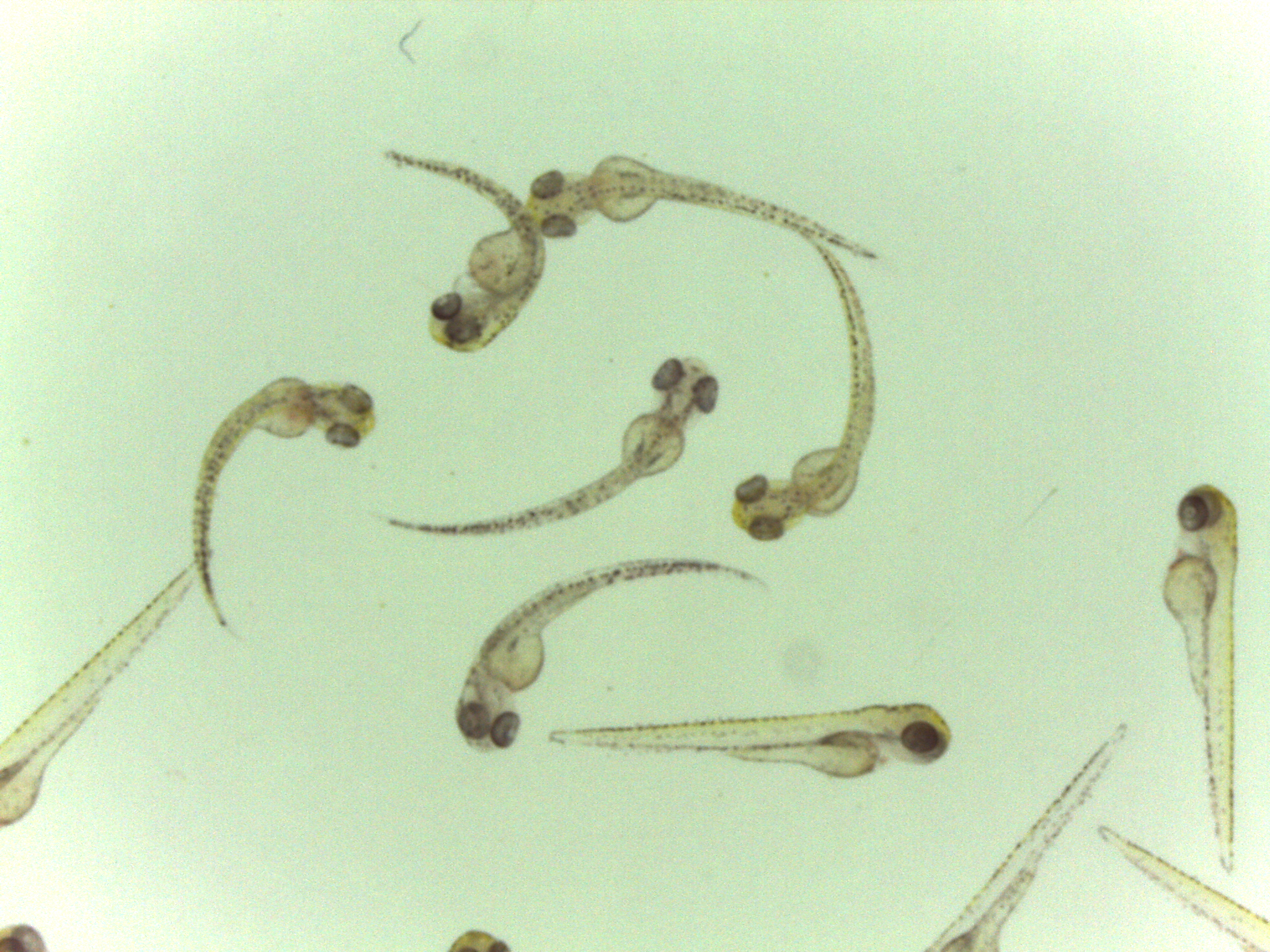

Supplement: Supplementary file 15 — EV Figure Source Data [file 44318_2024_307_MOESM15_ESM.zip › EMBOJ-2024-116734_sourcedataforexpandedviews/Fig EV2/Fig EV2_panel5_Katna1--MZ.tif]

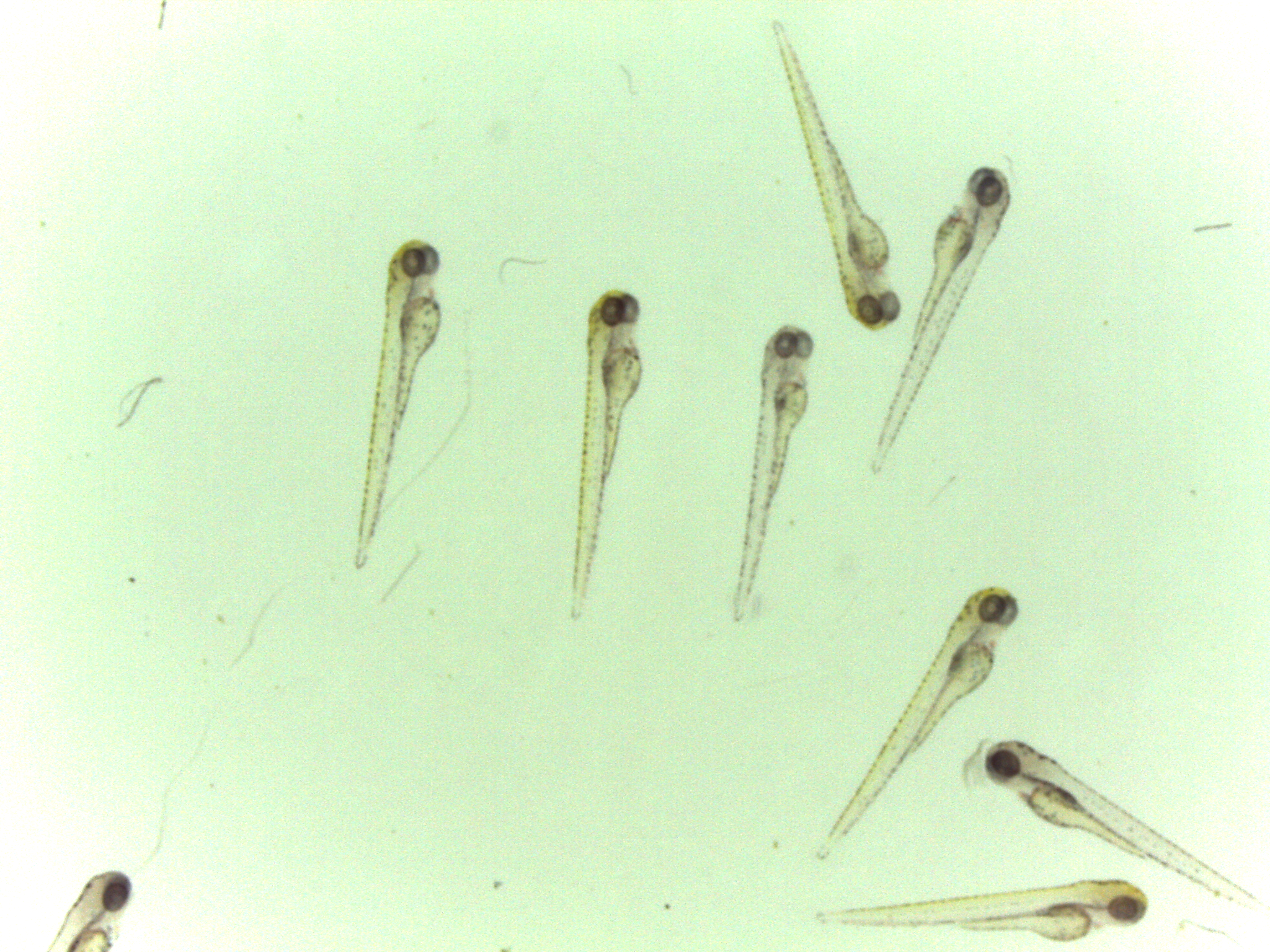

Supplement: Supplementary file 15 — EV Figure Source Data [file 44318_2024_307_MOESM15_ESM.zip › EMBOJ-2024-116734_sourcedataforexpandedviews/Fig EV2/Fig EV2_panel5_Katna1++.tif]

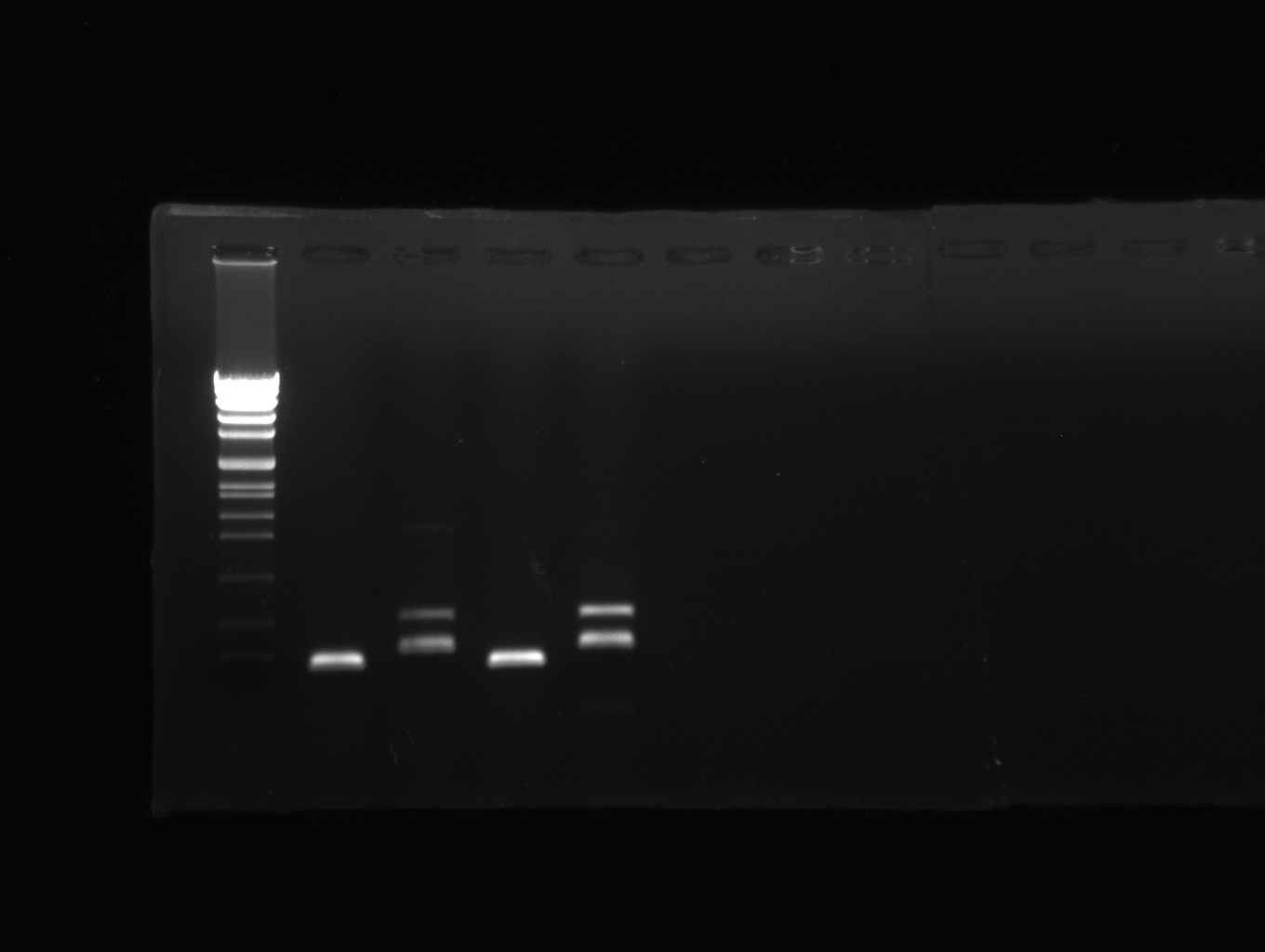

Supplement: Supplementary file 15 — EV Figure Source Data [file 44318_2024_307_MOESM15_ESM.zip › EMBOJ-2024-116734_sourcedataforexpandedviews/Fig EV2/FigEV2_panelC_RT-PCR.tif]

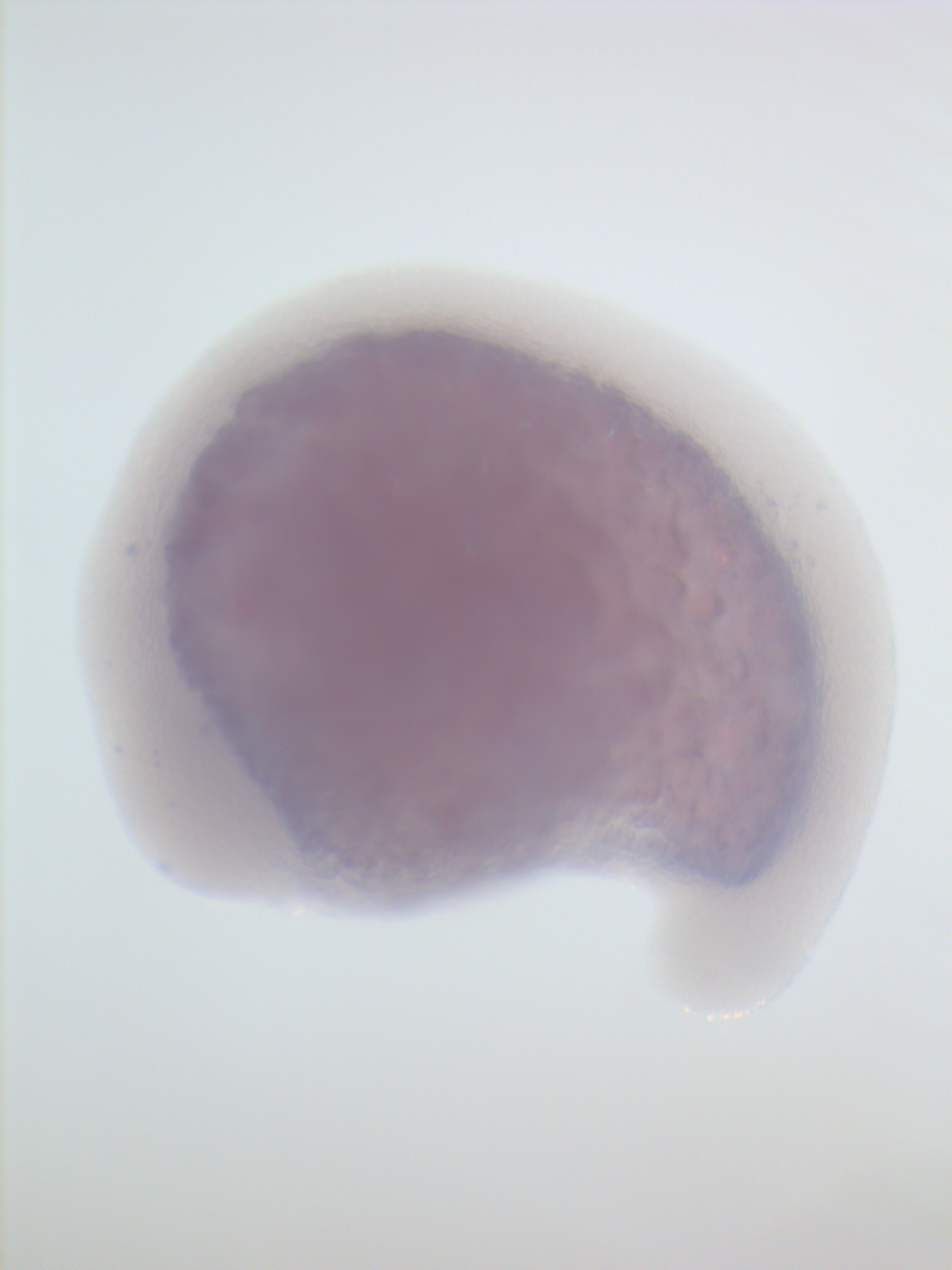

Supplement: Supplementary file 15 — EV Figure Source Data [file 44318_2024_307_MOESM15_ESM.zip › EMBOJ-2024-116734_sourcedataforexpandedviews/Fig EV3/FigEV3_panelA_sense probe_18s.tif]

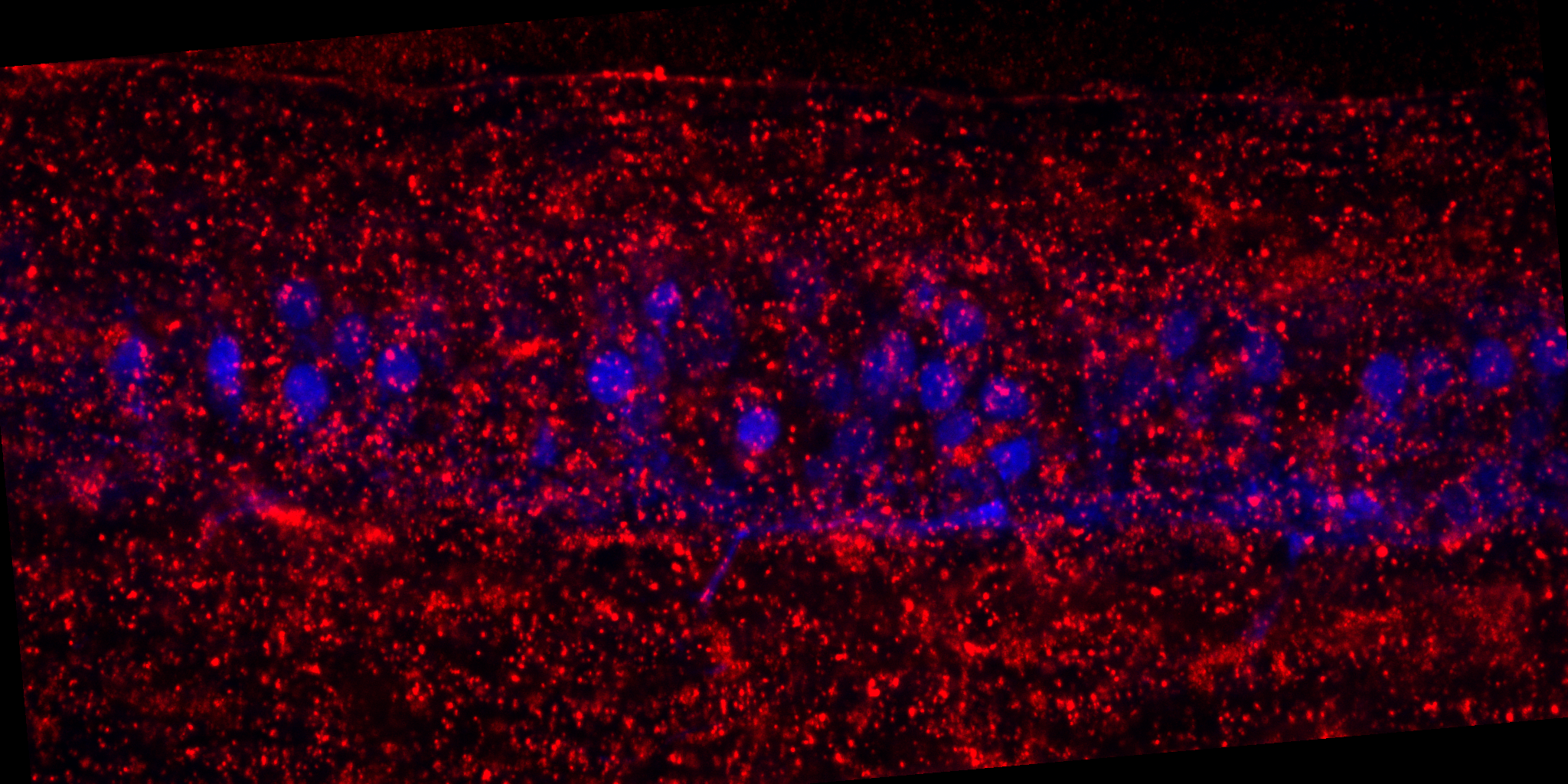

Supplement: Supplementary file 15 — EV Figure Source Data [file 44318_2024_307_MOESM15_ESM.zip › EMBOJ-2024-116734_sourcedataforexpandedviews/Fig EV3/FigEV3_panelB_Katna1probe_GFP.tif]

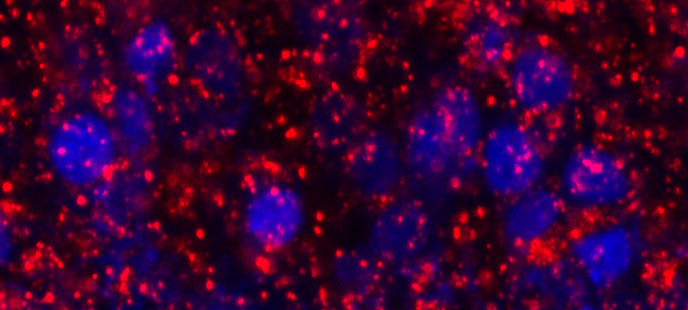

Supplement: Supplementary file 15 — EV Figure Source Data [file 44318_2024_307_MOESM15_ESM.zip › EMBOJ-2024-116734_sourcedataforexpandedviews/Fig EV3/FigEV3_panelB_Katna1probe_GFPzoom.tif]

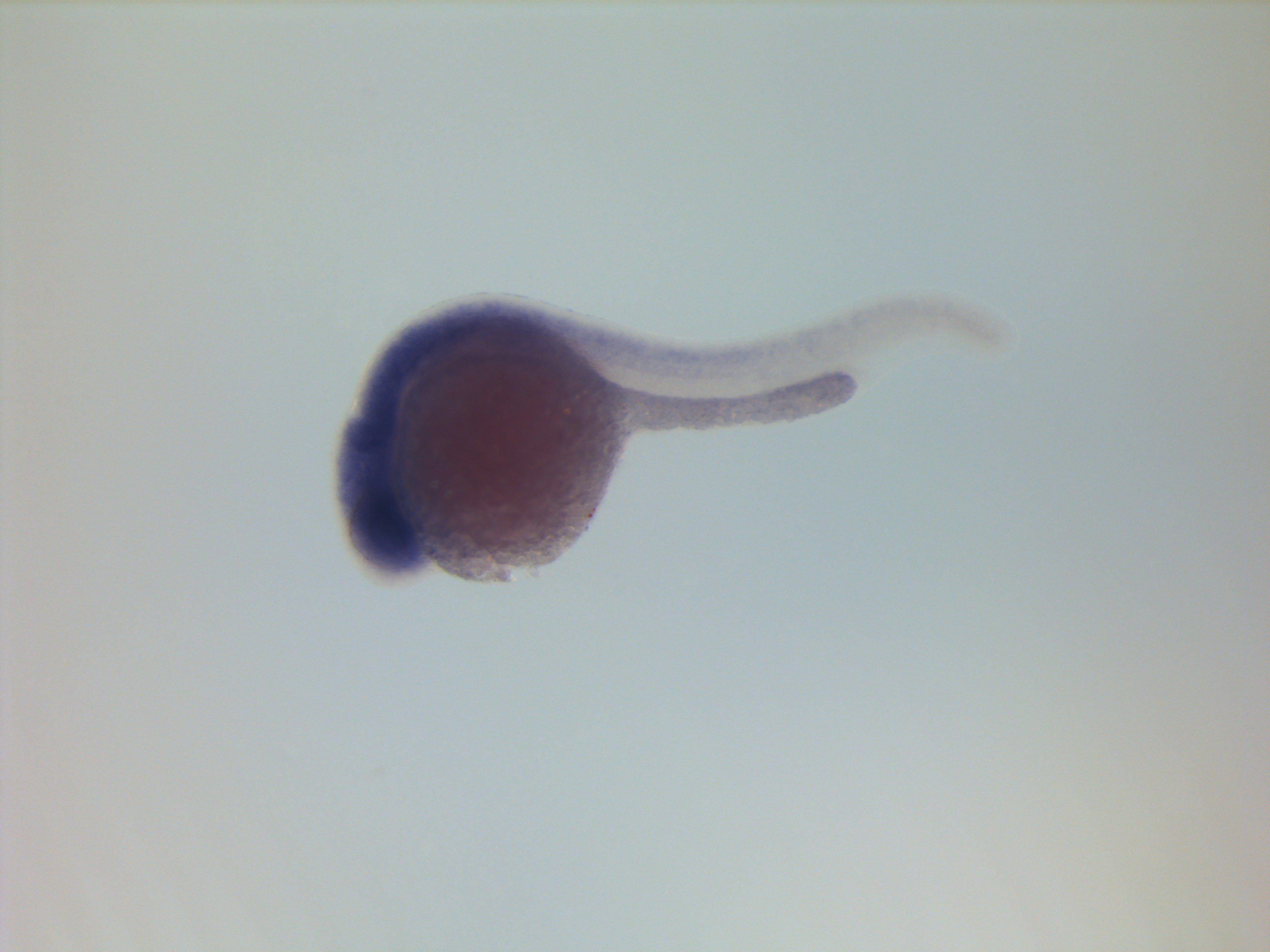

Supplement: Supplementary file 15 — EV Figure Source Data [file 44318_2024_307_MOESM15_ESM.zip › EMBOJ-2024-116734_sourcedataforexpandedviews/Fig EV3/FigEV3_panelA_antisense probe_24hpf.tif]

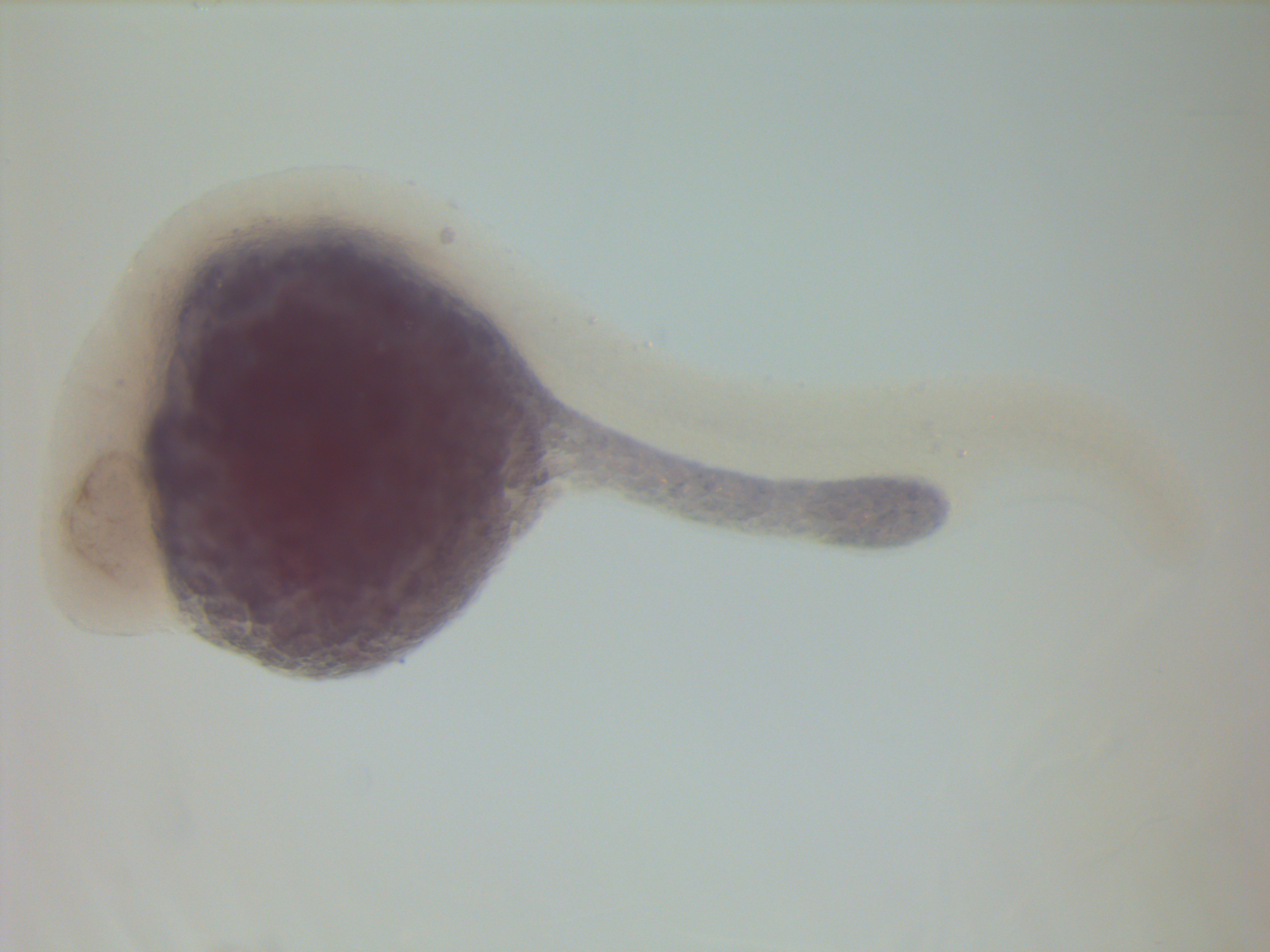

Supplement: Supplementary file 15 — EV Figure Source Data [file 44318_2024_307_MOESM15_ESM.zip › EMBOJ-2024-116734_sourcedataforexpandedviews/Fig EV3/FigEV3_panelA_sense probe_24hpf.tif]

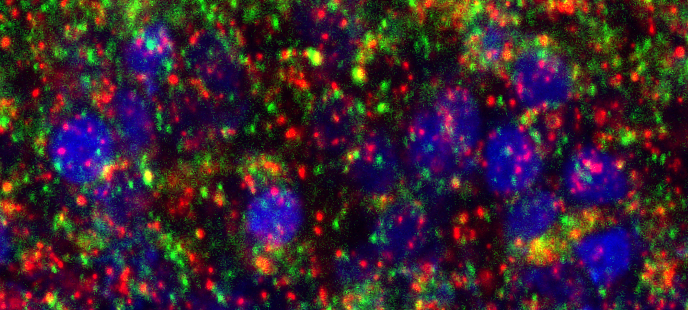

Supplement: Supplementary file 15 — EV Figure Source Data [file 44318_2024_307_MOESM15_ESM.zip › EMBOJ-2024-116734_sourcedataforexpandedviews/Fig EV3/FigEV3_panelB_katna1probe_spastinprobe_GFPzoom.tif]

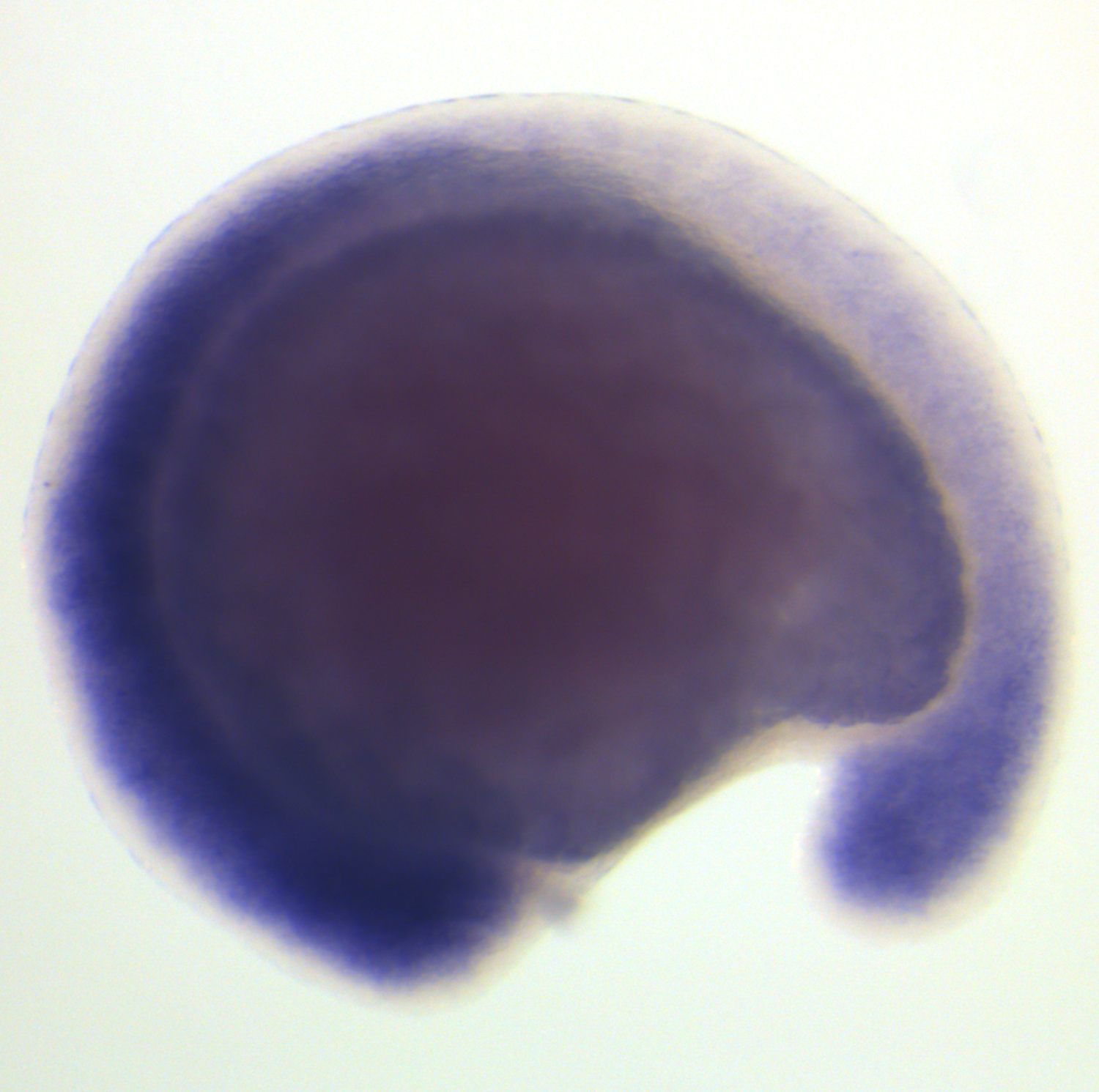

Supplement: Supplementary file 15 — EV Figure Source Data [file 44318_2024_307_MOESM15_ESM.zip › EMBOJ-2024-116734_sourcedataforexpandedviews/Fig EV3/FigEV3_panelA_antisense probe_18s.tif]

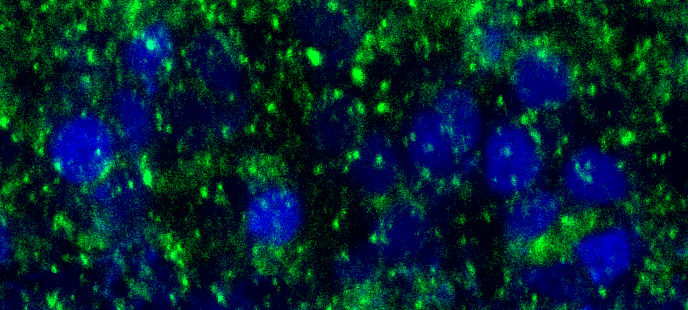

Supplement: Supplementary file 15 — EV Figure Source Data [file 44318_2024_307_MOESM15_ESM.zip › EMBOJ-2024-116734_sourcedataforexpandedviews/Fig EV3/FigEV3_panelB_spastinprobe_GFPzoom.tif]

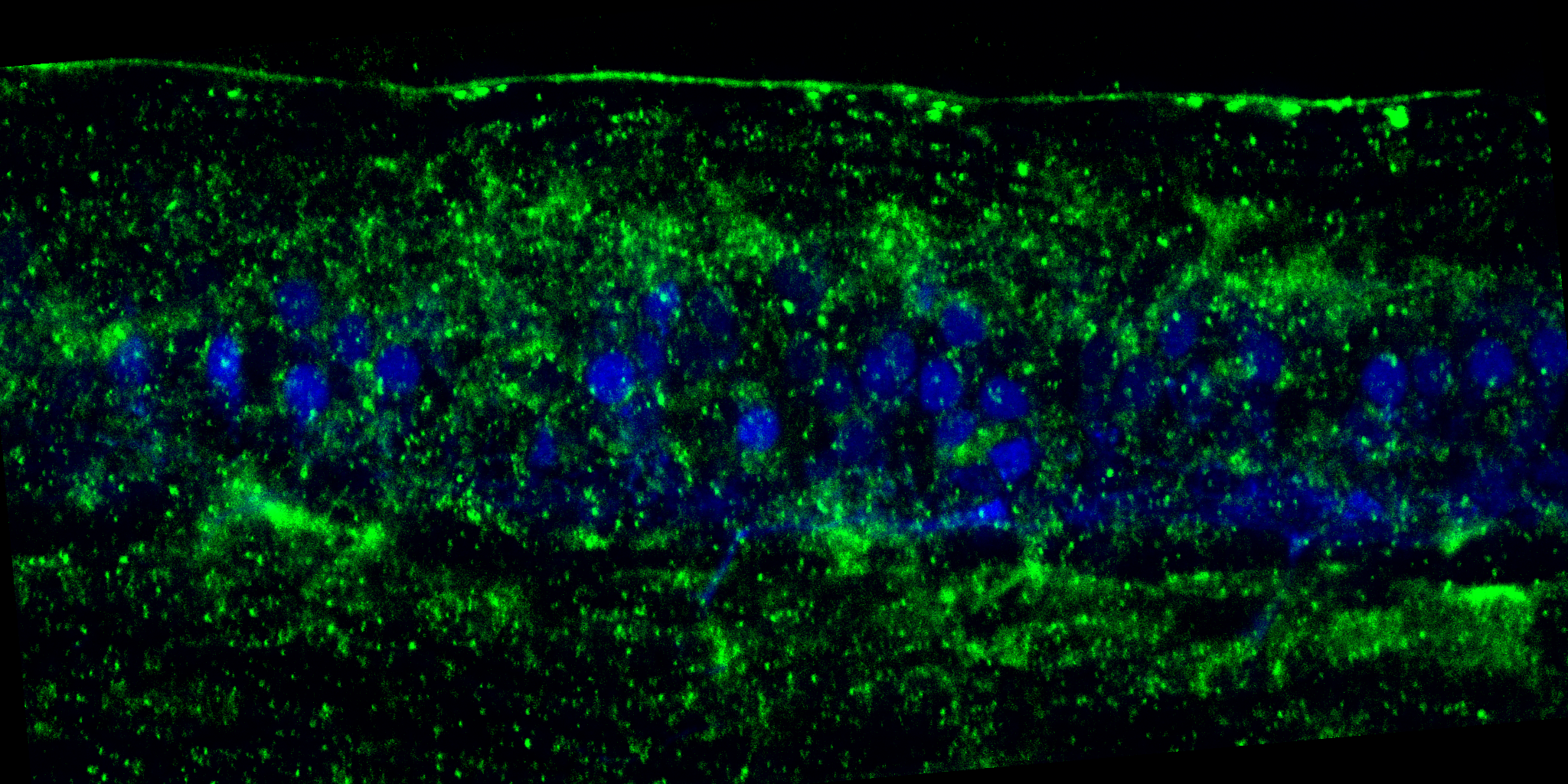

Supplement: Supplementary file 15 — EV Figure Source Data [file 44318_2024_307_MOESM15_ESM.zip › EMBOJ-2024-116734_sourcedataforexpandedviews/Fig EV3/FigEV3_panelB_spastinprobe_GFP.tif]

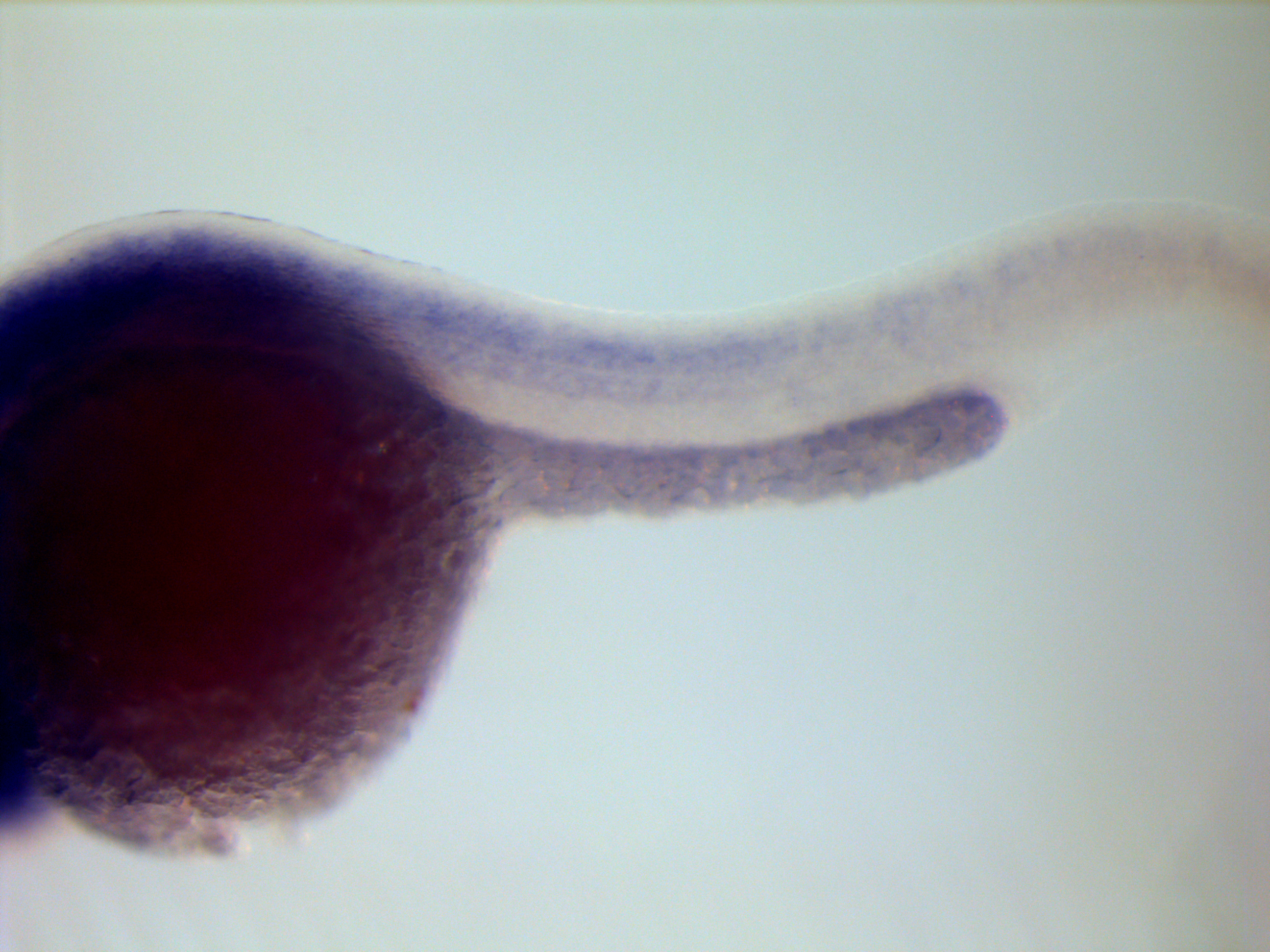

Supplement: Supplementary file 15 — EV Figure Source Data [file 44318_2024_307_MOESM15_ESM.zip › EMBOJ-2024-116734_sourcedataforexpandedviews/Fig EV3/FigEV3_panelA_antisense probe_24hpf_zoom.tif]

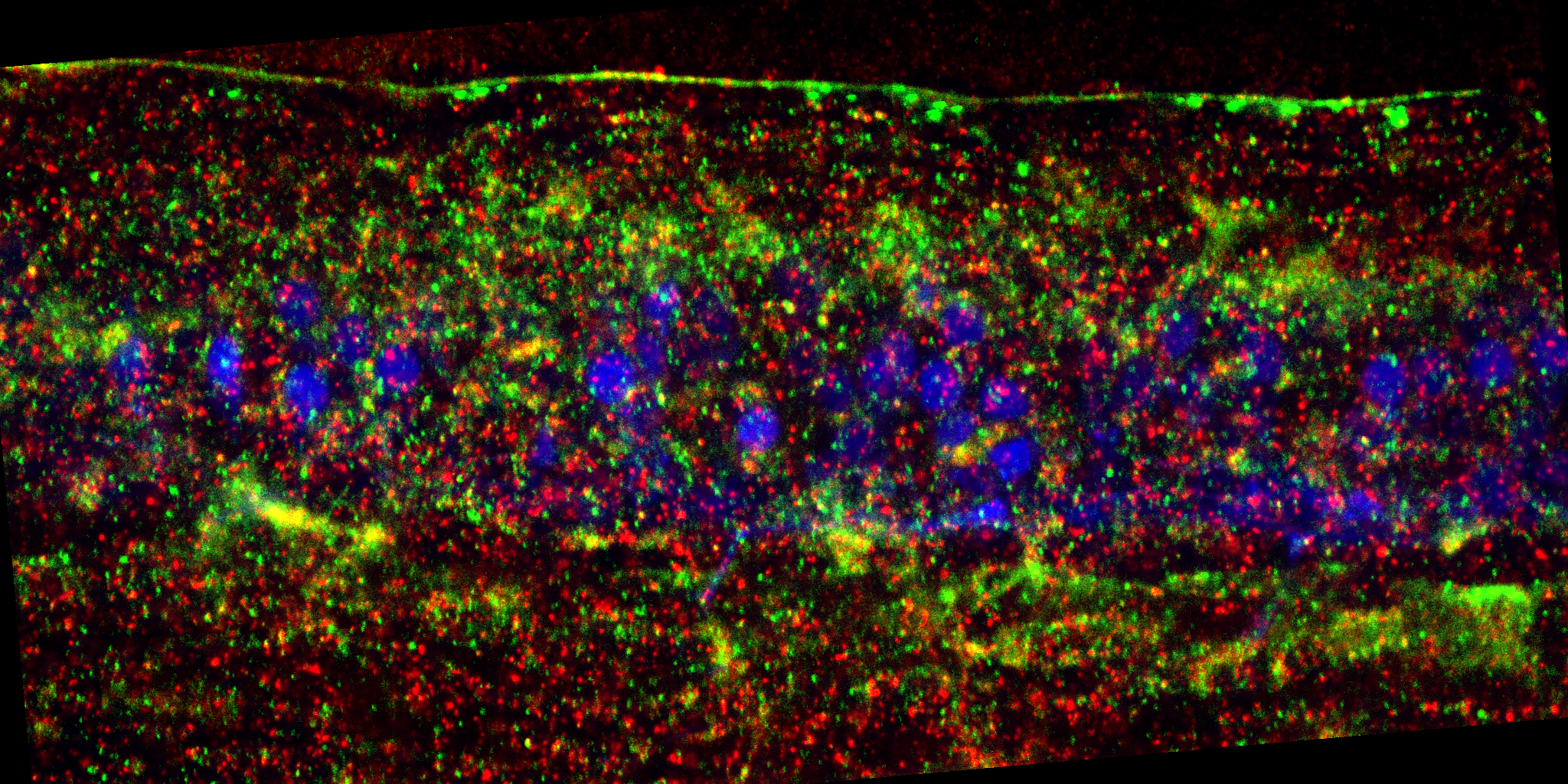

Supplement: Supplementary file 15 — EV Figure Source Data [file 44318_2024_307_MOESM15_ESM.zip › EMBOJ-2024-116734_sourcedataforexpandedviews/Fig EV3/FigEV3_panelB_katna1probe_spastinprobe_GFP.tif]

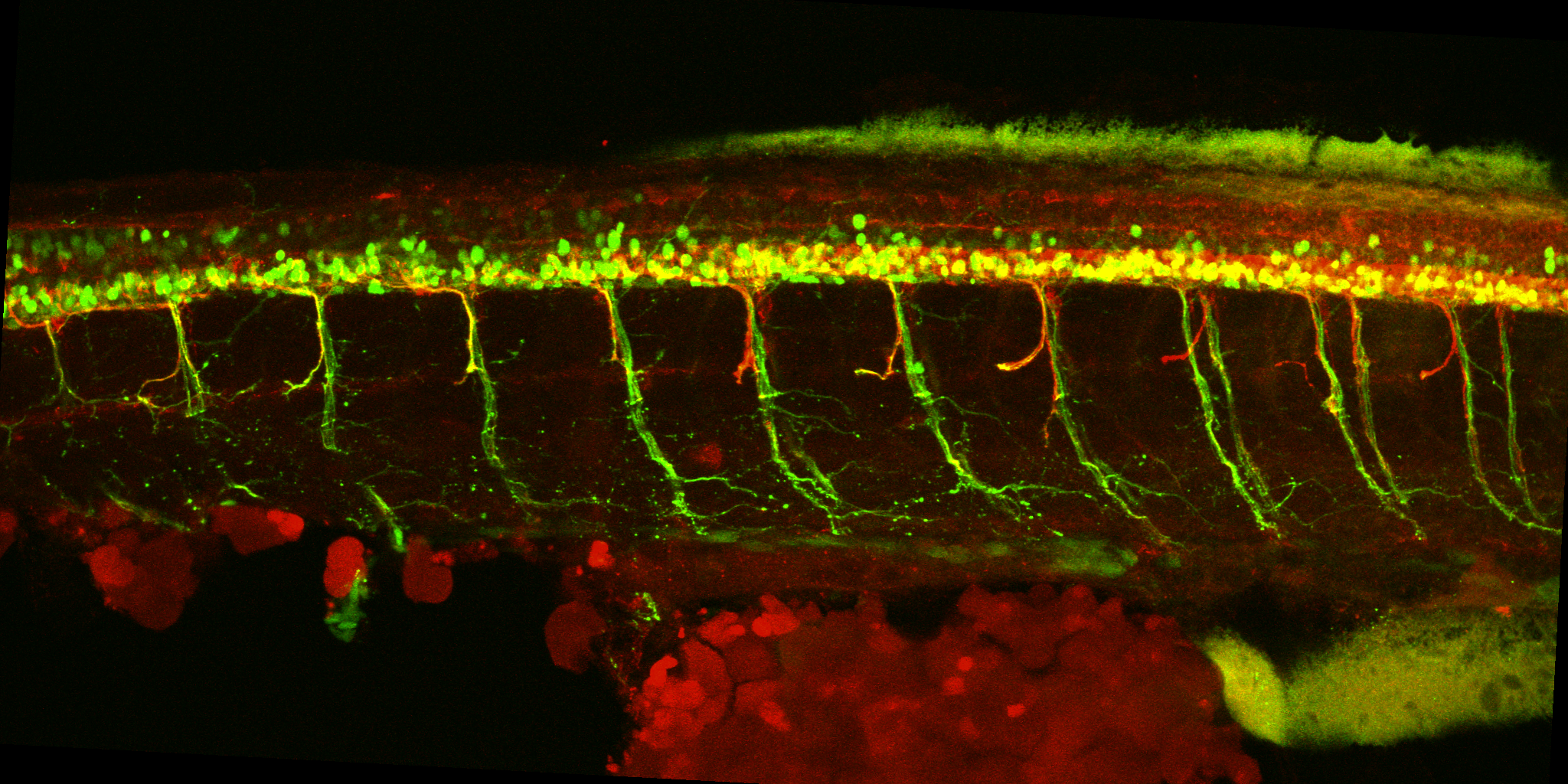

Supplement: Supplementary file 15 — EV Figure Source Data [file 44318_2024_307_MOESM15_ESM.zip › EMBOJ-2024-116734_sourcedataforexpandedviews/Fig EV4/FigEV4_panelB_MOCspATGand TTLL11deadmRNA_sMN_zn5GFP.tif]

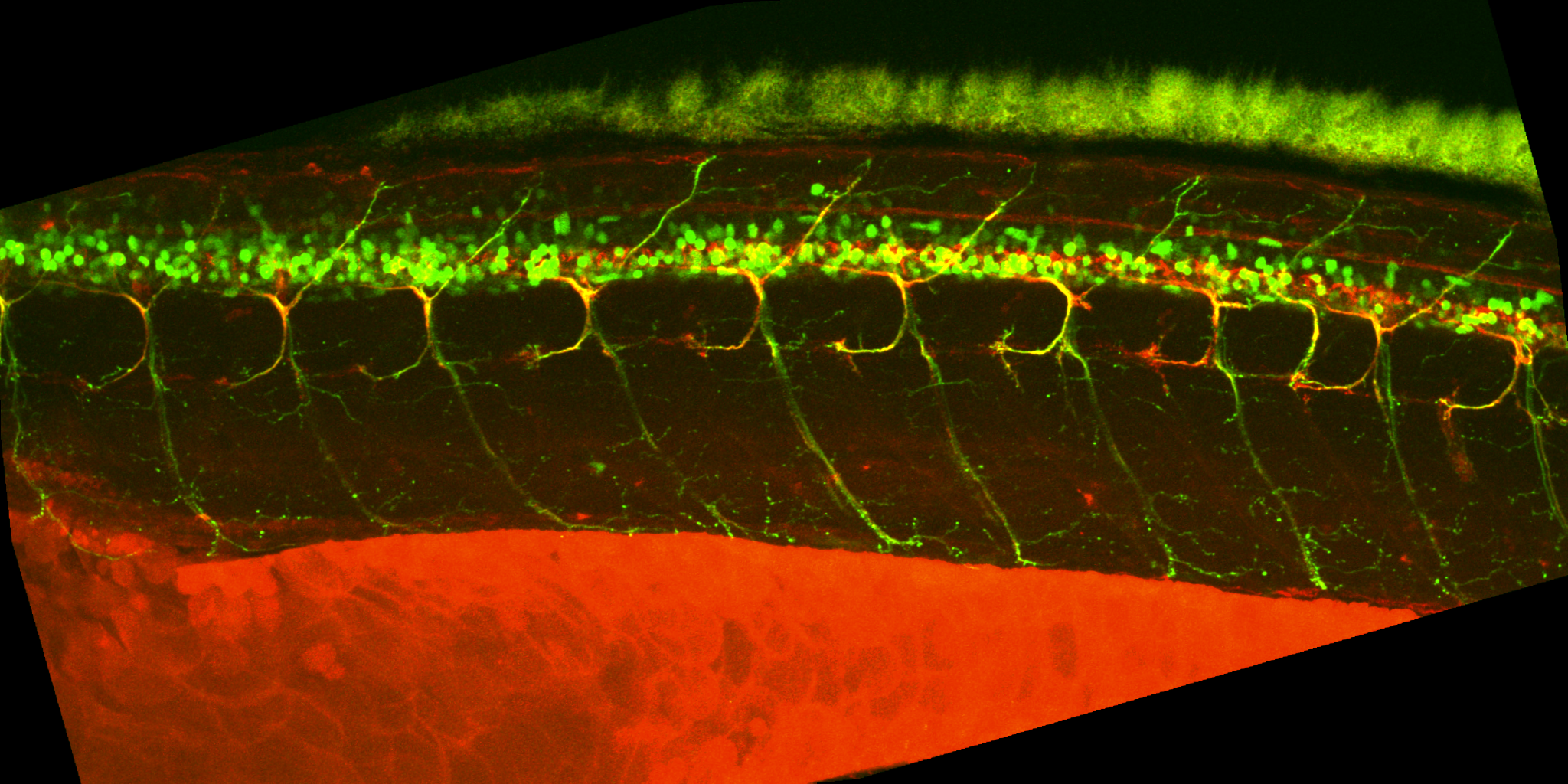

Supplement: Supplementary file 15 — EV Figure Source Data [file 44318_2024_307_MOESM15_ESM.zip › EMBOJ-2024-116734_sourcedataforexpandedviews/Fig EV4/FigEV4_panelB_MOCTL_sMN_zn5GFP.tif]

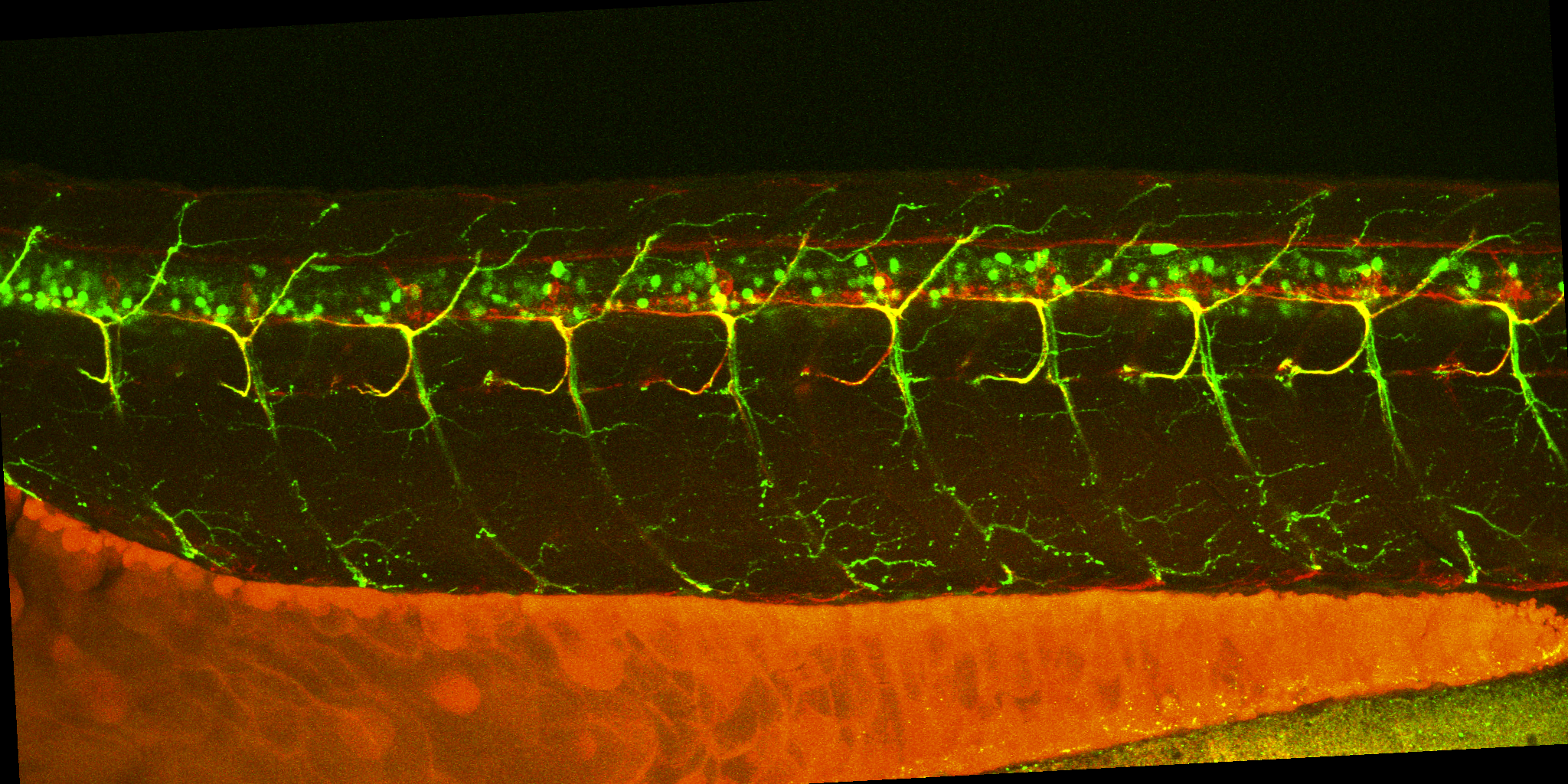

Supplement: Supplementary file 15 — EV Figure Source Data [file 44318_2024_307_MOESM15_ESM.zip › EMBOJ-2024-116734_sourcedataforexpandedviews/Fig EV4/FigEV4_panelA_MOCTL_sMN_zn5GFP.tif]

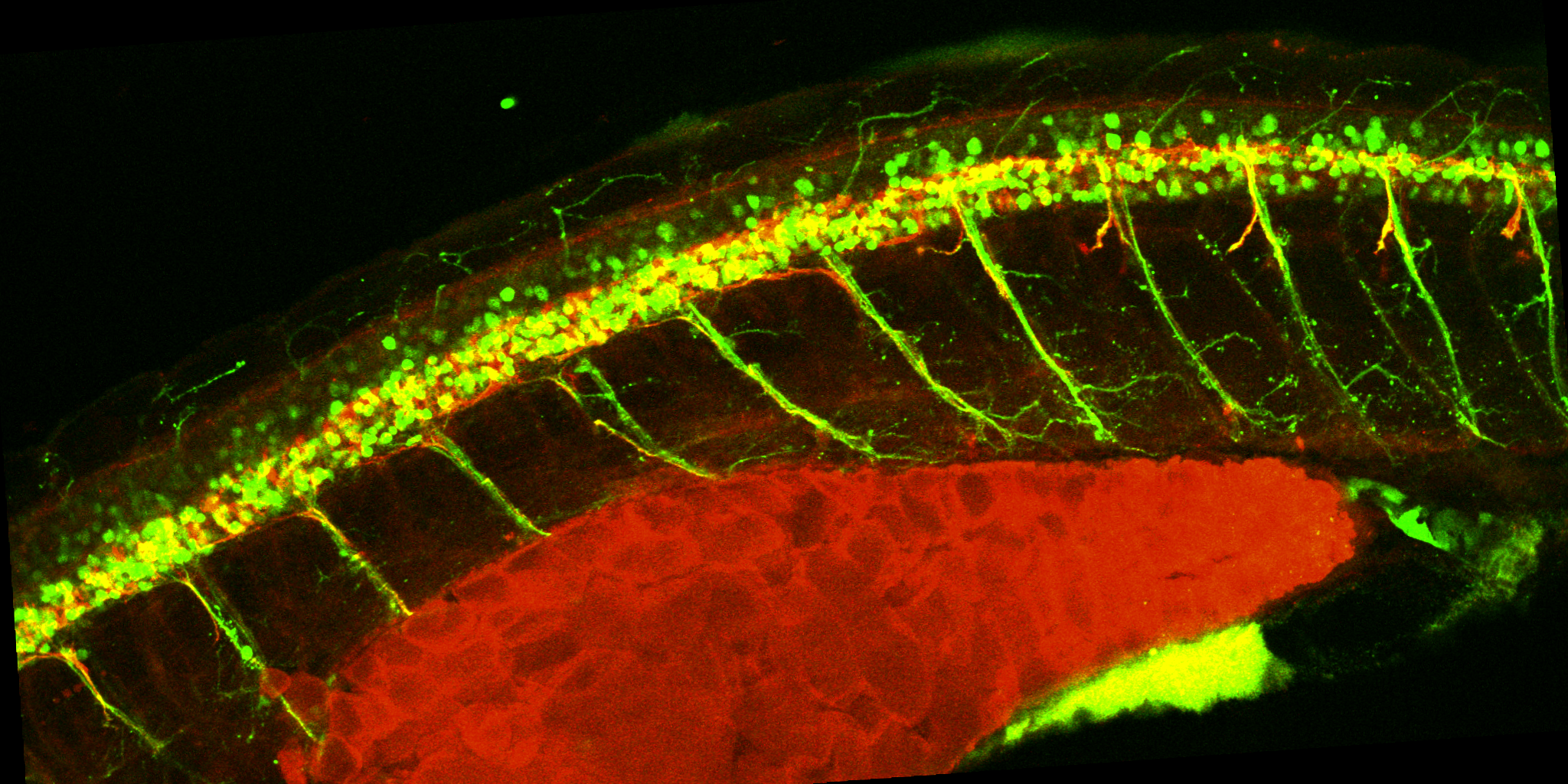

Supplement: Supplementary file 15 — EV Figure Source Data [file 44318_2024_307_MOESM15_ESM.zip › EMBOJ-2024-116734_sourcedataforexpandedviews/Fig EV4/FigEV4_panelB_MOCspATG_sMN_zn5GFP.tif]

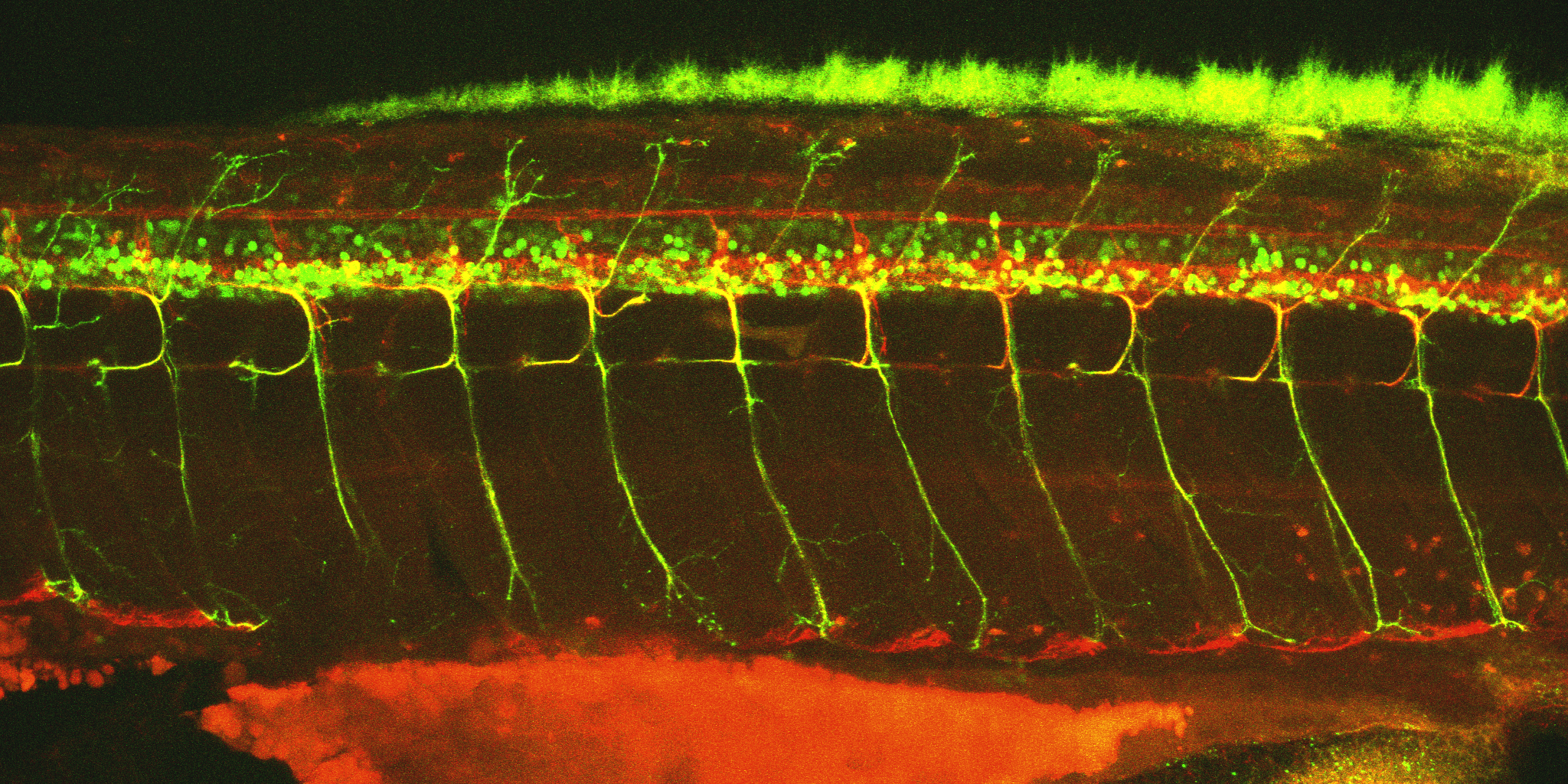

Supplement: Supplementary file 15 — EV Figure Source Data [file 44318_2024_307_MOESM15_ESM.zip › EMBOJ-2024-116734_sourcedataforexpandedviews/Fig EV4/FigEV4_panelA_MOKat1.3and TTLL6deadmRNA_sMN_zn5GFP.tif]

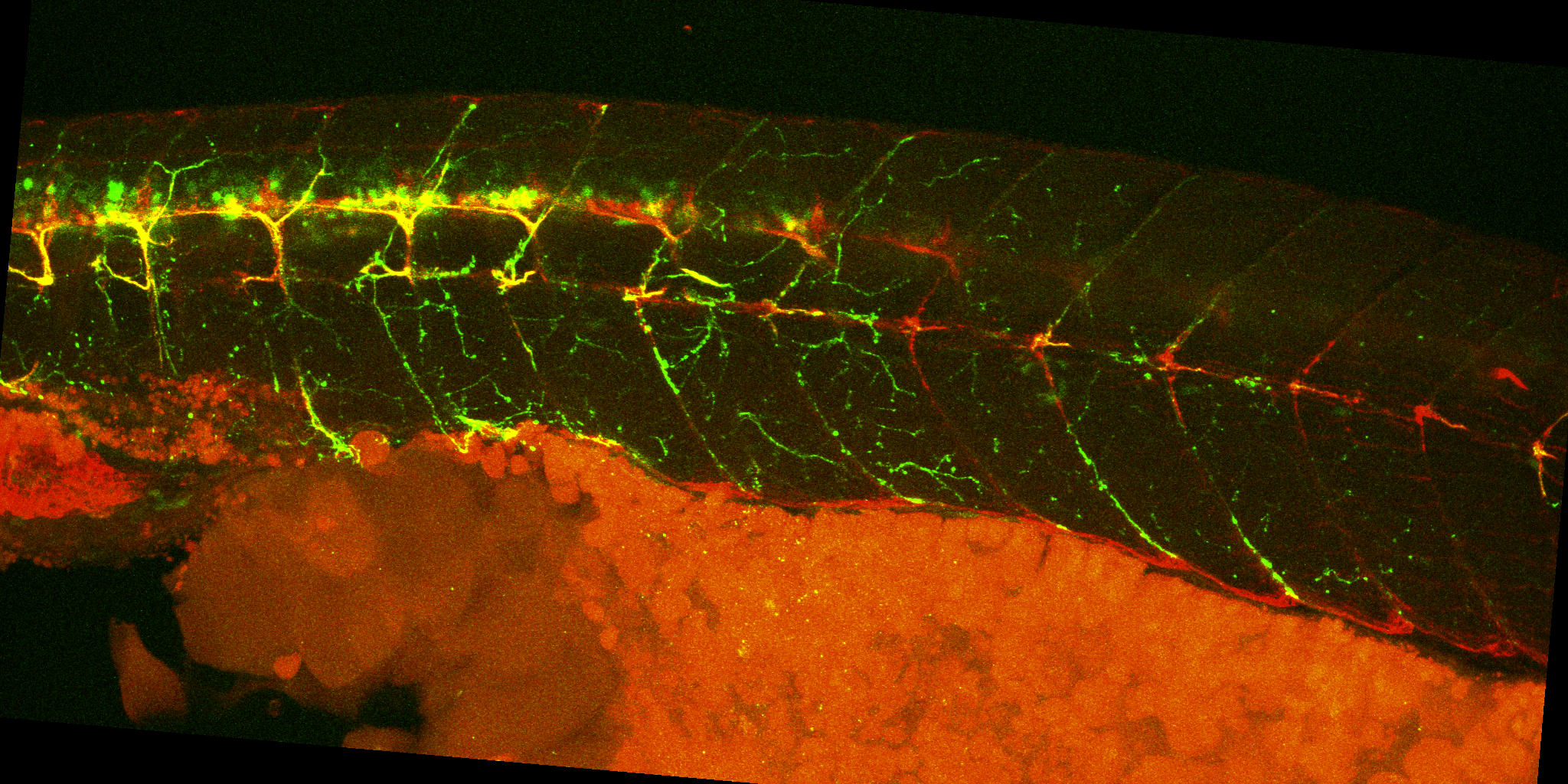

Supplement: Supplementary file 15 — EV Figure Source Data [file 44318_2024_307_MOESM15_ESM.zip › EMBOJ-2024-116734_sourcedataforexpandedviews/Fig EV4/FigEV4_panelA_MOKat1.3_sMN_zn5GFP.tif]
