# Supplementary material for: Tubulin glutamylation regulates axon guidance via the selective tuning of microtubule-severing enzymes
Source: EMBO J. 2024 Nov 29;44(1):107–40. doi: 10.1038/s44318-024-00307-x (PMC11695996; doi:10.1038/s44318-024-00307-x)
Supplement: Supplementary file 16 — Expanded View Figures [file 44318_2024_307_MOESM16_ESM.pdf]

Expanded View Figures

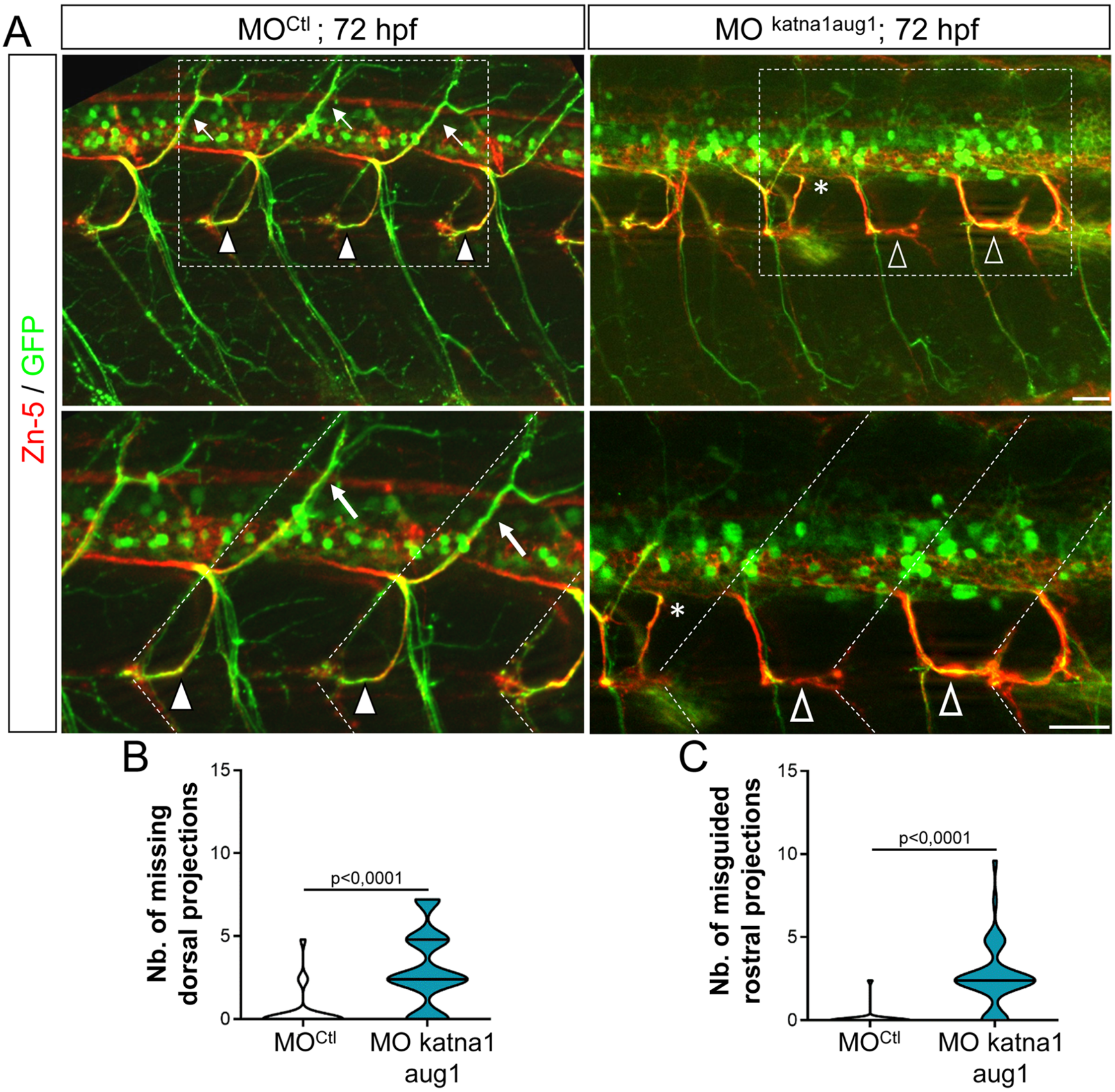

Figure EV1. ***p60-katanin*** **knockdown with MO^{katna1aug1} morpholino induces similar spinal motor axon defects to MO^{p60Kat} morpholino.**

(**A**) Immunolabelling of secondary motoneuron (sMN) axon tracts in 72 h post-fertilisation (hpf) transgenic Tg(*Hb9*:GFP) larvae injected with control ($n = 40$) or MO^{katna1aug1} ($n = 40$) morpholinos, using Zn-5 and GFP antibodies. Lateral views of the trunk, anterior to the left. Bottom panels represent higher magnifications of the boxed region in the corresponding top panels. Dotted lines delineate lateral myosepta. Full arrowheads and full arrows point at normal rostral and dorsal nerves, respectively. Empty arrowheads show misguided rostral nerves. Asterisks indicate ectopic sorting points of sMN axons from the spinal cord. (**B**, **C**) Quantifications of sMN defects in larvae analysed in panel A and pooled from three independent experiments. Mean number of missing dorsal nerves (**B**) and misguided rostral nerves (**C**) per larva. Non-blind quantifications were performed on 24 spinal hemisegments located around the yolk tube per larva. Violin Plots; horizontal bars indicate the median ± the 1st and 3rd quartiles. Mann–Whitney test. *P* values are displayed on graphs. Source data are available online for this figure

© The Author(s)

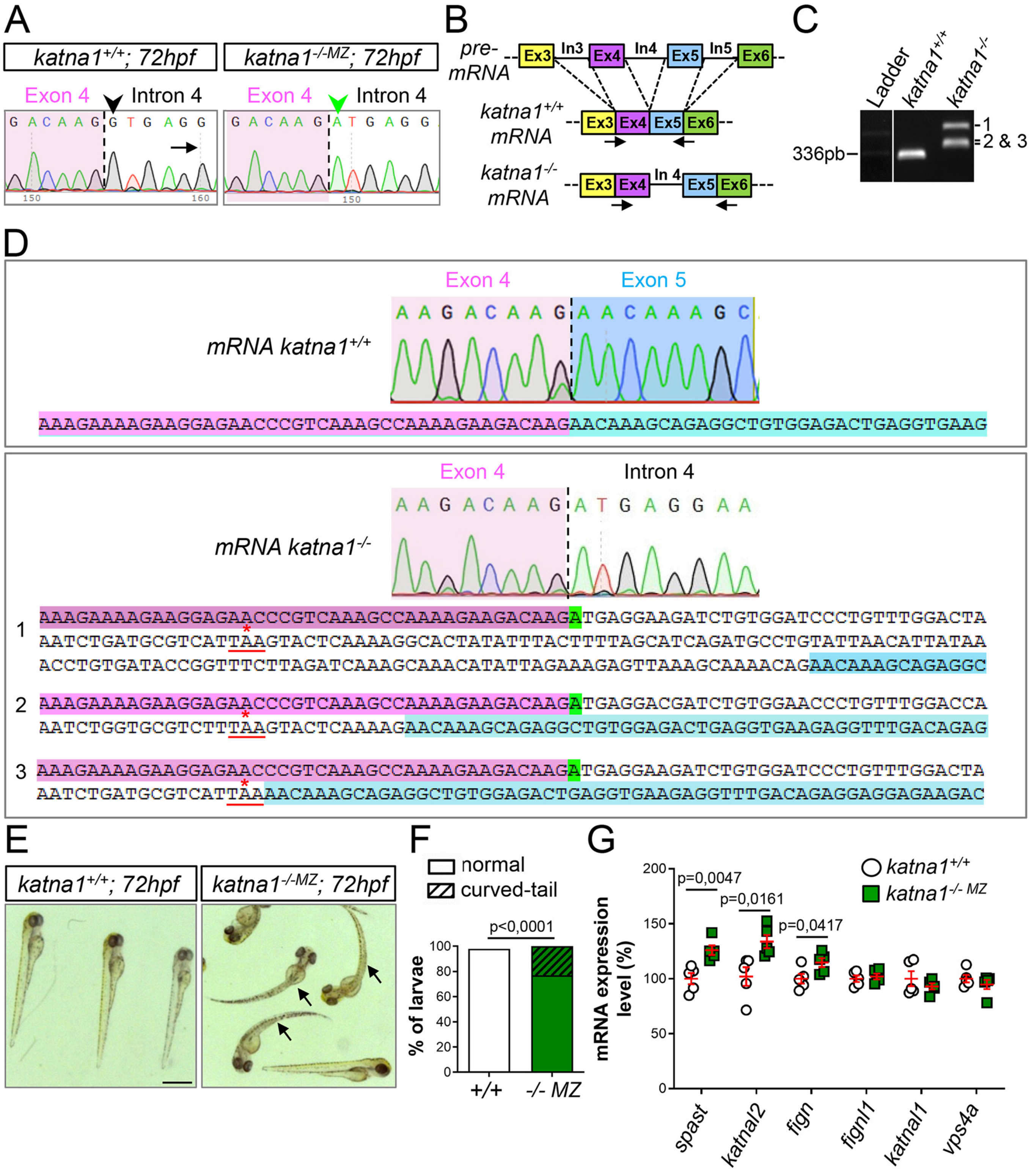
A
katna1+/+; 72hpf
katna1-/-MZ; 72hpf
Exon 4
Intron 4
GACAAGGTGAGG
GACAAGATGAGG
150
160
B
pre-mRNA
Ex3 In3 Ex4 In4 Ex5 In5 Ex6
katna1+/+ mRNA
Ex3 Ex4 Ex5 Ex6
katna1-/- mRNA
Ex3 Ex4 In 4 Ex5 Ex6
C
Ladder
katna1+/+
katna1-/-
336pb
1
2 & 3
D
Exon 4
Exon 5
mRNA katna1+/+
AAGACAAGAACAAAGC
AAAGAAAAGAAGGAGAACCCGTCAAAGCCAAAAGAAGACAAGAACAAAGCAGAGGCTGTGGAGACTGAGGTGAAG
Exon 4
Intron 4
mRNA katna1-/-
AAGACAAGATGAGGAA
1
AAAGAAAAGAAGGAGAACCCGTCAAAGCCAAAAGAAGACAAGATGAGGAAGATCTGTGGATCCCTGTTTGGACTA
AATCTGATGCGTCATTAAGTACTCAAAAGGCACTATATTTACTTTTAGCATCAGATGCCTGTATTAACATTATAA
ACCTGTGATACCGGTTTCTTAGATCAAAGCAAACATATTAGAAAGAGTTAAAGCAAAACAGAACAAAGCAGAGGC
2
AAAGAAAAGAAGGAGAACCCGTCAAAGCCAAAAGAAGACAAGATGAGGACGATCTGTGGAACCCTGTTTGGACCA
AATCTGGTGCGTCTTTAAGTACTCAAAAGAACAAAGCAGAGGCTGTGGAGACTGAGGTGAAGAGGTTTGACAGAG
3
AAAGAAAAGAAGGAGAACCCGTCAAAGCCAAAAGAAGACAAGATGAGGAAGATCTGTGGATCCCTGTTTGGACTA
AATCTGATGCGTCATTAAAACAAAGCAGAGGCTGTGGAGACTGAGGTGAAGAGGTTTGACAGAGGAGGAGAAGAC
E
katna1+/+; 72hpf
katna1-/-MZ; 72hpf
F
normal
curved-tail
p<0,0001
% of larvae
100
80
60
40
20
0
+/+
-/- MZ
G
katna1+/+
katna1-/- MZ
p=0,0047
p=0,0161
p=0,0417
mRNA expression level (%)
200
150
100
50
0
spast
katnal2
fign
fignl1
katnal1
vps4a

 © The Author(s)

Figure EV2. Molecular and morphological characterisation of zebrafish *katna1* mutants.

(**A**) Sequence analysis of control and *katna1* mutant genomic DNA. Dotted line indicates the junction between exon 4 (pink background) and intron 4 (white background). The green arrowhead points at the nucleotide substitution (G > A) affecting the donor splice site of intron 4 (G in control, black arrowhead) in *katna1* mutants. (**B**) Schematic representation of the RT-PCR strategy used to test the impact of the *katna1* splice-site mutation on *katna1* mRNA splicing. Dotted lines indicate intron splicing. Arrows represent the primers used for RT-PCR analysis. Primers were designed on exon/intron junctions to avoid contamination by genomic DNA amplification. In: intron; Ex: exon. (**C**) RT-PCR analysis of *katna1* intron-4 splicing on total RNA extracts from $katna1^{+/+}$ and $katna1^{-/-MZ}$ maternal zygotic mutant embryos. Homozygous $katna1^{-/-MZ}$ embryos lack wild-type transcript and show different populations of misspliced transcripts (1, 2 and 3). (**D**) Sequence analysis of *katna1* misspliced transcripts. Sequences corresponding to exon 4, intron 4 and exon 5 are respectively indicated in pink, white and blue. The splice-site mutation is highlighted in green. Misspliced transcripts include various sized insertions of intron 4, which all lead to a frameshift and the occurrence of a premature stop codon at the same amino-acid position (red asterisk). (**E**) Gross morphology of 72-hpf control ($katna1^{+/+}$) and maternal zygotic *katna1* mutant ($katna1^{-/-MZ}$) larvae. Arrows point at the curved-tail phenotype of some mutant larvae. Scale bar: 0.5 mm. (**F**) Percentage of larvae exhibiting a curved-tail phenotype. Chi^2 test. (**G**) RT-qPCR analysis showing the differential expression levels of *p60-katanin*-related genes from the ATPase Meiotic Clade in control and $katna1^{-/-MZ}$ larvae. RNAs were extracted from 5 independent pools of 10 $katna1^{+/+}$, 10 $katna1^{-/-MZ}$ and one pool of 10 wild-type embryos at 24 hpf. Unpaired *t* test. Values are shown as means ± SEM. (**F**, **G**) *P* values are displayed on graphs. Source data are available online for this figure

© The Author(s)

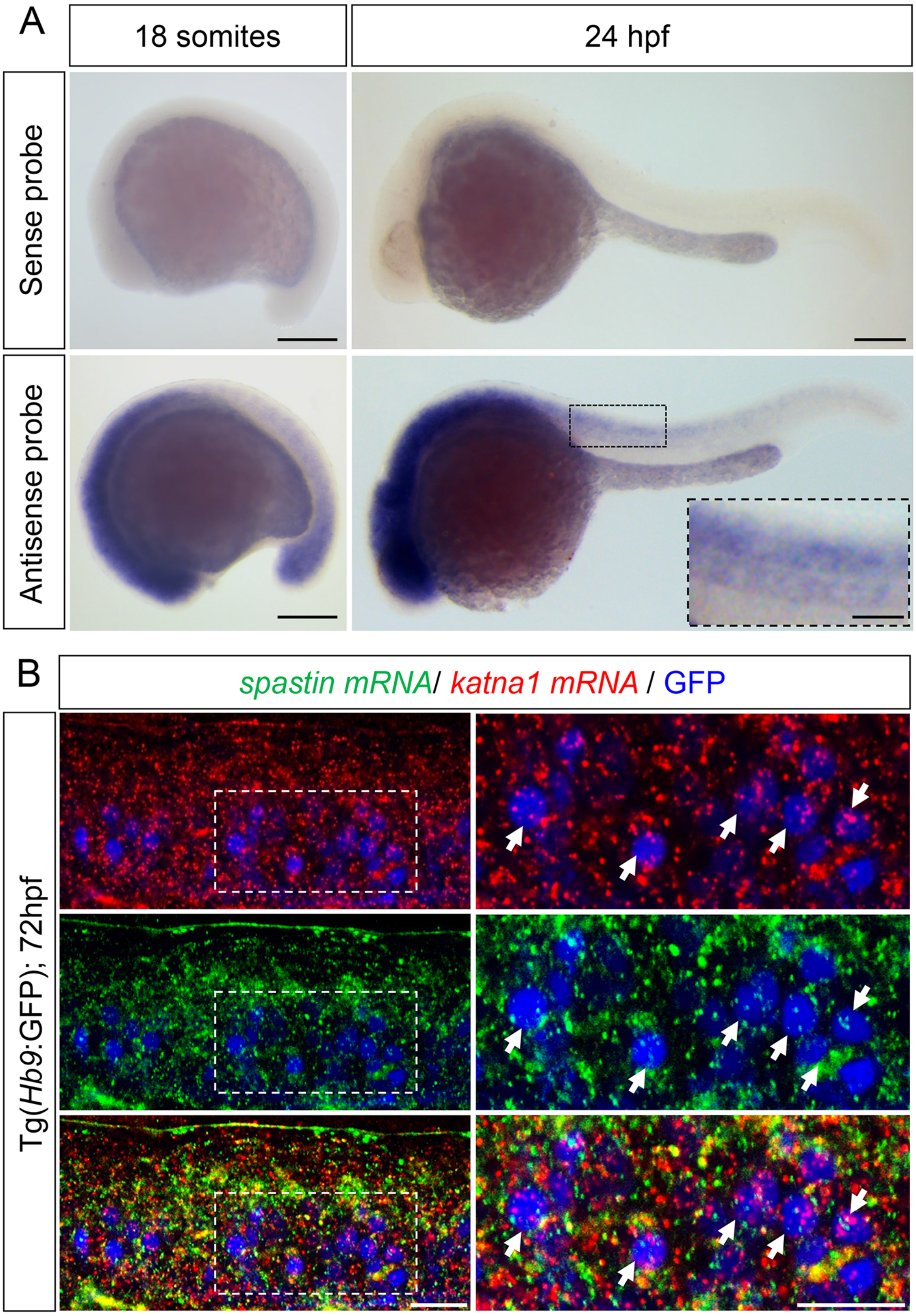

Figure EV3. *p60-katanin* and *spastin* transcripts are both expressed in developing spinal motor neurons.

(**A**) Whole-mount in situ hybridisation with *p60-katanin* sense (upper panel) and antisense (bottom panel) riboprobes at 18 somites and 24 h post-fertilisation (hpf). Lateral views of the embryos, anterior to the left. *P60-katanin* is highly enriched in the developing nervous system at both 18 somites and 24 hpf, two stages at which the axons of primary (pMN) and secondary (sMN) motor neurons exit the spinal cord to navigate towards their muscle targets. Scale bars: 200 µm. (**B**) *In toto* hybridisation chain reaction (HCR) on 72-hpf Tg(*Hb9*:GFP) larvae using zebrafish *spastin* and *katna1* probes. Lateral view of the trunk, anterior to the left. Right panels are higher magnifications of boxed region of the corresponding left panel. Arrows point at spinal motor neurons co-expressing *katna1* and *spastin* transcripts. Scale bars: 20 µm. Source data are available online for this figure

 © The Author(s)

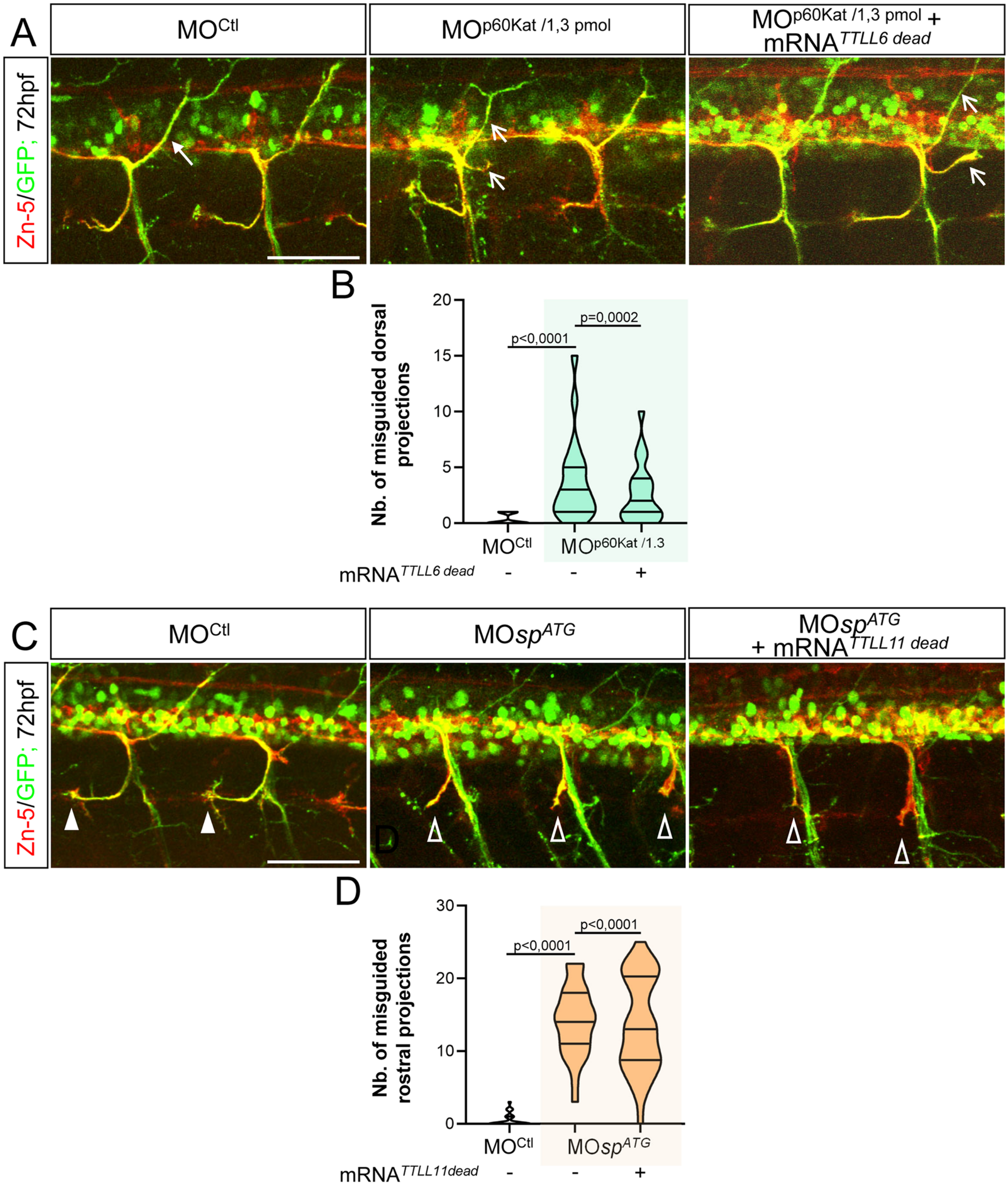
A
MOCtl
MOp60Kat /1,3 pmol
MOp60Kat /1,3 pmol + mRNATTLL6 dead
Zn-5/GFP; 72hpf
B
Nb. of misguided dorsal projections
p<0,0001
p=0,0002
20
15
10
5
0
MOCtl
MOp60Kat /1.3
mRNATTLL6 dead
- - +
C
MOCtl
MOspATG
MOspATG + mRNATTLL11 dead
Zn-5/GFP; 72hpf
D
Nb. of misguided rostral projections
p<0,0001
p<0,0001
30
20
10
0
MOCtl
MOspATG
mRNATTLL11dead
- - +

Figure EV4. Overexpression of a catalytic-dead variant of TTLL6 or TTLL11 respectively fails to rescue the axon pathfinding errors associated with p60-Katanin or Spastin partial knockdown.

(**A**) Immunolabelling of sMN axon tracts using Zn-5 and GFP antibodies in 72-hpf Tg(*Hb9*:GFP) larvae injected with MO^{Ctl} ($n = 29$), $MO^{p60Kat/1.3pmol}$ ($n = 21$) or co-injected with $MO^{p60Kat/1.3pmol}$ and the mRNA encoding a catalytic-dead variant of TTLL6 ($TTLL6^{dead}$) ($n = 23$). Full and empty arrows respectively point at normal and misguided dorsal projections. (**B**) Mean number of split/misguided dorsal nerves per larva. Quantifications were conducted on the larval set analysed in (**A**). (**C**) Immunolabelling of sMN axon tracts using Zn-5 and GFP antibodies in 72-hpf Tg(*Hb9*:GFP) larvae injected with MO^{Ctl} ($n = 27$), $MOsp^{ATG}$ ($n = 27$) or co-injected with $MOsp^{ATG}$ and the transcript encoding a catalytic-dead variant of TTLL11 ($TTLL11^{dead}$) ($n = 34$). Full and empty arrowheads indicate normal and misguided rostral projections, respectively. (**D**) Mean number of misguided rostral nerves per larva. Quantifications were conducted on the larval set analysed in (**B**). (**A**, **C**) Scale bars: 50 µm. (**B**, **D**) Non-blind quantifications were performed on 24 spinal hemisegments located around the yolk tube per larva. Analysed larvae were pooled from three independent experiments. Violin Plots; horizontal bars indicate the median ± the 1st and 3rd quartiles. Kruskal–Wallis ANOVA test with Dunn's post hoc test. *P* values are displayed on graphs. Source data are available online for this figure

 © The Author(s)

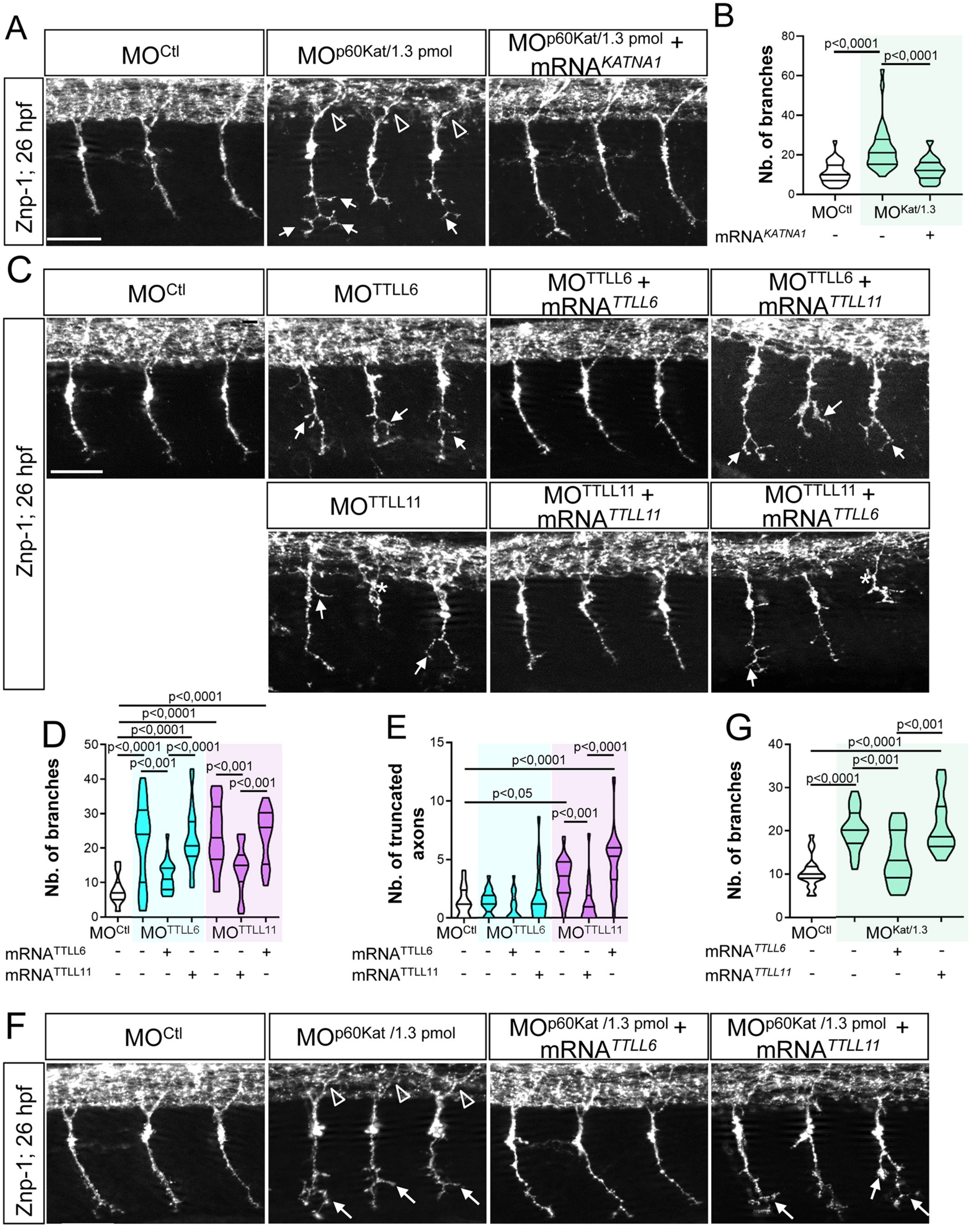
A
MOCtl
MOp60Kat/1.3 pmol
MOp60Kat/1.3 pmol + mRNAKATNA1
Znp-1; 26 hpf
B
p<0,0001
p<0,0001
Nb. of branches
MOCtl
MOKat/1.3
mRNAKATNA1 - - +
C
MOCtl
MOTTLL6
MOTTLL6 + mRNATTLL6
MOTTLL6 + mRNATTLL11
MOTTLL11
MOTTLL11 + mRNATTLL11
MOTTLL11 + mRNATTLL6
Znp-1; 26 hpf
D
p<0,0001
p<0,001
p<0,001
Nb. of branches
MOCtl MOTTLL6 MOTTLL11
mRNATTLL6 - - + - - - +
mRNATTLL11 - - - + - + -
E
p<0,0001
p<0,05
p<0,001
Nb. of truncated axons
MOCtl MOTTLL6 MOTTLL11
mRNATTLL6 - - + - - - +
mRNATTLL11 - - - + - + -
G
p<0,001
p<0,0001
Nb. of branches
MOCtl MOKat/1.3
mRNATTLL6 - - + -
mRNATTLL11 - - - +
F
MOCtl
MOp60Kat /1.3 pmol
MOp60Kat /1.3 pmol + mRNATTLL6
MOp60Kat /1.3 pmol + mRNATTLL11
Znp-1; 26 hpf

Figure EV5. TTLL6 also tunes p60-Katanin-driven pMN axon development.

(**A**) Immunolabelling of pMN axons in 26-hpf embryos injected with MO^{Ctl} ($n = 32$), $MO^{p60Kat/1.3pmol}$ ($n = 32$) or co-injected with $MO^{p60Kat/1.3pmol}$ and 120 pg of human *KATNA1* transcripts ($MO^{p60Kat/1.3pmol} + mRNA^{KATNA1}$, $n = 32$) using Znp-1 antibody. (**B**) Mean number of pMN axon branches per embryo analysed in the panel-A embryo set. (**C**) Immunodetection of pMN axons with Znp-1 antibody in 26-hpf embryos injected with MO^{Ctl} ($n = 19$), MO^{TTLL6} ($n = 22$), MO^{TTLL11} ($n = 19$) morpholinos or co-injected with MO^{TTLL6} or MO^{TTLL11} and mouse *TTLL6* or *TTLL11* mRNA ($MO^{TTLL6} + mRNA^{TTLL6}$, $n = 20$; $MO^{TTLL6} + mRNA^{TTLL11}$, $n = 21$; $MO^{TTLL11} + mRNA^{TTLL11}$, $n = 19$; $MO^{TTLL11} + mRNA^{TTLL6}$, $n = 18$). (**D**, **E**) Mean number of CaP pMN branches (**D**) and truncated CaP axons per embryo analysed in the panel-C embryo set. (**F**) Immunostaining of pMN axons with Znp-1 antibody in 26-hpf embryos injected with MO^{Ctl}, ($n = 20$), $MO^{p60Kat/1.3pmol}$ ($n = 21$) or co-injected with $MO^{p60Kat/1.3pmol}$ and mouse *TTLL6* ($MO^{p60Kat/1.3pmol} + mRNA^{TTLL6}$, $n = 19$) or *TTLL11* mRNA ($MO^{p60Kat/1.3pmol} + mRNA^{TTLL11}$, $n = 16$). (**G**) Mean number of CaP pMN branches per embryo analysed in the panel-F embryo set. (**A**, **C**, **F**) Arrows and asterisk respectively indicate hyper-branched and truncated ventrally projecting pMN CaP axons. Empty arrowheads indicate normal dorsally projecting pMN MiP axons. Scale bars: 25 µm. (**B**, **D**, **E**, **G**) Non-blind quantifications were performed on 24 spinal hemisegments located around the yolk tube per embryo. Analysed larvae were pooled from three independent experiments. Violin Plots; horizontal bars indicate the median ± the 1st and 3rd quartiles. One-way ANOVA test with Bonferroni's post test (**D**, **G**) or Kruskal–Wallis ANOVA test with Dunn's post test (**B**, **E**). *P* values are displayed on graphs. Source data are available online for this figure

© The Author(s)

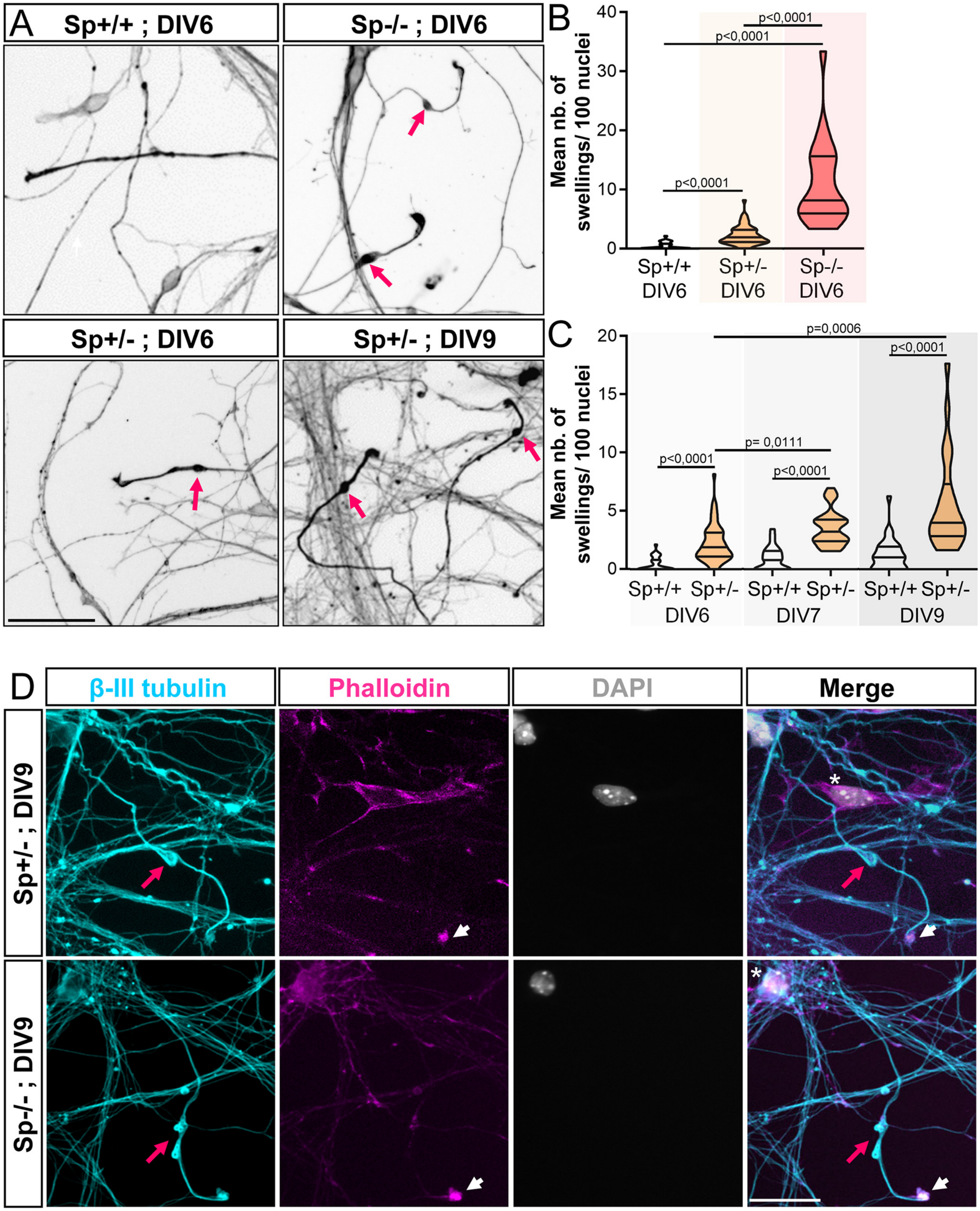
A
Sp+/+ ; DIV6
Sp-/- ; DIV6
Sp+/- ; DIV6
Sp+/- ; DIV9
B
Mean nb. of swellings/ 100 nuclei
p<0,0001
p<0,0001
p<0,0001
Sp+/+ DIV6
Sp+/- DIV6
Sp-/- DIV6
C
p=0,0006
p<0,0001
p= 0,0111
p<0,0001
p<0,0001
Sp+/+ Sp+/- DIV6
Sp+/+ Sp+/- DIV7
Sp+/+ Sp+/- DIV9
D
β-III tubulin
Phalloidin
DAPI
Merge
Sp+/- ; DIV9
Sp-/- ; DIV9

Figure EV6. Mouse Sp + /- cortical neurons exhibit a significant number of axonal swellings.

(**A**) Mouse Sp + /+, Sp + /- and Sp-/- cultured cortical neurons immunolabelled with a βIII-tubulin antibody at different days in vitro (DIV). Pink arrows point at axonal swellings. Scale bars: 50 µm. (**B**, **C**) Mean number of axonal swellings per 100 nuclei. At least 2500 neurons from two independent experiments were analysed in unblind manner per condition. Violin Plots; horizontal bars indicate the median ± the 1st and 3rd quartiles. Kruskal–Wallis ANOVA test with Dunn's post hoc test. *P* values are displayed on graphs. (**D**) Primary culture of Sp + /- and Sp-/- cortical neurons immunolabelled at DIV9 with βIII-tubulin antibodies, F-actin probes (Phalloidin, pink) and DAPI (grey). Axonal swellings (pink arrows) of Sp + /- exhibit the same characteristic features as those described in Sp-/- cultures. They are (i) always located close to the growth cone (arrowheads), (ii) their diameter is at least 2 to 3 times larger than the diameter of the axon shaft, (iii) they are always strongly labelled by tubulin antibodies and (iv) are always negative for DAPI staining (asterisk). Scale bars: 25 µm. Source data are available online for this figure

 © The Author(s)
